# Selective *ortho*‐Functionalization of Adamantylarenes Enabled by Dispersion and an Air‐Stable Palladium(I) Dimer

**DOI:** 10.1002/anie.202001326

**Published:** 2020-03-17

**Authors:** Indrek Kalvet, Kristina Deckers, Ignacio Funes‐Ardoiz, Guillaume Magnin, Theresa Sperger, Marius Kremer, Franziska Schoenebeck

**Affiliations:** ^1^ Institute of Organic Chemistry RWTH Aachen University Landoltweg 1 52074 Aachen Germany

**Keywords:** chemoselectivity, cross-coupling, DFT calculations, homogeneous catalysis, palladium

## Abstract

Contrary to the general belief that Pd‐catalyzed cross‐coupling at sites of severe steric hindrance are disfavored, we herein show that the oxidative addition to C−Br *ortho* to an adamantyl group is as favored as the corresponding adamantyl‐free system due to attractive dispersion forces. This enabled the development of a fully selective arylation and alkylation of C−Br *ortho* to an adamantyl group, even if challenged with competing non‐hindered C−OTf or C−Cl sites. The method makes use of an air‐stable Pd^I^ dimer and enables straightforward access to diversely substituted therapeutically important adamantylarenes in 5–30 min.

## Graphical abstract

**It takes two**: Contrary to the general belief that Pd‐catalyzed cross‐couplings at sites of severe steric hindrance are disfavored, it is shown that the oxidative addition to C−Br *ortho* to an adamantyl group is as favored as the corresponding adamantyl‐free system due to attractive dispersion forces. This enabled the development of a fully selective arylation and alkylation of C−Br *ortho* to an adamantyl group using an air‐stable Pd^I^ dimer.

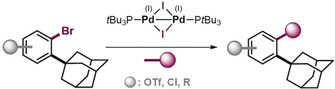

Owing to its exceptional liphophilicity and stability‐enhancing properties, the adamantyl group has found widespread applications in materials science,^1^ medicinal chemistry, and drug development.^2^ In fact, the only other hydrocarbon moiety that has been similarly successful in generating active therapeutics is the methyl group.^2^ Adamantane derivatives are used in fighting viral infections, such as influenza, herpes, hepatitis C, HIV, as well as malaria or Parkinson's disease, and as retinoid antibiotics (see Figure 1).^2^, ^3^ However, synthetic access to densely functionalized adamantyl‐group‐containing molecules is still challenging,^4^ and in particular, diversification through arylation or alkylation *ortho* to the adamantyl group is currently out of reach. However, the future discovery of superior therapeutics or antibiotics would vastly benefit from synthetic methods that allow access to a larger chemical space of diversely substituted adamantyl derivatives. Ideally, this is achieved in a modular and rapid fashion that enables the introduction of various potential arene substituents late in the synthesis and with complete site‐control, since the nature of the substituents and their relative positioning will ultimately control the function of the final target molecule. While methods for the introduction of the 1‐adamantyl group to an arene have significantly advanced in recent years, particularly due to successes in devising radical‐based or organometallic methods,4c, 4e, 4f, 4g, 4h, 5 these approaches predominantly deliver the adamantly group in the *meta* or *para* position relative to an alternative substituent in the arene, but not in the *ortho* position. Similarly, although electrophilic adamantylations (Friedel–Crafts) could in principle deliver an *ortho* substitution pattern (along with alternative regioisomers),^6^ the process requires harsh conditions (concentrated/strong acids, elevated temperature) and will strongly depend on the electronic bias of the particular substrate, thus precluding late‐stage applications.^7^

**Figure 1:**
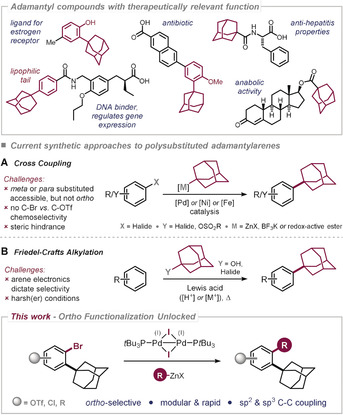
Medicinally relevant adamantyl compounds, current synthetic approaches to adamantylarenes, and this work.

We therefore set out to address the *ortho*‐functionalization challenge and focused on *ortho*‐bromo adamantylarenes as a coupling motif. More specifically, to maximize the potential for downstream diversification, we initially targeted a poly(pseudo)halogenated adamantyl arene as a modular platform. The selective manipulation of C−Br versus C−OTf, for example, would ultimately deliver a library of diversely substituted adamantyl derivatives with an *ortho*/*meta* relationship that is not yet accessible in a general manner. The key requirements for success of this strategy are 1) that selective C−Br versus C−OTf functionalization can be realized and 2) that coupling *ortho* to the large adamantyl group is possible.

The adverse impact of steric hindrance on the efficiency of Pd‐catalyzed cross‐coupling has been widely documented.^8^ For example, although 4‐bromoaryl triflate can be selectively coupled at C−Br with a [PdCl_2_{P(*o*‐tol)_3_}_2_]‐catalyzed Kumada reaction, the introduction of two relatively small methyl substituents *ortho* to the C−Br (at the 3‐ and 5‐positions) resulted in erosion of selectivity and mixtures of products.^9^ The vast majority of reports conclude that the oxidative addition step controls the site‐selectivity^10^ and that this step is most likely inhibited by steric hindrance.^11^

In this context, we wondered about the feasibility of oxidative addition *ortho* to the adamantyl group. Although the adamantyl group is large, it has also been ascribed to be a privileged dispersion donor.^12^ Attractive dispersion forces have previously been found to outweigh steric effects, thus counterintuitively stabilizing more crowded molecules over their less crowded counterparts.^13^

We focused on compound **1** (see Scheme 1), which bears a C−Br *ortho* to the adamantyl and allows for further *meta*‐derivatization through C−OTf coupling. The methoxy group mimics a potential C−O linkage to biomolecules.^14^

**Scheme 1:**
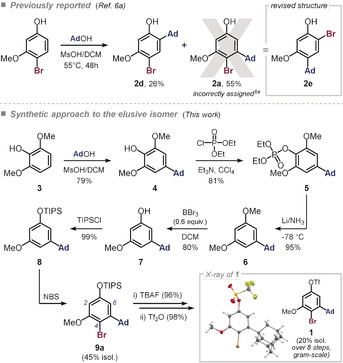
Structural revision of previously claimed *ortho*‐bromo‐adamantylarene (**2 a**) and synthesis of 4‐Br‐5‐methoxy‐3‐adamantyl‐phenyl triflate (**1**) from 1,3‐dimethoxyphenol **3**. See the Supporting Information for detailed reaction conditions.

As a theoretical exercise, we computationally assessed the impact of the adamantyl group on the oxidative addition step, utilizing Pd^0^(P*t*Bu_3_)_2_ as a model system, which should be particularly prone to engage in attractive C−H⋅⋅⋅H−C dispersion interaction with the adamantyl group.^15^ To this end, we calculated the activation free‐energy barrier for the oxidative addition of 2‐adamantyl‐6‐methoxybromoarene (Figure 2, C) versus the corresponding adamantyl‐free systems (Figure 2 A,B). The lowest energy pathway of oxidative addition involves initial ligand loss, followed by oxidative addition to C−Br by the monophosphine Pd^0^(P*t*Bu_3_) complex.^15^, 17b, 17c, 30 The barriers for oxidative addition to Ar‐Br were compared using DFT methods^16^ with and without dispersion corrections. The results are illustrated in Figure 2.

**Figure 2:**
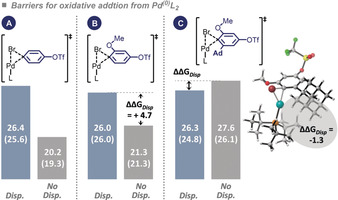
Computational study of activation free energies and enthalpies (in parentheses) for C−Br oxidative addition relative to Pd(P*t*Bu_3_)_2_ in kcal mol^−1^; calculated at the CPCM (THF) B3LYP‐D3/Def2TZVP// B3LYP‐D3/6‐31G(d)/LANL2DZ level of theory. See the Supporting Information for additional computational data with alternative methods on this trend, including D4 dispersion results.^17^, ^30^

Interestingly, while the classical DFT methods (that do not account for dispersion) predict activation barriers in accord with expected steric effects, that is, with barriers A<B<C, inclusion of dispersion in turn predicts roughly the same activation free‐energy barriers for A, B, and C of approximately 26 kcal mol^−1^,^17^ thus suggesting that attractive dispersion between the adamantyl and *tert*‐butyl substituent of the Pd‐catalyst outweigh steric clashes. As such, although computational challenges exist in unambiguously capturing this phenomenon relative to solute–solvent dispersive interactions,12a, 15, 18 these calculations suggest that, contrary to the general belief of steric effects on the oxidative addition step, the adamantyl substituent should not impede oxidative addition.

We therefore next set out to experimentally target compound **1**. The required substitution pattern has so far been synthetically inaccessible. There is one patent that claims the synthesis of 3‐(adamantan‐1‐yl)‐4‐bromo‐5‐methoxyphenol (**2 a**) in a single step (see Scheme 1).6a However, our follow‐up investigation revealed that these claims are incorrect and the alternative isomer, 4‐(adamantan‐1‐yl)‐2‐bromo‐5‐methoxyphenol (**2 e**) was instead generated. As such, previous C−Br coupling attempts of starting materials prepared by this method instead yielded derivatives of **2 e**.9b, 19, 26b We hence embarked on developing a synthetic route to **1**, which would establish the 3‐(adamantan‐1‐yl)‐2‐bromo‐phenol substitution pattern for the first time.

Scheme 1 presents our successful synthesis. The 1‐adamantyl group was introduced to 1,3‐dimethoxyphenol **3** through Friedel–Crafts alkylation with 1‐adamantanol.^20^ The free OH group of **4** was then protected as a diethyl phosphate ester (**5**) and subsequently reduced under Li/NH_3_ conditions to give 5‐adamantylresorcinol dimethyl ether **6**. Selective removal of one of the methyl groups was accomplished with BBr_3_, followed by the introduction of a TIPS protecting group. This enabled predominant reaction at the 6‐position in the electrophilic bromination with NBS.^21^ The structure of **1** was unambiguously confirmed through single‐crystal X‐ray diffraction analysis (Scheme 1),^22^ and 2D NMR analyses were conducted on all intermediates and the target compound **1**.^23^

We subsequently set out to study the functionalization of **1**. In the context of Pd^0^/Pd^II^ catalysis, the relative reactivity of aryl triflates and bromides is frequently referred to as roughly the same,^24^ and selectivity has therefore historically been a result of a subtle interplay of reaction conditions, catalyst, coupling partner, and the steric and electronic effects imposed by the substrate.9a, 10e, 10f, 24, 25 By contrast, we recently established a substrate‐independent and a priori predictable C_sp2_−C_sp2_ and C_sp2_−C_sp3_ functionalization exclusively at C−Br in competition with the C−OTf and C−Cl sites.9b, 26 Key to this exquisite selectivity was the employment of an air‐ and moisture stable Pd^I^ dimer that facilitated the couplings within 5 min at room temperature.^27^ As such, we envisioned that if the privileged reactivity for C−Br also applies to the adamantyl motif **1**, we would then be in a position to diversify C−Br at will, leaving possibilities for functionalization of the remaining C−OTf with any type of method.

When we explored the functionalization of **1** with methyl‐ or TMS‐CH_2_‐organozinc reagents in toluene with the air‐stable Pd^I^ dimer (5 mol %), we saw efficient conversion into the corresponding coupling products arising from exclusive C−Br coupling (**10 a** and **10 b**) within 5 min reaction time at r.t. (see Table 1). Similarly, arylation and *n*‐butylation were equally selective for C−Br and left C−OTf untouched, despite the need for slightly elevated temperatures (50 °C), slower addition rates (12 min), and increased organozinc (5–7 equiv) as well as catalyst (10 mol %) loading to reach high yields. Under these conditions we were able to introduce phenyl (**10 d**, Table 1), 2‐thienyl (**10 f**), *n*‐butyl (**10 c**), 4‐chlorophenyl (**10 e**), and 4‐methoxyphenyl (**10 g**) groups under exclusive coupling at the C−Br site, as unambiguously confirmed also through X‐ray crystallographic analysis of **10 g** (see Table 1).

**Table 1:** Pd^I^ dimer catalyzed Br‐selective cross‐coupling reactions with **1**.^28^ 
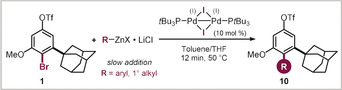

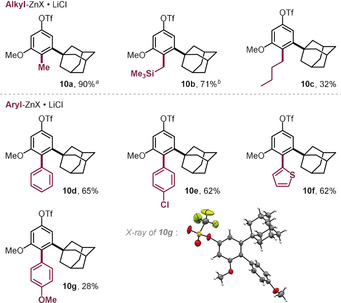

Reaction conditions: **1** (0.1 mmol), organometallic reagent (0.7 mmol in THF),^29^ Pd^I^ iodo dimer (0.01 mmol), toluene (0.6 mL), 50 °C, slow addition of organometallic reagent over 12 minutes. [a] **1** (0.05 mmol), organometallic reagent (0.15 mmol in THF), Pd^I^ iodo dimer (0.0013 mmol), toluene (0.3 mL), r.t., slow addition of organometallic reagent over 4 minutes. [b] As in [a], with 0.25 mmol of organometallic reagent.

As such, both arylation and alkylation could be accomplished selectively *ortho* to the adamantyl group, even in the presence of the additional and deactivating methoxy substituent. With this stringent test passed, we next investigated the wider scope with the functionalization of a range of 2‐bromo adamantylarenes (**11**, see Table 2). We were able to couple the C−Br within 5 min at room temperature, and successfully introduced the alkyl substituents methyl, butyl, cyclopropyl, and CH_2_TMS (**12 a**–**d**, Table 2). Arylations were similarly effective, and electron‐rich (**12 i**,**j**) as well as electron‐deficient (**12 h**) groups coupled equally efficiently. Even the introduction of the more hindered 2‐methyl phenyl (**12 f**) and heterocyclic thiophene (**12 g**) substituents proceeded smoothly.

**Table 2:** Pd^I^ dimer catalyzed Br‐selective cross‐coupling reactions with **11**. 
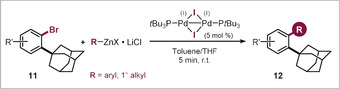

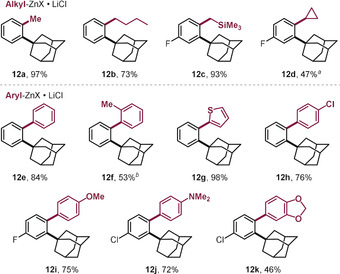

Reaction conditions: **11** (0.2 mmol), organometallic reagent (0.3 mmol in THF),^29^ Pd^I^ iodo dimer (0.01 mmol), toluene (0.8 mL), r.t. [a] Organomagnesium was used. [b] Yield obtained by quantitative ^1^H NMR analysis of the crude product.

In summary, we have demonstrated C−Br selective *ortho*‐functionalization in adamantylarenes, while leaving the less sterically encumbered C−OTf or C−Cl sites fully untouched. Our computational studies indicated that oxidative addition to C−Br *ortho* to the adamantyl group is just as facile as to the corresponding adamantyl‐free arene, thus suggesting that attractive P*t*Bu_3_⋅⋅⋅adamantyl dispersion forces override steric repulsion and stabilize the transition state for oxidative addition, which is at odds with current expectations in metal‐catalyzed cross‐coupling chemistry. Given the pronounced therapeutic potential of adamantly‐containing motifs, we anticipate widespread interest in the herein reported unlocked *ortho* diversification method in adamantylarenes.

## Conflict of interest

The authors declare no conflict of interest.

## Supporting information

As a service to our authors and readers, this journal provides supporting information supplied by the authors. Such materials are peer reviewed and may be re‐organized for online delivery, but are not copy‐edited or typeset. Technical support issues arising from supporting information (other than missing files) should be addressed to the authors.

Supplementary
