# Supplementary material for: Selective ortho‐Functionalization of Adamantylarenes Enabled by Dispersion and an Air‐Stable Palladium(I) Dimer
Source: Angew Chem Int Ed Engl. 2020 Mar 17;59(20):7721–5. doi: 10.1002/anie.202001326 (PMC7317867; doi:10.1002/anie.202001326)
Supplement: Supplementary file 1 — Supplementary [file ANIE-59-7721-s001.pdf]

Angewandte Chemie
Eine Zeitschrift der Gesellschaft Deutscher Chemiker

Supporting Information

Selective *ortho*-Functionalization of Adamantylarenes Enabled by Dispersion and an Air-Stable Palladium(I) Dimer

*Indrek Kalvet, Kristina Deckers, Ignacio Funes-Ardoiz, Guillaume Magnin, Theresa Sperger, Marius Kremer, and Franziska Schoenebeck**

anie_202001326_sm_miscellaneous_information.pdf

Supporting Information

Contents

1. **General Information 3**

2. **Synthetic procedures 4**

    2.1. Synthesis of adamantylarenes 4

        2.1.1. Synthesis of 5-(adamantan-1-yl)-4-bromo-3-methoxyphenyl triflate (**1**) 4

        2.1.2. Studies into the bromination of 5-adamantyl-3-methoxyphenol derivatives 12

        2.1.3. Synthesis of 2-bromo adamantylarenes (**11**) 13

    2.2. Cross-coupling reactions 15

        2.2.1. Optimization of reaction conditions for the cross-coupling of **1** with organozinc reagents .. 15

        2.2.2. General cross-coupling procedures 16

        2.2.3. Characterization data for cross-coupling products 17

3. **Crystallographic data 29**

4. **Computational details 30**

    4.1. Benchmarking of the Methodology 30

    4.2. Cartesian Coordinates of Calculated compounds 32

    4.3. Functional Benchmarking Single Point Energies 40

5. **NMR spectra 41**

6. **References 103**

1. General Information

^{1}H, ^{13}C and ^{19}F NMR spectra were recorded either on Varian V-NMRS 600 and Varian V-NMRS 400 spectrometers. ^{1}H and ^{13}C spectra are referenced to residual solvent signals; $CDCl_3$ 7.26 ppm for ^{1}H and 77.0 ppm for ^{13}C; CD_2Cl_2 5.32 ppm for ^{1}H and 53.84 ppm for ^{13}C.[1] Chemical shifts (δ) of ^{19}F NMR spectra are reported in ppm relative to trifluorotoluene (-62.78 ppm). Coupling constants (*J*) are reported in Hz and coupling patterns are described as br = broad, s = singlet, d = doublet, t = triplet, q = quartet, hept = heptet, m = multiplet. High resolution mass spectra (HRMS) were recorded on Thermo Scientific LTQ Orbitrap XL (ESI) or Finnigan MAT 95 (EI) spectrometer in positive ion mode. Melting points were measured with a LLG Labware MPM-H2 apparatus. Flash column chromatography was performed with Merck silica gel 60 (35–70 mesh). Thin layer chromatography (TLC) analyses were performed with aluminum sheets silica gel 60 F254 from Merck with detection by UV light, $KMnO_4$ or phosphomolybdic acid (PMA) staining. Preparative HPLC was performed on a Gilson-Abimed HPLC (employing UV detector model 117) using a LiChrosorb Si 60 column (porosity 7 µm, 250 x 25 mm).

Anhydrous toluene, THF and DCM were dried by solvent purification system (Innovative Technology PS-MD-5). Unless stated otherwise, other anhydrous solvents as well as all starting materials, ligands and Pd-complexes were commercially available and used as received. Solvents used for column chromatography (pentane, hexane, ethyl acetate, DCM, acetone, Et_2O) were received in technical grade and distilled prior to use. $Pd^{(I)}$-I-dimer was prepared according to its corresponding literature procedure.[2]

2. Synthetic procedures

2.1. Synthesis of adamantylarenes

*2.1.1. Synthesis of 5-(adamantan-1-yl)-4-bromo-3-methoxyphenyl triflate (**1**)*

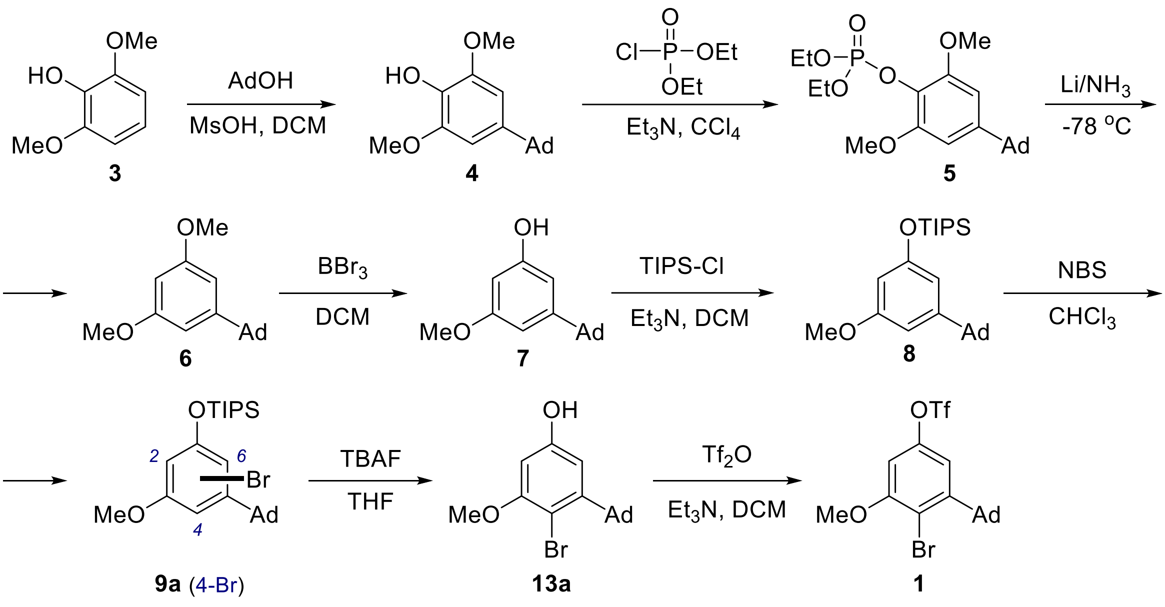

4-(adamantan-1-yl)-2,6-dimethoxyphenol (4)

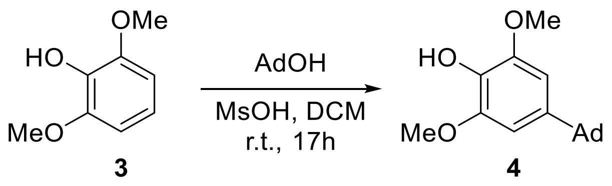

1-adamantanol (2.72 g, 17.8 mmol) was dissolved in 15 mL of methanesulfonic acid. Subsequently, a DCM (17 mL) solution of 1,3-dimethoxyphenol (2.5 g, 16.2 mmol) was slowly (over 1 hour) added and the reaction stirred at room temperature for 17 hours. Thereafter, the reaction was diluted with 40 mL of DCM, stirred for another 10 minutes, and poured into ice-water. After 15 minutes of stirring, the layers were separated and the aqueous layer was further extracted twice with DCM. The organic phase was washed with H_2O, sat. aq. $NaHCO_3$ and brine, and dried over $MgSO_4$. Volatiles were removed under reduced pressure, and the crude material was purified by flash column chromatography (7:3 Hexane/Acetone) to yield the title compound as a white solid. 3.69 g (79%). R_f = 0.39 (7:3 Hexane/Acetone). ^{1}H NMR (400 MHz, $CDCl_3$) δ 6.59 (s, 2H), 5.36 (s, 1H), 3.90 (s, 6H), 2.14 – 2.03 (m, 3H), 1.92 – 1.86 (m, 6H), 1.83 – 1.71 (m, 6H). ^{13}C NMR (101 MHz, $CDCl_3$) δ 146.6, 142.9, 132.6, 101.9, 56.3, 43.5, 36.8, 36.2, 29.0. MS (70eV, EI): *m/z* (%): 288 (100) [M^+], 231 (8), 199 (14), 194 (11), 91 (7). These data are in agreement with those reported previously in the literature.[3]

4-(adamantan-1-yl)-2,6-dimethoxyphenyl diethyl phosphate (5)

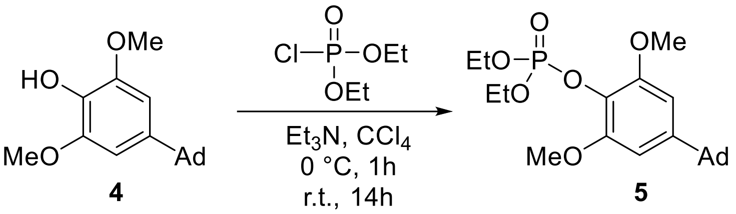

4-(adamantan-1-yl)-2,6-dimethoxyphenol (1.851 g, 6.418 mmol, 1.0 equiv.) was dissolved in 9 mL of CCl_4 and cooled to 0 °C. Followingly, diethylchlorophosphate (1.11 mL, 7.70 mmol, 1.2 equiv.) was added, followed by slow (over 30 minutes) addition of Et_3N (1.25 mL, 8.99 mmol, 1.4 equiv.) The reaction stirred at 0 °C for 1 hour and at room temperature for another 14 hours. It was then diluted with excess of DCM and washed with H_2O, 2 M aq. NaOH, 2M aq. HCl, H_2O and brine, and dried over $MgSO_4$. Volatiles were removed under reduced pressure, and the crude material was purified by flash column chromatography (4:1 Pentane/Acetone) to yield the title compound as a white solid. 2.20 g (81%). R_f = 0.35 (3:1 Hexane/Acetone). ^{1}H NMR (400 MHz, $CDCl_3$) δ 6.57 (s, 2H), 4.30 (p, J = 7.1 Hz, 4H), 3.86 (s, 6H), 2.12 – 2.04 (m, 3H), 1.89 – 1.85 (m, 6H), 1.83 – 1.70 (m, 6H), 1.38 (t, J = 7.1 Hz, 6H). ^{13}C NMR (101 MHz, $CDCl_3$) δ 151.3 (d, $J_{C\text{-}P}$ = 3.5 Hz), 148.6 (d, $J_{C\text{-}P}$ = 1.9 Hz), 127.5 (d, $J_{C\text{-}P}$ = 7.7 Hz), 102.2 (d, $J_{C\text{-}P}$ = 1.6 Hz), 64.1 (d, $J_{C\text{-}P}$ = 6.1 Hz), 56.1, 43.3, 36.7, 36.5, 28.9, 16.1 (d, $J_{C\text{-}P}$ = 7.5 Hz). ^{31}P NMR (121 MHz, $CDCl_3$) δ -5.31. These data are in agreement with those reported previously in the literature.[3]

1-(adamantan-1-yl)-3,5-dimethoxybenzene (6)

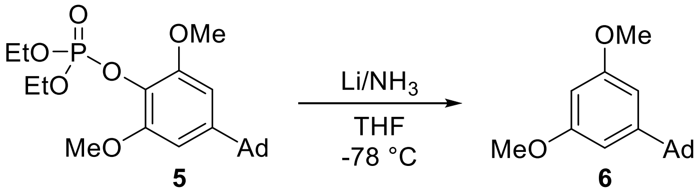

Performed in analogy to a published procedure.[3] Under Ar atmosphere, 40-50 mL of liquid NH_3 was condensed to a three-necked flask cooled to -78 °C. Lithium (375 mg, 4 mmol, 8 equiv.) was added to NH_3 and allowed to stir for 10 minutes. 4-(adamantan-1-yl)-2,6-dimethoxyphenyl diethyl phosphate (2.865 g, 6.750 mmol, 1 equiv.) was dissolved in 20 mL of anhydrous THF and slowly added over 30 minutes. The reaction was further stirred for 2 hours at -78 °C. Solid NH_4Cl was then added to quench the excess lithium, water-saturated Et_2O was carefully added to the mixture, it was warmed to r.t. and allowed to stand for 2 hours to let the NH_3 evaporate. The obtained residue was extracted three times with Et_2O and the collected organic phases washed with H_2O, 1M NaOH, H_2O, brine and dried over $MgSO_4$. Volatiles were removed under reduced pressure, and the crude material was purified by flash column chromatography (7:1 Hexane/Acetone) to yield the title compound as a white solid. 1.75 g (95%). R_f = 0.69 (5:1 Hexane/Acetone). ^{1}H NMR (400 MHz, $CDCl_3$) δ 6.54 (d, J = 2.2 Hz, 2H), 6.33 – 6.30 (m, 1H), 3.80 (s, 6H), 2.11 – 2.01 (m, 3H), 1.93 – 1.87 (m, 6H), 1.83 – 1.70 (m, 6H). ^{13}C NMR (101 MHz, $CDCl_3$) δ 160.5, 154.1, 103.5, 97.0, 55.2, 43.2, 36.8, 36.5, 29.0. MS (70eV, EI): *m/z* (%): 272 (100) [M^+], 215 (20). These data are in agreement with those reported previously in the literature.[3]

Alternatively, the addition of THF solution of the starting material to liquid NH_3, followed by slow addition of lithium (to maintain the blue color of the solution) was equally efficient in our hands.

5-(adamantan-1-yl)-3-methoxyphenol (7)

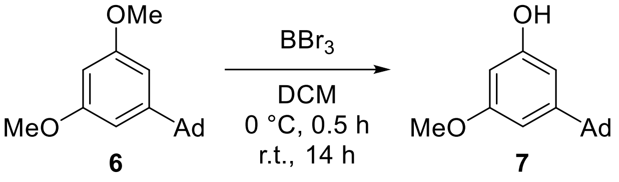

Performed in analogy to a published procedure.[3] Under Ar, atmosphere, 1-(adamantan-1-yl)-3,5-dimethoxybenzene (1.748 g, 6.417 mmol, 1.0 equiv.) was dissolved in 26 mL of DCM and cooled to 0 °C. Followingly, a 1M solution of BBr_3 in DCM (3.85 mL, 3.85 mmol, 0.6 equiv.) was added and the reaction stirred at 0 °C for 30 minutes and at room temperature for another 14 hours. DCM was then removed under reduced pressure and the residue taken up in EtOAc, washed with sat. aq. $NaHCO_3$ and dried over $MgSO_4$. Volatiles were removed under reduced pressure, and the crude material was purified by flash column chromatography (4:1 → 2:1 Pentane/Et_2O) to yield the title compound as a white solid. 1.33 g (80%). R_f = 0.26 (4:1 Pentane/Et_2O). M.p. 86 - 87 °C. ^1H NMR (600 MHz, $CDCl_3$) δ 6.53 – 6.51 (m, 1H), 6.45 – 6.43 (m, 1H), 6.26 – 6.24 (m, 1H), 4.71 (s, 1H), 3.78 (s, 3H), 2.09 – 2.06 (m, 3H), 1.88 – 1.86 (m, 6H), 1.81 – 1.70 (m, 6H). ^{13}C NMR (151 MHz, $CDCl_3$) δ 160.7, 156.3, 154.6, 104.6, 104.0, 98.2, 55.2, 43.1, 36.8, 36.3, 28.9. MS (70eV, EI): *m/z* (%): 258 (100) [M^+], 201 (28), 164 (8). HRMS (ESI) calculated for $C_{17}H_{23}O_2$: 259.1693 $[M+H]^+$, found: 259.1690.

(5-(adamantan-1-yl)-3-methoxyphenoxy)triisopropylsilane (8)

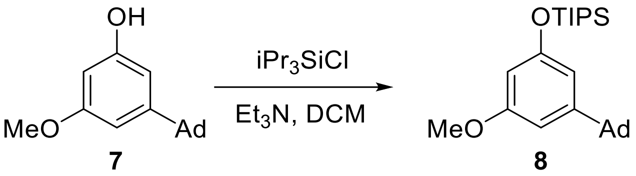

Performed according to a published procedure.[4] Under Ar, atmosphere, 5-(adamantan-1-yl)-3-methoxyphenol (1.95 g, 7.532 mmol, 1.0 equiv.) was dissolved in 10 mL of DCM and cooled to 0 °C. Et_3N (1.1 mL, 7.909 mmol, 1.05 equiv.) was added to the solution, followed by drop-wise addition (over 30 minutes) of triisopropylsilyl chloride (1.64 mL, 7.683 mmol, 1.02 equiv.). The reaction was allowed to warm to room temperature and was left stirring for 14 hours. Ice-water was added to the mixture, followed by extraction with DCM. The organic phase was washed with sat. aq. $NaHCO_3$ and dried over $MgSO_4$. Volatiles were removed under reduced pressure, and the crude material was purified by flash column chromatography (10:1 Pentane/Et_2O) to yield the title compound as a colorless oil. 3.11 g (99%). R_f = 0.41 (9:1 Pentane/DCM). ^1H NMR (600 MHz, $CDCl_3$) δ 6.51 – 6.50 (m, 1H), 6.50 – 6.49 (m, 1H), 6.29 – 6.27 (m, 1H), 3.77 (s, 3H), 2.07 (s, 3H), 1.88 – 1.85 (m, 6H), 1.76 (q, J = 12.1 Hz, 6H), 1.25 (q, J = 7.4 Hz, 3H), 1.11 (d, J = 7.4 Hz, 18H). ^{13}C NMR (151 MHz, $CDCl_3$) δ 160.2, 156.8, 153.7, 109.4, 104.0, 102.6, 55.1, 43.1, 36.8, 36.3, 28.9, 18.0, 12.7. HRMS (ESI) calculated for $C_{26}H_{43}O_2Si$: 415.3027 $[M+H]^+$, found: 415.3021.

(Bromo-5-(adamantan-1-yl)-3-methoxyphenoxy)triisopropylsilane (9)

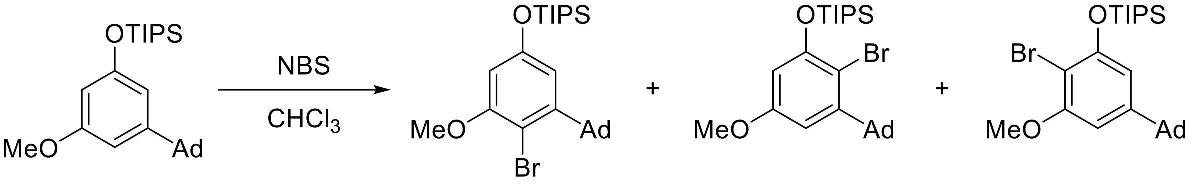

8 **9a**, 45% **9b**, 39% **9c**, 14%

(5-(adamantan-1-yl)-3-methoxyphenoxy)triisopropylsilane (744 mg, 1.794 mmol, 1.0 equiv.) and N-bromosuccinimide (319 mg, 1.794 mmol, 1.0 equiv.) were dissolved in DCM (36 mL) and stirred and r.t. for 15 hours. Thereafter, H_2O was added and the layers separated. Aqueous phase was further washed with DCM and the combined organic layers were washed with brine and dried over $MgSO_4$. ^1H NMR analysis of the crude mixture indicated the formation of three isomers (see scheme above). Isomer **9b** was first separated by flash column chromatography (Pentane/DCM 10:1). While most of isomer **9a** could be isolated by second column chromatography run (with Pentane/DCM 6:1), clean separation of isomers **9a** and **9c** turned out to be possible only by using preparative HPLC (Pentane/Et_2O 9:1).

Alternatively, the isomers **9a** and **9c** can be left unseparated at this stage and carried over to the next step. We found this approach to be slightly more convenient due to easier separation of these isomers after the removal of the TIPS group.

(5-(adamantan-1-yl)-4-bromo-3-methoxyphenoxy)triisopropylsilane (9a): White solid. 394 mg (45%). R_f = 0.54 (5:1 Hexane/DCM). M.p. 97 - 98 °C.

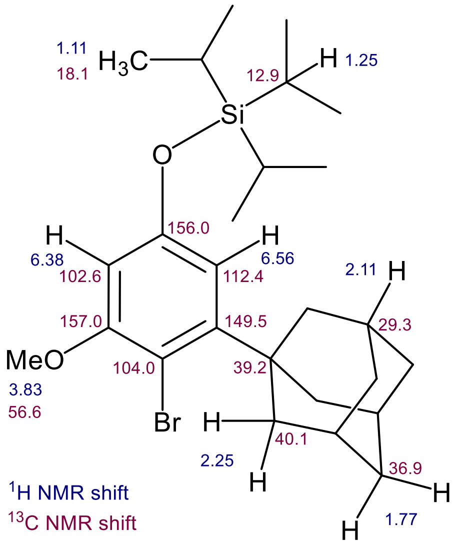

^1H NMR (600 MHz, $CDCl_3$) δ 6.57 (d, J = 2.6 Hz, 1H), 6.38 (d, J = 2.6 Hz, 1H), 3.83 (s, 3H), 2.25 (s, 6H), 2.11 (s, 3H), 1.83 – 1.72 (m, 6H), 1.31 – 1.21 (m, 3H), 1.11 (d, J = 7.5 Hz, 18H).

^{13}C NMR (151 MHz, $CDCl_3$) δ 156.8, 155.8, 149.4, 112.2, 103.8, 102.4, 56.4, 39.9, 39.0, 36.7, 29.1, 17.9, 12.7.

HRMS (ESI) calculated for $C_{26}H_{42}BrO_2Si$: 493.2132 $[M+H]^+$, found: 493.2126.

The ^1H NMR signal at 1.77 ppm exhibits an AB coupling pattern giving an AB quartet with J_{AB} = 19.7 Hz and ν_{AB} = 31.9 Hz. Each of the peaks in the multiplet contains an underlying higher order multiplet that does not resolve.

(5-(adamantan-1-yl)-6-bromo-3-methoxyphenoxy)-triisopropylsilane (9b): White solid. 298 mg (34%). R_f = 0.61 (5:1 Hexane/DCM). M.p. 77 - 79 °C.

^{1}H NMR (600 MHz, $CDCl_3$) δ 6.58 (d, *J* = 2.9 Hz, 1H), 6.39 (d, *J* = 2.9 Hz, 1H), 3.75 (s, 3H), 2.29 – 2.25 (m, 6H), 2.12 – 2.08 (m, 3H), 1.81 – 1.71 (m, 6H), 1.34 (hept, *J* = 7.5 Hz, 3H), 1.14 (d, *J* = 7.5 Hz, 18H).

^{13}C NMR (151 MHz, $CDCl_3$) δ 158.6, 154.0, 149.6, 107.2 (2C), 102.8, 55.2, 39.8, 39.1, 36.8, 29.2, 18.1, 13.1.

HRMS (ESI) calculated for $C_{26}H_{42}BrO_2Si$: 493.2132 $[M+H]^+$, found: 493.2126.

The ^{1}H NMR signal at 1.76 ppm exhibits an AB coupling pattern giving an AB quartet with J_{AB} = 12.1 Hz and ν_{AB} = 27.5 Hz. Each of the peaks in the multiplet contains an underlying higher order multiplet that does not resolve.

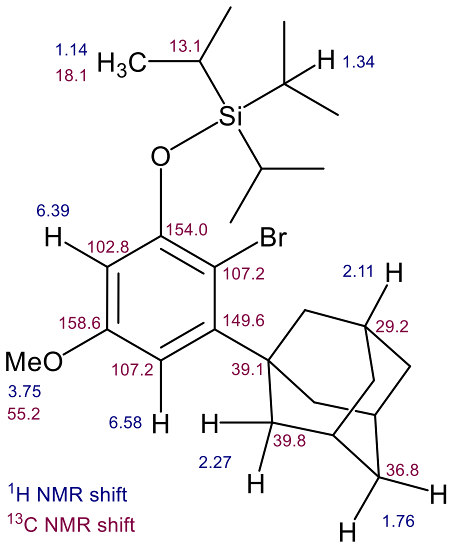

(5-(adamantan-1-yl)-2-bromo-3-methoxyphenoxy)-triisopropylsilane (9c): White solid. 120 mg (14%). R_f = 0.49 (5:1 Hexane/DCM). M.p. 105 - 106 °C.

^{1}H NMR (600 MHz, $CDCl_3$) δ 6.56 (d, *J* = 1.9 Hz, 1H), 6.51 (d, *J* = 1.9 Hz, 1H), 3.89 (s, 3H), 2.10 (s, 3H), 1.85 (d, *J* = 2.4 Hz, 6H), 1.81 – 1.72 (m, 6H), 1.32 (hept, *J* = 7.5 Hz, 3H), 1.14 (d, *J* = 7.5 Hz, 18H).

^{13}C NMR (151 MHz, $CDCl_3$) δ 156.8, 153.7, 151.7, 109.4, 101.4, 101.2, 56.3, 43.1, 36.7, 36.3, 28.9, 18.0, 13.0.

HRMS (ESI) calculated for $C_{26}H_{41}BrO_2SiNa$: 515.1951 $[M+Na]^+$, found: 515.1945.

The ^{1}H NMR signal at 1.77 ppm exhibits an AB coupling pattern giving an AB quartet with J_{AB} = 12.2 Hz and ν_{AB} = 30.0 Hz. Each of the peaks in the multiplet contains an underlying higher order multiplet that does not resolve.

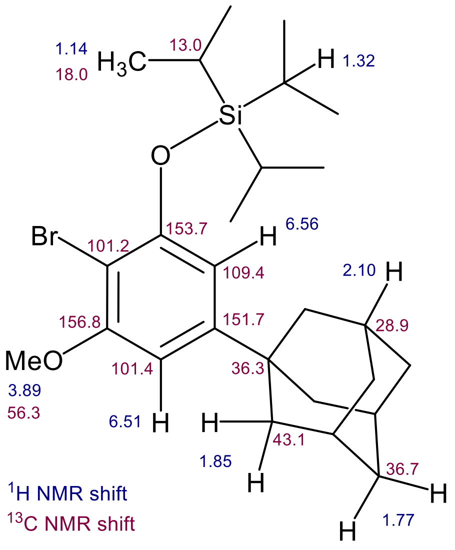

5-(adamantan-1-yl)-4-bromo-3-methoxyphenol (13a)

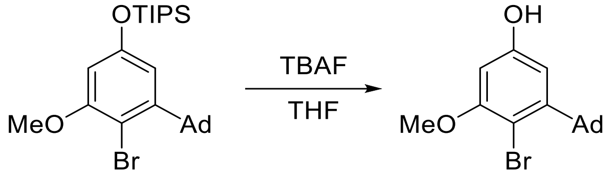

9a **13a**

Procedure with (5-(adamantan-1-yl)-4-bromo-3-methoxyphenoxy)triisopropylsilane as substrate:

Performed according to a published procedure.[5] Under Ar atmosphere, (5-(adamantan-1-yl)-4-bromo-3-methoxyphenoxy)triisopropylsilane (806 mg, 1.63 mmol) was dissolved in 1 mL of anhydrous THF. A 1M solution of TBAF (tetrabutylammonium fluoride) in THF (2.04 mL, 2.04 mmol, 1.25 equiv.) was slowly added. The reaction was stirred at r.t. for 20 minutes, followed by addition of H_2O and excess of EtOAc. The layers were separated and the organic phase was washed with H_2O and brine, and dried over $MgSO_4$. Volatiles were removed under reduced pressure. The title product was obtained after purification by flash column chromatography (1:1 → 1:3 Pentane/DCM) as a white solid. 530 mg (96%).

Procedure with a mixture of two isomers as substrates:

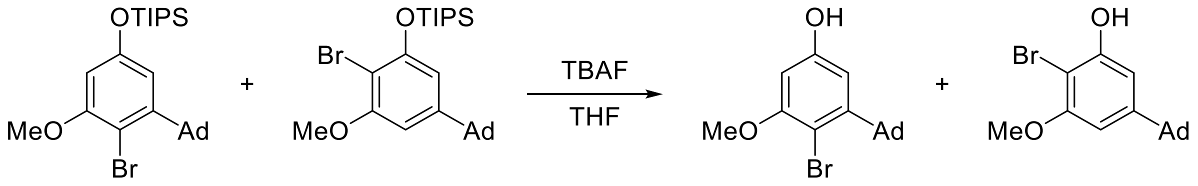

9a **9c** **13a** **13c**

Following the procedure as described above, using 2.49 g (5.041 mmol) of a mixture of isomers **9a** and **9c**, as mentioned in the previous step.
Isomer **13c** was first separated by flash column chromatography (Pentane/EtOAc 10:1 → 5:1 → 1:1) and isolated as a white solid. 436 mg (26%). A mixture of **13a** and traces of **13c** was collected and subjected to second flash column chromatography (5:1 → 1:1 Pentane/Et_2O) which allowed most of isomer **13a** to be isolated as a white solid. 1.093 g (64%).

5-(adamantan-1-yl)-4-bromo-3-methoxyphenol (13a): R_f = 0.23 (1:1 Hexane/Et_2O). M.p. 148 - 149 °C.

^{1}H NMR (600 MHz, $CDCl_3$) δ 6.50 (d, *J* = 2.8 Hz, 1H), 6.37 (d, *J* = 2.8 Hz, 1H), 4.81 (s, 1H), 3.84 (s, 3H), 2.27 – 2.24 (m, 6H), 2.12 – 2.08 (m, 3H), 1.80 – 1.71 (m, 6H).

^{13}C NMR (151 MHz, $CDCl_3$) δ 157.1, 155.2, 150.0, 107.7, 103.4, 98.0, 56.5, 39.8, 39.0, 36.7, 29.1.

HRMS (ESI) calculated for $C_{17}H_{21}BrO_2Na$: 359.0617 $[M+Na]^+$, found: 359.0616.

The ^{1}H NMR signal at 1.76 ppm exhibits an AB coupling pattern giving an AB quartet with J_{AB} = 12.2 Hz and ν_{AB} = 24.8 Hz. Each of the peaks in the multiplet contains an underlying higher order multiplet that does not resolve.

5-(adamantan-1-yl)-2-bromo-3-methoxyphenol (13c): R_f = 0.46 (10:1 Pentane/Et_2O).

^{1}H NMR (600 MHz, $CDCl_3$) δ 6.71 (d, *J* = 2.0 Hz, 1H), 6.50 (d, *J* = 1.9 Hz, 1H), 5.54 (s, 1H), 3.90 (s, 3H), 2.12 – 2.07 (m, 3H), 1.90 – 1.86 (m, 6H), 1.82 – 1.71 (m, 6H).

^{13}C NMR (151 MHz, $CDCl_3$) δ 155.9, 153.0, 152.9, 105.5, 100.8, 96.8, 56.3, 43.0, 36.7, 36.5, 28.8.

HRMS (ESI) calculated for $C_{17}H_{21}BrO_2$: 337.0798 $[M+H]^+$, found: 337.0797.

The ^{1}H NMR signal at 1.76 ppm exhibits an AB coupling pattern giving an AB quartet with J_{AB} = 12.3 Hz and ν_{AB} = 30.2 Hz. Each of the peaks in the multiplet contains an underlying higher order multiplet that does not resolve.

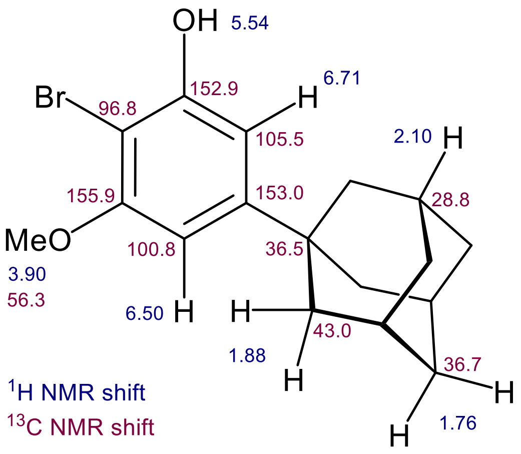

5-(adamantan-1-yl)-4-bromo-3-methoxyphenyl trifluoromethanesulfonate (1)

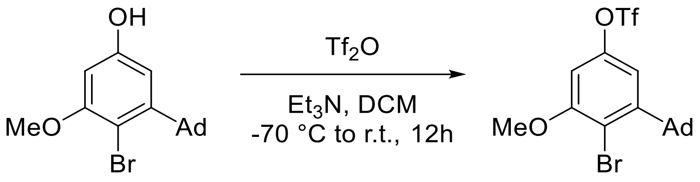

13a **1**

Under Ar atmosphere, 5-(adamantan-1-yl)-4-bromo-3-methoxyphenol (1.093 g, 3.24 mmol) was dissolved in 11 mL of anhydrous DCM and cooled to -70 °C. Et_3N (0.94 mL, 6.80 mmol, 2.1 equiv.) was added to the solution, followed by slow addition of triflic anhydride (0.60 mL, 3.56 mmol, 1.1 equiv.). The mixture was allowed to slowly warm to r.t. where it was stirred for 12 hours. Sat. aq. $NaHCO_3$ was added to the mixture, the layers were separated and the aqueous phase was further extracted with Et_2O. The combined organic layers were washed with brine and dried over $MgSO_4$. Volatiles were removed under reduced pressure, and the crude material was purified by flash column chromatography (10:1 Pentane/Et_2O) to yield the title compound as a white solid. 1.49 g (98%). R_f = 0.44 (5:1 Hexane/DCM). M.p. 100 - 101 °C.

^{1}H NMR (600 MHz, $CDCl_3$) δ 6.93 (d, *J* = 2.7 Hz, 1H), 6.71 (d, *J* = 2.7 Hz, 1H), 3.90 (s, 3H), 2.30 – 2.25 (m, 6H), 2.15 – 2.10 (m, 3H), 1.83 – 1.73 (m, 6H).

^{13}C NMR (151 MHz, $CDCl_3$) δ 157.3, 151.2, 149.0, 118.7 (q, *J* = 320.9 Hz), 113.1, 112.4, 103.1, 56.9, 39.6 (2C), 36.5, 28.9.

^{19}F NMR (376 MHz, $CDCl_3$) δ -72.76.

MS (70eV, EI): *m/z* (%): 470 (100, ^{81}Br) 468 (98, ^{79}Br) [M^+], 332 (14), 256 (18), 213 (15), 135 (46), 93 (16), 79 (20), 69 (17).

HRMS (ESI) calculated for $C_{18}H_{20}BrF_3O_4S$: 468.0212 $[M]^+$, found: 468.0206.

The ^{1}H NMR signal at 1.77 ppm exhibits an AB coupling pattern giving an AB quartet with J_{AB} = 12.3 Hz and ν_{AB} = 20.4 Hz. Each of the peaks in the multiplet contains an underlying higher order multiplet that does not resolve.

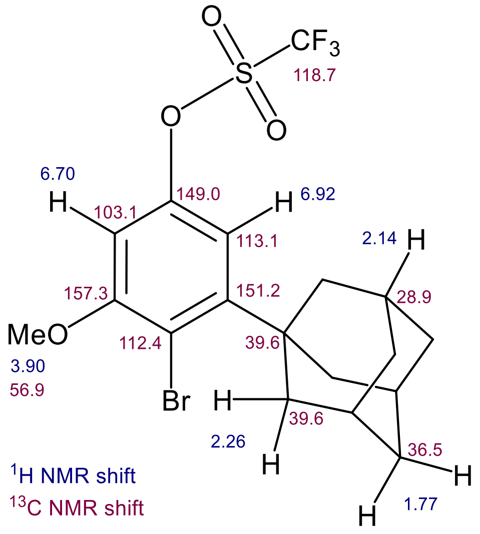

Single crystals suitable for X-Ray diffraction analysis were grown by slow evaporation of hexane from the solution of the title compound. All ellipsoids are drawn at 50% probability. The asymmetric unit consists of two independent molecules one of which is displayed on the right.

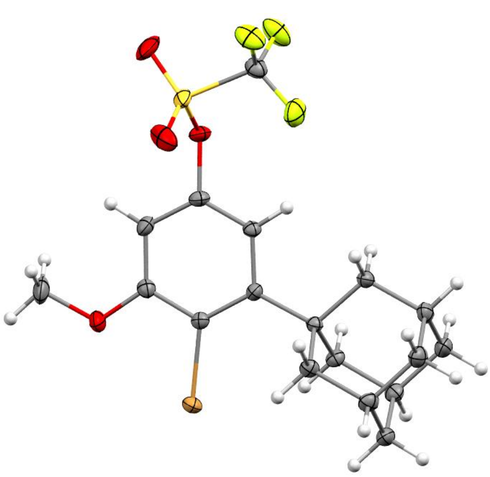

2.1.2. *Studies into the bromination of 5-adamantyl-3-methoxyphenol derivatives*

We also explored alternative strategies for selectively accessing various isomers of bromo-5-(adamantan-1-yl)-3-methoxyphenol. Using different OH-protecting groups on the phenol (Table S1, Entries 1-5) led to varying ratios of 2-, 4-, and 6-bromo isomers (C, A, B, respectively) with no distinctive preference for a specific one under any conditions. Somewhat notably the TIPS protecting group slightly favors bromination of the 4-position (A), while the TBDMS group tilts the ratio slightly in favor of the 6-position (B). Triphenylphosphine sulfide and the Nagasawa's chiral bis-thiourea organocatalyst have been reported in the literature to be capable of controlling the selectivity of electrophilic bromination reactions.[6] In this case both catalysts led to the increased bromination at the 2-position (C), as shown by Table S1, entries 6-8.

Table S1. Various strategies for attempting to control the selectivity of the electrophilic bromination of 5-adamantyl-3-methoxyphenol derivatives.

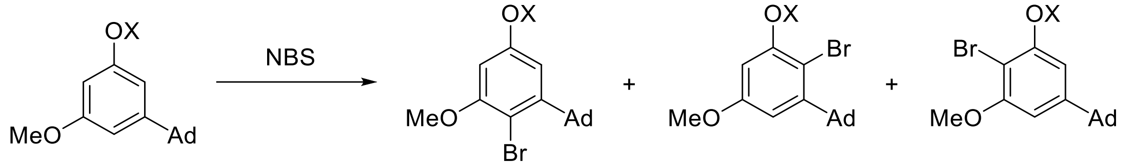

X = H, SiR_3, SO_2CF_3 **A** *(desired product)*   **B**   **C**

Entry	OX	A	B	C	Other products		Notes
1	OH	13%	38%	24%	25%	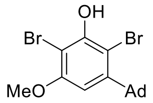	
2	OTf	-	-	-		-	
3	OTBDMS	36%	51%	14%			
4	OTBDPS	42%	42%	15%			
5	OTIPS	49%	36%	15%			
6	OH	-	-	57%	43%	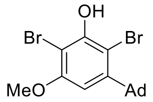	*Ph_3PS as a catalyst*[6]
7	OH	-	-	62%	38%	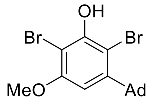	*Nagasawa's chiral thiourea catalyst**[6]
8	OTIPS	28%	18%	54%			*Ph_3PS as a catalyst*[6]

* *Nagasawa's chiral bis-thiourea organocatalyst:*

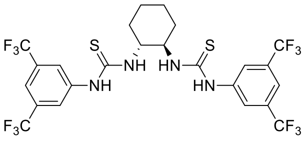

Procedure: To a vial, containing 5-adamantan-1-yl-3-methoxyphenol derivative (0.1 mmol), NBS (18 mg, 0.1 mmol) and a catalyst *(only entries 6-8)* (0.01 mmol, 10 mol%) was added 0.5 mL of anhydrous DCM and the reaction was stirred at r.t. for 13-16 hours. Thereafter, the reaction mixture was filtered through a plug of silica, and all of the volatiles removed under reduced pressure. The crude mixture was analyzed by quantitative ^1H NMR to determine the product distribution.

2.1.3. *Synthesis of 2-bromo adamantylarenes* (**11**)

1-(2-Bromophenyl)adamantane (11a)

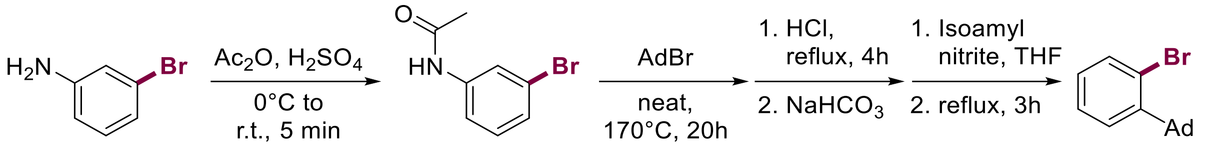

11a

***N*-(3-bromophenyl)acetamide:** 3-Bromoaniline (6.5 mL, 60 mmol) was weighed into a 50 mL round-bottom flask and cooled to 0°C using an ice bath. Acetic anhydride (11.3 mL, 120 mmol) was added carefully under stirring, followed by concentrated sulfuric acid (1.0 mL). The reaction mixture was allowed to warm and stirred for further 5 minutes once reaching ambient temperature. The obtained mixture was poured onto ice water (100 mL) and the aqueous phase was extracted using EtOAc (150 mL). The organic layer was then washed with a saturated aqueous solution of $NaHCO_3$ (100 mL) and brine (100 mL), followed by drying over $MgSO_4$. $MgSO_4$ was removed by filtration over silica and the solvent removed under reduced pressure. The product was obtained as an off-white solid and used without further purification. 12.7 g, (99%). M.p. 85 – 86°C. ^{1}H NMR (600 MHz, CD_2Cl_2) δ 8.18 (s, 1H), 7.84 (s, 1H), 7.40 (d, *J* = 8.1 Hz, 1H), 7.22 (d, *J* = 8.1 Hz, 1H), 7.17 (dd, *J* = 8.1, 8.1 Hz, 1H), 2.15 (s, 3H). ^{13}C NMR (151 MHz, CD_2Cl_2) δ 169.5, 140.0, 130.6, 127.3, 123.1, 122.7, 118.7, 24.6. MS (70eV, EI): *m/z* (%): 215 (25, ^{81}Br), 213 (25, ^{79}Br) $[M]^+$, 174 (7), 173 (98), 172 (8), 171 (100), 92 (38), 91 (14), 90 (5), 65 (25), 64 (13), 63 (20), 62 (6). These data are in agreement with those reported previously in the literature.[7]

1-(2-bromophenyl)adamantane (11a): 1-Bromoadamantane (1.5 g, 6.98 mmol) and *N*-(3-bromophenyl) acetamide (7.5 g, 35 mmol) were weighed into a pressure tube with a PTFE screw cap. The tube was placed in an oil bath (covered up to the cap) and heated at 170°C for 20 h under stirring. The reaction mixture was allowed to cool to ambient temperature and was transferred to a round bottom flask. An aqueous solution of HCl (40 mL, 6N) was added and the mixture refluxed for 4 h. After cooling to ambient temperature a saturated aqueous solution of $NaHCO_3$ (roughly 200 mL) was added until a pH of 7 was reached. The aqueous phase was extracted with EtOAc (3x 150 mL) and the combined organic layer dried over $MgSO_4$. The solvent was removed under reduced pressure and the crude dissolved in THF (40 mL). Isoamylnitrite (9.4 mL, 70 mmol) was added drop-wise. After completion of the addition, the reaction mixture was heated to reflux. After 3h the mixture was cooled to ambient temperature and celite was added. The solvent was removed under reduced pressure and the crude (adsorbed onto celite) was purified by column chromatography (hexane). The formed bromobenzene byproduct was removed under reduced pressure (together with the solvent after column chromatography). The title product was obtained as a white crystalline solid. 789.9 mg (39% over 3 steps). R_f = 0.74 (hexane). M.p. 98 – 99°C. ^{1}H NMR (600 MHz, CD_2Cl_2) δ 7.59 – 7.54 (m, 1H), 7.38 (dd, *J* = 8.2, 1.4 Hz, 1H), 7.31 – 7.25 (m, 1H), 7.06 – 7.00 (m, 1H), 2.28 – 2.24 (m, 6H), 2.12 (br, 3H), 1.82 – 1.75 (m, 6H). ^{13}C NMR (151 MHz, CD_2Cl_2) δ 147.9, 136.4, 128.8, 127.8, 127.7, 122.5, 40.3, 38.8, 37.1, 29.6. MS (70eV, EI): *m/z* (%): 293 (17), 292 (98, ^{81}Br), 291 (18), 290 (100, ^{79}Br) $[M]^+$, 250 (3), 249 (10), 248 (3), 247 (10), 236 (35), 235 (35), 234 (42), 233 (31), 171 (15), 169 (20), 168 (15), 167 (12), 165 (9), 156 (6), 155 (35), 154 (98), 153 (29), 152 (20), 142 (7), 141 (18), 135 (31), 129 (15), 128 (24), 127 (9), 121 (6), 116 (13), 115 (28), 107 (6), 103 (5), 102 (9), 95 (7), 94 (41), 93 (21), 91 (23), 90 (6), 89 (8), 79 (27), 78 (6), 77 (22), 76 (7), 67 (8), 65 (7), 55 (5), 53 (5), 51 (6). HRMS (APCI) calculated for $C_{16}H_{19}{}^{81}Br$: 292.0644 $[M]^+$, found: 292.0635. These data are in agreement with those reported previously in the literature.[8]

1-(2-Bromo-5-fluorophenyl)adamantane (11b)

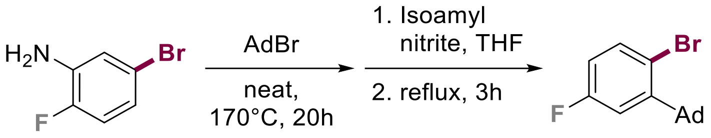

11b

1-Bromoadamantane (1 g, 4.65 mmol) and 5-bromo-2-fluoroaniline (4.4 g, 23.2 mmol) were weighed into a pressure tube. The tube was sealed with a PTFE screw cap and placed in an oil bath (covered up to the cap) and heated at 170°C for 20 h under stirring. The reaction mixture was allowed to cool to ambient temperature and was transferred to a round bottom flask. The crude was dissolved in THF (7.7 mL, 0.6M) and isoamylnitrite (5.4 mL, 46.5 mmol) was added drop-wise at room temperature. After completion of the addition, the reaction mixture was heated to reflux. After 3h the mixture was cooled to ambient temperature and celite was added. The solvent was removed under reduced pressure and the crude (adsorbed onto celite) was purified by column chromatography (hexane). The formed 1-bromo-4-fluorobenzene byproduct was removed under reduced pressure (together with the solvent after column chromatography). The title product was obtained as a white crystalline solid. 721.6 mg (50% over 2 steps) R_f = 0.70 (hexane). M.p. 99 – 100°C. ^{1}H NMR (600 MHz, CD_2Cl_2) δ 7.52 (dd, *J* = 8.7, 6.0 Hz, 1H), 7.11 (dd, *J* = 11.9, 3.1 Hz, 1H), 6.80 – 6.75 (m, 1H), 2.24 – 2.20 (m, 6H), 2.12 (br, 3H), 1.82 – 1.73 (m, 6H). ^{13}C NMR (151 MHz, CD_2Cl_2) δ 162.5 (d, *J* = 245.0 Hz), 150.4 (d, *J* = 6.0 Hz), 137.3 (d, *J* = 7.4 Hz), 116.4 (d, *J* = 3.5 Hz), 116.2 (d, *J* = 24.1 Hz), 114.4 (d, *J* = 22.2 Hz), 39.9, 38.9, 36.9, 29.5. ^{19}F NMR (564 MHz, CD_2Cl_2) δ -115.39 – -115.46 (m). MS (70eV, EI): *m/z* (%): 311 (17), 310 (100, ^{81}Br), 309 (19), 308 (100, ^{79}Br) $[M]^+$, 267 (7), 265 (7), 254 (29), 253 (26), 252 (36), 251 (23), 250 (6), 189 (16), 187 (21), 186 (15), 185 (11), 183 (8), 174 (8), 173 (35), 172 (98), 171 (20), 170 (17), 165 (7), 161 (6), 160 (10), 159 (24), 153 (11), 152 (12), 147 (16), 146 (28), 136 (9), 135 (80), 134 (17), 133 (36), 127 (6), 121 (9), 120 (13), 109 (16), 108 (12), 107 (17), 95 (14), 94 (47), 93 (40), 92 (7), 91 (18), 83 (5), 82 (6), 81 (10), 80 (7), 79 (44), 78 (6), 77 (21), 75 (5), 67 (15), 66 (5), 65 (8), 55 (9), 53 (9), 51 (6). HRMS (APCI) calculated for $C_{16}H_{18}{}^{79}BrF$: 308.0570 $[M]^+$, found: 308.0563.

1-(2-Bromo-5-chlorophenyl)adamantane (11c)

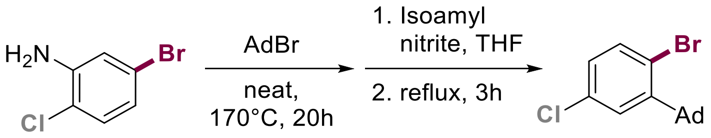

11c

The title compound was synthesized in analogy to **11b** from 1-bromoadamantane (0.86 g, 4.00 mmol) and 5-bromo-2-chloroaniline (4.2 g, 20.0 mmol). The crude was purified by column chromatography (hexane) to yield the product as a white crystalline solid. 969.0 mg (74% over 2 steps). R_f = 0.80 (hexane). M.p. 126 – 127°C. ^{1}H NMR (600 MHz, CD_2Cl_2) δ 7.49 (d, *J* = 8.4 Hz, 1H), 7.34 (d, *J* = 2.6 Hz, 1H), 7.03 (dd, *J* = 8.5, 2.5 Hz, 1H), 2.25 – 2.19 (m, 6H), 2.12 (br, 3H), 1.80 – 1.75 (m, 6H). ^{13}C NMR (151 MHz, CD_2Cl_2) δ 149.7, 137.4, 133.8, 129.1, 127.5, 120.3, 40.0, 39.0, 36.9, 29.5. MS (70eV, EI): *m/z* (%): 328 (26, ^{37}Cl^{81}Br), 327 (18), 326 (100, ^{35}Cl^{81}Br/^{37}Cl^{79}Br), 325 (14), 324 (77, ^{35}Cl^{79}Br) $[M]^+$, 283 (7), 281 (6), 272 (7), 271 (10), 270 (31), 269 (27), 268 (31), 267 (18), 266 (5), 207 (6), 205 (17), 203 (16), 202 (8), 191 (8), 190 (28), 189 (22), 188 (69), 175 (7), 167 (13), 166 (9), 165 (19), 163 (6), 162 (6), 155 (7), 154 (16), 153 (30), 152 (38), 151 (11), 150 (8), 149 (8), 142 (7), 141 (15), 139 (10), 138 (5), 136 (17), 135 (94), 129 (8), 128 (21), 127 (17), 126 (8), 125 (10), 121 (6), 116 (7), 115 (31), 108 (7), 107 (13), 102 (7), 101 (7), 95 (13), 94 (53), 93 (41), 91 (21), 89 (12), 83 (6), 82 (11), 81 (11), 80 (7), 79 (49), 78 (7), 77 (29), 76 (11), 75 (10), 67 (17), 66 (5), 65 (10), 63 (8), 55 (12), 53 (11), 51 (8). HRMS (APCI) calculated for $C_{16}H_{18}{}^{79}Br^{35}Cl$: 324.0275 $[M]^+$, found: 324.0275.

2.2. Cross-coupling reactions

2.2.1. Optimization of reaction conditions for the cross-coupling of **1** *with organozinc reagents*

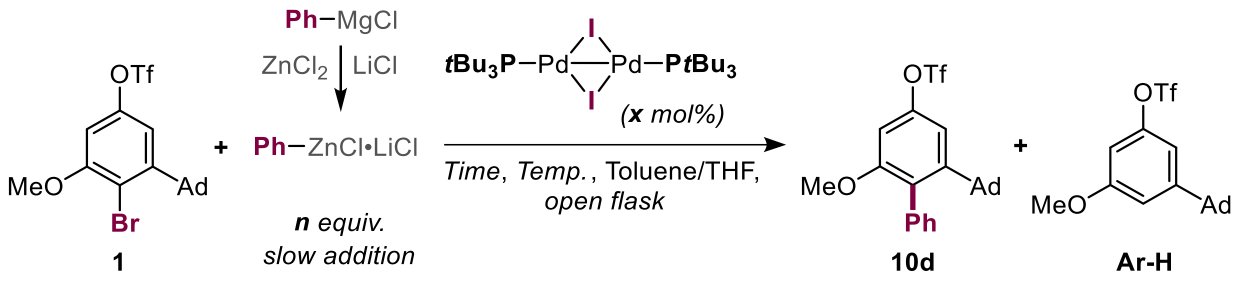

	Catalyst [mol%]	PhZnCl [equiv.]	PhZnCl [addn. rate]	Temp. [°C]	10d	1	Ar-H
**1**	2.5	5	5 min	r.t.	**46%**	52%	1%
**2**	5 + 5 (after 3 min.)	5	5 min	r.t.	**48%**	49%	3%
**3**	10	5	5 min	50 °C	**67%**	17%	16%
**4**	10	5	7.5 min	50 °C	**70%**	20%	10%
**5**	10	7	9 min	50 °C	**82%**	6%	12%

All reactions were performed as described in general cross-coupling procedure A (see below), with appropriate modifications as specified above.

2.2.2. *General cross-coupling procedures*

General cross-coupling procedure A

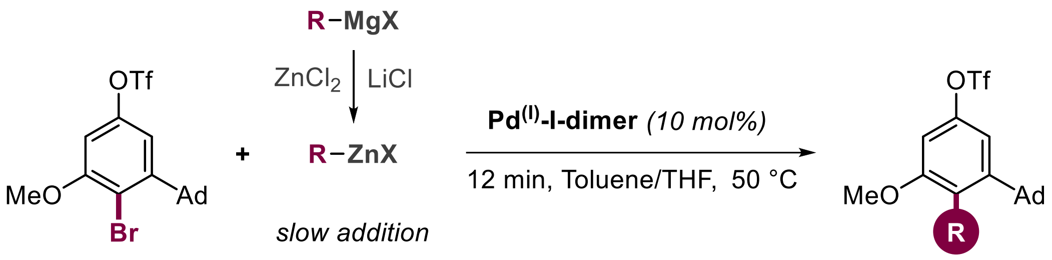

1 **10**

All reactions following this procedure were performed using $[(PtBu_3)Pd^I(I)]_2$ (8.7 mg, 0.01 mmol, 10 mol%) catalyzed cross-couplings between the aryl bromide (47 mg, 0.1 mmol) and organozinc chloride. A stock solution of $RZnCl{\cdot}LiCl{\cdot}MgCl_2$ was prepared by mixing a solution of RMgCl (1 equiv.) with 1M solution of $ZnCl_2$ (1.1 equiv.) in THF and 0.5M solution of LiCl (1 equiv.) in THF. After 15 minutes of stirring, a corresponding aliquot was taken from the stock solution and used in the coupling reaction.

The aryl bromide and the Pd catalyst were weighed to a septum-capped vial equipped with a stir-bar, the vial was evacuated and back-filled with argon. Toluene (0.6 mL) was added and the solution warmed to 50 °C. RZnCl was added to the reaction over 12 minutes. After an additional 5 minutes of stirring the mixture was allowed to cool to r.t., and hexane was added to the mixture. Solid residues were removed by filtration through a short plug of silica (~2 cm Pasteur pipette column). Volatiles were evaporated and the crude mixture was analyzed by ^{1}H NMR and GC/MS to determine the conversion. The crude material was then subjected to purification by column chromatography.

General cross-coupling procedure B

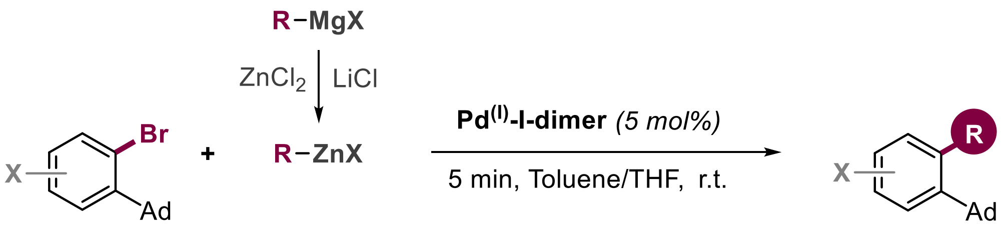

11 **12**

All reactions following this procedure were performed using $[(PtBu_3)Pd^I(I)]_2$ (8.7 mg, 0.01 mmol, 5 mol%) catalyzed cross-couplings between the aryl bromide (0.2 mmol) and organozinc chloride. A stock solution of $RZnCl{\cdot}LiCl{\cdot}MgCl_2$ was prepared by mixing a solution of RMgCl (0.3 mmol, 1.5 equiv.) with 1M solution of $ZnCl_2$ (0.32 mmol, 1.6 equiv.) in THF and 0.5M solution of LiCl (0.3 mmol, 1.5 equiv.) in THF. After 15 minutes of stirring, a corresponding aliquot was taken from the stock solution and used in the coupling reaction.

The aryl bromide and the Pd catalyst were weighed to a septum-capped vial equipped with a stir-bar, the vial was evacuated and back-filled with argon. After the addition of toluene (0.8 mL), the organozinc solution was added and the mixture was stirred for 5 minutes at ambient temperature. Hexane was added to the mixture. Solid residues were removed by filtration through a short plug of silica (~2 cm Pasteur pipette column). Volatiles were evaporated and the crude material was then subjected to purification by column chromatography.

2.2.3. *Characterization data for cross-coupling products*

3-(adamantan-1-yl)-5-methoxy-4-methylphenyl trifluoromethanesulfonate (10a)

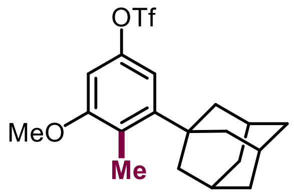

Prepared by a coupling reaction between the aryl bromide (23 mg, 0.05 mmol) and MeZnCl (0.15 mmol, 3 equiv.), catalyzed by [(P*t*Bu$_3$)Pd^I(I)]$_2$ (1.1 mg, 0.0013 mmol, 2.5 mol%), in toluene (0.3 mL), performed at room temperature under open flask conditions. MeZnCl was prepared from a 3M solution of MeMgCl in Et$_2$O, 1M ZnCl$_2$ in THF and 0.5M LiCl in THF. From the obtained ~0.29M stock solution of MeZnCl, 0.52 mL (0.15 mmol, 3 equiv.) was taken to be added to the reaction slowly, over 4 minutes. The reaction was worked up as described in the general cross-coupling procedure.

Analysis of the crude material showed 98% conversion to the desired product, along with 2% of debrominated starting material (5-(adamantan-1-yl)-3-methoxyphenyl triflate).

The title product was obtained after purification by column chromatography (10:1 Hexane/DCM) as a white solid. 18 mg (90%). R$_f$ = 0.23 (10:1 Hexane/DCM). M.p. 83 - 84 °C.

^{1}H NMR (400 MHz, CDCl$_3$) δ 6.85 (d, *J* = 2.4 Hz, 1H), 6.63 (d, *J* = 2.4 Hz, 1H), 3.83 (s, 3H), 2.42 (s, 3H), 2.14 – 2.10 (m, 3H), 2.08 – 2.03 (m, 6H), 1.81 – 1.75 (m, 6H).

^{13}C NMR (101 MHz, CDCl$_3$) δ 159.3, 150.7, 148.0, 125.9, 118.8 (q, *J* = 320.9 Hz), 111.0, 101.2, 55.8, 41.2, 38.2, 36.7, 29.1, 14.9.

^{19}F NMR (376 MHz, CDCl$_3$) δ -72.95.

MS (70eV, EI): *m/z* (%): 404 (100) [M$^+$], 271 (10), 243 (22), 187 (10), 149 (36), 135 (29), 91 (12), 79 (13), 69 (10).

HRMS (ESI) calculated for $C_{19}H_{23}F_3O_4SNa$: 427.1161 [M+Na]$^+$, found: 427.1156.

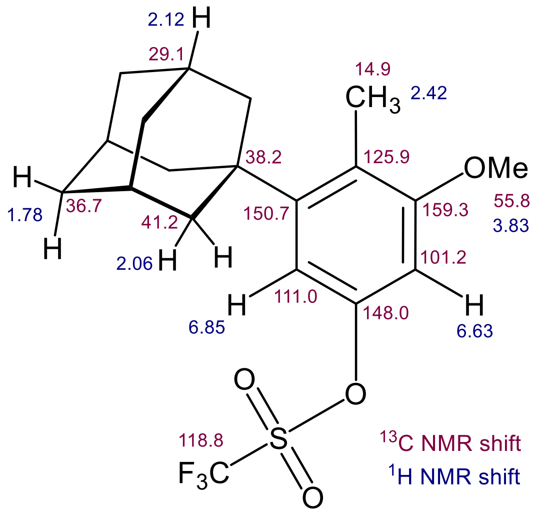

3-(adamantan-1-yl)-5-methoxy-4-((trimethylsilyl)methyl)phenyl trifluoromethanesulfonate (10b)

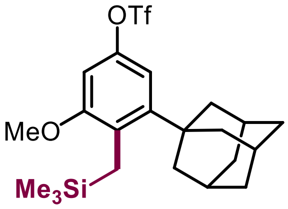

Prepared by a coupling reaction between the aryl bromide (23 mg, 0.05 mmol) and (TMS-CH_2)ZnCl (0.25 mmol, 5 equiv.), catalyzed by $[(PtBu_3)Pd^I(I)]_2$ (1.1 mg, 0.0013 mmol, 2.5 mol%), in toluene (0.3 mL), performed at room temperature under open flask conditions. (TMS-CH_2)ZnCl was prepared from a 1M solution of (TMS-CH_2)MgCl in Et_2O, 1M $ZnCl_2$ in THF and 0.5M LiCl in THF. From the obtained ~0.24M stock solution of (TMS-CH_2)ZnCl, 1 mL (0.25 mmol, 5 equiv.) was taken to be added to the reaction slowly, over 4 minutes. The reaction was worked up as described in the general cross-coupling procedure.

Analysis of the crude material showed 98% conversion to the desired product, along with <1% of remaining starting material and 2% of debrominated starting material (5-(adamantan-1-yl)-3-methoxyphenyl triflate).

The title product was obtained after purification by column chromatography (10:1 Hexane/DCM) as a colorless oil. 17 mg (71%). R_f = 0.34 (10:1 Hexane/DCM.

^{1}H NMR (400 MHz, $CDCl_3$) δ 6.83 (d, J = 2.5 Hz, 1H), 6.59 (d, J = 2.5 Hz, 1H), 3.78 (s, 3H), 2.45 (s, 2H), 2.12 (m, 3H), 2.09 – 2.01 (m, 6H), 1.83 – 1.71 (m, 6H), -0.02 (s, 9H).

^{13}C NMR (101 MHz, $CDCl_3$) δ 157.9, 148.7, 146.7, 129.7, 118.8 (q, J = 320.9 Hz), 111.1, 100.5, 55.1, 41.4, 37.9, 36.7, 29.0, 19.3, -0.1.

^{19}F NMR (376 MHz, $CDCl_3$) δ -72.93.

MS (70eV, EI): *m/z* (%): 476 (10) [M^+], 461 (46), 343 (59), 328 (16), 223 (33), 207 (28), 135 (13), 91 (22), 79 (20), 73 (100).

HRMS (ESI) calculated for $C_{22}H_{31}F_3O_4SSiNa$: 499.1557 $[M+Na]^+$, found: 499.1545.

The ^{1}H NMR signal at 1.77 ppm exhibits an AB coupling pattern giving an AB quartet with J_{AB} = 12.9 Hz and ν_{AB} = 14.9 Hz. Each of the peaks in the multiplet contains an underlying higher order multiplet that does not resolve.

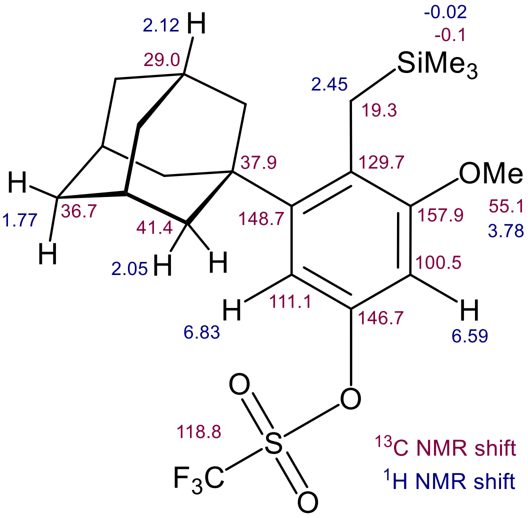

3-(adamantan-1-yl)-4-butyl-5-methoxyphenyl trifluoromethanesulfonate (10c)

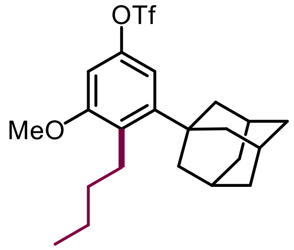

Prepared according to the general cross-coupling procedure A. (*n*-butyl)ZnCl was prepared from a 2M solution of (*n*-butyl)MgCl in THF. From the obtained ~0.28M stock solution of (*n*-butyl)ZnCl, 2.5 mL (0.7 mmol, 7 equiv.) was taken to be used in the reaction.

Analysis of the crude material showed 43% conversion to the desired product, along with 4% of remaining starting material and 52% of debrominated SM (5-(adamantan-1-yl)-3-methoxyphenyl triflate).

The title product was obtained after purification by column chromatography (20:1 → 10:1 Hexane/DCM) as a white solid. 21 mg (32%). R_f = 0.61 (5:1 Hexane/DCM). M.p. 73 - 74 °C.

^{1}H NMR (600 MHz, $CDCl_3$) δ 6.84 (d, *J* = 2.3 Hz, 1H), 6.63 (d, *J* = 2.3 Hz, 1H), 3.81 (s, 3H), 2.91 – 2.85 (m, 2H), 2.15 – 2.09 (m, 3H), 2.06 – 1.98 (m, 6H), 1.82 – 1.74 (m, 6H), 1.52 – 1.41 (m, 4H), 0.98 (t, *J* = 7.0 Hz, 3H).

^{13}C NMR (151 MHz, $CDCl_3$) δ 159.5, 150.4, 148.0, 131.4, 118.8 (q, *J* = 320.7 Hz), 111.1, 101.7, 55.8, 41.9, 38.5, 36.7, 31.8, 29.1, 28.0, 23.5, 13.9.

^{19}F NMR (564 MHz, $CDCl_3$) δ -72.96.

MS (70eV, EI): *m/z* (%): 446 (100) [M$^+$], 403 (88), 283 (48), 270 (45), 253 (47), 135 (59), 119 (15), 91 (20), 79 (27).

HRMS (ESI) calculated for $C_{22}H_{29}F_3O_4SNa$: 469.1631 [M+Na]$^+$, found: 469.1623.

The ^{1}H NMR signal at 1.77 ppm exhibits an AB coupling pattern giving an AB quartet where the ν_{AB} is too small to allow the measurement of any coupling constants. Each of the peaks in the multiplet contains an underlying higher order multiplet that does not resolve.

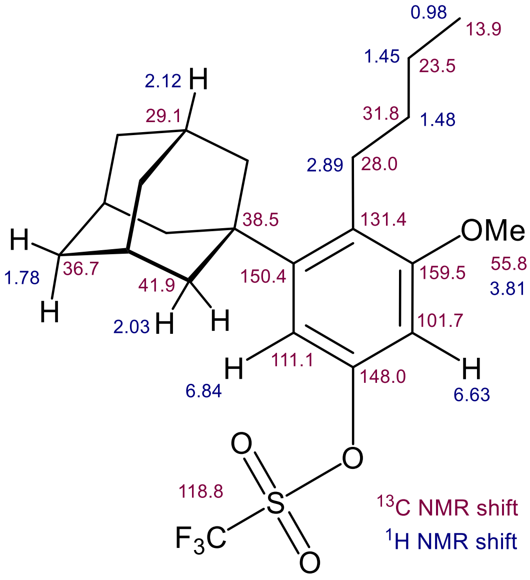

2-(adamantan-1-yl)-6-methoxy-[1,1'-biphenyl]-4-yl trifluoromethanesulfonate (10d)

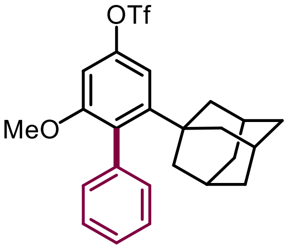

Prepared according to the general cross-coupling procedure A. PhZnCl was prepared from a 1M solution of PhMgCl in 2-MeTHF. From the obtained ~0.24M stock solution of PhZnCl, 2.87 mL (0.7 mmol, 7 equiv.) was taken to be used in the reaction.

Analysis of the crude material showed 82% conversion to the desired product, along with 6% of remaining starting material and 12% of debrominated SM (5-(adamantan-1-yl)-3-methoxyphenyl triflate). A large amount of biphenyl from the homocoupling of PhZnCl is also observed.

The title product was obtained after purification by column chromatography (20:1 → 10:1 → 5:1 Hexane/DCM) as a white solid. 30 mg (65%). R_f = 0.19 (10:1 Hexane/DCM). M.p. 126 - 127 °C.

^{1}H NMR (400 MHz, $CDCl_3$) δ 7.40 – 7.31 (m, 3H), 7.19 – 7.14 (m, 2H), 7.07 (d, J = 2.4 Hz, 1H), 6.70 (d, J = 2.4 Hz, 1H), 3.62 (s, 3H), 1.92 – 1.85 (m, 3H), 1.85 – 1.77 (m, 6H), 1.64 – 1.44 (m, 6H).

^{13}C NMR (101 MHz, $CDCl_3$) δ 159.0, 151.6, 149.3, 137.9, 131.5, 131.2, 127.2, 127.0, 118.8 (q, J = 320.8 Hz), 111.6, 101.9, 56.3, 42.6, 39.5, 36.3, 28.9.

^{19}F NMR (376 MHz, $CDCl_3$) δ -72.90.

MS (70eV, EI): *m/z* (%): 466 (100) [M^+], 333 (14), 305 (21), 273 (23), 211 (28), 207 (17), 135 (30), 79 (17).

HRMS (ESI) calculated for $C_{24}H_{25}F_3O_4SNa$: 489.1318 [$M+Na$]$^+$, found: 489.1324.

The ^{1}H NMR signal at 1.53 ppm exhibits an AB coupling pattern giving an AB quartet with J_{AB} = 12.1 Hz and ν_{AB} = 47.4 Hz. Each of the peaks in the multiplet contains an underlying higher order multiplet that does not resolve.

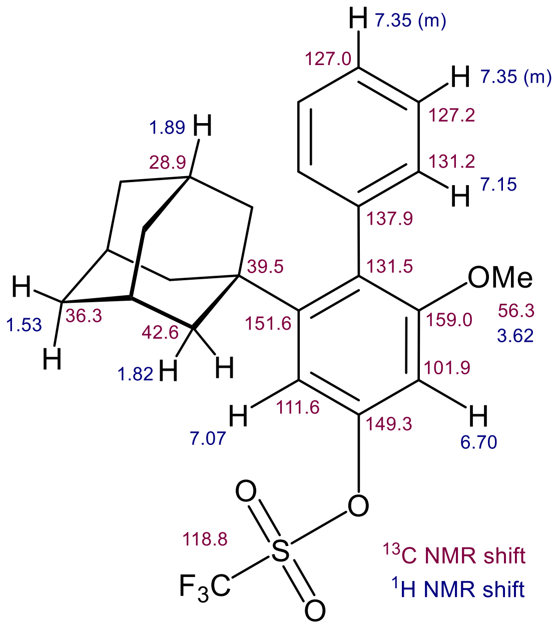

2-(adamantan-1-yl)-4'-chloro-6-methoxy-[1,1'-biphenyl]-4-yl trifluoromethanesulfonate (10e)

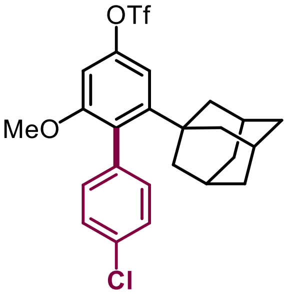

Prepared according to the general cross-coupling procedure A. (*p*-chlorophenyl)ZnCl was prepared from a 1M solution of (*p*-chlorophenyl)MgCl in THF, 1M $ZnCl_2$ in THF and 0.5M LiCl in THF. From the obtained ~0.24M stock solution of (*p*-chlorophenyl)ZnCl, 2.87 mL (0.70 mmol, 7 equiv.) was taken to be used in the reaction.

The title product was obtained after purification by column chromatography (100:0 → 50:1 Pentane/EtOAc) as a white solid. 31 mg (62%). R_f = 0.51 (75:1 Pentane/EtOAc). M.p. 128 - 129 °C.

^{1}H NMR (600 MHz, $CDCl_3$) δ 7.35 (d, *J* = 8.2 Hz, 2H), 7.09 (d, *J* = 8.2 Hz, 2H), 7.06 (d, *J* = 2.4 Hz, 1H), 6.69 (d, *J* = 2.4 Hz, 1H), 3.61 (s, 3H), 1.93 – 1.89 (m, 3H), 1.82 – 1.78 (m, 6H), 1.64 – 1.46 (m, 6H).

^{13}C NMR (151 MHz, $CDCl_3$) δ 158.9, 151.6, 149.5, 136.4, 132.9, 132.6, 130.1, 127.6, 118.8 (q, *J* = 321.0 Hz), 111.7, 101.9, 56.2, 42.7, 39.4, 36.3, 28.8.

^{19}F NMR (564 MHz, $CDCl_3$) δ -72.85.

MS (70eV, EI): *m/z* (%): 502 (18, ^{37}Cl) 500 (45, ^{35}Cl) [M^+], 369 (10, ^{37}Cl) 367 (6, ^{35}Cl), 309 (13, ^{37}Cl) 307 (5, ^{35}Cl), 247 (25, ^{37}Cl) 245 (10, ^{35}Cl), 135 (26), 79 (41), 69 (100).

HRMS (ESI) calculated for $C_{24}H_{25}F_3O_4ClS$: 501.1106 $[M+H]^+$, found: 501.1109.

The ^{1}H NMR signal at 1.55 ppm exhibits an AB coupling pattern giving an AB quartet with J_{AB} = 12.1 Hz and ν_{AB} = 72.0 Hz. Each of the peaks in the multiplet contains an underlying higher order multiplet that does not resolve.

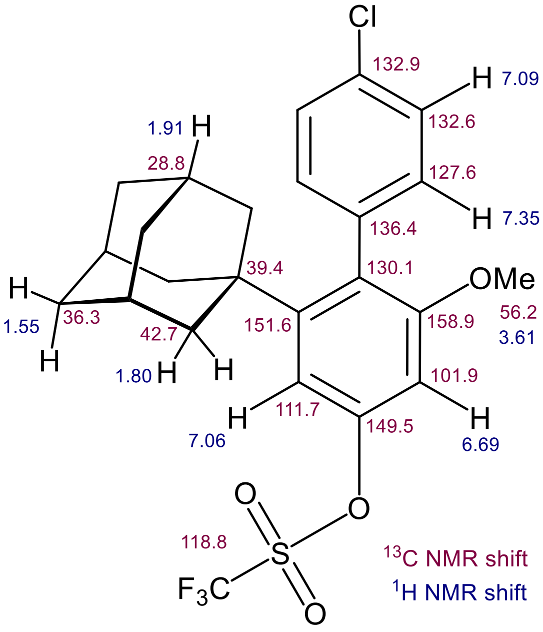

3-(adamantan-1-yl)-5-methoxy-4-(thiophen-2-yl)phenyl trifluoromethanesulfonate (10f)

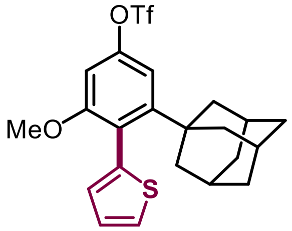

Prepared according to the general cross-coupling procedure A. (2-thienyl)ZnCl was prepared from a 1M solution of (2-thienyl)MgCl in THF. From the obtained ~0.24M stock solution of (2-thienyl)ZnCl, 2.87 mL (0.7 mmol, 7 equiv.) was taken to be used in the reaction.

Analysis of the crude material showed 83% conversion to the desired product, along with 14% of remaining starting material and 2% of debrominated SM (5-(adamantan-1-yl)-3-methoxyphenyl triflate). A large amount of biaryl from the homocoupling of (2-thienyl)ZnCl is also observed.

The title product was obtained after purification by column chromatography (20:1 → 10:1 → 5:1 Hexane/DCM) as a white solid. 29 mg (62%). R_f = 0.20 (10:1 Hexane/DCM). M.p. 137 - 138 °C.

^{1}H NMR (400 MHz, $CDCl_3$) δ 7.42 (d, J = 5.0 Hz, 1H), 7.08 – 7.05 (m, 2H), 6.88 (d, J = 3.1 Hz, 1H), 6.70 (d, J = 2.2 Hz, 1H), 3.68 (s, 3H), 1.97 – 1.92 (m, 3H), 1.92 – 1.87 (m, 6H), 1.68 – 1.53 (m, 6H).

^{13}C NMR (101 MHz, $CDCl_3$) δ 160.3, 154.1, 150.2, 138.0, 129.2, 126.13, 126.07, 123.3, 118.8 (q, J = 320.8 Hz), 111.7, 102.0, 56.5, 42.4, 39.7, 36.4, 29.0.

^{19}F NMR (376 MHz, $CDCl_3$) δ -72.85.

MS (70eV, EI): *m/z* (%): 472 (100) [M$^+$], 339 (100), 311 (17), 217 (39), 207 (48), 135 (39), 79 (29), 69 (27).

HRMS (ESI) calculated for $C_{22}H_{24}F_3O_4S_2$: 473.1063 [M+H]$^+$, found: 473.1071.

The ^{1}H NMR signal at 1.60 ppm exhibits an AB coupling pattern giving an AB quartet with J_{AB} = 12.0 Hz and ν_{AB} = 33.1 Hz. Each of the peaks in the multiplet contains an underlying higher order multiplet that does not resolve.

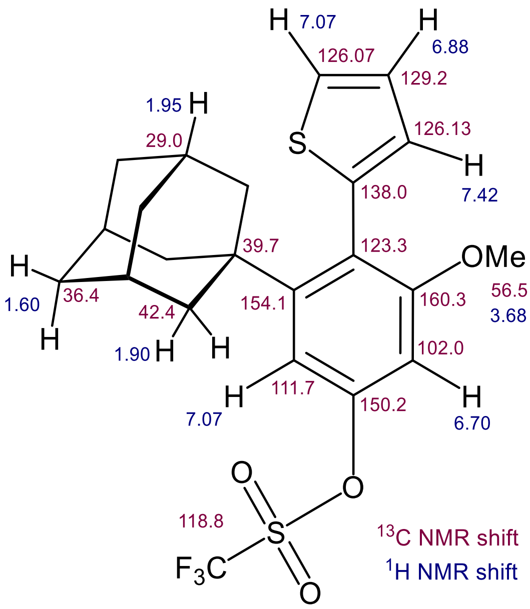

Single crystals suitable for X-Ray diffraction analysis were grown by slow evaporation of hexane from the solution of the title compound. All ellipsoids are drawn at 50% probability. The asymmetric unit consists of two independent molecules one of which is displayed on the right. The structure exhibits disorder at the thiophene and triflate moieties (displayed in light blue).

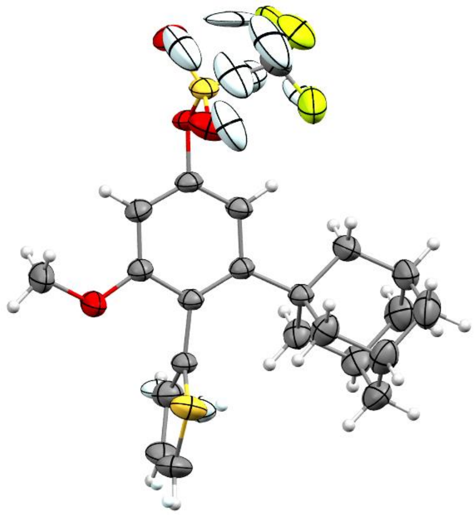

2-(adamantan-1-yl)-4',6-dimethoxy-[1,1'-biphenyl]-4-yl trifluoromethanesulfonate (10g)

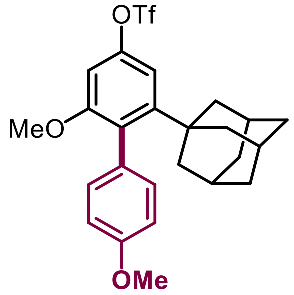

Prepared according to the general cross-coupling procedure A. (*p*-methoxyphenyl)ZnCl was prepared from a 1M solution of (*p*-methoxyphenyl)MgCl in THF, 1M $ZnCl_2$ in THF and 0.5M LiCl in THF. From the obtained ~0.24M stock solution of (*p*-methoxyphenyl)ZnCl, 2.87 mL (0.70 mmol, 7 equiv.)

The title product was obtained after purification by column chromatography (50:1 Pentane/EtOAc) as a white solid. 14 mg (28%). R_f = 0.50 (50:1 Pentane/EtOAc). M.p. 124 - 125 °C.

^{1}H NMR (600 MHz, $CDCl_3$) δ 7.05 (d, *J* = 8.4 Hz, 2H) 7.04 (m, 1H), 6.91 (d, *J* = 8.4 Hz, 2H), 6.68 (d, *J* = 1.9 Hz, 1H), 3.86 (s, 3H), 3.62 (s, 3H), 1.92 – 1.87 (m, 3H), 1.83 – 1.79 (m, 6H), 1.62 – 1.45 (m, 6H).

^{13}C NMR (151 MHz, $CDCl_3$) δ 159.3, 158.5, 152.0, 149.2, 132.1, 131.2, 129.8, 118.8 (q, *J* = 321.4 Hz)., 112.7, 111.6, 101.8, 56.3, 55.2, 42.6, 39.4, 36.4, 28.9.

^{19}F NMR (564 MHz, $CDCl_3$) δ -72.88.

MS (70eV, EI): *m/z* (%): 496 (60) [M$^+$], 363 (6), 335 (9), 241 (15), 135 (25), 69 (100).

HRMS (ESI) calculated for $C_{25}H_{27}F_3O_5SNa$: 519.1421 [M+Na]$^+$, found: 519.1424.

The ^{1}H NMR signal at 1.54 ppm exhibits an AB coupling pattern giving an AB quartet with J_{AB} = 12.0 Hz and ν_{AB} = 64.2 Hz. Each of the peaks in the multiplet contains an underlying higher order multiplet that does not resolve.

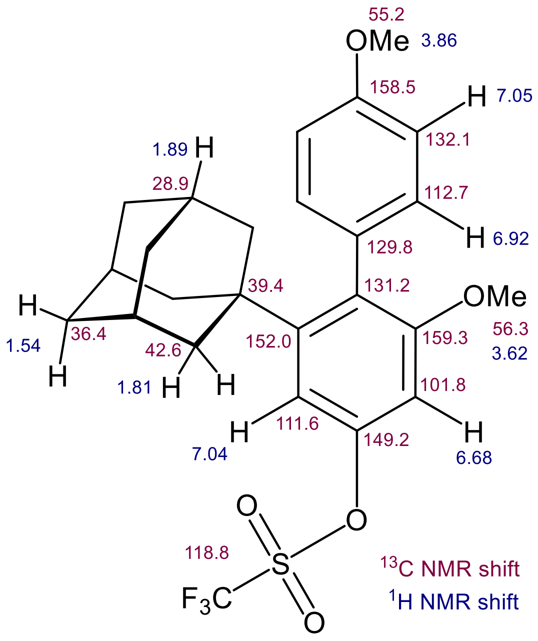

Single crystals suitable for X-Ray diffraction analysis were grown by slow evaporation of hexane from the solution of the title compound. All ellipsoids are drawn at 50% probability. The structure exhibits disorder at the triflate moieties (displayed in light blue).

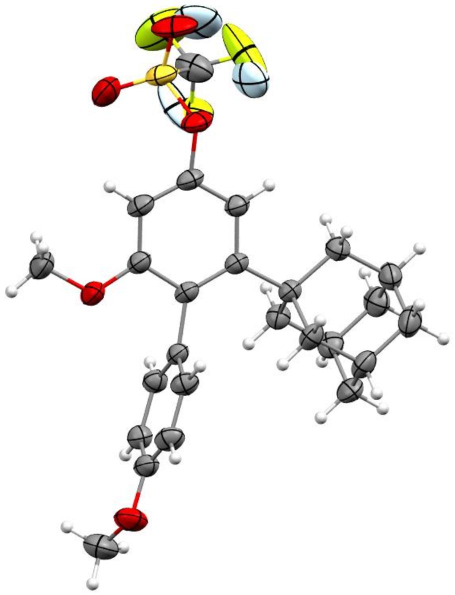

1-(*o*-Tolyl)adamantane (12a)

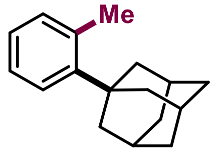

Prepared according to the general cross-coupling procedure B. The title product was obtained after purification by column chromatography (hexane) as a white solid. 44.0 mg (97%). R_f = 0.55 (hexane). M.p. 85 – 86°C. ^{1}H NMR (600 MHz, CD_2Cl_2) δ 7.32 (d, *J* = 8.1 Hz, 1H), 7.16 – 7.12 (m, 1H), 7.11 – 7.06 (m, 2H), 2.62 (s, 3H), 2.10 (br, 9H), 1.81 (br, 6H). ^{13}C NMR (151 MHz, CD_2Cl_2) δ 148.4, 136.6, 133.4, 126.3, 126.2, 126.0, 41.7, 38.3, 37.3, 29.7, 23.6. MS (70eV, EI): *m/z* (%): 227 (14), 226 (73) $[M]^+$, 183 (13), 170 (22), 169 (100), 168 (7), 167 (5), 155 (8), 154 (11), 153 (7), 142 (5), 141 (9), 135 (11), 129 (9), 128 (12), 115 (14), 105 (15), 94 (21), 93 (7), 91 (15), 79 (13), 77 (9). HRMS (APCI) calculated for $C_{17}H_{22}$: 226.1716 $[M]^+$, found: 226.1708.

1-(2-Butylphenyl)adamantane (12b)

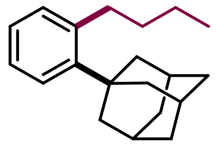

Prepared according to the general cross-coupling procedure B. The title product was obtained after purification by column chromatography (hexane) as a colorless oil. 39.0 mg (73%). R_f = 0.62 (hexane). ^{1}H NMR (600 MHz, CD_2Cl_2) δ 7.30 – 7.27 (m, 1H), 7.18 – 7.15 (m, 1H), 7.13 – 7.09 (m, 2H), 2.95 – 2.87 (m, 2H), 2.11 (br, 3H), 2.09 – 2.05 (m, 6H), 1.84 – 1.77 (m, 6H), 1.63 – 1.57 (m, 2H), 1.52 – 1.44 (m, 2H), 0.99 (t, *J* = 7.3 Hz, 3H). ^{13}C NMR (151 MHz, CD_2Cl_2) δ 147.7, 142.3, 132.4, 126.2, 126.0, 125.9, 42.7, 38.2, 37.3, 36.2, 34.6, 29.8, 23.7, 14.2. MS (70eV, EI): *m/z* (%): 269 (20), 268 (89) $[M]^+$, 226 (10), 225 (45), 212 (7), 211 (24), 183 (6), 169 (16), 168 (6), 167 (15), 165 (7), 159 (9), 156 (9), 155 (57), 154 (7), 153 (9), 152 (6), 145 (7), 143 (17), 142 (8), 141 (22), 136 (11), 135 (100), 132 (5), 131 (11), 130 (12), 129 (27), 128 (24), 127 (7), 119 (6), 117 (13), 116 (6), 115 (22), 107 (7), 105 (41), 95 (7), 94 (21), 93 (13), 92 (8), 91 (29), 81 (7), 79 (24), 77 (14), 67 (10), 65 (5), 57 (20), 55 (9). HRMS (APCI) calculated for $C_{20}H_{28}$: 268.2186 $[M]^+$, found: 268.2176.

(2-(Adamantan-1-yl)-4-fluorobenzyl)trimethylsilane (12c)

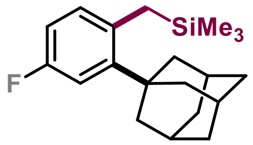

Prepared according to the general cross-coupling procedure B. The title product was obtained after purification by column chromatography (hexane) as a white solid. 58.7 mg (93%). R_f = 0.61 (hexane). M.p. 100 – 101°C. ^{1}H NMR (600 MHz, CD_2Cl_2) δ 6.99 (dd, *J* = 12.8, 2.8 Hz, 1H), 6.95 (dd, *J* = 8.3, 6.7 Hz, 1H), 6.76 (ddd, *J* = 8.2, 8.2, 2.8 Hz, 1H), 2.49 (s, 2H), 2.10 (br, 3H), 2.05 – 2.01 (m, 6H), 1.81 – 1.74 (m, 6H), 0.03 (s, 9H). ^{13}C NMR (151 MHz, CD_2Cl_2) δ 160.6 (d, *J* = 239.7 Hz), 149.0 (d, *J* = 5.3 Hz), 135.3 (d, *J* = 3.0 Hz), 132.4 (d, *J* = 7.7 Hz), 113.5 (d, *J* = 22.0 Hz), 112.0 (d, *J* = 20.7 Hz), 42.1, 38.0, 37.1, 29.6, 24.8, -0.5. ^{19}F NMR (564 MHz, CD_2Cl_2) δ -120.37 – -120.44 (m). MS (70eV, EI): *m/z* (%): 317 (3), 316 (10) $[M]^+$, 224 (19), 209 (9), 195 (13), 186 (6), 185 (8), 183 (12), 182 (24), 181 (47), 169 (6), 168 (6), 167 (14), 165 (9), 159 (6), 157 (15), 156 (20), 155 (15), 146 (8), 145 (7), 144 (20), 143 (36), 142 (10), 141 (7),

135 (8), 133 (8), 131 (8), 130 (15), 129 (10), 128 (6), 123 (9), 115 (7), 105 (7), 104 (7), 93 (7), 91 (10), 81 (6), 79 (16), 77 (10), 74 (8), 73 (100), 67 (7), 59 (7). HRMS (APCI) calculated for $C_{20}H_{30}FSi$: 317.2095 $[M+H]^+$, found: 317.2091.

1-(2-Cyclopropyl-5-fluorophenyl)adamantane (12d)

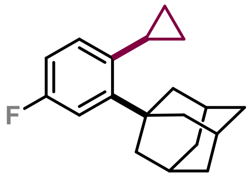

Prepared according to the general cross-coupling procedure B, but using cyclopropyl magnesium chloride (0.3 mmol) instead of the corresponding organozinc. The title product was obtained after purification by column chromatography (hexane) as a white solid. 25.6 mg (47%). R_f = 0.54 (hexane). M.p. 102 – 103°C. ^1H NMR (600 MHz, CD_2Cl_2) δ 7.04 (dd, *J* = 12.5, 2.5 Hz, 1H), 6.82 – 6.74 (m, 2H), 2.48 – 2.42 (m, 1H), 2.20 – 2.17 (m, 6H), 2.11 (br, 3H), 1.82 – 1.77 (m, 6H), 1.08 – 0.99 (m, 2H), 0.77 – 0.73 (m, 2H). ^{13}C NMR (151 MHz, CD_2Cl_2) δ 160.9 (d, *J* = 241.2 Hz), 151.2 (d, *J* = 6.2 Hz), 137.1 (d, *J* = 2.4 Hz), 126.0 (d, *J* = 8.4 Hz), 113.0 (d, *J* = 22.1 Hz), 111.8 (d, *J* = 20.2 Hz), 41.6, 38.2, 36.8, 29.3, 14.2, 10.5. ^{19}F NMR (564 MHz, CD_2Cl_2) δ -118.76 – -118.84 (m). MS (70eV, EI): *m/z* (%): 271 (5), 270 (21) $[M]^+$, 242 (6), 241 (27), 213 (14), 199 (10), 187 (6), 186 (8), 185 (36), 184 (8), 183 (16), 175 (5), 173 (11), 171 (9), 170 (8), 165 (11), 163 (16), 162 (100), 161 (13), 160 (14), 159 (38), 152 (6), 149 (11), 148 (15), 147 (32), 146 (32), 136 (11), 135 (97), 134 (13), 133 (25), 127 (6), 115 (5), 109 (14), 107 (10), 95 (6), 94 (8), 93 (24), 92 (5), 91 (12), 81 (11), 79 (29), 77 (13), 67 (15), 55 (10), 53 (7). HRMS (APCI) calculated for $C_{19}H_{24}F$: 271.1857 $[M+H]^+$, found: 271.1846.

1-([1,1'-Biphenyl]-2-yl)adamantane (12e)

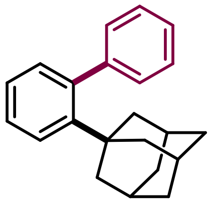

Prepared according to the general cross-coupling procedure B. The title product was obtained after purification by column chromatography (hexane) as a colorless oil. 48.5 mg (84%). R_f = 0.46 (hexane). ^1H NMR (600 MHz, CD_2Cl_2) δ 7.53 (d, *J* = 8.2 Hz, 1H), 7.35 – 7.30 (m, 4H), 7.28 – 7.25 (m, 2H), 7.14 (dd, *J* = 7.3, 7.3 Hz, 1H), 6.96 – 6.94 (m, 1H), 1.89 (br, 9H), 1.65 – 1.49 (m, 6H). ^{13}C NMR (151 MHz, CD_2Cl_2) δ 148.3, 145.9, 142.6, 133.2, 130.5, 127.6, 127.4, 126.7, 125.0, 43.5, 39.2, 36.9, 29.6. MS (70eV, EI): *m/z* (%): 289 (24), 288 (100) $[M]^+$, 232 (6), 231 (21), 217 (9), 216 (14), 215 (16), 203 (11), 202 (9), 193 (18), 192 (60), 191 (22), 189 (6), 180 (8), 179 (33), 178 (29), 167 (6), 166 (6), 165 (24), 153 (5), 152 (12), 135 (9), 79 (7), 77 (6). HRMS (APCI) calculated for $C_{22}H_{24}$: 288.1873 $[M]^+$, found: 288.1864.

1-(2'-Methyl-[1,1'-biphenyl]-2-yl)adamantane (12f)

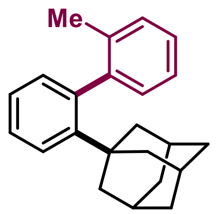

Prepared according to the general cross-coupling procedure B. The yield was determined from the crude via quantitative ^{1}H NMR (53%). The title product was then isolated after purification by column chromatography (hexane) and preparative HPLC (95:5 Hexane/EtOAc) as a white solid. 23.4 mg (39%). R_f = 0.49 (hexane). M.p. 95 – 96°C. ^{1}H NMR (600 MHz, CD_2Cl_2) δ 7.55 (d, *J* = 8.1 Hz, 1H), 7.33 – 7.29 (m, 1H), 7.25 – 7.19 (m, 2H), 7.16 (d, *J* = 6.2 Hz, 3H), 6.85 (d, *J* = 7.3 Hz, 1H), 2.01 (s, 3H), 1.97 – 1.75 (m, 9H), 1.66 – 1.47 (m, 6H). ^{13}C NMR (151 MHz, CD_2Cl_2) δ 148.1, 145.2, 141.1, 136.6, 132.7, 130.8, 129.9, 127.4, 127.3, 127.1, 125.5, 124.6, 43.0, 39.1, 37.0, 29.6, 21.1. MS (70eV, EI): *m/z* (%): 303 (26), 302 (100) $[M]^+$, 287 (11), 246 (6), 245 (23), 231 (9), 230 (13), 229 (9), 217 (12), 216 (10), 215 (19), 207 (9), 206 (19), 205 (15), 204 (5), 203 (15), 202 (11), 194 (7), 193 (32), 192 (27), 191 (38), 190 (6), 189 (9), 181 (5), 180 (6), 179 (25), 178 (29), 167 (6), 166 (13), 165 (29), 152 (9), 135 (18), 115 (7), 95 (8), 93 (8), 91 (9), 79 (12), 77 (8). HRMS (APCI) calculated for $C_{23}H_{26}$: 302.2029 $[M]^+$, found: 302.2022.

2-(2-(Adamantan-1-yl)phenyl)thiophene (12g)

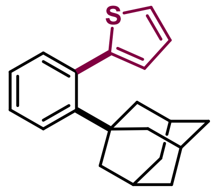

Prepared according to the general cross-coupling procedure B. The title product was obtained after purification by column chromatography (hexane) as a colorless oil. 57.7 mg (98%). R_f = 0.50 (hexane). ^{1}H NMR (600 MHz, CD_2Cl_2) δ 7.53 (d, *J* = 8.2 Hz, 1H), 7.36 – 7.32 (m, 2H), 7.15 (d, *J* = 4.1 Hz, 2H), 7.03 – 6.99 (m, 1H), 6.97 (d, *J* = 3.6 Hz, 1H), 1.98 (br, 6H), 1.95 (br, 3H), 1.71 – 1.57 (m, 6H). ^{13}C NMR (151 MHz, CD_2Cl_2) δ 150.5, 146.3, 135.1, 134.0, 128.8, 128.2, 126.8, 126.3, 125.1, 125.0, 43.1, 39.2, 36.9, 29.7. MS (70eV, EI): *m/z* (%): 296 (7), 295 (23), 294 (100) $[M]^+$, 293 (6), 261 (21), 235 (7), 223 (7), 222 (28), 221 (6), 205 (9), 204 (7), 203 (21), 202 (7), 199 (5), 198 (8), 197 (18), 191 (6), 185 (15), 184 (19), 179 (13), 178 (12), 171 (6), 167 (6), 166 (6), 165 (19), 153 (5), 152 (10), 141 (9), 128 (5), 115 (9), 93 (5), 91 (5), 79 (8), 77 (6). HRMS (APCI) calculated for $C_{20}H_{23}S$: 295.1515 $[M+H]^+$, found: 295.1512.

1-(4'-Chloro-[1,1'-biphenyl]-2-yl)adamantane (12h)

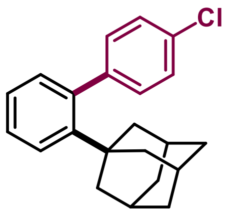

Prepared according to the general cross-coupling procedure B. The title product was obtained after purification by column chromatography (hexane) as a white solid. 48.9 mg (76%). R_f = 0.55 (hexane). M.p. 125 – 126°C. ^{1}H NMR (600 MHz, CD_2Cl_2) δ 7.53 (d, *J* = 8.3 Hz, 1H), 7.34 – 7.30 (m, 3H), 7.24 –

7.20 (m, 2H), 7.15 (dd, J = 7.3, 7.3 Hz, 1H), 6.91 (dd, J = 7.4, 1.5 Hz, 1H), 1.91 (br, 3H), 1.89 – 1.86 (m, 6H), 1.67 – 1.52 (m, 6H). ^{13}C NMR (151 MHz, CD_2Cl_2) δ 148.3, 144.5, 141.2, 133.1, 132.6, 131.9, 127.9, 127.5, 126.9, 125.1, 43.6, 39.2, 36.9, 29.6. MS (70eV, EI): *m/z* (%): 325 (8), 324 (35, ^{37}Cl), 323 (24), 322 (100, ^{35}Cl) $[M]^+$, 287 (13), 265 (13), 231 (10), 230 (26), 229 (41), 228 (25), 227 (15), 226 (27), 225 (12), 217 (7), 216 (10), 215 (25), 214 (8), 213 (18), 212 (12), 204 (6), 203 (14), 202 (17), 201 (8), 199 (9), 193 (9), 192 (17), 191 (63), 190 (7), 189 (12), 179 (14), 178 (27), 177 (7), 176 (9), 166 (12), 165 (26), 152 (17), 151 (7), 135 (29), 115 (7), 114 (6), 107 (6), 101 (6), 95 (9), 94 (6), 93 (12), 91 (9), 79 (18), 77 (12), 67 (10), 55 (8). HRMS (APCI) calculated for $C_{22}H_{23}{}^{35}Cl$: 322.1483 $[M]^+$, found: 322.1477.

1-(4-Fluoro-4'-methoxy-[1,1'-biphenyl]-2-yl)adamantane (12i)

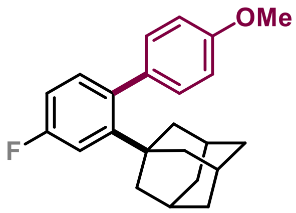

Prepared according to the general cross-coupling procedure B. The title product was obtained after purification by column chromatography (10:1 Hexane/EtOAc) as a colorless oil. 50.3 mg (75%). R_f = 0.66 (10:1 Hexane/EtOAc). ^{1}H NMR (600 MHz, CD_2Cl_2) δ 7.21 (dd, J = 12.8, 2.7 Hz, 1H), 7.16 – 7.12 (m, 2H), 6.93 – 6.90 (m, 1H), 6.88 – 6.86 (m, 2H), 6.85 – 6.81 (m, 1H), 3.84 (s, 3H), 1.90 (br, 3H), 1.88 – 1.84 (m, 6H), 1.67 – 1.50 (m, 6H). ^{13}C NMR (151 MHz, CD_2Cl_2) δ 162.4 (d, J = 243.6 Hz), 158.8, 151.5 (d, J = 6.1 Hz), 138.3 (d, J = 2.5 Hz), 136.9, 135.0 (d, J = 7.5 Hz), 131.7, 113.6 (d, J = 22.6 Hz), 112.8, 111.4 (d, J = 20.9 Hz), 55.6, 43.1, 39.2, 36.8, 29.5. ^{19}F NMR (564 MHz, CD_2Cl_2) δ -116.42 – -116.49 (m). MS (70eV, EI): *m/z* (%): 338 (3), 337 (25), 336 (100) $[M]^+$, 240 (19), 239 (10), 227 (16), 226 (7), 209 (9), 196 (7), 183 (12), 170 (5), 135 (10), 79 (6). HRMS (APCI) calculated for $C_{23}H_{26}FO$: 337.1962 $[M+H]^+$, found: 337.1958.

2'-(Adamantan-1-yl)-4'-chloro-*N,N*-dimethyl-[1,1'-biphenyl]-4-amine (12j)

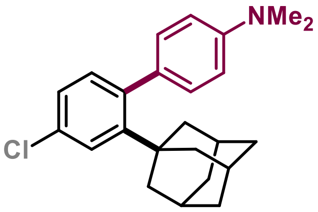

Prepared according to the general cross-coupling procedure B. The title product was obtained after purification by column chromatography (95:5 Hexane/EtOAc) as a white solid. 52.4 mg (72%). R_f = 0.59 (95:5 Hexane/EtOAc). M.p. 155 – 156°C. ^{1}H NMR (600 MHz, CD_2Cl_2) δ 7.46 (d, J = 2.2 Hz, 1H), 7.10 (dd, J = 8.1, 2.0 Hz, 1H), 7.07 – 7.03 (m, 2H), 6.89 (d, J = 8.1 Hz, 1H), 6.72 – 6.68 (m, 2H), 2.97 (s, 6H), 1.89 (br, 9H), 1.67 – 1.50 (m, 6H). ^{13}C NMR (151 MHz, CD_2Cl_2) δ 151.1, 149.8, 141.7, 135.3, 132.9, 132.3, 131.0, 126.9, 124.8, 111.4, 43.1, 40.8, 39.3, 36.9, 29.5. MS (70eV, EI): *m/z* (%): 368 (9), 367 (35, ^{37}Cl), 366 (28), 365 (100, ^{35}Cl) $[M]^+$, 364 (7), 79 (5). HRMS (APCI) calculated for $C_{24}H_{29}N^{35}Cl$: 366.1983 $[M+H]^+$, found: 366.2000.

5-(2-(Adamantan-1-yl)-4-chlorophenyl)benzo[1,3]dioxole (12k)

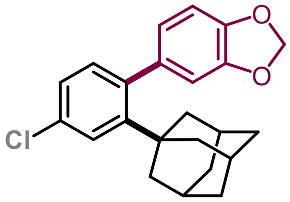

Prepared according to the general cross-coupling procedure B. The title product was obtained after purification by column chromatography (9:1 Hexane/DCM) as a white solid. 33.6 mg (46%). R_f = 0.44 (9:1 Hexane/DCM). M.p. 63 – 64°C. ^{1}H NMR (600 MHz, CD_2Cl_2) δ 7.48 (d, *J* = 2.2 Hz, 1H), 7.11 (dd, *J* = 8.1, 2.2 Hz, 1H), 6.90 (d, *J* = 8.1 Hz, 1H), 6.78 (d, *J* = 7.9 Hz, 1H), 6.71 (d, *J* = 1.6 Hz, 1H), 6.68 (dd, *J* = 7.9, 1.6 Hz, 1H), 6.00 (d, *J* = 13.0 Hz, 2H), 1.96 – 1.83 (m, 9H), 1.68 – 1.52 (m, 6H). ^{13}C NMR (151 MHz, CD_2Cl_2) δ 150.7, 147.0, 146.8, 140.6, 138.1, 134.9, 133.4, 127.2, 124.9, 123.8, 111.2, 107.4, 101.6, 43.2, 39.4, 36.8, 29.5. MS (70eV, EI): *m/z* (%): 369 (9), 368 (35, ^{37}Cl), 367 (26), 366 (100, ^{35}Cl) $[M]^+$, 308 (7), 271 (5), 270 (7), 269 (11), 256 (6), 244 (8), 243 (5), 227 (7), 217 (5), 216 (9), 215 (17), 203 (7), 202 (10), 201 (6), 199 (8), 189 (10), 178 (9), 176 (7), 165 (11), 163 (7), 152 (10), 135 (12), 108 (5), 107 (6), 101 (5), 93 (10), 91 (6), 79 (14), 77 (7), 67 (8), 55 (7). HRMS (APCI) calculated for $C_{23}H_{24}{}^{35}ClO_2$: 367.1459 $[M+H]^+$, found: 367.1455.

3. Crystallographic data

Compound	1	10f	10g
CCDC number	1946371	1946658	1946657
Formula	$C_{18}H_{20}BrF_3O_4S$	$C_{22}H_{23}F_3O_4S_2$	$C_{25}H_{27}F_3O_5S$
Formula Weight	469.31	472.52	496.52
Colour	colourless	yellowish	colourless
Shape	rod	plate	plate
Size/mm^3	0.27×0.08×0.03	0.75×0.43×0.20	0.85×0.49×0.23
Temperature/K	100	296(2)	296(2)
Crystal System	triclinic	monoclinic	triclinic
Space Group	*P*-1	$P2_1/n$	*P*-1
a/Å	6.5880(8)	13.8850(19)	10.2800(11)
b/Å	16.667(2)	19.562(3)	10.3310(10)
c/Å	17.334(2)	15.969(2)	12.7404(13)
$\alpha/^\circ$	80.426(2)	90	106.242(2)
$\beta/^\circ$	89.644(2)	94.617(3)	106.271(3)
$\gamma/^\circ$	85.417(2)	90	105.274(2)
V/Å^3	1870.8(4)	4323.3(10)	1158.1(2)
Z	4	8	2
Z'	2	2	1
ρ_{calc}/g cm^{-3}	1.666	1.452	1.424
μ/mm^{-1}	2.360	0.299	0.199
Radiation type	MoK_α	MoK_α	MoK_α
Wavelength/Å	0.71073	0.71073	0.71073
$\Theta_{min}/^\circ$	1.191	1.802	1.802
$\Theta_{max}/^\circ$	31.042	35.608	34.708
Measured Refl.	28884	149054	73558
Independent Refl.	10840	19822	9865
Reflections with I > 2(I)	7997	7941	7316
R_{int}	0.0556	0.0769	0.0214
Parameters	489	731	337
Restraints	0	265	21
Largest Peak/eÅ^{-3}	1.085	0.340	0.436
Largest Hole/eÅ^{-3}	-1.184	-0.307	-0.373
GooF on F^2	0.906	1.003	1.059
wR_2 (all data)	0.1537	0.1994	0.1435
wR_2	0.1308	0.1331	0.1222
R_1 (all data)	0.0735	0.1725	0.0641
R_1	0.0485	0.0601	0.0440

4. Computational details

All the calculations reported in the manuscript were carried out using Gaussian16 (A.03)[9] at the B3LYP-D3 level of theory[10] (other functionals and the ORCA software[11] were used for the benchmarking, see below). For optimizations and frequency calculations, LanL2DZ basis set[12] and the corresponding pseudopotential was used for Pd center and 6-31G(d) for the rest of the atoms.[13] Then, potential energies were further refined at the triple-zeta level using the Def2TZVP basis set.[14] Solvent was considered implicitly, in both optimization/frequency calculations and single point calculations, using the CPCM model and the experimental solvent (tetrahydrofuran),[15] while other solvent methods were tested in the benchmarking (see below). Dispersion corrections (using the original damping function,[10d] unless stated otherwise) were turned off when needed for analyzing the dispersion energy contribution to the free energy activation barriers, following the equation:

$$\Delta\Delta G^{disp} = \Delta G^{act}_{B3LYP(D3)} - \Delta G^{act}_{B3LYP}$$

The nature of the stationary points was checked by frequency analysis (zero imaginary frequency for minima and one imaginary frequency for transition states). Free energy correction of 1.89 kcal/mol was added to every point at 298.15 K and 1 atm.[16] All energies in the main text are free energies in kcal/mol.

3D structures were prepared using CYLview.[17]

4.1. Benchmarking of the Methodology

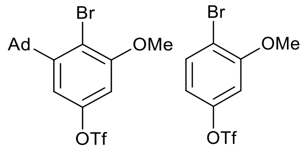

Ar^1Br **Ar^2Br**

Basis set influence:

Method	Basis set	Solvation Method	ΔG$^{Act.}$ (Ar^1Br)	ΔG$^{Act.}$ (Ar^2Br)
**B3LYPD3**	6-31G*/LANL2DZ	CPCM (THF)	23.8	24.6
**B3LYPD3**	Def2TZVP	CPCM (THF)	26.3	26.0
**B3LYPD3**	Def2QZVP	CPCM (THF)	26.0	25.6

Solvation model influence:

Method	Basis set	Solvation Method	ΔG$^{Act.}$ (Ar^1Br)	ΔG$^{Act.}$ (Ar^2Br)
**B3LYPD3**	Def2TZVP	CPCM (THF)	26.3	26.0
**B3LYPD3**	Def2TZVP	SMD(THF)	26.1	25.7
**B3LYPD3**	Def2TZVP	PCM(THF)	25.9	25.6
**B3LYPD3**	Def2TZVP	GAS PHASE	27.4	27.4

Functional influence:

Method	Basis set	Solvation Method	ΔG$^{Act.}$ (Ar^{1}Br)	ΔG$^{Act.}$ (Ar^{2}Br)
**B3LYPD3**	Def2TZVP	CPCM (THF)	26.3	26.0
**M06**	Def2TZVP	CPCM (THF)	29.2	28.9
**M06L**	Def2TZVP	CPCM (THF)	25.8	26.0
**M06D3**	Def2TZVP	CPCM (THF)	28.1	30.2
**M06LD3**	Def2TZVP	CPCM (THF)	25.2	26.7
**B97D**	Def2TZVP	CPCM (THF)	25.3	27.6
**wB97xD**	Def2TZVP	CPCM (THF)	29.7	30.2
**PBE0D3**	Def2TZVP	CPCM (THF)	27.2	26.8
**PBEPBED3**	Def2TZVP	CPCM (THF)	24.0	23.7
**DLPNO-CCSD(T)**	Def2TZVPP	CPCM(THF) from DFT	25.5	24.5
**DLPNO-CCSD(T)**	Def2TZVPP	GAS PHASE	26.6	25.9

Dispersion model influence:

Method	Basis set	Solvation Method	ΔG$^{Act.}$ (Ar^{1}Br)	ΔG$^{Act.}$ (Ar^{2}Br)
**B3LYPD3**	Def2TZVP	CPCM (THF)	26.3	26.0
**B3LYPD2**	Def2TZVP	CPCM (THF)	26.6	27.9
**B3LYPD3(BJ)**	Def2TZVP	CPCM (THF)	26.0	26.2
**B3LYPD4***	Def2TZVP	CPCM (THF)	24.2	23.3

*These calculations were carried out in ORCA program

ΔΔG$^{Disp.}$ Functional dependency (basis set Def2TZVP, solvation method = CPCM(THF):

Method	ΔG$^{Act.}$ (ArBr)	ΔG$^{Act.}$ (Ar^{2}Br)	ΔΔG$^{Disp.}$ (Ar^{1}Br)	ΔΔG$^{Disp.}$ (Ar^{2}Br)
**B3LYPD3**	26.3	26.0	-1.3	+4.7
**B3LYP**	27.6	21.3		
**M06LD3**	25.2	26.7	-0.6	+0.7
**M06L**	25.8	26.0		
**M06D3**	28.1	30.2	-0.9	+1.2
**M06**	29.2	28.9		
**PBE0D3**	27.2	26.8	-1.3	+3.2
**PBE0**	28.5	23.6		
**PBEPBED3**	25.5	20.8	-1.5	+2.9
**PBEPBE**	24.0	23.7		
**B3LYPD4***	24.2	23.3	-1.9	+3.4
**B3LYP***	26.1	19.9		

*These calculations were carried out in ORCA program.

The dispersion values show a significant effect in all the functionals except M06 and M06L, both including dispersion in the intrinsic parametrization of the functional and showing that the use of Grimme's dispersion correction would not be appropriate for these cases. D4 values are lower due to the stabilization of the transition state in ORCA program respect to the Gaussian (B3LYP without dispersion has a barrier 1.4-1.5 lower in ORCA program).

4.2. Cartesian Coordinates of Calculated compounds

Energies are given in Hartrees and correspond to the potential energy with the large basis set (first line) and to the free energy correction (second line). The energies used to compute all the profiles were calculated by the sum of E + G_{corr}.

P*t*Bu$_3$

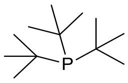

E_{disp} = -815.1325289
E_{nodisp} = -815.0838429
G_{corr} = 0.331600
H_{corr} = 0.390755

P	0.10068100	1.66062300	-0.05120700
C	-1.72634300	2.31567000	0.07675300
C	-2.42871900	2.14476300	-1.28910700
C	-2.47896900	1.36942800	1.04622300
C	-1.92009700	3.77247900	0.54027600
H	-2.07205200	2.84934900	-2.04294400
H	-2.31827300	1.12813300	-1.68239400
H	-3.50321200	2.33055100	-1.15850600
H	-2.39645200	0.32486700	0.72554900
H	-2.12830300	1.43430500	2.07628500
H	-3.54516800	1.63492800	1.04971200
H	-2.99139000	4.01925100	0.52797400
H	-1.56644200	3.93475400	1.56100700
H	-1.41079300	4.48691700	-0.11069400
C	1.07179500	2.33837500	1.49158400
C	1.45641200	3.83060100	1.48019600
C	0.25634900	2.05907000	2.77413300
C	2.35686600	1.48290800	1.62755800
H	2.14690800	4.07635300	0.67012800
H	0.58499500	4.48350400	1.39053000
H	1.96319600	4.08327900	2.42255700
H	-0.06010600	1.01214500	2.83889600
H	0.89082500	2.26419900	3.64677100
H	-0.62640600	2.69503600	2.86440700
H	2.87185200	1.75585800	2.55900800
H	2.11883700	0.41448900	1.67874900
H	3.06571900	1.63150700	0.81286300
C	0.89513900	2.46027800	-1.63641300
C	2.43073300	2.29876500	-1.57760800
C	0.43904300	1.60880700	-2.84830300
C	0.57543600	3.94255300	-1.91073100
H	2.89882200	2.94593800	-0.83341400
H	2.72630700	1.26350500	-1.37447700
H	2.85179600	2.57424800	-2.55378800
H	-0.62937200	1.68054200	-3.05211500
H	0.96469900	1.95662400	-3.74831600
H	0.68623900	0.55082900	-2.70553900
H	1.11386500	4.27085700	-2.81130500
H	-0.48802600	4.11111900	-2.09446500
H	0.88270600	4.59333500	-1.08862400

(P*t*Bu$_3$)$_2$Pd

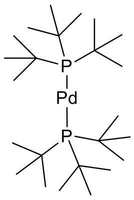

E_{disp} = -1758.330911
E_{nodisp} = -1758.201564
G_{corr} = 0.680107
H_{corr} = 0.786948

P	0.09430500	1.67378800	-0.04505200
C	-1.73047600	2.32444500	0.09174600
C	-2.43333000	2.12922700	-1.26962600
C	-2.49757900	1.41529600	1.08431800
C	-1.89567000	3.79358800	0.52646900
H	-2.07737300	2.81928600	-2.03701700
H	-2.31807200	1.10342600	-1.63680900
H	-3.50708600	2.31761400	-1.14013300
H	-2.43604400	0.36490200	0.77956200
H	-2.13790400	1.48889100	2.11027500
H	-3.55528300	1.71221800	1.08632200
H	-2.96217500	4.05608000	0.50339200
H	-1.54577100	3.96601500	1.54697300
H	-1.37293200	4.48927700	-0.13356800
C	1.08869400	2.33350700	1.48731800
C	1.46601700	3.82737900	1.45764400
C	0.27362600	2.05482800	2.76918900
C	2.38036600	1.48982900	1.62933600
H	2.16330200	4.06315200	0.65025900
H	0.59577100	4.47956200	1.35596700
H	1.96521500	4.08931500	2.40054100
H	-0.04617600	1.00826500	2.82149500
H	0.91182200	2.25181200	3.64047200
H	-0.60489200	2.69587000	2.86407100
H	2.89631900	1.79085000	2.55135900
H	2.14435600	0.42252800	1.70038700
H	3.08150200	1.62423300	0.80611600
C	0.88612900	2.42868000	-1.65019200
C	2.41689200	2.23253300	-1.59153300
C	0.41093800	1.59350200	-2.86542900
C	0.58847400	3.91695900	-1.91691300
H	2.90213500	2.87831400	-0.85723700
H	2.67932500	1.19138600	-1.37361700
H	2.84080500	2.48447300	-2.57235800
H	-0.65784800	1.67687500	-3.06055700
H	0.93629800	1.94881900	-3.76246300
H	0.64663800	0.53285800	-2.72600700
H	1.13752200	4.23682200	-2.81308200
H	-0.47142200	4.09966200	-2.10865200
H	0.90058400	4.56352400	-1.09373100
Pd	0.14432200	-0.67028300	-0.12526900
P	0.22984500	-3.01250800	-0.23016000
C	-0.00408000	-3.80068200	1.52885000
C	-1.16479900	-3.70273000	-1.39295000

C	1.94445100	-3.59612000	-0.93176700
C	-0.34097200	-5.30418300	1.55820900
C	1.27844000	-3.56489800	2.35642500
C	-1.11938200	-3.01869500	2.26685100
C	-2.52482500	-3.57183400	-0.67293100
C	-1.26563800	-2.77860200	-2.63232000
C	-0.99462700	-5.15963600	-1.86572200
C	1.99378600	-3.29043100	-2.44491100
C	3.05421400	-2.71965300	-0.29905300
C	2.29454600	-5.08133500	-0.71644700
H	-1.31271400	-5.52040100	1.10810200
H	0.41082200	-5.91404000	1.05217600
H	-0.38832500	-5.64006800	2.60310500
H	1.57783900	-2.51108100	2.34027100
H	1.07730100	-3.83795900	3.40054600
H	2.12016600	-4.17450500	2.02223100
H	-1.18522500	-3.39086000	3.29838400
H	-0.88832600	-1.94852100	2.30178500
H	-2.10464400	-3.13500400	1.81624400
H	-2.64145300	-4.27812100	0.15123800
H	-2.68031500	-2.55641500	-0.29202200
H	-3.32527300	-3.78300000	-1.39396900
H	-0.38345100	-2.80830800	-3.27117800
H	-2.12173600	-3.10013000	-3.24109900
H	-1.42894500	-1.73819200	-2.33103000
H	-1.87239700	-5.44782400	-2.46015500
H	-0.11855800	-5.28846200	-2.50572300
H	-0.91538300	-5.86555700	-1.03592600
H	1.35133200	-3.94736100	-3.03434200
H	1.71989800	-2.25012300	-2.65261600
H	3.02151300	-3.44149900	-2.80006500
H	2.85905900	-1.65601300	-0.47373400
H	3.16803300	-2.86814100	0.77441000
H	4.01381100	-2.97590400	-0.76855900
H	3.25034200	-5.29934700	-1.21223600
H	2.41857800	-5.32953300	0.34035300
H	1.54583500	-5.75563200	-1.13794300

Ar^{1}Br:

Ar^{1}Br

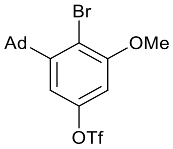

E_{disp} = -4271.325542
E_{nodisp} = -4271.260446
G_{corr} = 0.312226
H_{corr} = 0.392141

C	1.28774800	-6.22898300	-0.12953500
C	-1.15609100	-6.64737500	-0.10012100
C	1.45551900	-7.62713400	-0.11607200
C	-0.94535300	-8.02816000	-0.08337200
C	0.36481300	-8.47470300	-0.09086700
H	2.43669100	-8.07464200	-0.11772700
H	-1.75899800	-8.73917900	-0.06210100
O	-2.39080800	-6.09439900	-0.09044600
C	-3.52888500	-6.95502000	-0.01861100
H	-3.58915000	-7.60925000	-0.89645300
H	-3.51011100	-7.56168200	0.89471300
H	-4.39383100	-6.29148400	0.00102800
O	0.57338100	-9.87510000	-0.13040100
S	1.00131000	-10.65200000	1.24528800
O	1.33133900	-12.00442400	0.83129700
O	1.89040000	-9.83454400	2.05666100
C	-0.65981400	-10.71649900	2.12340600
F	-0.92789000	-9.52261500	2.64615100
F	-1.60793400	-11.04829100	1.24799200
F	-0.58741400	-11.63103700	3.08428000
C	2.55106200	-5.33821800	-0.14357000
C	2.59670600	-4.46192400	-1.43075700
C	3.85961500	-6.18266700	-0.14030200
C	2.60552100	-4.44091200	1.12909300
H	2.61992300	-5.12458800	-2.30655700
H	1.69759000	-3.85016200	-1.51602800
C	3.83715300	-3.54608500	-1.42481300
H	3.88640700	-6.83828200	-1.02035500
H	3.89117100	-6.82401700	0.75003300
C	5.11466800	-5.28456900	-0.15204400
H	1.70704800	-3.82773100	1.21066800
H	2.63473400	-5.08947200	2.01517000
C	3.84567800	-3.52520600	1.09985900
H	3.81609100	-2.91957700	-2.32604300
C	5.11337800	-4.40610400	-1.41665200
C	3.80810200	-2.64887400	-0.16968800
H	6.00187300	-5.93085000	-0.14965900
C	5.12195200	-4.38511600	1.09763000
H	3.83032700	-2.88390600	1.99073300
H	6.00609700	-3.76612700	-1.43204400
H	5.14898900	-5.03601200	-2.31607200
H	2.90111500	-2.02909200	-0.17175400
H	4.66818500	-1.96563900	-0.17826100
H	5.16364600	-4.99995700	2.00711200
H	6.01469300	-3.74498100	1.09624600
C	-0.04635400	-5.76545700	-0.12323800
Br	-0.54327400	-3.90421100	-0.14305500

$^{Ar1}TS_{OxAdd}$

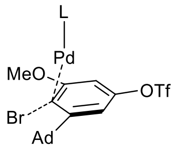

E_{disp} = -5214.481957
E_{nodisp} = -5214.334065
G_{corr} = 0.660654
H_{corr} = 0.785878

Br	3.50533600	-2.00564700	-3.38594600
C	1.72244700	-3.09374400	-2.74945100
C	1.77132700	-4.14608900	-1.78874400
C	0.78784200	-3.12096600	-3.84522400
C	0.83520700	-5.18904000	-1.96203000
C	-0.12186500	-4.16693700	-3.95757000
C	-0.05684700	-5.18580900	-3.01185800
H	0.79961000	-6.01911300	-1.26949300
H	-0.85081300	-4.22100800	-4.75418600
O	0.85644200	-2.10578100	-4.74392600
C	-0.10379900	-2.06147100	-5.79759200
H	-0.02407900	-2.94085100	-6.44824300
H	-1.12357000	-1.98681200	-5.40039300
H	0.13189800	-1.16299100	-6.36856300
O	-1.00889600	-6.23081900	-3.12249800
S	-0.52723100	-7.67223000	-3.72796200
O	0.80775900	-8.02106400	-3.26480800
O	-1.67045900	-8.55428800	-3.56695300
C	-0.38892200	-7.24860700	-5.55414800
F	-1.44102100	-6.51637100	-5.91870600
F	0.73174900	-6.56302000	-5.76527800
F	-0.36600700	-8.38467600	-6.24357700

C	2.67752900	-4.28224600	-0.53997500
C	3.51863500	-5.59019000	-0.65023200
C	1.76076500	-4.37608500	0.72204000
C	3.66906100	-3.12216900	-0.28262800
H	2.86819100	-6.45452400	-0.82720200
H	4.18692400	-5.51187300	-1.51831100
C	4.33818100	-5.82586800	0.63560400
H	1.02517000	-5.17957300	0.61034100
H	1.19760800	-3.43800200	0.81163400
C	2.58575900	-4.62968300	1.99765900
H	4.35613400	-3.02059300	-1.12324500
H	3.11153800	-2.17515500	-0.21355700
C	4.48929700	-3.34809700	1.00309800
H	4.90722900	-6.75812600	0.52528800
C	3.38233600	-5.94128100	1.84078300
C	5.30496700	-4.64782200	0.85993100
H	1.89905700	-4.71403100	2.85016400
C	3.55541000	-3.45623000	2.22049900
H	5.16967300	-2.49598400	1.13124400
H	3.95509500	-6.14474200	2.75569600
H	2.69302500	-6.78455300	1.69535200
H	6.00107100	-4.56463300	0.01415100
H	5.90769300	-4.81925900	1.76228700
H	2.99178100	-2.52223000	2.35013800
H	4.14090900	-3.61212100	3.13692500
Pd	1.36613400	-1.33925800	-1.73373400
P	0.58904000	0.64833800	-0.70587300
C	-1.01126700	0.30961900	0.33055900
C	-2.18539100	0.02989100	-0.63325900
C	-0.80660100	-1.00707200	1.12017400
C	-1.42821300	1.42843800	1.30489700
H	-2.52697300	0.92271000	-1.16024000
H	-1.92680300	-0.73808500	-1.37086200
H	-3.03550500	-0.34511200	-0.04907800
H	-0.58700100	-1.83913500	0.44252100
H	-0.00855000	-0.95710300	1.86014700
H	-1.73570600	-1.24355700	1.65583400
H	-2.37771600	1.15194300	1.78260300
H	-0.69871100	1.57673000	2.10438900
H	-1.58087100	2.38617900	0.80194000
C	1.92586000	1.43355300	0.45705600
C	1.70116900	2.91189800	0.83017300
C	2.01414900	0.60074500	1.75485100
C	3.31080200	1.28695700	-0.22081300
H	1.76991900	3.57301700	-0.03692200
H	0.73575600	3.08230500	1.31187000
H	2.48163100	3.22501700	1.53664900
H	2.14677400	-0.46500500	1.54075800
H	2.89091500	0.93357500	2.32497100
H	1.14448600	0.72321600	2.40303400
H	4.07922200	1.65079400	0.47422100
H	3.53140200	0.23938500	-0.44911000
H	3.40938800	1.85991700	-1.14202200
C	0.16250800	1.91319100	-2.11176800
C	1.47852800	2.45378000	-2.71280700
C	-0.53446600	1.14681400	-3.26515000
C	-0.71782700	3.10715300	-1.69403300
H	2.00972700	3.12836700	-2.03873600
H	2.15266000	1.64058300	-3.00297300
H	1.23977500	3.02589300	-3.61854500
H	-1.51330200	0.75144000	-2.99556900
H	-0.68303900	1.84046800	-4.10377100
H	0.08183300	0.31337100	-3.62139600
H	-0.83850200	3.78038200	-2.55337700
H	-1.71952400	2.79796500	-1.38630100
H	-0.27729500	3.68916100	-0.88135500

^{Ar1}Prod

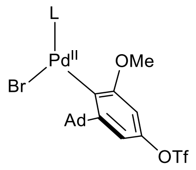

E_{disp} = -5214.520332

E_{nodisp} = -5214.367864

G_{corr} = 0.665132

H_{corr} = 0.788189

Br	2.93661900	-1.71500900	-3.30642100
C	0.89898000	-3.75711200	-2.11581500
C	1.32941600	-4.83412900	-1.31624700
C	0.13897300	-3.98442900	-3.28358500
C	0.98188600	-6.13817800	-1.72337300
C	-0.18700600	-5.28175400	-3.68957000
C	0.25917900	-6.32372400	-2.88771100
H	1.27160600	-7.01198800	-1.15748600
H	-0.76128300	-5.49651500	-4.58087200
O	-0.27447300	-2.87315700	-3.96314300
C	-0.91419100	-3.03882500	-5.22457100
H	-0.27827900	-3.59749800	-5.92294100
H	-1.88032100	-3.54946100	-5.12250700
H	-1.07731500	-2.03011900	-5.60680600
O	-0.11650600	-7.64112900	-3.27170600
S	0.99186500	-8.59282100	-3.99776600
O	2.31206300	-8.41873500	-3.41005300
O	0.35573300	-9.89362500	-4.12335600
C	1.04374300	-7.82335200	-5.71150700
F	-0.19884700	-7.56145200	-6.11674700
F	1.75183200	-6.69753300	-5.67315200
F	1.61569400	-8.69299300	-6.53909600
C	2.15609900	-4.63216700	-0.03761500
C	3.60910000	-4.21893000	-0.40533000
C	2.24004300	-5.91420400	0.83456500
C	1.53040000	-3.52054500	0.84843600
H	4.06220500	-5.01748400	-1.00863000
H	3.58952200	-3.31987100	-1.02842700
C	4.44660700	-3.97135200	0.86571100
H	2.71039300	-6.72459500	0.26415700
H	1.22935800	-6.24896700	1.10511600
C	3.07375600	-5.66962000	2.10898800
H	1.48067300	-2.57431600	0.27004800
H	0.49820600	-3.78551500	1.11043200
C	2.37171300	-3.26077300	2.10956000
H	5.46122700	-3.67293900	0.57134800
C	4.50512400	-5.25855600	1.71015800
C	3.79826100	-2.84485000	1.69497800
H	3.10490800	-6.59842400	2.69300300
C	2.42977900	-4.55127800	2.94959400
H	1.90318600	-2.45902800	2.69543100
H	5.11459400	-5.09526600	2.60947900
H	4.98212100	-6.06480400	1.13639700
H	3.76672100	-1.92052300	1.10448600
H	4.40084500	-2.63686900	2.58932000
H	1.41755000	-4.84459800	3.25953400
H	3.01375900	-4.37948700	3.86381600
Pd	1.05514400	-1.79832100	-1.64463100
P	0.78262900	0.58097000	-1.04846700
C	2.17924700	1.27732400	0.08163700
C	0.61936300	1.68070000	-2.63032100
C	-0.89831800	0.61296400	-0.08336000
C	2.18548200	2.80950600	0.25042300

C	2.07248900	0.63233300	1.48021500
C	3.53628200	0.81426600	-0.50085000
C	2.02249900	1.98623800	-3.20171900
C	-0.10960000	0.85382100	-3.71931600
C	-0.11852500	3.01714600	-2.41546800
C	-2.05827200	0.36817600	-1.07509100
C	-0.93876700	-0.58753600	0.89758800
C	-1.17743100	1.90780900	0.70447100
H	2.38207100	3.33250100	-0.68757400
H	1.24786400	3.18964200	0.66268000
H	2.98789900	3.08411100	0.94779900
H	2.02726100	-0.45827200	1.42342000
H	2.97192800	0.89163400	2.05232100
H	1.21377400	0.99080900	2.05090000
H	4.33199000	1.10928800	0.19561500
H	3.57012800	-0.27184900	-0.62146700
H	3.76581000	1.25943400	-1.46810200
H	2.59662600	2.67179200	-2.57567000
H	2.60126100	1.07349400	-3.36088200
H	1.89279000	2.47456300	-4.17624700
H	-1.13624900	0.59805800	-3.45794700
H	-0.14787100	1.45366900	-4.63834400
H	0.42844800	-0.07214700	-3.94113500
H	-0.09788200	3.58374200	-3.35569400
H	-1.16825600	2.88175300	-2.14648500
H	0.35372700	3.63712900	-1.64937500
H	-2.23630200	1.21227700	-1.74292700
H	-1.89328000	-0.52964500	-1.68118700
H	-2.97922700	0.21184200	-0.49920800
H	-0.89826400	-1.54880200	0.37135500
H	-0.14736000	-0.57958300	1.64504300
H	-1.89581300	-0.56239400	1.43471600
H	-2.18158900	1.84676400	1.14470800
H	-0.47410600	2.05124900	1.52803300
H	-1.14922600	2.79843100	0.07380000

<u>Ar^{2}Br:</u>

Ar^{2}Br

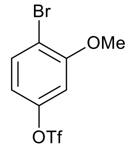

E_{disp} = -3881.64568
E_{nodisp} = -3881.619586
G_{corr} = 0.095506
H_{corr} = 0.159453

C	1.23887100	-6.24765100	-0.15466400
C	-1.17047300	-6.64455800	-0.10023500
C	1.48874400	-7.62183300	-0.12623400
C	-0.92125500	-8.02250700	-0.07533400
C	0.39511700	-8.47314700	-0.08665600
H	2.49731200	-8.01591800	-0.13195500
H	-1.72474300	-8.74557200	-0.04585200
O	-2.40468100	-6.09776500	-0.08341700
C	-3.53566700	-6.96798700	0.00925400
H	-3.59987200	-7.62798700	-0.86379400
H	-3.49703200	-7.56826000	0.92601100
H	-4.40644500	-6.31272400	0.03640100
O	0.59255800	-9.87321000	-0.11865100
S	0.99271700	-10.65222100	1.26676600
O	1.34450400	-11.99999000	0.85657900
O	1.85309100	-9.82737400	2.10081400
C	-0.68990000	-10.73493400	2.10230100
F	-0.98175200	-9.54517300	2.62184700
F	-1.61247400	-11.07188500	1.20205100
F	-0.63304300	-11.65191300	3.06145800
C	-0.06609700	-5.76656700	-0.14178700
Br	-0.36693200	-3.88278800	-0.17888500
H	2.06493200	-5.54663700	-0.18645100

$^{Ar2}TS_{OxAdd}$

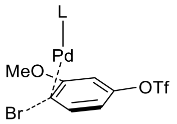

E_{disp} = -4824.800356
E_{nodisp} = -4824.701092
G_{corr} = 0.441812
H_{corr} =

Br	3.61113900	-2.11200100	-2.95356800
C	1.66480400	-3.09313200	-2.72348200
C	1.62497700	-4.13733100	-1.78311000
C	0.84569100	-3.13448100	-3.89097000
C	0.70870900	-5.18467300	-1.92650700
C	-0.06805900	-4.18087100	-4.02284800
C	-0.11098300	-5.17689200	-3.04385200
H	0.64687500	-5.98806500	-1.20220700
H	-0.74539600	-4.24471900	-4.86392900
O	1.00217000	-2.13810900	-4.79508400
C	0.15993600	-2.13316900	-5.94846300
H	0.30050100	-3.04186000	-6.54619100
H	-0.89501900	-2.03635200	-5.66477700
H	0.46359900	-1.26211300	-6.52936400
O	-1.08896400	-6.18869700	-3.20356900
S	-0.63734900	-7.63791900	-3.81863700
O	0.69618400	-8.00774800	-3.36883100
O	-1.79413800	-8.50061400	-3.65104300
C	-0.51208800	-7.21432200	-5.64623600
F	-1.54569000	-6.44804500	-5.99437300
F	0.62622100	-6.56363300	-5.87323100
F	-0.53539200	-8.34897400	-6.33743000
Pd	1.36888500	-1.35285300	-1.69028700
P	0.59551100	0.65123900	-0.66260900
C	-0.97810400	0.30069200	0.40952400
C	-2.15742000	-0.03858200	-0.52837100
C	-0.72574600	-0.98419100	1.23728700
C	-1.40945400	1.43684400	1.35711700
H	-2.52681600	0.82779800	-1.08003400
H	-1.89085500	-0.82497900	-1.24364500
H	-2.99083300	-0.41317800	0.07948600
H	-0.46104000	-1.82633400	0.58803200
H	0.05886800	-0.87491800	1.98512200
H	-1.65020600	-1.24487600	1.76948000
H	-2.34081000	1.14742500	1.86204000
H	-0.66909400	1.63052700	2.13675700
H	-1.60017800	2.37379400	0.82886600
C	1.93699900	1.49257900	0.45185700
C	1.68475900	2.97459300	0.79192600
C	2.06686700	0.69590800	1.76841200
C	3.31047500	1.35849900	-0.25073000
H	1.72152700	3.61514600	-0.09225300
H	0.72488400	3.13477800	1.28804800
H	2.47096100	3.32302500	1.47500500
H	2.21854700	-0.37274000	1.57996100
H	2.94558300	1.06358100	2.31342600
H	1.20611300	0.81837000	2.42845100
H	4.08529100	1.74864300	0.42252100
H	3.54502200	0.31105500	-0.46665900

H	3.38035600	1.91784200	-1.18294100
C	0.11775000	1.86792800	-2.09464200
C	1.40941100	2.42608600	-2.73143300
C	-0.57813800	1.05647700	-3.21753900
C	-0.78572200	3.04940400	-1.69098000
H	1.93351700	3.13234800	-2.08489600
H	2.09991200	1.62324600	-3.01140100
H	1.14054500	2.96696100	-3.64778300
H	-1.53493700	0.62969600	-2.91881200
H	-0.77127500	1.72938400	-4.06381700
H	0.06077400	0.24099200	-3.57670400
H	-0.93329600	3.70052800	-2.56300500
H	-1.77573400	2.72324800	-1.36360600
H	-0.34888300	3.65923300	-0.89689600
H	2.32011000	-4.13051100	-0.95086100

^{Ar2}Prod

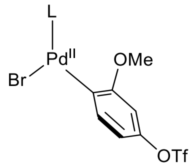

E_{disp} = -4824.828776
E_{nodisp} = -4824.72843
G_{corr} = 0.445524
H_{corr} = 0.555716

Br	2.87002700	-1.53493000	-3.52875600
C	0.93719700	-3.61601800	-2.40071800
C	1.70593300	-4.53013200	-1.68326100
C	0.00401500	-4.08152600	-3.35120400
C	1.52898500	-5.91296100	-1.84059700
C	-0.18332500	-5.45678200	-3.52938700
C	0.58770000	-6.33274200	-2.76611000
H	2.11169600	-6.63543400	-1.28135200
H	-0.89541900	-5.86292900	-4.23569700
O	-0.68618100	-3.13137500	-4.04592800
C	-1.58622700	-3.55818900	-5.06640500
H	-1.06756100	-4.14864000	-5.83214800
H	-2.41322000	-4.14672800	-4.64935500
H	-1.98092500	-2.64589400	-5.51527000
O	0.33532700	-7.72046500	-2.93675400
S	1.36147000	-8.60427400	-3.84950700
O	2.74113100	-8.18311300	-3.65698600
O	0.93011300	-9.98191600	-3.68320500
C	0.85193100	-8.05987500	-5.57481500
F	-0.47813400	-8.01101900	-5.64923000
F	1.36122500	-6.85759800	-5.83040900
F	1.32150100	-8.94846800	-6.44504700
Pd	0.97167500	-1.66854900	-1.93546100
P	0.75352900	0.66368700	-1.14267200
C	2.12340300	1.11278600	0.13699700
C	0.76009500	1.95486800	-2.57893500
C	-0.97618900	0.69490600	-0.26944200
C	2.19823500	2.60430400	0.51874600
C	1.90517300	0.28211200	1.42052400
C	3.48976100	0.65762400	-0.43081200
C	2.21248100	2.23485000	-3.02713400
C	0.05893500	1.31468800	-3.80283400
C	0.08864000	3.30088700	-2.24127500
C	-2.08170200	0.63853000	-1.34780700
C	-1.14315200	-0.60384900	0.56120600
C	-1.23177400	1.90295200	0.65232800
H	2.47858500	3.23605200	-0.32681500
H	1.25826500	2.98126600	0.92806900
H	2.96966800	2.73438500	1.28923400
H	1.80722000	-0.78706200	1.20048100
H	2.78282800	0.40596400	2.06713200
H	1.03596400	0.60679000	1.99579700
H	4.25413600	0.81821100	0.34078300
H	3.48183600	-0.40491200	-0.68942300
H	3.79876800	1.21167900	-1.31573400
H	2.78177400	2.80534500	-2.29077200
H	2.74874300	1.31184400	-3.26116700
H	2.17447500	2.84005100	-3.94208000
H	-0.98888900	1.07059800	-3.62936800
H	0.09359900	2.03058900	-4.63466400
H	0.57804800	0.40535400	-4.11953600
H	0.20545900	3.97680500	-3.09855800
H	-0.98287300	3.20256200	-2.05451500
H	0.54340400	3.78841000	-1.37516300
H	-2.16119600	1.55878800	-1.92843400
H	-1.93280800	-0.19996700	-2.03758000
H	-3.04711500	0.48725600	-0.84854900
H	-1.06745800	-1.50678700	-0.05844900
H	-0.43373900	-0.69822200	1.38095700
H	-2.15027500	-0.60765400	0.99805900
H	-2.26048200	1.84911600	1.03298600
H	-0.56790200	1.90728600	1.52020800
H	-1.12275500	2.85826900	0.13510000
H	2.45941400	-4.17289200	-0.98595300

Ar^{3}Br:

Ar^{3}Br

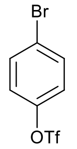

E_{disp} = -3767.071544
E_{nodisp} = -3767.050389
G_{corr} = 0.065838
H_{corr} = 0.123996

C	-1.49589500	-0.28934900	0.07241600
C	-0.10559600	-0.36977200	-0.00214700
C	0.64136300	0.80810400	-0.05883500
C	0.03041100	2.06336100	-0.04661900
C	-1.36042600	2.14443700	0.02224700
C	-2.09634900	0.96551200	0.08235400
H	-2.10758000	-1.18302500	0.12210400
H	0.38614000	-1.33537100	-0.01412600
H	0.62785300	2.96629800	-0.09332700
H	-1.86596200	3.10276300	0.03136000
Br	2.54804100	0.69928300	-0.15759500
O	-3.50856100	1.01877300	0.08657700
S	-4.29075000	1.40790000	1.47400100
O	-3.54193400	2.39590300	2.23555200
O	-5.67852800	1.59160500	1.08901700
C	-4.16193500	-0.22723000	2.39073700
F	-4.44748900	-1.22284600	1.55422100
F	-2.92263300	-0.36943700	2.85456000
F	-5.02920200	-0.21239100	3.39650400

^{Ar3}TS$_{OxAdd}$

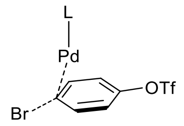

E_{disp}	=	-4710.227004	
E_{nodisp}	=	-4710.135114	
G_{corr}	=	0.413509	
H_{corr}	=	0.51799	

C	0.91436300	-3.10311400	-2.83790300
C	1.88009400	-2.87726900	-3.84151600
C	1.11589500	-4.07116300	-1.83153800
C	3.09466300	-3.55903800	-3.77847200
C	2.33247000	-4.75008800	-1.78217100
C	3.29474900	-4.48222100	-2.75388200
H	3.87182500	-3.39132000	-4.51540300
H	2.53496200	-5.48490600	-1.01062500
Pd	0.49255300	-1.33091000	-1.93398000
P	0.25593900	0.74416700	-0.77253900
C	-1.61549000	1.16119700	-0.50042300
C	-2.23605400	1.58499400	-1.84955300
C	-2.35210800	-0.14068700	-0.09766400
C	-1.90755400	2.25468000	0.54559600
H	-1.89976000	2.56812800	-2.18405800
H	-2.02815600	0.85264400	-2.63708900
H	-3.32544900	1.64111600	-1.72955600
H	-2.20796500	-0.92509000	-0.84849600
H	-2.04382400	-0.53526700	0.86986700
H	-3.42727700	0.07340800	-0.03449900
H	-2.98820400	2.44909100	0.56667000
H	-1.61651800	1.95320000	1.55452500
H	-1.40889500	3.19901000	0.31584200
C	1.12990000	0.64174800	0.95180300
C	1.41108300	1.99262300	1.63873400
C	0.27460600	-0.22760600	1.89963400
C	2.46307200	-0.12763000	0.77688900
H	2.12713500	2.60119200	1.08164200
H	0.50505300	2.58455400	1.78762400
H	1.84870400	1.80577600	2.62849200
H	0.02223000	-1.19211800	1.44529200
H	0.85727100	-0.43158400	2.80698600
H	-0.64738800	0.26446500	2.21429500
H	2.91986400	-0.26548700	1.76590400
H	2.29184100	-1.12009800	0.34425700
H	3.18983300	0.39158300	0.15330000
C	1.07057200	2.16544300	-1.80430300
C	2.60674700	2.02469000	-1.72863200
C	0.70828500	1.95460300	-3.29582900
C	0.68339200	3.59796600	-1.38785800
H	3.01129200	2.27959000	-0.74739400
H	2.93311800	1.01262600	-1.99279800
H	3.05650000	2.71650900	-2.45211900
H	-0.35332900	2.07428000	-3.50850100
H	1.24796700	2.69978500	-3.89519200
H	1.01286400	0.95965100	-3.63974900
H	1.25115400	4.31297800	-1.99814100
H	-0.37625300	3.80625700	-1.55287100
H	0.91201500	3.80691100	-0.34034600
H	0.33573500	-4.28307400	-1.10903900
H	1.68112300	-2.17874500	-4.64658200
Br	-1.18860800	-2.78619300	-3.44747400
O	4.53993700	-5.14792800	-2.64550400
S	4.85910400	-6.40693300	-3.64297700
O	4.31748300	-6.17332400	-4.97349400
O	6.25393800	-6.73689400	-3.40696100
C	3.81354300	-7.74766400	-2.84186700
F	3.96772700	-7.69554200	-1.51982800
F	2.53478800	-7.55282700	-3.15455700
F	4.22236300	-8.92555800	-3.30153800

^{Ar3}Prod

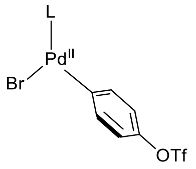

E_{disp}	=	-4710.251959	
E_{nodisp}	=	-4710.158578	
G_{corr}	=	0.414364	
H_{corr}	=	0.519938	

C	0.83200300	-3.80304200	-2.17822400
C	1.48364100	-4.62404700	-1.24820500
C	-0.02299000	-4.36175000	-3.13691800
C	1.22788700	-5.99858700	-1.22410100
C	-0.28126300	-5.73498300	-3.12571400
C	0.34848000	-6.52212800	-2.16609500
H	1.70532500	-6.65397400	-0.50423000
H	-0.94724700	-6.19495700	-3.84817800
Pd	0.87930800	-1.83565700	-1.86787600
P	0.67548200	0.54159500	-1.18841800
C	2.15950200	1.04314000	-0.06233200
C	0.55214200	1.76855200	-2.67425600
C	-0.96441900	0.62614000	-0.16118200
C	2.27462900	2.55029000	0.23998700
C	2.06149100	0.27383500	1.27332200
C	3.46729700	0.55426400	-0.73237700
C	1.95639000	2.00952100	-3.27316800
C	-0.27065900	1.08396500	-3.79377800
C	-0.07184900	3.13722500	-2.33576600
C	-2.17099500	0.55081500	-1.12332800
C	-1.06485000	-0.64409000	0.72255300
C	-1.11647200	1.86924900	0.73712900
H	2.47335700	3.13949900	-0.65786800
H	1.37975500	2.95289700	0.71965200
H	3.11731800	2.71119300	0.92531000
H	1.95570000	-0.80526200	1.11290800
H	2.99089800	0.43266600	1.83442800
H	1.24190500	0.61926800	1.90640900
H	4.30115600	0.75278500	-0.04630500
H	3.43795800	-0.52106100	-0.93094500
H	3.69035500	1.05912900	-1.67097300
H	2.59868600	2.60618000	-2.62258400
H	2.46033800	1.07028900	-3.51454100
H	1.83496900	2.57271400	-4.20747700
H	-1.30224100	0.87378900	-3.51222500
H	-0.30076400	1.75624400	-4.66141900
H	0.20091500	0.14858000	-4.10897900
H	-0.03510200	3.77121100	-3.23142600
H	-1.12034100	3.06200100	-2.03960000
H	0.47026800	3.65862700	-1.54293000
H	-2.30122500	1.45487100	-1.72012600
H	-2.09719800	-0.30869100	-1.79894400
H	-3.08308200	0.42326300	-0.52659600
H	-1.10878100	-1.56116600	0.12041300
H	-0.25484300	-0.74944700	1.44194300
H	-2.00322100	-0.59775200	1.29065000
H	-2.10725100	1.84826900	1.21018400
H	-0.37699500	1.89141700	1.54116800
H	-1.03949300	2.80381300	0.17762100

H	-0.49433700	-3.73708200	-3.89033200
H	2.18194200	-4.20337300	-0.52860000
Br	2.50931500	-1.78419200	-3.74175700
O	0.02605800	-7.90472100	-2.13505800
S	1.09875500	-8.98610400	-2.72561400
O	2.46688000	-8.59918500	-2.41407000
O	0.55444400	-10.28953100	-2.38527300
F	-0.44726800	-8.67675600	-4.84046600
F	1.44278500	-7.59786800	-4.94085800
C	0.85586400	-8.73353700	-4.57073300
F	1.40791800	-9.76137200	-5.20788700

<u>PhBr:</u>

PhBr

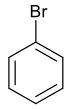

E_{disp} = -2805.92724
E_{nodisp} = -2805.919621
G_{corr} = 0.060007
H_{corr} = 0.097522

C	-1.45854600	-0.34366500	0.00005700
C	-0.06120000	-0.35248100	0.00055400
C	0.61847900	0.86549000	0.00002300
C	-0.06110300	2.08354400	-0.00100000
C	-1.45842600	2.07485200	-0.00149600
C	-2.15849800	0.86561500	-0.00096300
H	-1.99600600	-1.28764100	0.00047200
H	0.48836300	-1.28733500	0.00134900
H	0.48857600	3.01832900	-0.00140000
H	-1.99583000	3.01886000	-0.00229000
H	-3.24446800	0.86568000	-0.00134600
Br	2.53912500	0.86543200	0.00070700

$^{PhBr}TS_{OxAdd}$

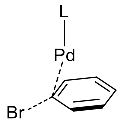

E_{disp} = -3749.080775
E_{nodisp} = -3749.002646
G_{corr} = 0.407533
H_{corr} = 0.491513

C	0.91591600	-3.25453200	-2.53003900
C	1.58102500	-3.02535300	-3.74980100
C	1.47769200	-4.06460400	-1.52481600
C	2.86961300	-3.54131400	-3.91332600
C	2.76780800	-4.56680100	-1.71718000
C	3.46160500	-4.31018900	-2.90498700
H	3.40516700	-3.34981400	-4.83949900
H	3.22435000	-5.17150000	-0.93797200
Pd	0.44337200	-1.44750000	-1.70522300
P	0.22618300	0.70233800	-0.70000700
C	-1.63151800	1.24470300	-0.59170800
C	-2.12937800	1.60852800	-2.00760200
C	-2.47574000	0.02033700	-0.15916400
C	-1.92775300	2.42353800	0.35601600
H	-1.71111700	2.54577300	-2.37943000
H	-1.91105600	0.81308400	-2.72855900
H	-3.21917500	1.73338500	-1.97310600
H	-2.33542500	-0.82148400	-0.84509800
H	-2.25750100	-0.32514800	0.85080500
H	-3.53641300	0.30403900	-0.18369200
H	-2.99272900	2.68174000	0.28279700
H	-1.72976900	2.17542800	1.40140500
H	-1.35533600	3.31938000	0.10506900
C	0.96896000	0.67695800	1.08953300
C	1.27604100	2.05636000	1.70482800
C	0.00031500	-0.07903200	2.02532000
C	2.26726500	-0.16792500	1.06899600
H	2.06536300	2.58519000	1.16546000
H	0.39755800	2.70419900	1.74303900
H	1.62806900	1.91729900	2.73582900
H	-0.27398500	-1.05770300	1.61629300
H	0.50424300	-0.25042700	2.98513400
H	-0.91206000	0.48120100	2.23783700
H	2.64161100	-0.25856700	2.09743100
H	2.07584700	-1.17802400	0.68983900
H	3.06511400	0.27093000	0.47099600
C	1.19268000	2.00802900	-1.75584000
C	2.70877900	1.78889300	-1.55858600
C	0.92413200	1.71703600	-3.25361500
C	0.86418100	3.48483000	-1.46207400
H	3.05814200	2.08821000	-0.56870300
H	2.99116800	0.74438400	-1.73166400
H	3.24886700	2.40339000	-2.29016700
H	-0.10910700	1.88905600	-3.55288600
H	1.55478900	2.38242300	-3.85815600
H	1.18298200	0.68267200	-3.50693000
H	1.51384800	4.12406400	-2.07488800
H	-0.16743500	3.73920600	-1.71609100
H	1.03281400	3.75249300	-0.41656200
H	0.91650800	-4.28977200	-0.62418300
H	4.45721100	-4.71920200	-3.04977300
H	1.09848400	-2.46000200	-4.53997600
Br	-1.30854400	-3.26680300	-2.63767700

$^{PhBr}Prod$

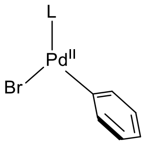

E_{disp} = -3749.104624
E_{nodisp} = -3749.025302
G_{corr} = 0.408348
H_{corr} = 0.493456

C	0.93524500	-3.70532100	-2.29803700
C	1.75706000	-4.53229500	-1.52389100
C	-0.06705500	-4.25274200	-3.10658700
C	1.51785600	-5.91187000	-1.49842000
C	-0.29902100	-5.63358900	-3.07529500
C	0.49045700	-6.46153300	-2.27136600
H	2.14195000	-6.55522000	-0.88267300
H	-1.08974800	-6.06058100	-3.68761600
Pd	0.94352400	-1.74846100	-1.94925800
P	0.68707800	0.60415100	-1.18198400
C	2.11061400	1.06061600	0.03866200
C	0.63940200	1.89327500	-2.61996400
C	-1.00224500	0.65271900	-0.23639400
C	2.21354200	2.55449100	0.40403900
C	1.94135100	0.24310700	1.33809900
C	3.45042000	0.59057600	-0.57978100
C	2.07356600	2.14819800	-3.13653600
C	-0.12766900	1.26176100	-3.80813900
C	0.00336400	3.25010500	-2.25806800
C	-2.15509100	0.60939300	-1.26422200
C	-1.14272000	-0.64863000	0.59434100

C	-1.20878800	1.86165500	0.69667900
H	2.46426300	3.17631300	-0.45809200
H	1.29384100	2.94300400	0.84737600
H	3.01657600	2.68567500	1.14149500
H	1.83322400	-0.82803900	1.13261200
H	2.84352500	0.37120900	1.94940400
H	1.09512300	0.57404000	1.94328200
H	4.24845600	0.75806200	0.15551700
H	3.42704100	-0.47676400	-0.81967800
H	3.72419100	1.12894400	-1.48585100
H	2.68579700	2.71106900	-2.42929800
H	2.58242600	1.21503600	-3.39200100
H	2.00406600	2.75113900	-4.05121400
H	-1.17384000	1.04903900	-3.58962900
H	-0.10836500	1.96977000	-4.64734100
H	0.35395000	0.33654500	-4.13721500
H	0.08842100	3.92095600	-3.12321400
H	-1.05956100	3.16680900	-2.02076700
H	0.50392000	3.73534500	-1.41648800
H	-2.25493300	1.53393300	-1.83508900
H	-2.04151600	-0.22552700	-1.96480700
H	-3.09831300	0.46033100	-0.72345700
H	-1.09961200	-1.54515800	-0.03763100
H	-0.39676900	-0.75352600	1.38006300
H	-2.12865400	-0.64828400	1.07737700
H	-2.22017900	1.81596000	1.12222600
H	-0.50738500	1.86198700	1.53444400
H	-1.11517300	2.81597700	0.17420800
H	-0.67203100	-3.61601200	-3.74822700
H	2.57007800	-4.11233500	-0.93580000
H	0.31473100	-7.53387900	-2.25810200
Br	2.59045400	-1.63619300	-3.81493700

4.3. Functional Benchmarking Single Point Energies

Method	Basis	Solvent	$(PtBu_3)_2Pd$	$PtBu_3$	Ar^1Br	$^{Ar1}TS_{OxAdd}$	Ar^2Br	$^{Ar2}TS_{OxAdd}$
**B3LYPD3**	6-31G*/LANL2DZ	CPCM (THF)	-1756.687308	-814.9161874	-4267.932681	-5209.665828	-3878.385713	-4820.115492
**B3LYPD3**	Def2TZVP	CPCM (THF)	-1758.330911	-815.1325289	-4271.325542	-5214.481957	-3881.64568	-4824.800356
**B3LYP**	Def2TZVP	CPCM (THF)	-1757.521457	-814.7522429	-4270.94822	-5213.673411	-3881.52408	-4824.25928
**B3LYPD3**	Def2QZVP	CPCM (THF)	-1758.440196	-815.1861121	-4271.476236	-5214.688809	-3881.768379	-4824.979439
**B3LYPD3**	Def2TZVP	SMD(THF)	-1758.340214	-815.1387338	-4271.337368	-5214.497124	-3881.652657	-4824.811043
**B3LYPD3**	Def2TZVP	PCM(THF)	-1758.3295	-815.1322124	-4271.325002	-5214.480927	-3881.645105	-4824.799328
**B3LYPD3**	Def2TZVP	GAS PHASE	-1758.326359	-815.1299547	-4271.318277	-5214.470902	-3881.637786	-4824.788307
**B3LYPD2**	Def2TZVP	CPCM (THF)	-1758.400781	-815.1633572	-4271.350544	-5214.545568	-3881.648897	-4824.8396
**B3LYPD3(BJ)**	Def2TZVP	CPCM (THF)	-1758.412988	-815.1691069	-4271.380195	-5214.582534	-3881.671731	-4824.871681
**B3LYPD4***	Def2TZVP	CPCM (THF)	-1757.492207	-814.7479776	-4270.488909	-5213.194439	-3881.053899	-5638.524612
**B3LYP***	Def2TZVP	CPCM (THF)	-1757.298473	-814.6681905	-4270.367919	-5212.956499	-3880.998432	-5638.247529
**M06**	Def2TZVP	CPCM (THF)	-1757.375215	-814.664269	-4270.3653	-5213.029618	-3881.01572	-4823.678393
**M06L**	Def2TZVP	CPCM (THF)	-1758.082739	-814.9891395	-4270.953676	-5214.006002	-3881.358537	-4824.40851
**M06D3**	Def2TZVP	CPCM (THF)	-1757.397967	-814.6719429	-4270.3762	-5213.0574	-3881.019616	-4823.695268
**M06LD3**	Def2TZVP	CPCM (THF)	-1758.092848	-814.9921473	-4270.957963	-5214.018482	-3881.359995	-4824.415874
**B97D**	Def2TZVP	CPCM (THF)	-1757.822521	-814.749979	-4271.735256	-5214.767349	-3882.369378	-4825.395761
**wB97xD**	Def2TZVP	CPCM (THF)	-1757.928752	-814.9363163	-4270.905681	-5213.850769	-3881.356374	-4824.298478
**PBE0**	Def2TZVP	CPCM (THF)	-1756.658359	-814.332153	-4269.381259	-5211.661999	-3880.194658	-4822.481006
**PBE0D3**	Def2TZVP	CPCM (THF)	-1756.73926	-814.3621928	-4269.421735	-5211.755332	-3880.210663	-4822.542827
**PBE**	Def2TZVP	CPCM (THF)	-1756.44439	-814.2050765	-4269.195162	-5211.393839	-3880.080522	-4822.284546
**PBED3**	Def2TZVP	CPCM (THF)	-1756.520612	-814.233467	-4269.23353	-5211.482274	-3880.095625	-4822.342752
**DLPNO**	Def2TZVPP	CPCM(THF) from DFT	-1754.9	-813.6	-4266.831022	-5208.139379	-3878.017882	-4819.325261
**DLPNO**	Def2TZVPP	GAS PHASE	-1754.915731	-813.5648896	-4266.831022	-5208.139379	-3878.017882	-4819.325261

. *These calculations were carried out in ORCA program

5. NMR spectra

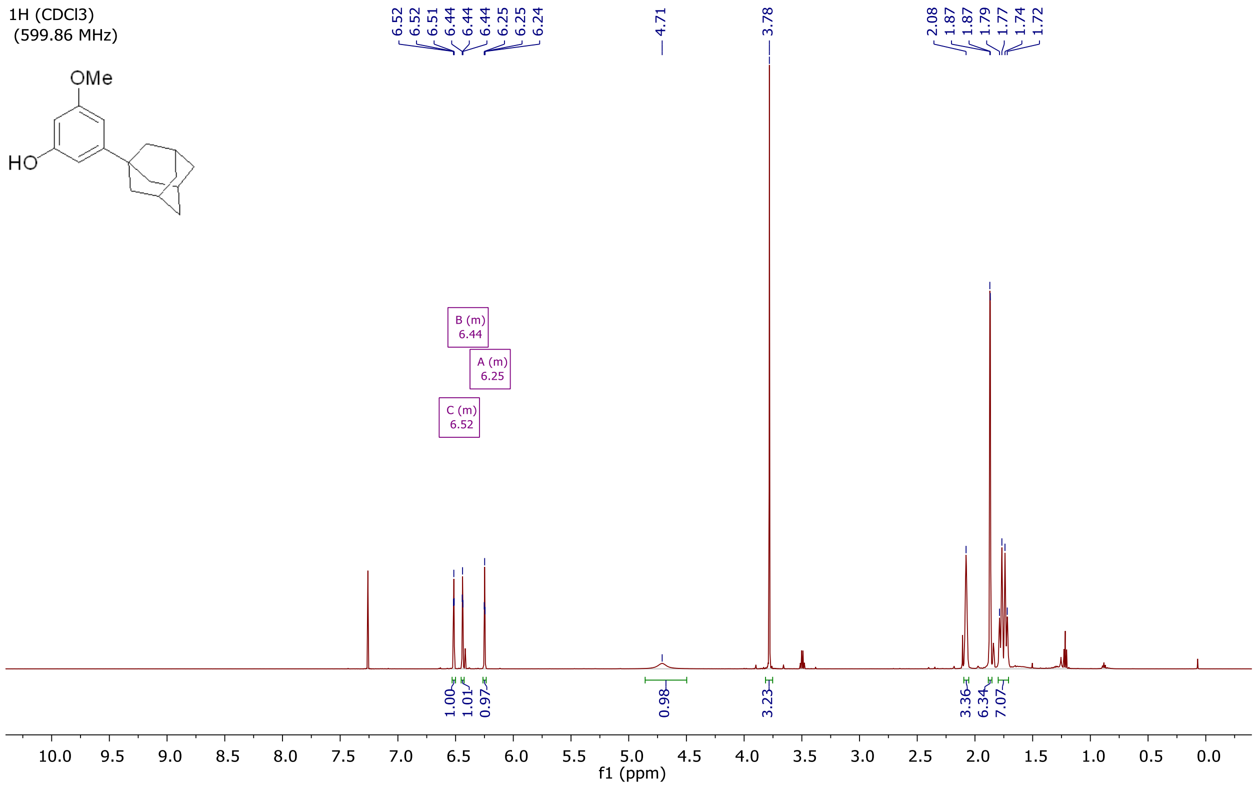

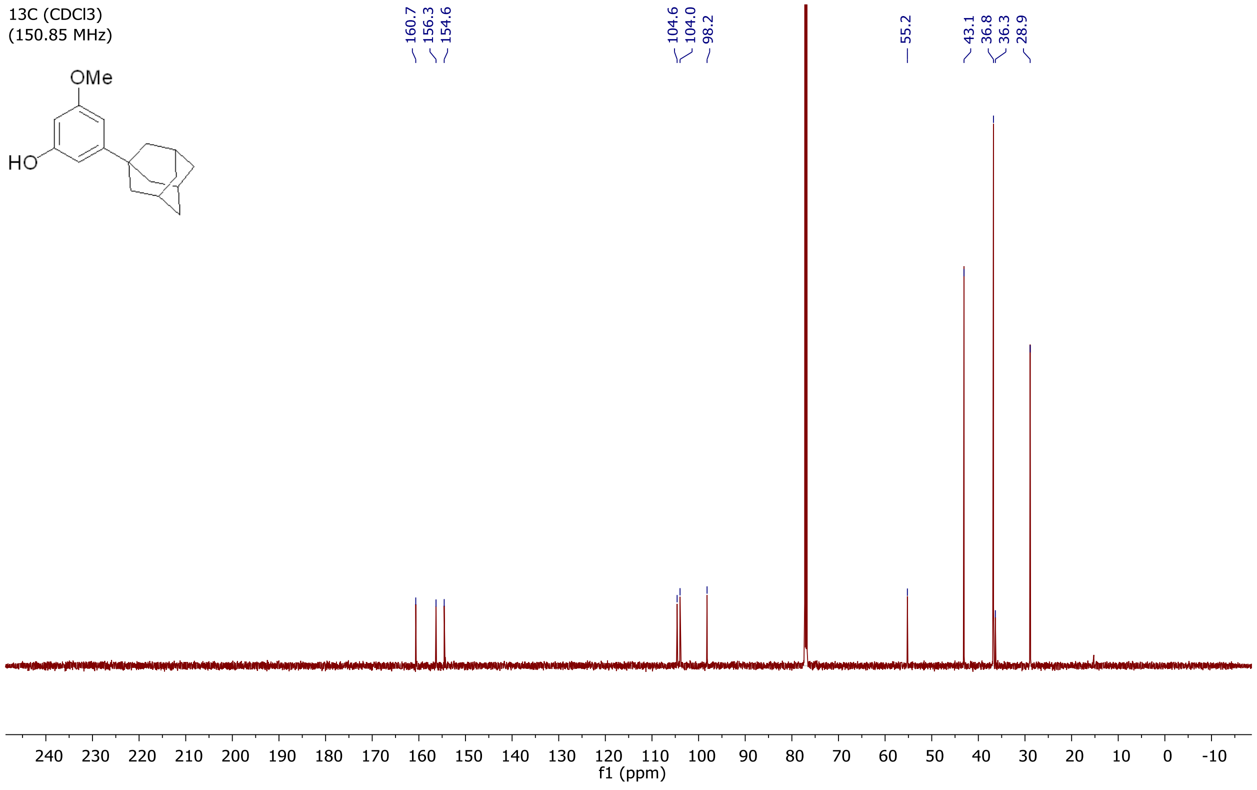

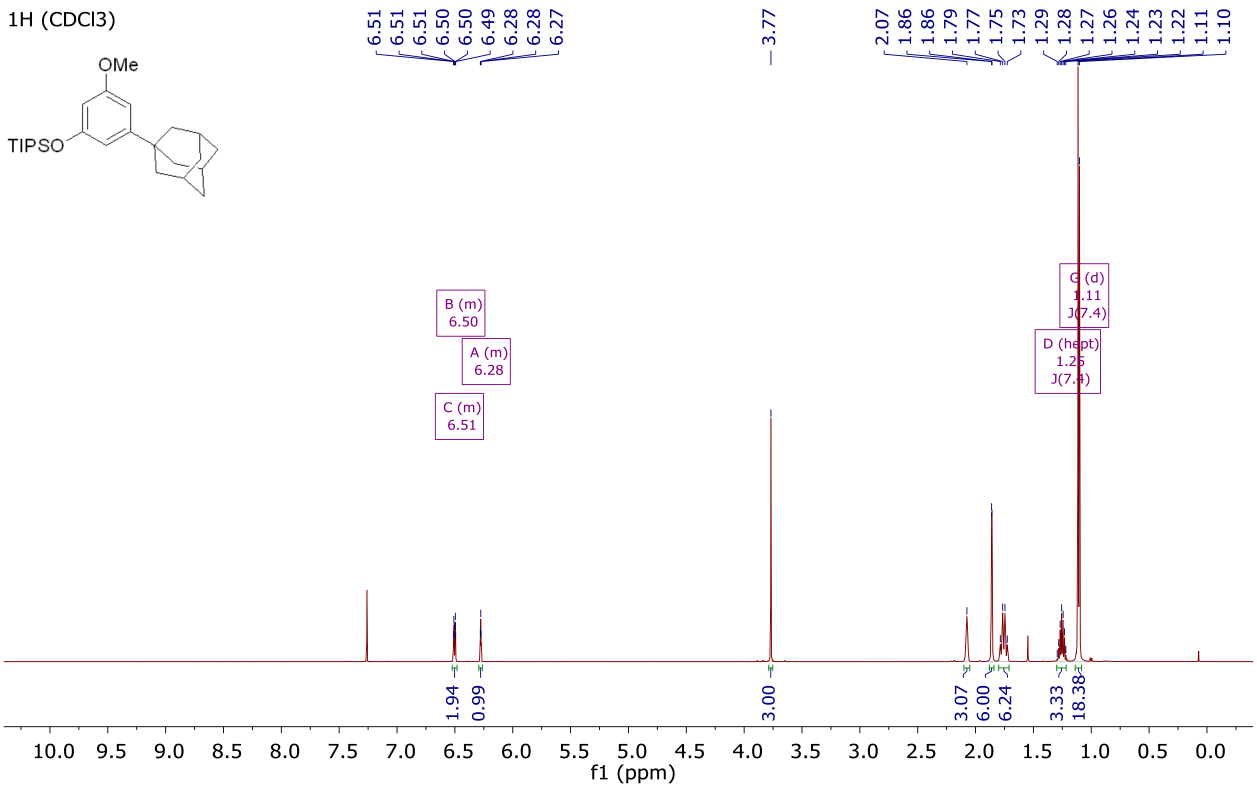
1H (CDCl3)
OMe
TIPSO
6.51 6.51 6.51 6.50 6.50 6.49 6.28 6.28 6.27
3.77
2.07 1.86 1.86 1.79 1.77 1.75 1.73 1.29 1.28 1.27 1.26 1.24 1.23 1.22 1.11 1.10
B (m) 6.50
A (m) 6.28
C (m) 6.51
G (d) 1.11 J(7.4)
D (hept) 1.25 J(7.4)
1.94 0.99 3.00 3.07 6.00 6.24 3.33 18.38
10.0 9.5 9.0 8.5 8.0 7.5 7.0 6.5 6.0 5.5 5.0 4.5 4.0 3.5 3.0 2.5 2.0 1.5 1.0 0.5 0.0
f1 (ppm)

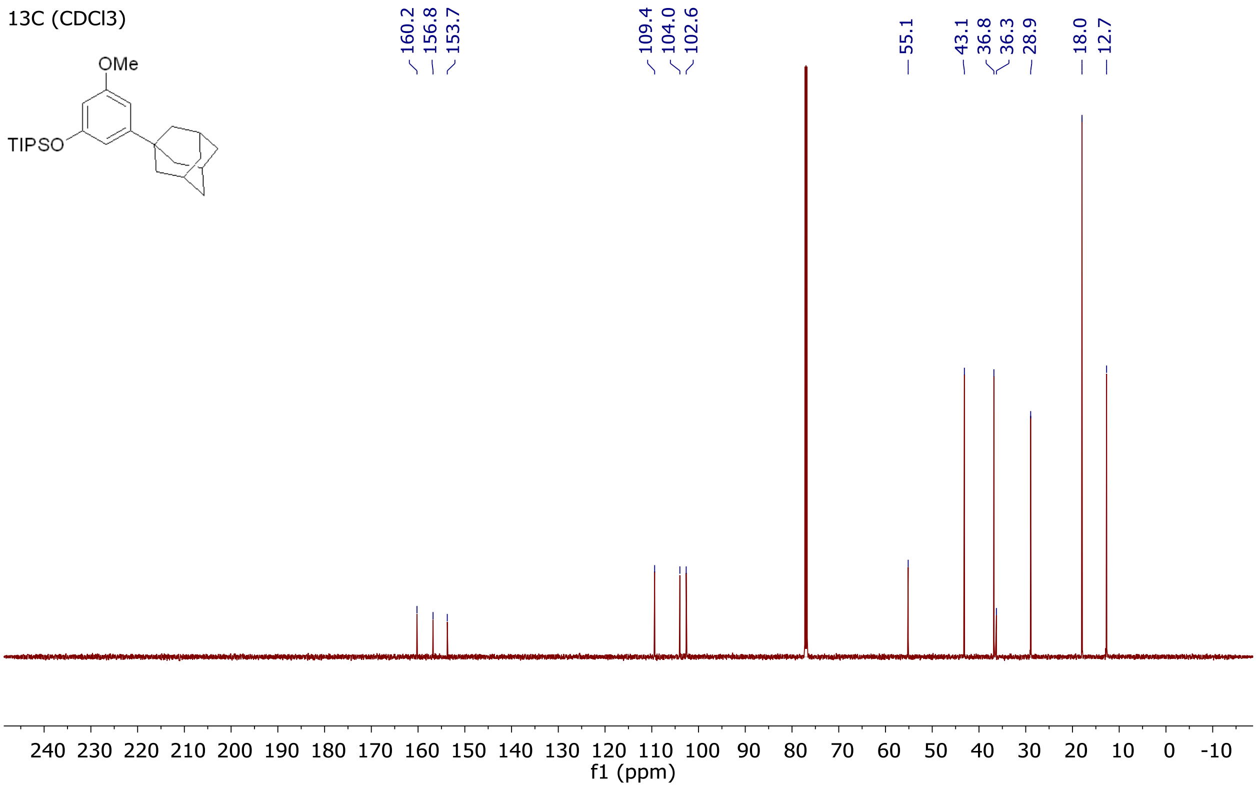
13C (CDCl3)
OMe
TIPSO
160.2 156.8 153.7
109.4 104.0 102.6
55.1
43.1 36.8 36.3 28.9
18.0 12.7
240 230 220 210 200 190 180 170 160 150 140 130 120 110 100 90 80 70 60 50 40 30 20 10 0 -10
f1 (ppm)

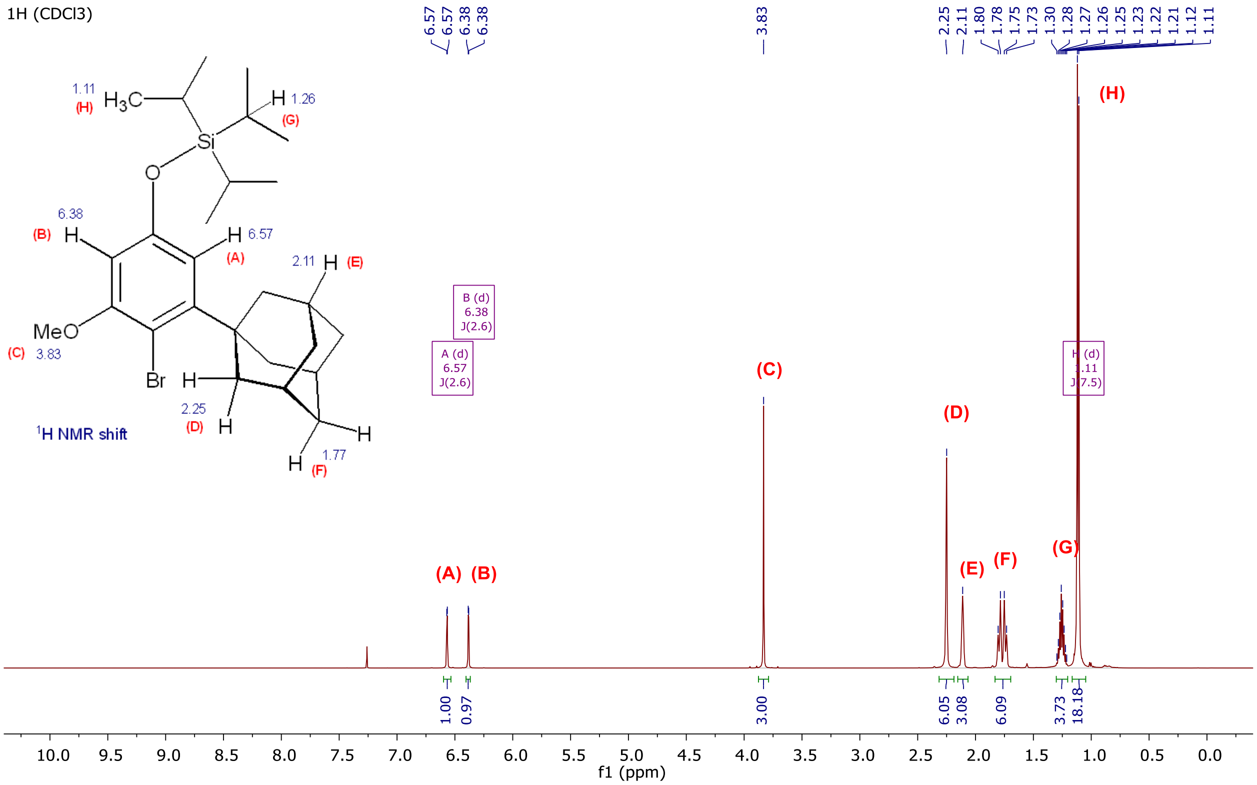
1H (CDCl3)
1.11 (H)
1.26 (G)
6.38 (B)
6.57 (A)
2.11 (E)
MeO
(C) 3.83
Br
2.25 (D)
1.77 (F)
¹H NMR shift
(A) (B) (C) (D) (E) (F) (G) (H)
A (d) 6.57 J(2.6)
B (d) 6.38 J(2.6)
1.00 0.97 3.00 6.05 3.08 6.09 3.73 18.18
f1 (ppm)

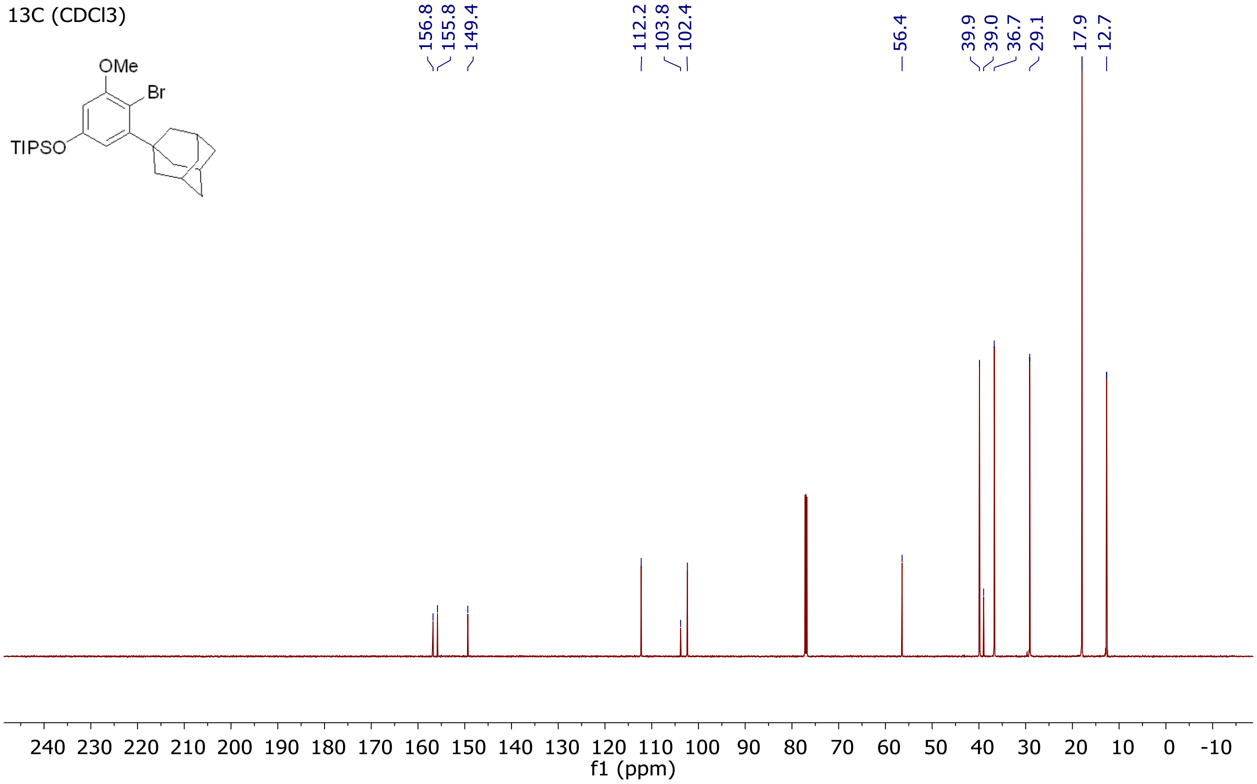
13C (CDCl3)
OMe
Br
TIPSO
156.8 155.8 149.4 112.2 103.8 102.4 56.4 39.9 39.0 36.7 29.1 17.9 12.7
f1 (ppm)

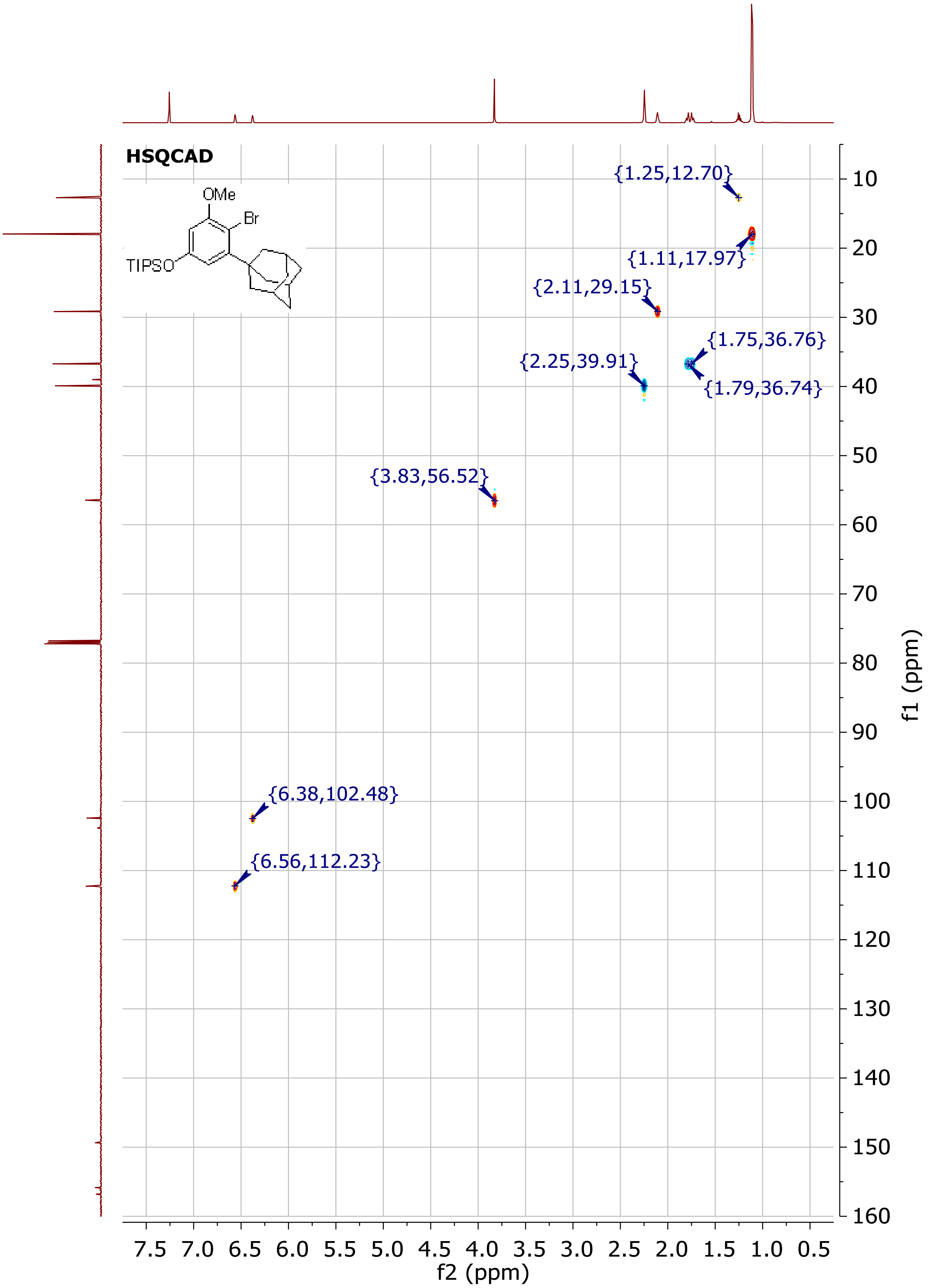
HSQCAD
OMe
Br
TIPSO
{1.25,12.70}
{1.11,17.97}
{2.11,29.15}
{1.75,36.76}
{2.25,39.91}
{1.79,36.74}
{3.83,56.52}
{6.38,102.48}
{6.56,112.23}
f1 (ppm)
f2 (ppm)

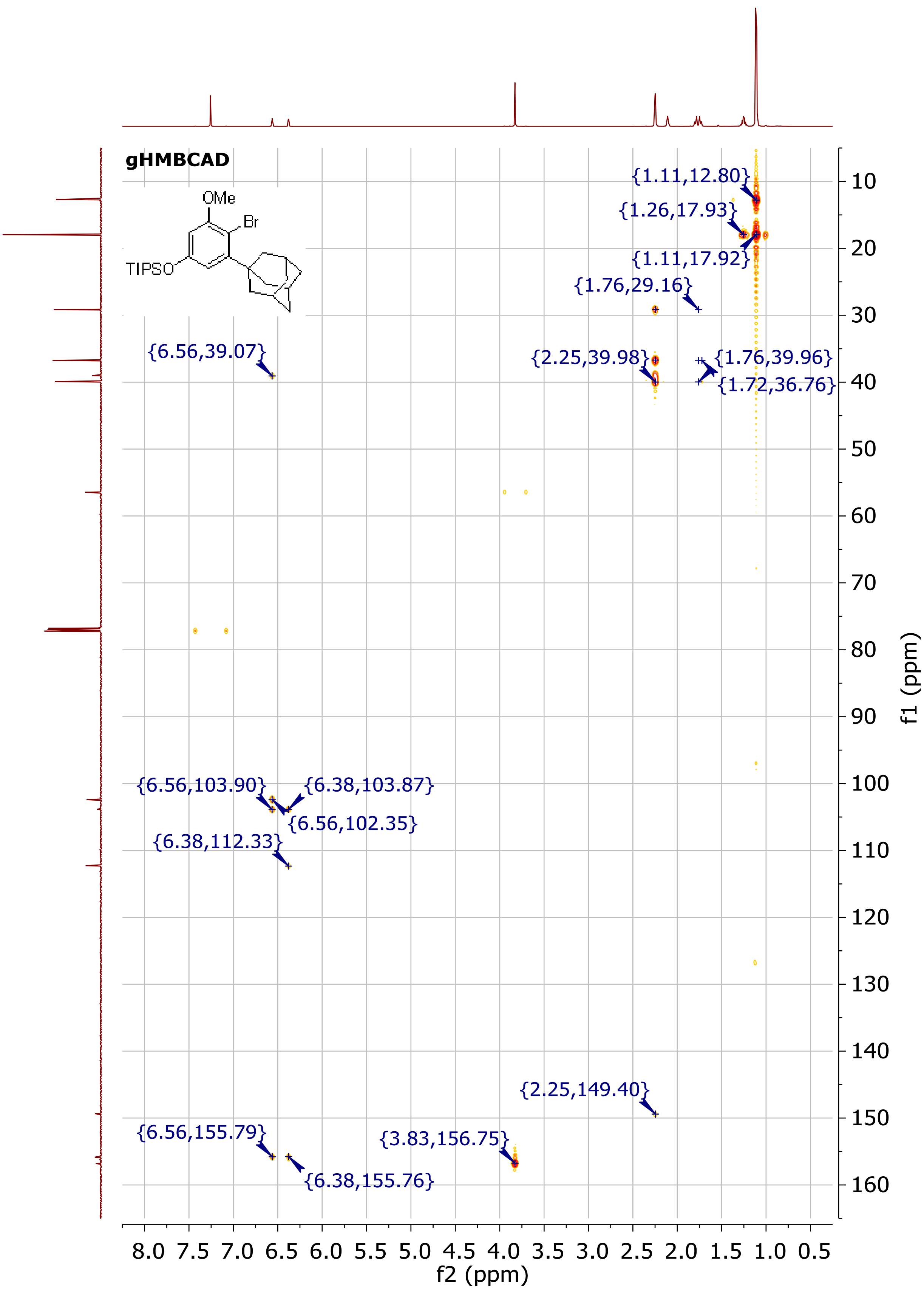
gHMBCAD
OMe
Br
TIPSO
{1.11,12.80}
{1.26,17.93}
{1.11,17.92}
{1.76,29.16}
{6.56,39.07}
{2.25,39.98}
{1.76,39.96}
{1.72,36.76}
{6.56,103.90}
{6.38,103.87}
{6.56,102.35}
{6.38,112.33}
{2.25,149.40}
{6.56,155.79}
{3.83,156.75}
{6.38,155.76}
f2 (ppm)
f1 (ppm)

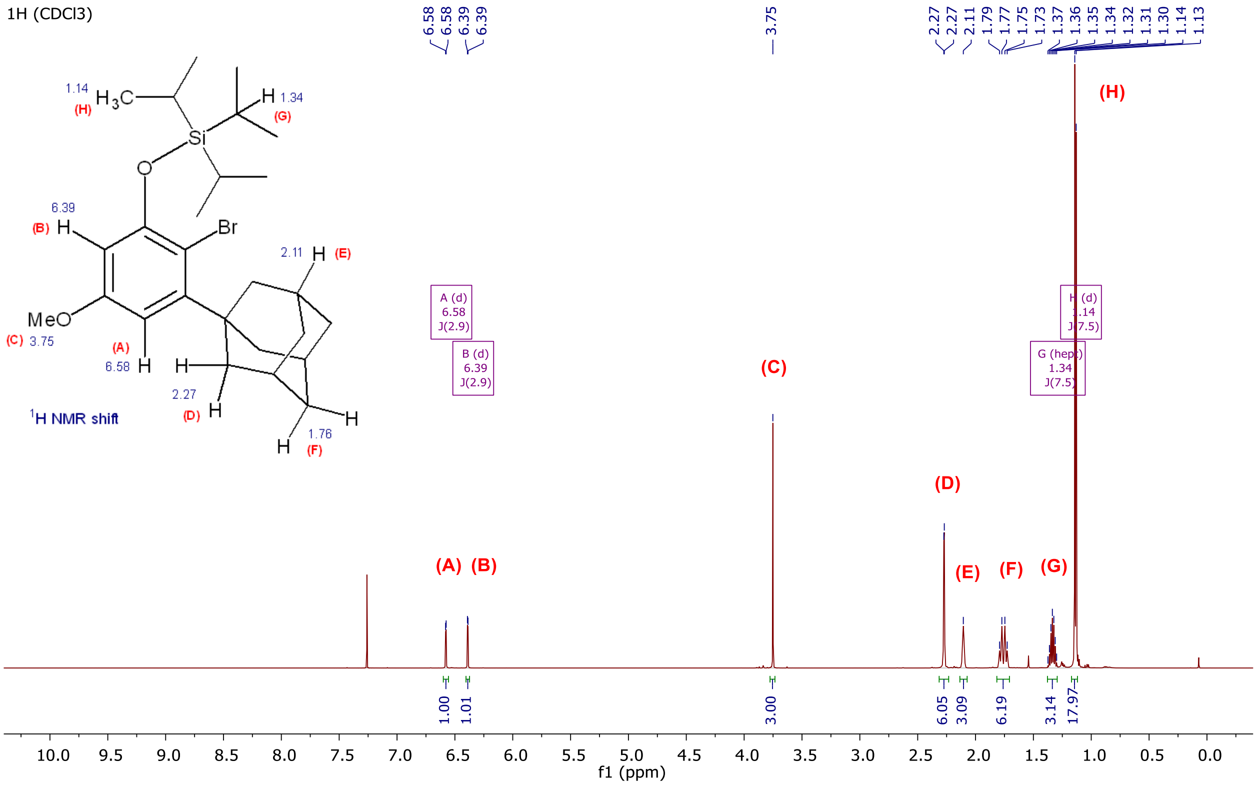
1H (CDCl3)
6.58
6.58
6.39
6.39
3.75
2.27
2.27
2.11
1.79
1.77
1.75
1.73
1.37
1.36
1.35
1.34
1.32
1.31
1.30
1.14
1.13
^{1}H NMR shift
A (d) 6.58 J(2.9)
B (d) 6.39 J(2.9)
(A) (B) (C) (D) (E) (F) (G) (H)
1.00
1.01
3.00
6.05
3.09
6.19
3.14
17.97
f1 (ppm)

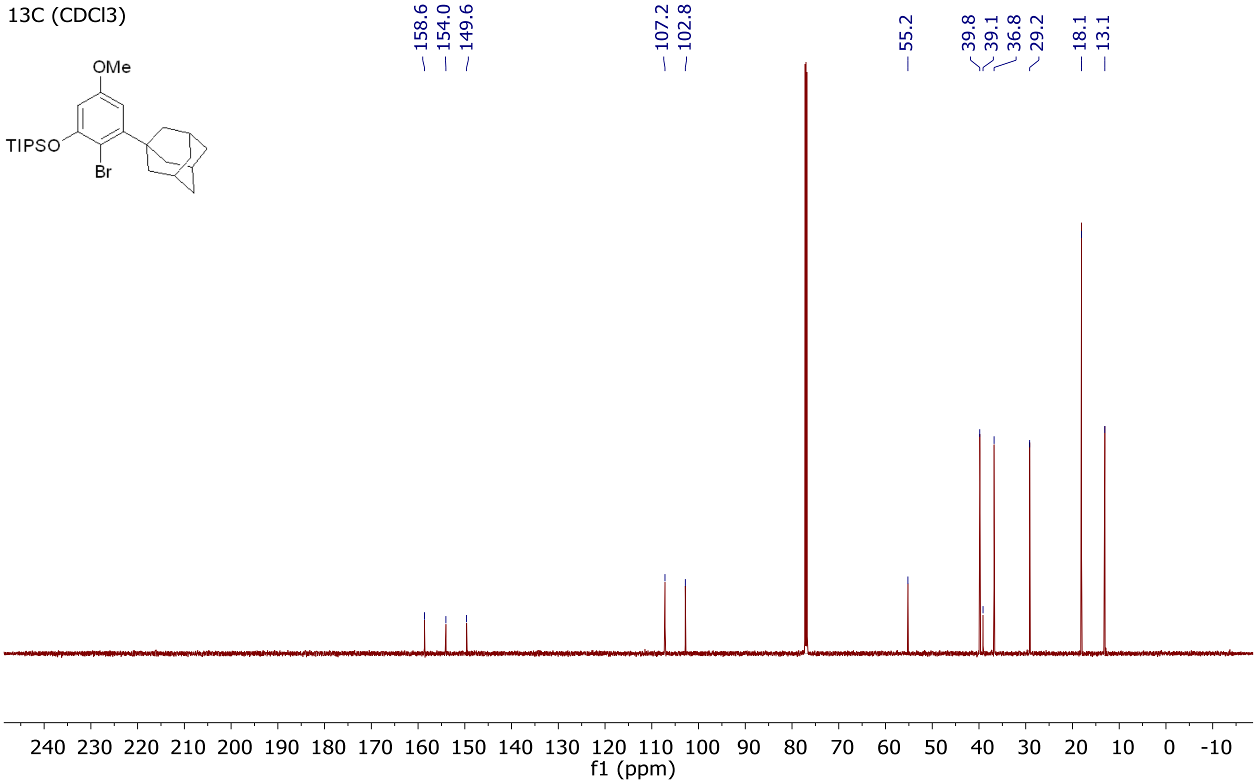
13C (CDCl3)
158.6
154.0
149.6
107.2
102.8
55.2
39.8
39.1
36.8
29.2
18.1
13.1
OMe
TIPSO
Br
f1 (ppm)

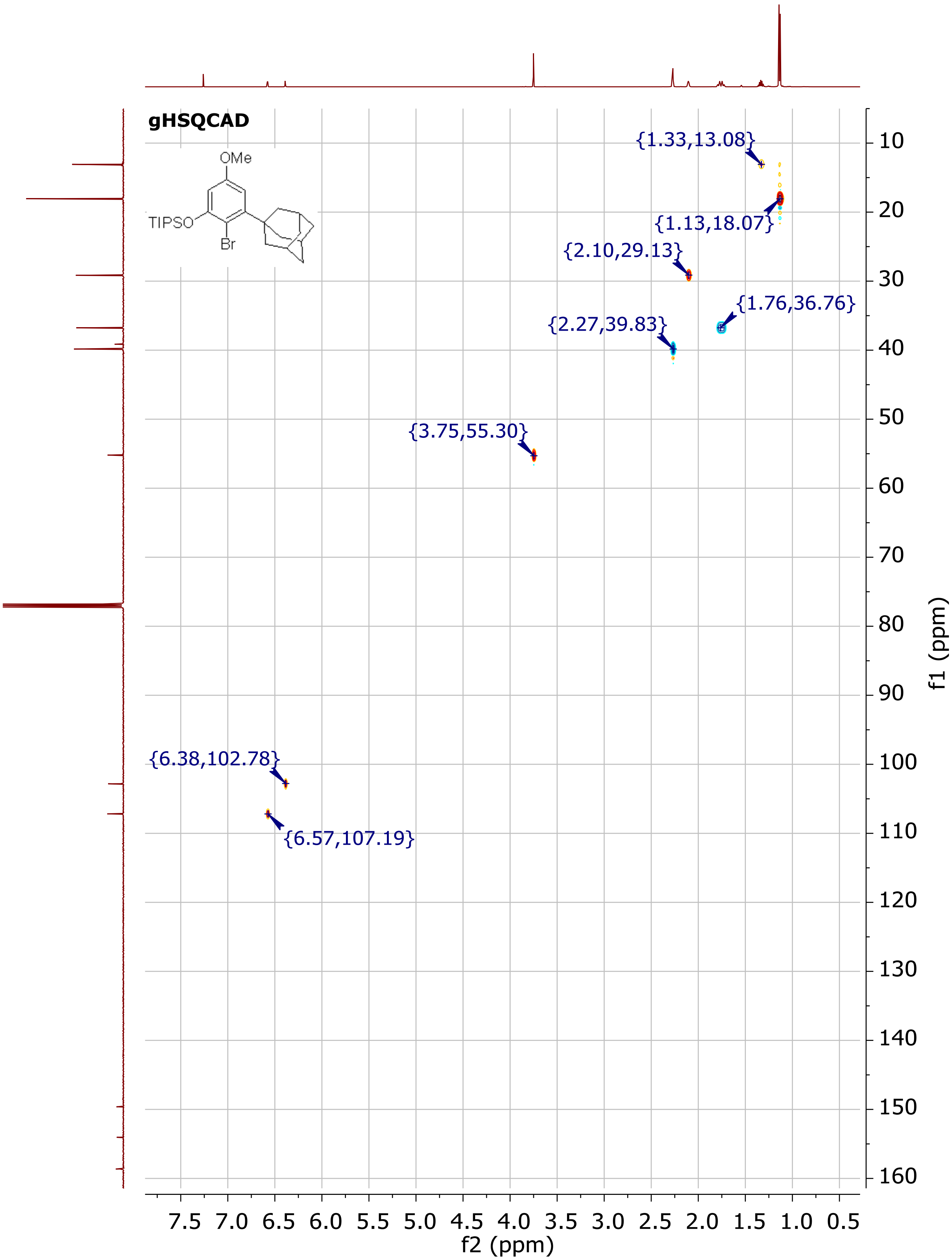
gHSQCAD
OMe
TIPSO
Br
{1.33,13.08}
{1.13,18.07}
{2.10,29.13}
{1.76,36.76}
{2.27,39.83}
{3.75,55.30}
{6.38,102.78}
{6.57,107.19}
f1 (ppm)
f2 (ppm)

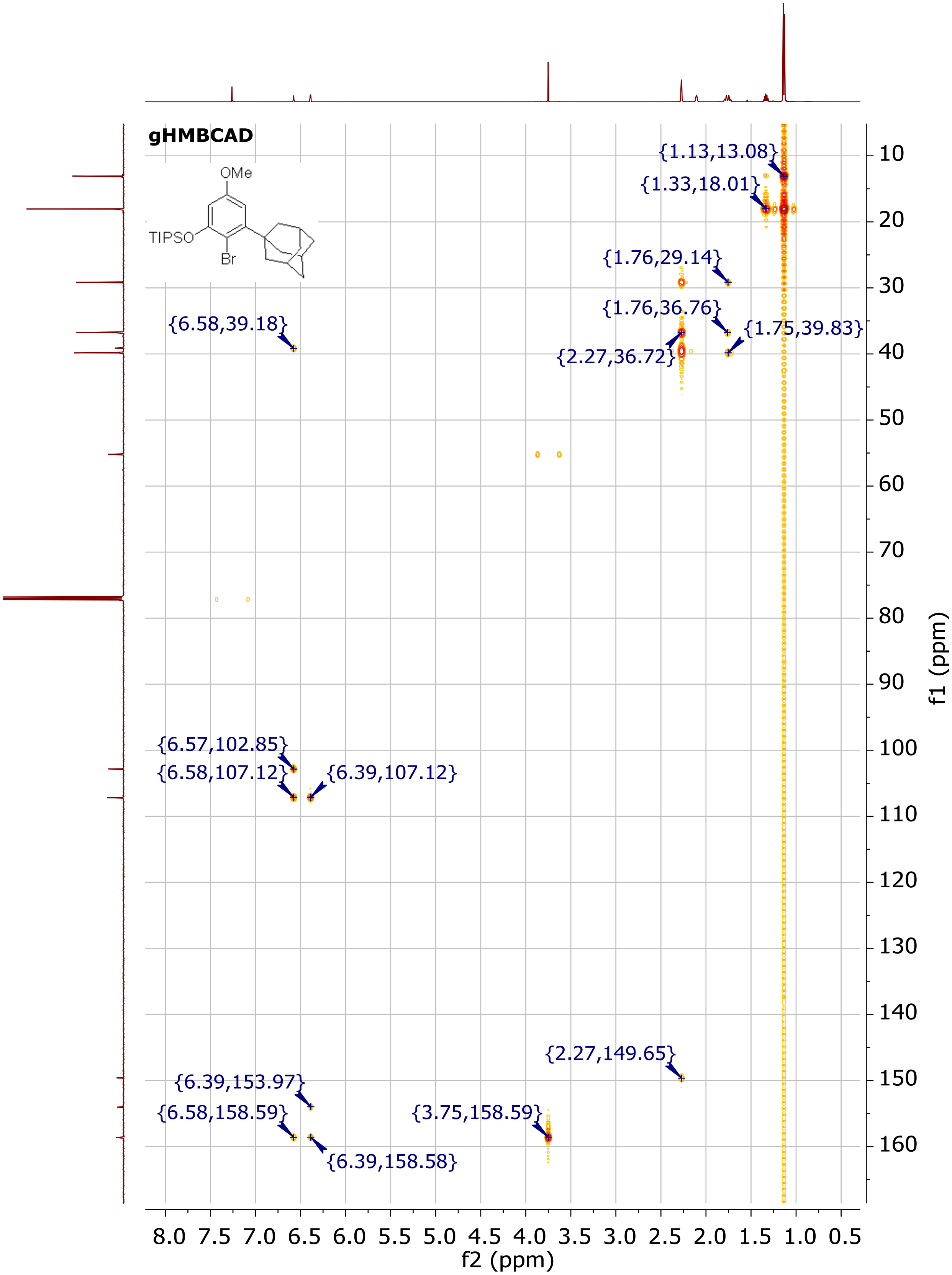
gHMBCAD
OMe
TIPSO
Br
{1.13,13.08}
{1.33,18.01}
{1.76,29.14}
{1.76,36.76}
{1.75,39.83}
{2.27,36.72}
{6.58,39.18}
{6.57,102.85}
{6.58,107.12}
{6.39,107.12}
{2.27,149.65}
{6.39,153.97}
{6.58,158.59}
{3.75,158.59}
{6.39,158.58}
f2 (ppm)
f1 (ppm)

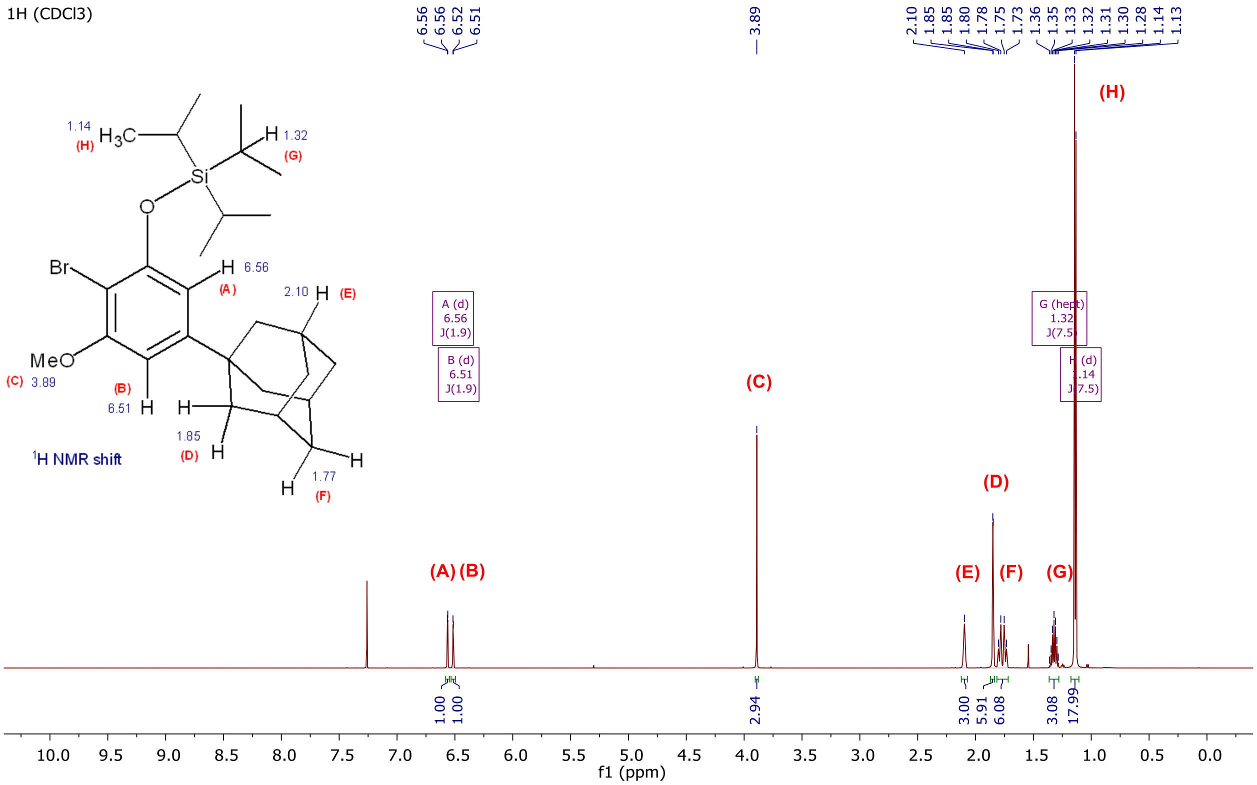
1H (CDCl3)
6.56
6.56
6.52
6.51
3.89
2.10
1.85
1.85
1.80
1.78
1.75
1.73
1.36
1.35
1.33
1.32
1.31
1.30
1.28
1.14
1.13
1.14 (H) H_3C
H 1.32 (G)
Si
O
Br
H 6.56 (A)
2.10 H (E)
MeO
(C) 3.89
(B) 6.51 H
H
1.85 (D) H
H
H 1.77 (F)
^{1}H NMR shift
A (d) 6.56 J(1.9)
B (d) 6.51 J(1.9)
(C)
G (hept) 1.32 J(7.5)
H (d) 1.14 J(7.5)
(H)
(D)
(A) (B)
(E) (F) (G)
1.00
1.00
2.94
3.00
5.91
6.08
3.08
17.99
10.0 9.5 9.0 8.5 8.0 7.5 7.0 6.5 6.0 5.5 5.0 4.5 4.0 3.5 3.0 2.5 2.0 1.5 1.0 0.5 0.0
f1 (ppm)

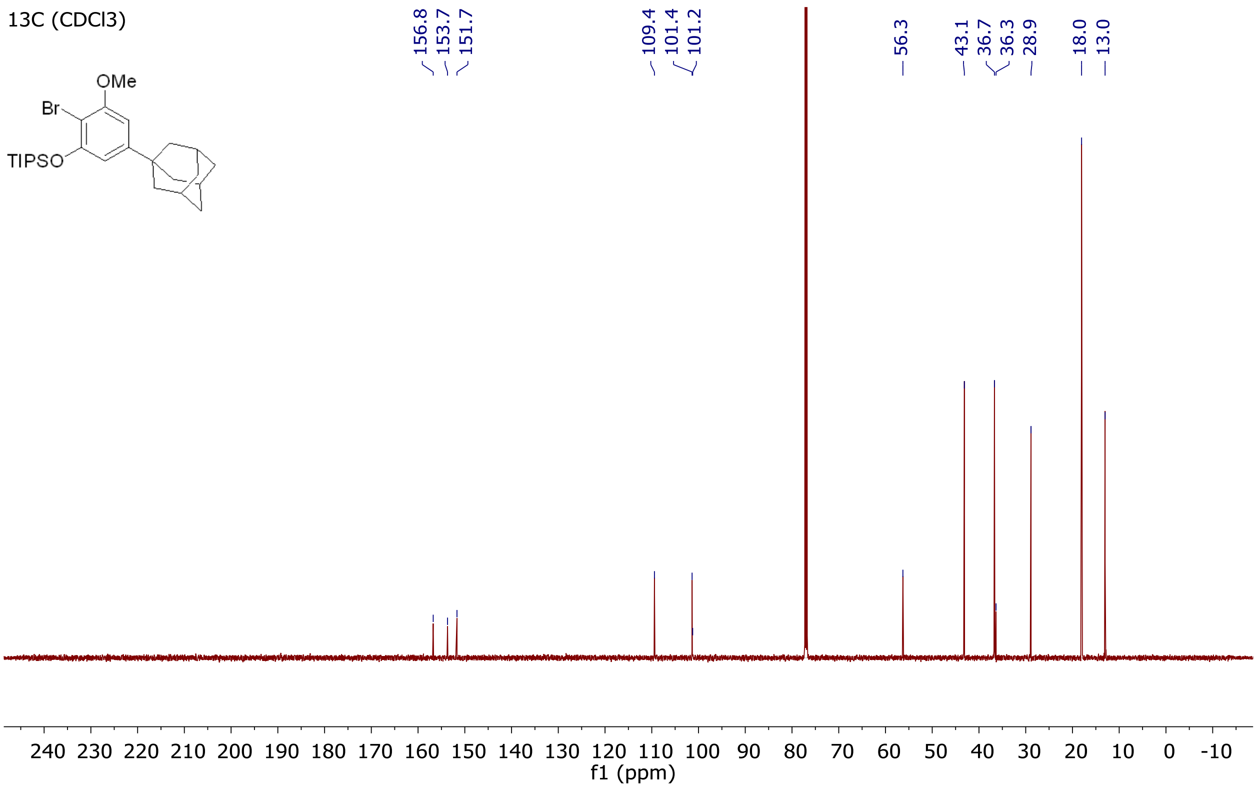
13C (CDCl3)
156.8
153.7
151.7
109.4
101.4
101.2
56.3
43.1
36.7
36.3
28.9
18.0
13.0
OMe
Br
TIPSO
240 230 220 210 200 190 180 170 160 150 140 130 120 110 100 90 80 70 60 50 40 30 20 10 0 -10
f1 (ppm)

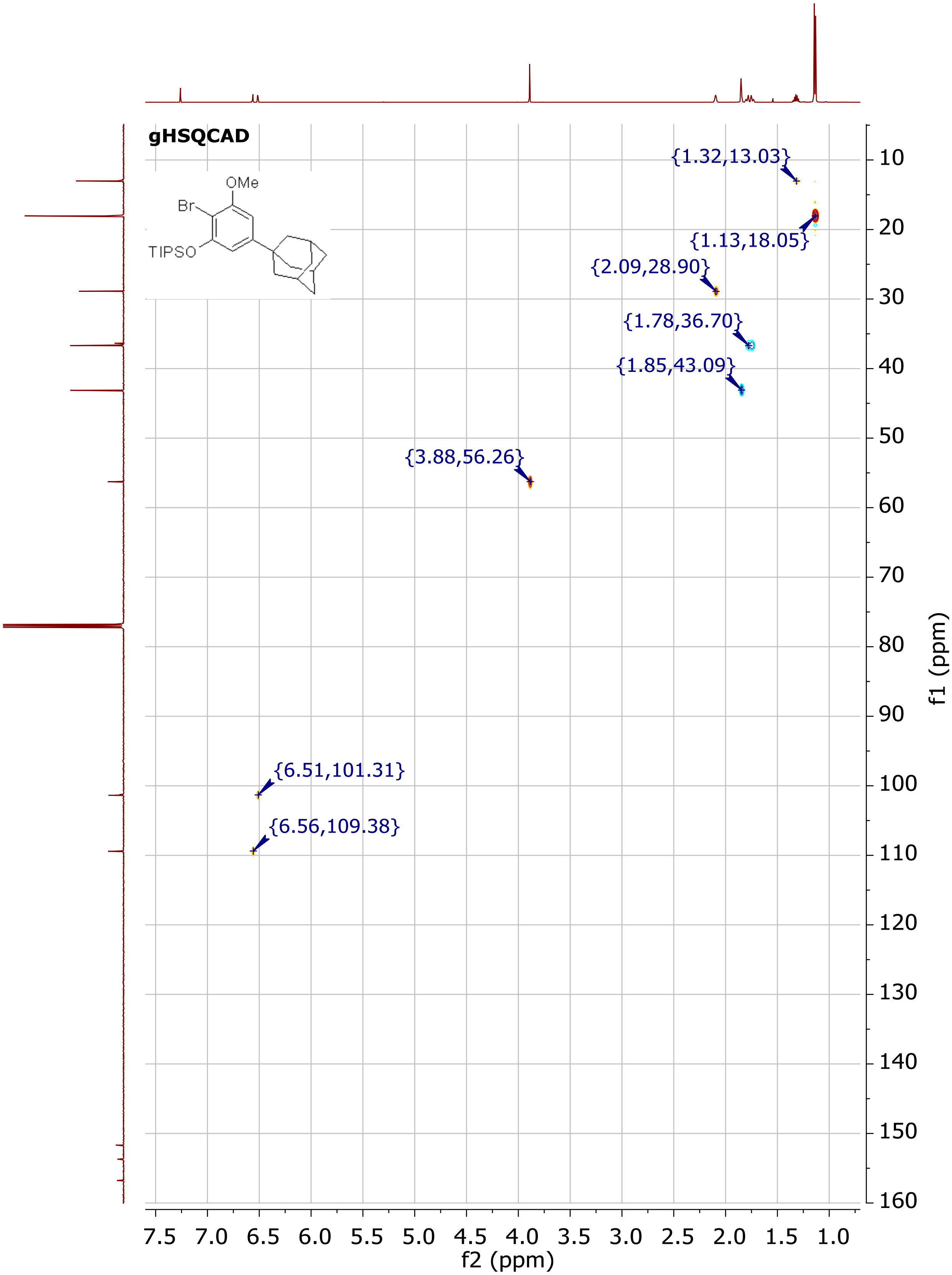
gHSQCAD
OMe
Br
TIPSO
{1.32,13.03}
{1.13,18.05}
{2.09,28.90}
{1.78,36.70}
{1.85,43.09}
{3.88,56.26}
{6.51,101.31}
{6.56,109.38}
f2 (ppm)
f1 (ppm)

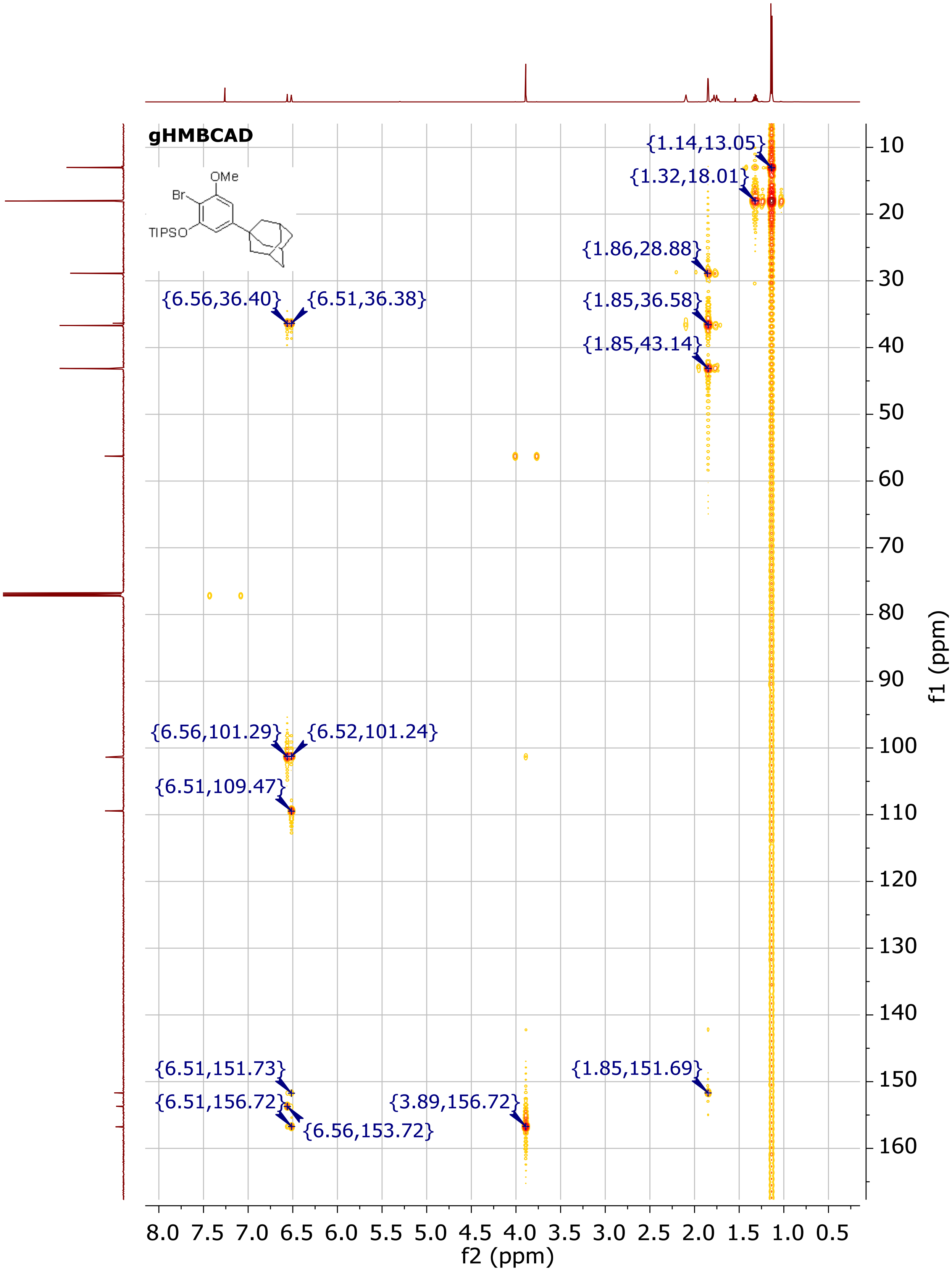
gHMBCAD
OMe
Br
TIPSO
{1.14,13.05}
{1.32,18.01}
{1.86,28.88}
{6.56,36.40}
{6.51,36.38}
{1.85,36.58}
{1.85,43.14}
{6.56,101.29}
{6.52,101.24}
{6.51,109.47}
{6.51,151.73}
{1.85,151.69}
{6.51,156.72}
{3.89,156.72}
{6.56,153.72}
f2 (ppm)
f1 (ppm)

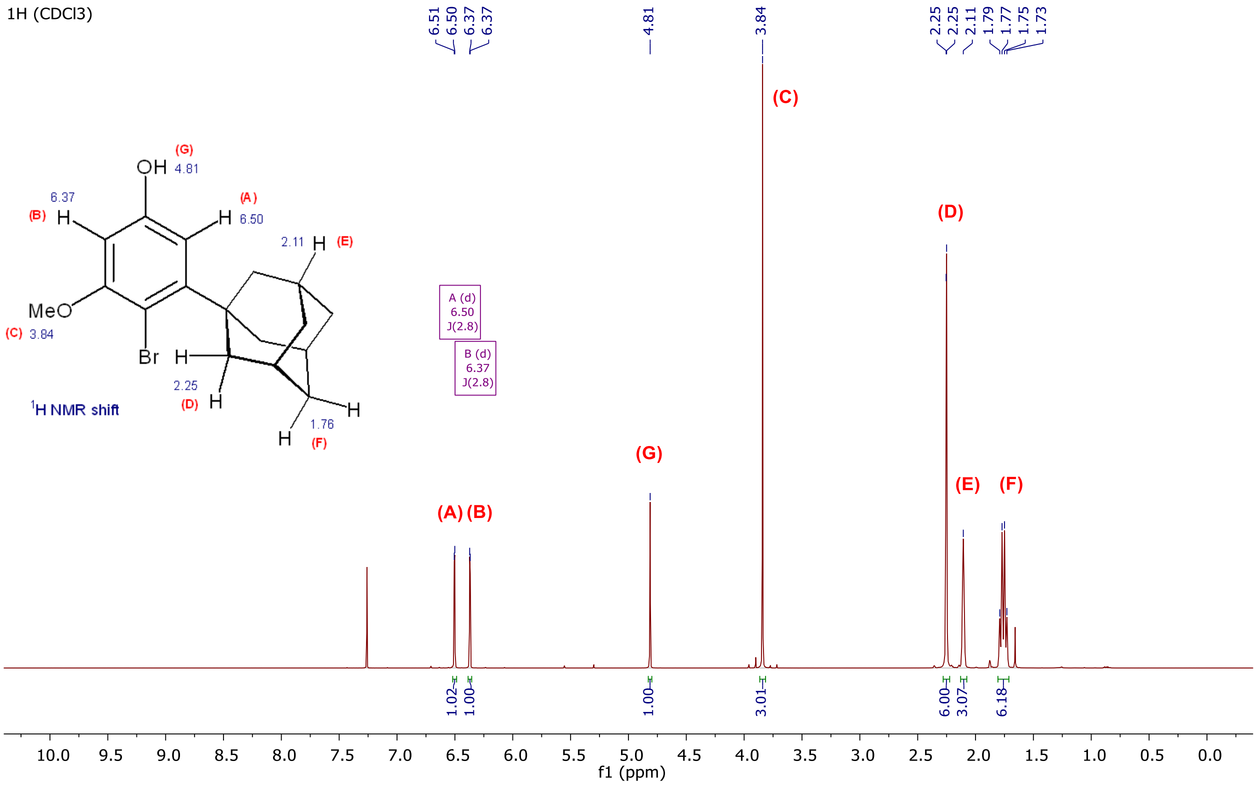
1H (CDCl3)
6.51
6.50
6.37
6.37
4.81
3.84
2.25
2.25
2.11
1.79
1.77
1.75
1.73
OH (G) 4.81
6.37 (B) H
(A) H 6.50
2.11 H (E)
MeO
(C) 3.84
Br H
2.25 (D) H
1.76 H (F)
1H NMR shift
A (d) 6.50 J(2.8)
B (d) 6.37 J(2.8)
(C)
(D)
(G)
(E) (F)
(A) (B)
1.02
1.00
1.00
3.01
6.00
3.07
6.18
10.0 9.5 9.0 8.5 8.0 7.5 7.0 6.5 6.0 5.5 5.0 4.5 4.0 3.5 3.0 2.5 2.0 1.5 1.0 0.5 0.0
f1 (ppm)

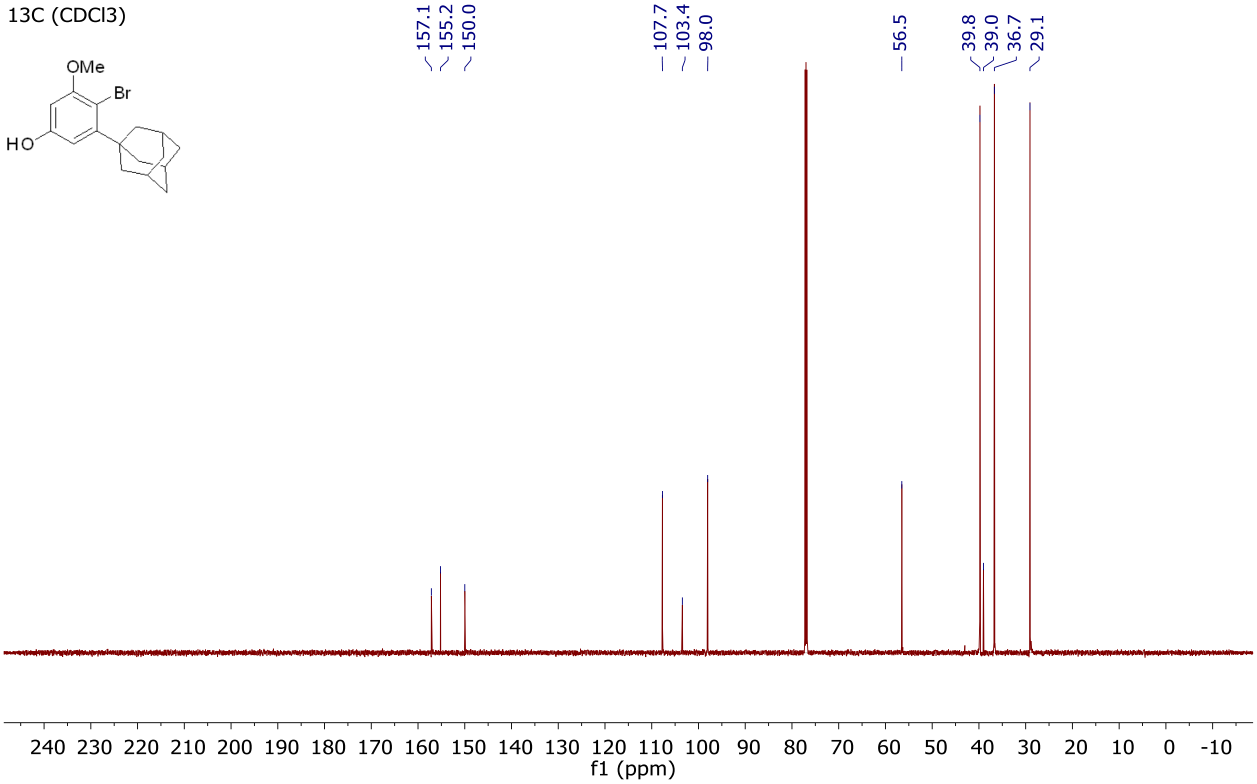
13C (CDCl3)
157.1
155.2
150.0
107.7
103.4
98.0
56.5
39.8
39.0
36.7
29.1
OMe
Br
HO
240 230 220 210 200 190 180 170 160 150 140 130 120 110 100 90 80 70 60 50 40 30 20 10 0 -10
f1 (ppm)

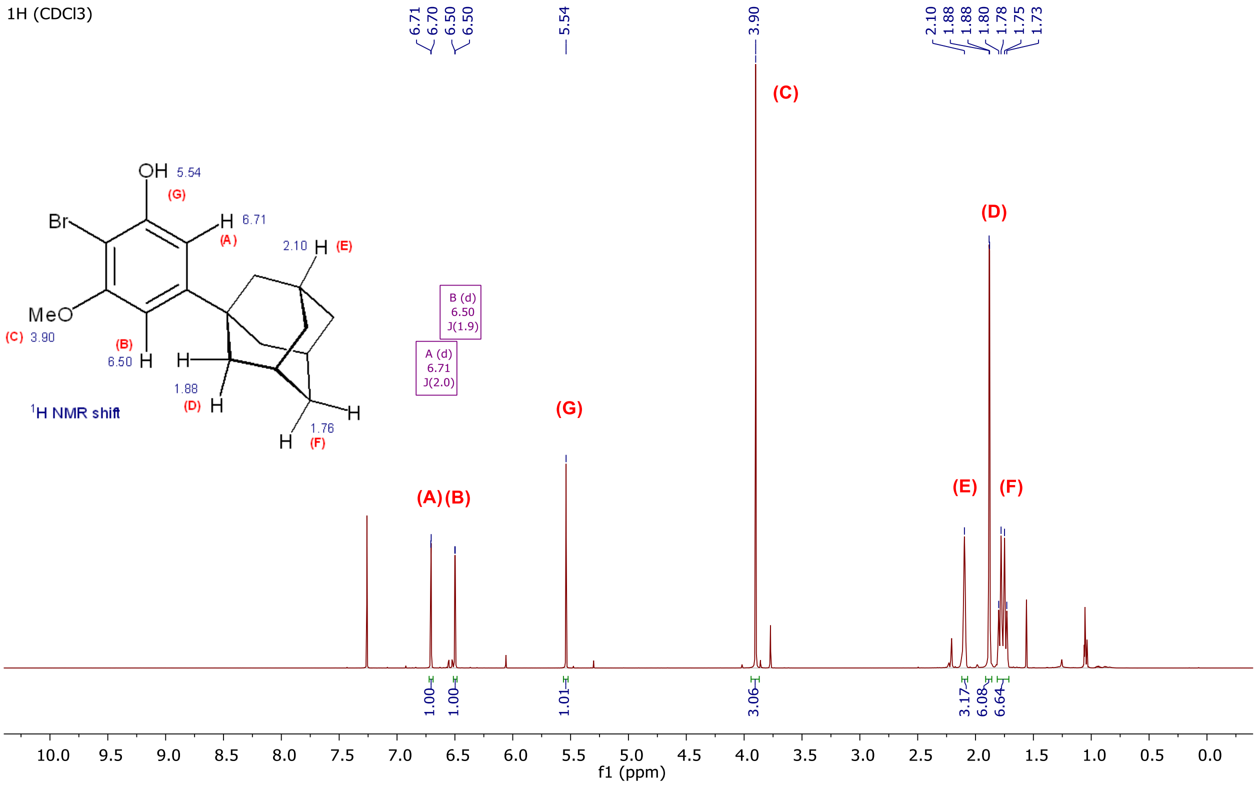
1H (CDCl3)
6.71
6.70
6.50
6.50
5.54
3.90
2.10
1.88
1.88
1.80
1.78
1.75
1.73
(C)
(D)
(G)
(A)(B)
(E)
(F)
OH 5.54
(G)
Br
H 6.71
(A)
2.10 H (E)
MeO
(C) 3.90
(B)
6.50 H
H
1.88
(D) H
H
1.76
H (F)
¹H NMR shift
B (d)
6.50
J(1.9)
A (d)
6.71
J(2.0)
1.00
1.00
1.01
3.06
3.17
6.08
6.64
10.0 9.5 9.0 8.5 8.0 7.5 7.0 6.5 6.0 5.5 5.0 4.5 4.0 3.5 3.0 2.5 2.0 1.5 1.0 0.5 0.0
f1 (ppm)

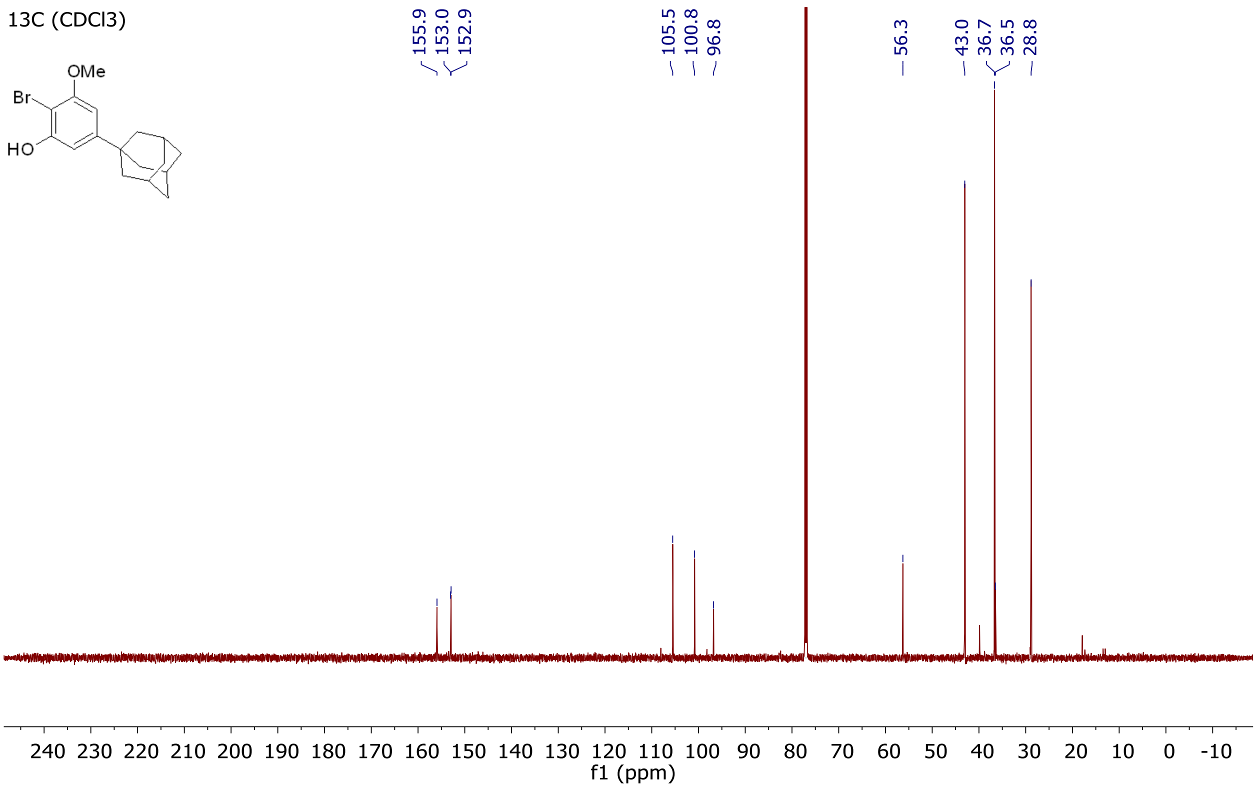
13C (CDCl3)
155.9
153.0
152.9
105.5
100.8
96.8
56.3
43.0
36.7
36.5
28.8
OMe
Br
HO
240 230 220 210 200 190 180 170 160 150 140 130 120 110 100 90 80 70 60 50 40 30 20 10 0 -10
f1 (ppm)

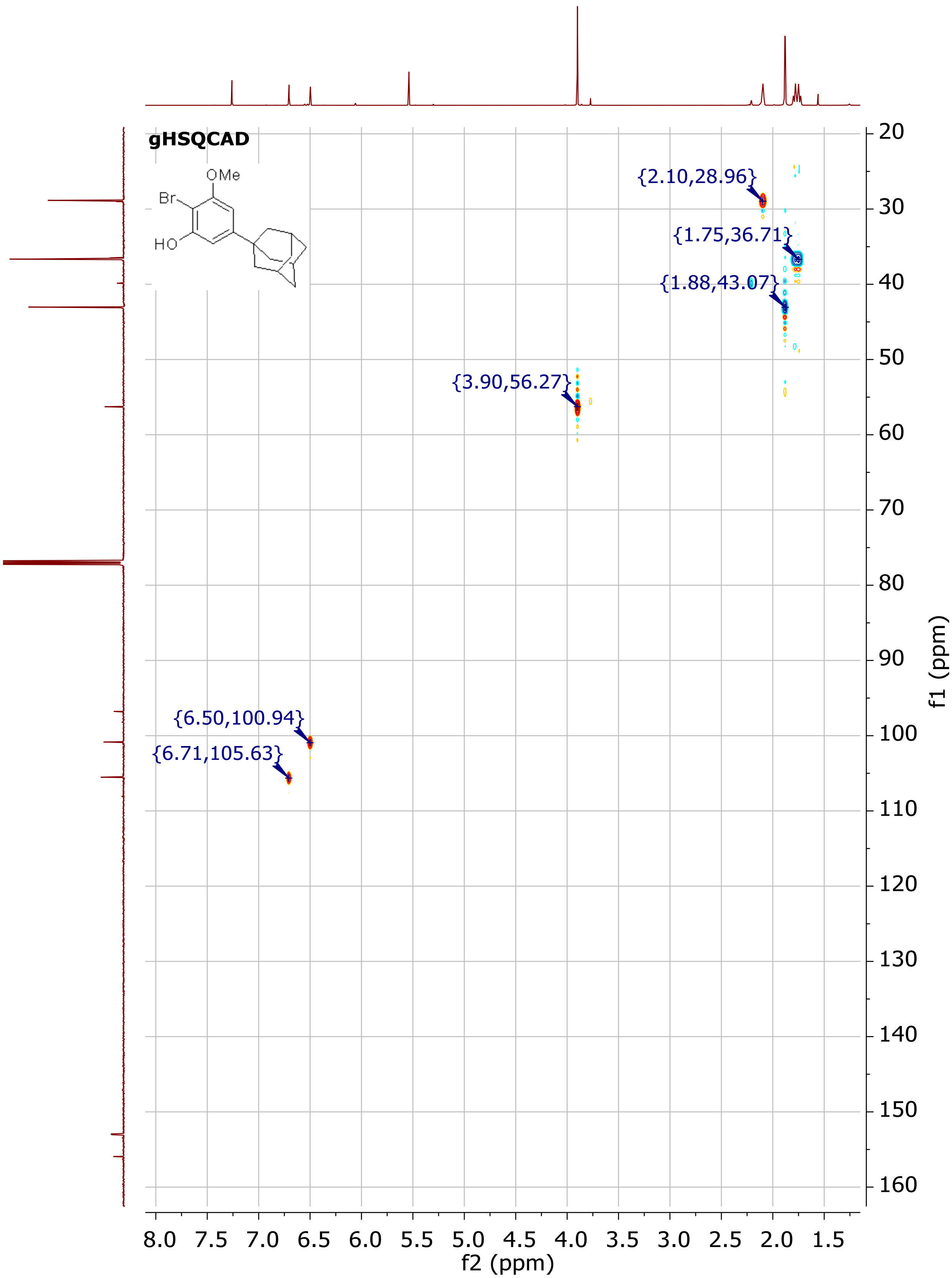
gHSQCAD
OMe
Br
HO
{2.10,28.96}
{1.75,36.71}
{1.88,43.07}
{3.90,56.27}
{6.50,100.94}
{6.71,105.63}
f2 (ppm)
f1 (ppm)

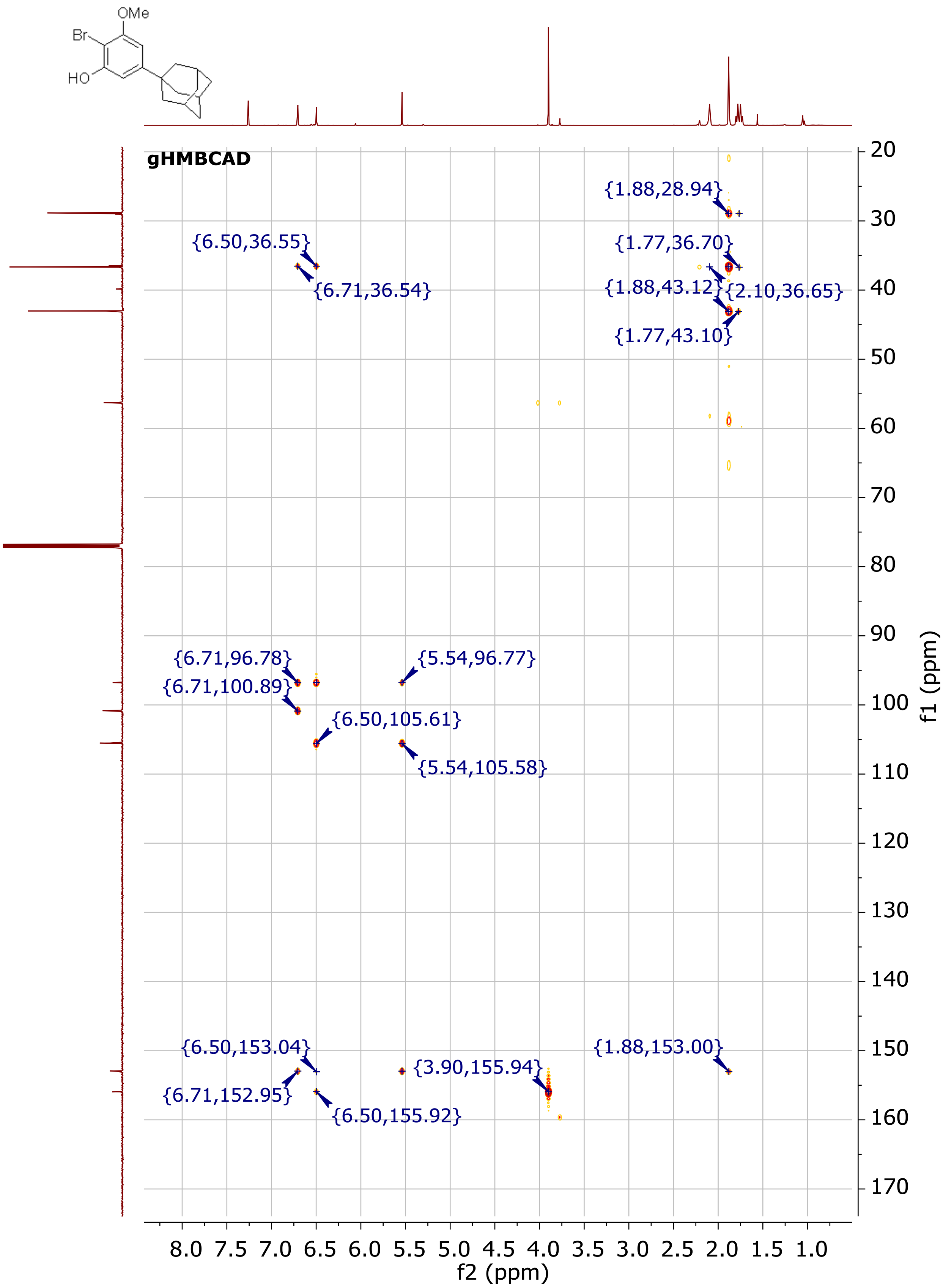
OMe
Br
HO
gHMBCAD
{1.88,28.94}
{6.50,36.55}
{1.77,36.70}
{6.71,36.54}
{1.88,43.12}
{2.10,36.65}
{1.77,43.10}
{6.71,96.78}
{5.54,96.77}
{6.71,100.89}
{6.50,105.61}
{5.54,105.58}
{6.50,153.04}
{1.88,153.00}
{3.90,155.94}
{6.71,152.95}
{6.50,155.92}
f1 (ppm)
f2 (ppm)

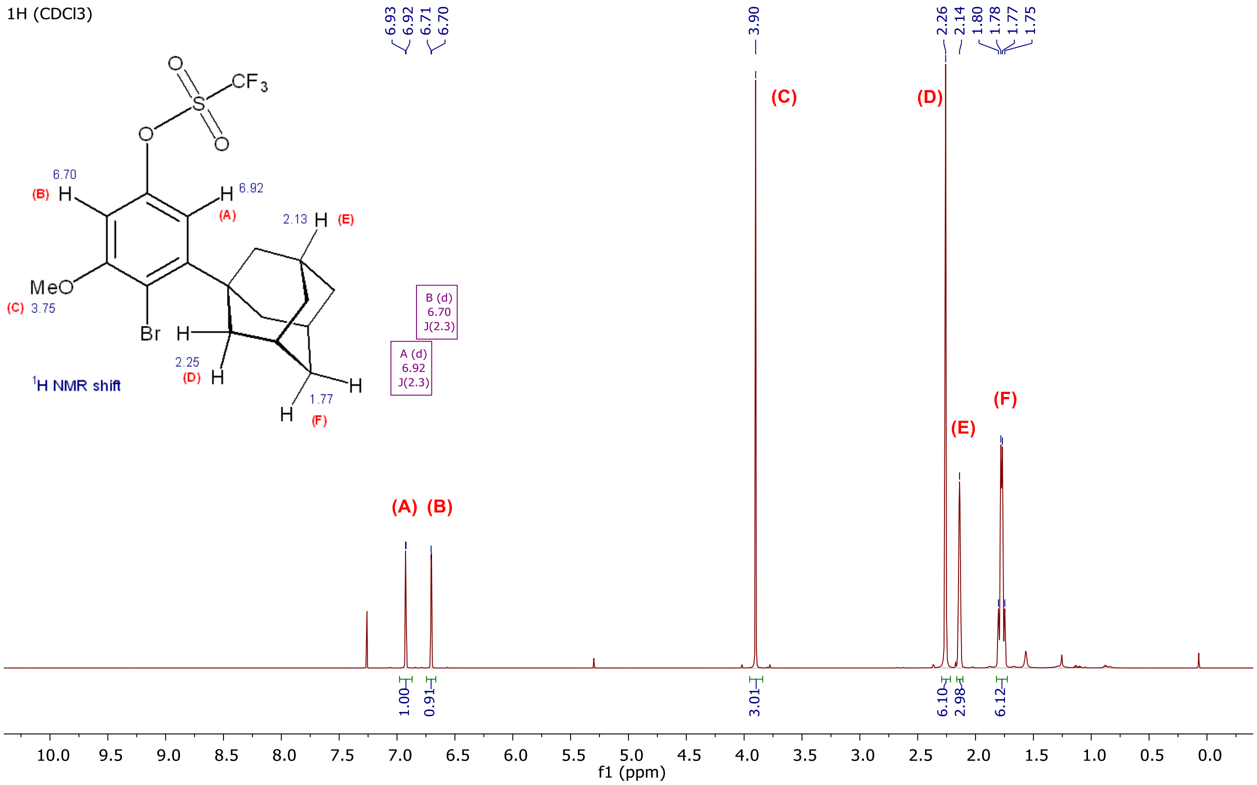
1H (CDCl3)
6.93
6.92
6.71
6.70
3.90
2.26
2.14
1.80
1.78
1.77
1.75
O
CF3
S
O
O
6.70
(B) H
H 6.92
(A)
2.13 H (E)
MeO
(C) 3.75
Br H
2.25
(D) H
H
1.77
H (F)
1H NMR shift
B (d)
6.70
J(2.3)
A (d)
6.92
J(2.3)
(C)
(D)
(E)
(F)
(A) (B)
1.00
0.91
3.01
6.10
2.98
6.12
10.0 9.5 9.0 8.5 8.0 7.5 7.0 6.5 6.0 5.5 5.0 4.5 4.0 3.5 3.0 2.5 2.0 1.5 1.0 0.5 0.0
f1 (ppm)

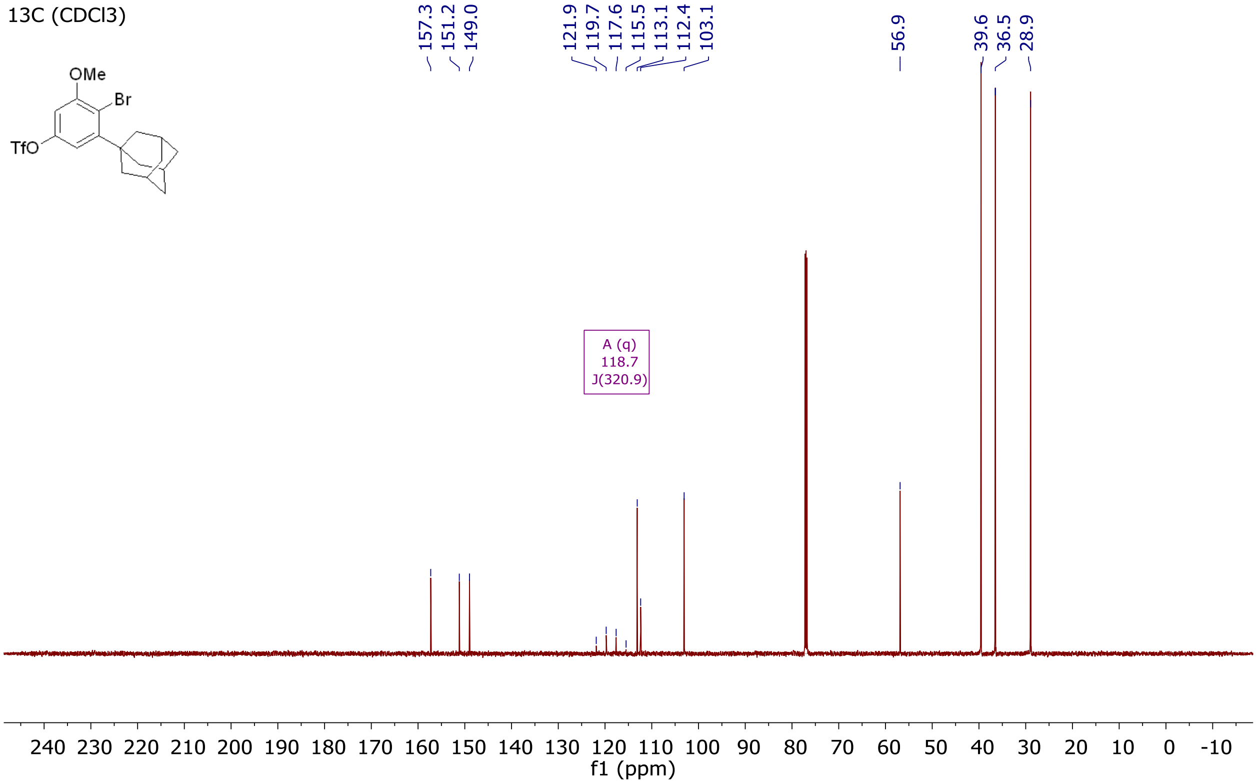
13C (CDCl3)
157.3
151.2
149.0
121.9
119.7
117.6
115.5
113.1
112.4
103.1
56.9
39.6
36.5
28.9
OMe
Br
TfO
A (q)
118.7
J(320.9)
240 230 220 210 200 190 180 170 160 150 140 130 120 110 100 90 80 70 60 50 40 30 20 10 0 -10
f1 (ppm)

19F (CDCl3)

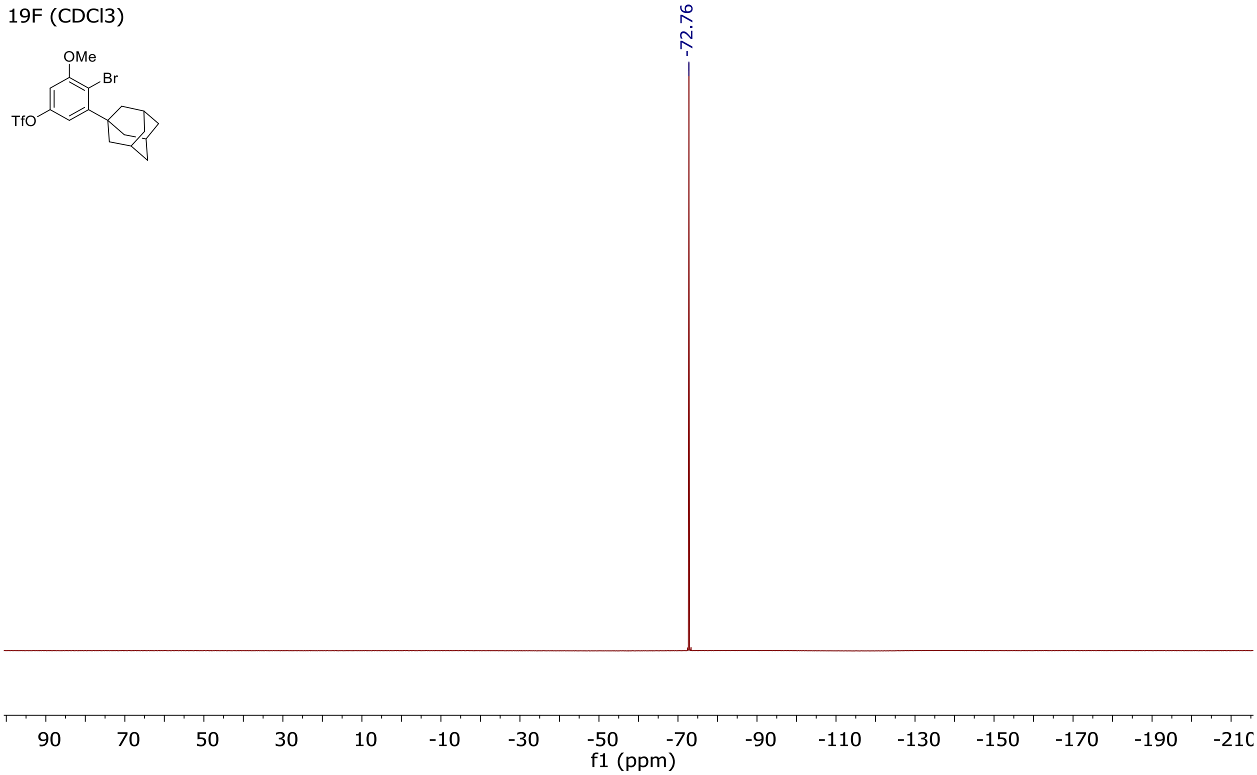
OMe
Br
TfO
-72.76
90 70 50 30 10 -10 -30 -50 -70 -90 -110 -130 -150 -170 -190 -210
f1 (ppm)

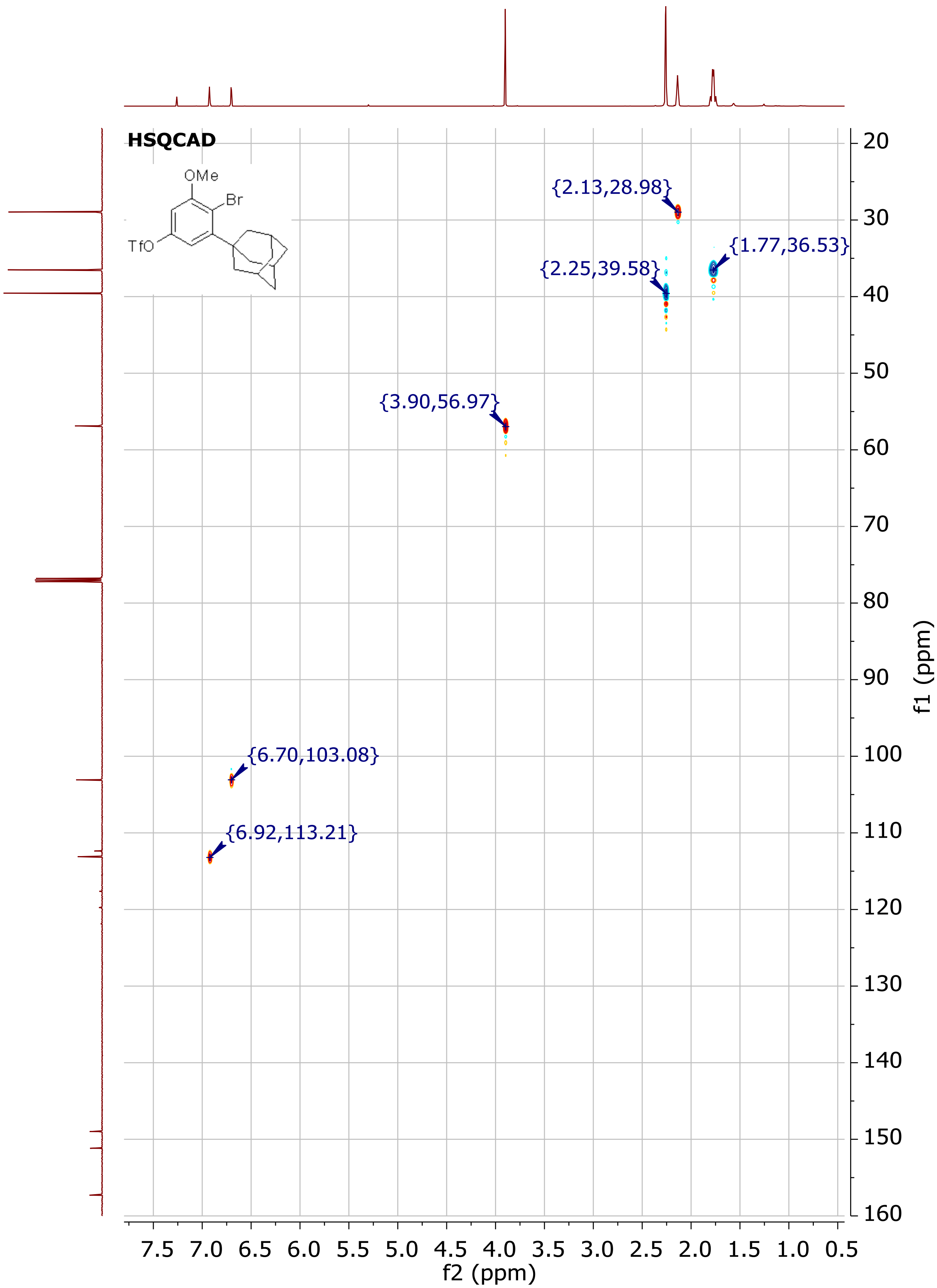
HSQCAD
OMe
Br
TfO
{2.13,28.98}
{1.77,36.53}
{2.25,39.58}
{3.90,56.97}
{6.70,103.08}
{6.92,113.21}
f2 (ppm)
f1 (ppm)

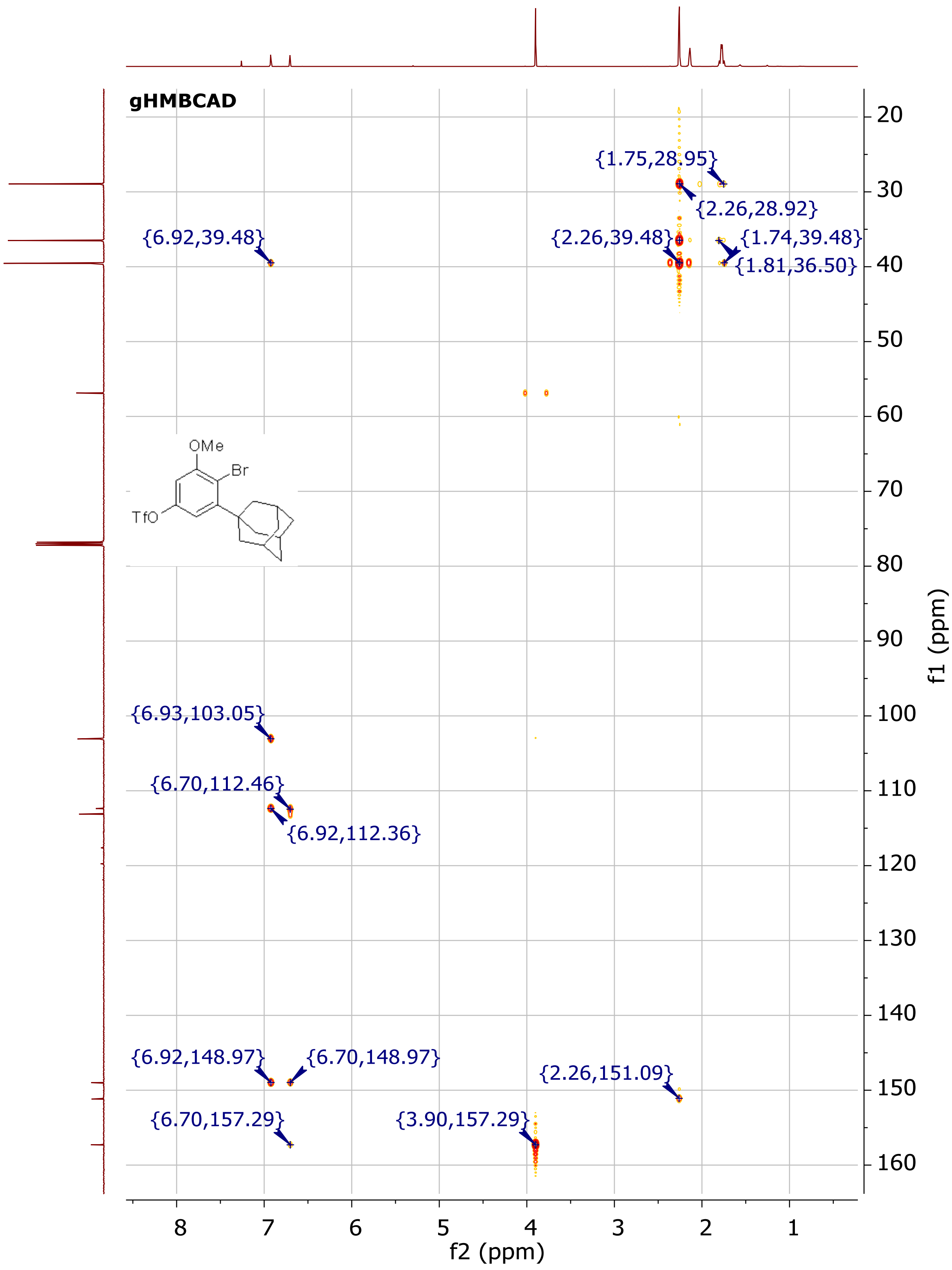
gHMBCAD
{1.75,28.95}
{2.26,28.92}
{6.92,39.48}
{2.26,39.48}
{1.74,39.48}
{1.81,36.50}
OMe
Br
TfO
{6.93,103.05}
{6.70,112.46}
{6.92,112.36}
{6.92,148.97}
{6.70,148.97}
{2.26,151.09}
{6.70,157.29}
{3.90,157.29}
f1 (ppm)
f2 (ppm)

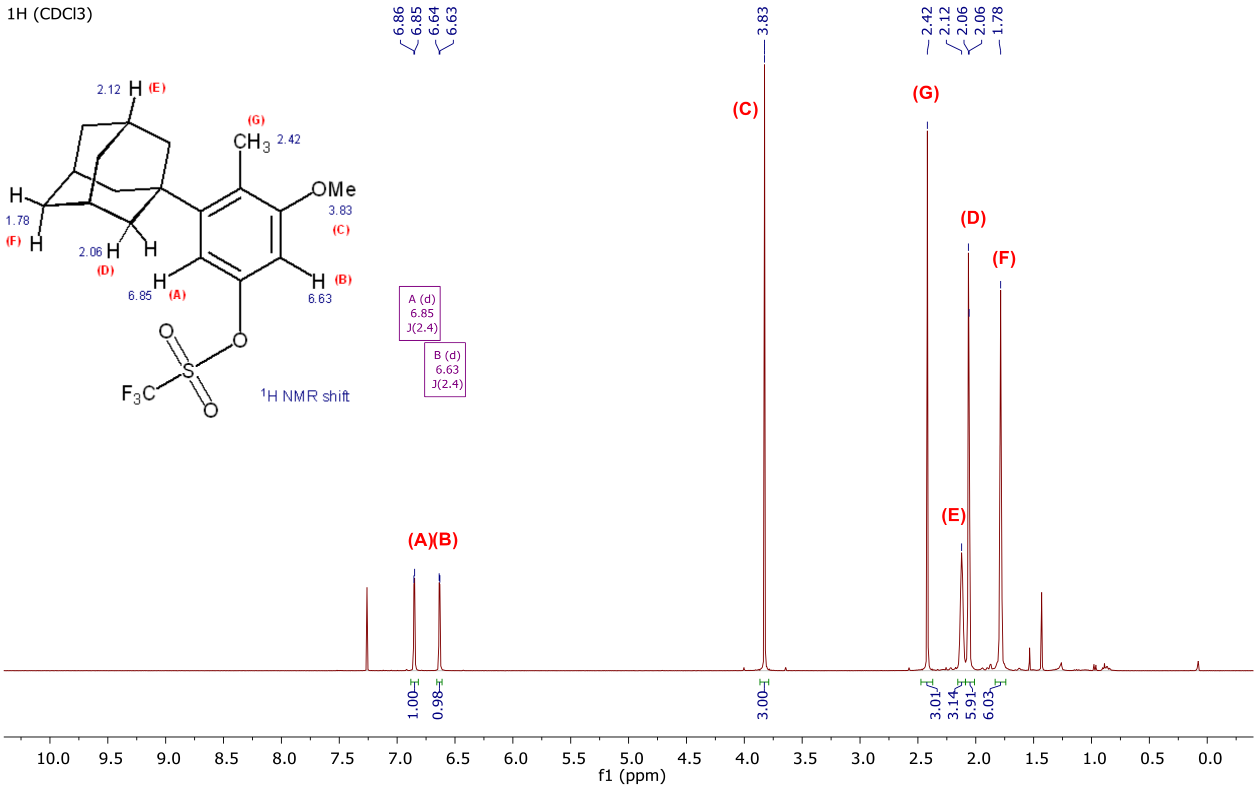
1H (CDCl3)
6.86
6.85
6.64
6.63
3.83
2.42
2.12
2.06
2.06
1.78
2.12 H (E)
(G)
CH3 2.42
OMe
3.83
(C)
H
1.78
(F) H
2.06 H
(D)
H
H (B)
6.63
H
6.85 (A)
O
O
S
F3C
O
O
1H NMR shift
A (d)
6.85
J(2.4)
B (d)
6.63
J(2.4)
(C)
(G)
(D)
(F)
(E)
(A)(B)
1.00
0.98
3.00
3.01
3.14
5.91
6.03
10.0 9.5 9.0 8.5 8.0 7.5 7.0 6.5 6.0 5.5 5.0 4.5 4.0 3.5 3.0 2.5 2.0 1.5 1.0 0.5 0.0
f1 (ppm)

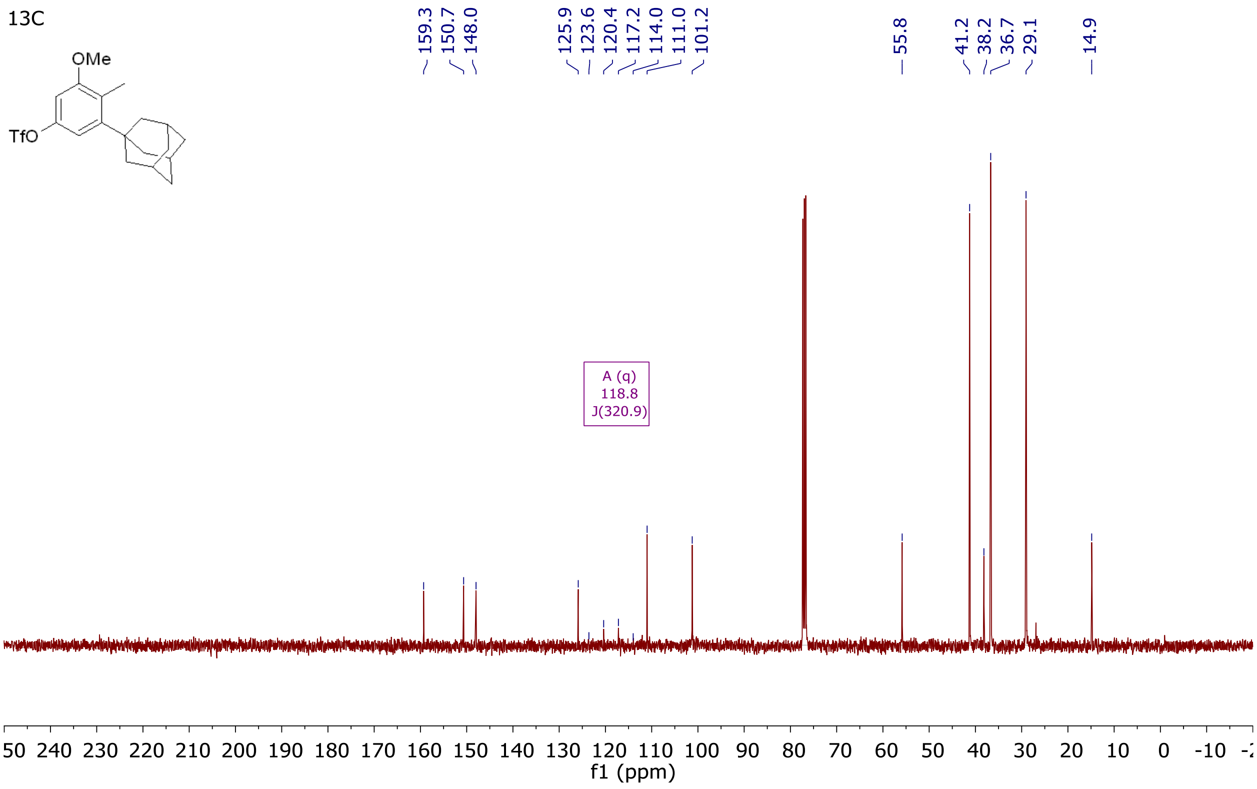
13C
159.3
150.7
148.0
125.9
123.6
120.4
117.2
114.0
111.0
101.2
55.8
41.2
38.2
36.7
29.1
14.9
OMe
TfO
A (q)
118.8
J(320.9)
50 240 230 220 210 200 190 180 170 160 150 140 130 120 110 100 90 80 70 60 50 40 30 20 10 0 -10
f1 (ppm)

19F (CDCl3)

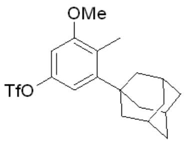

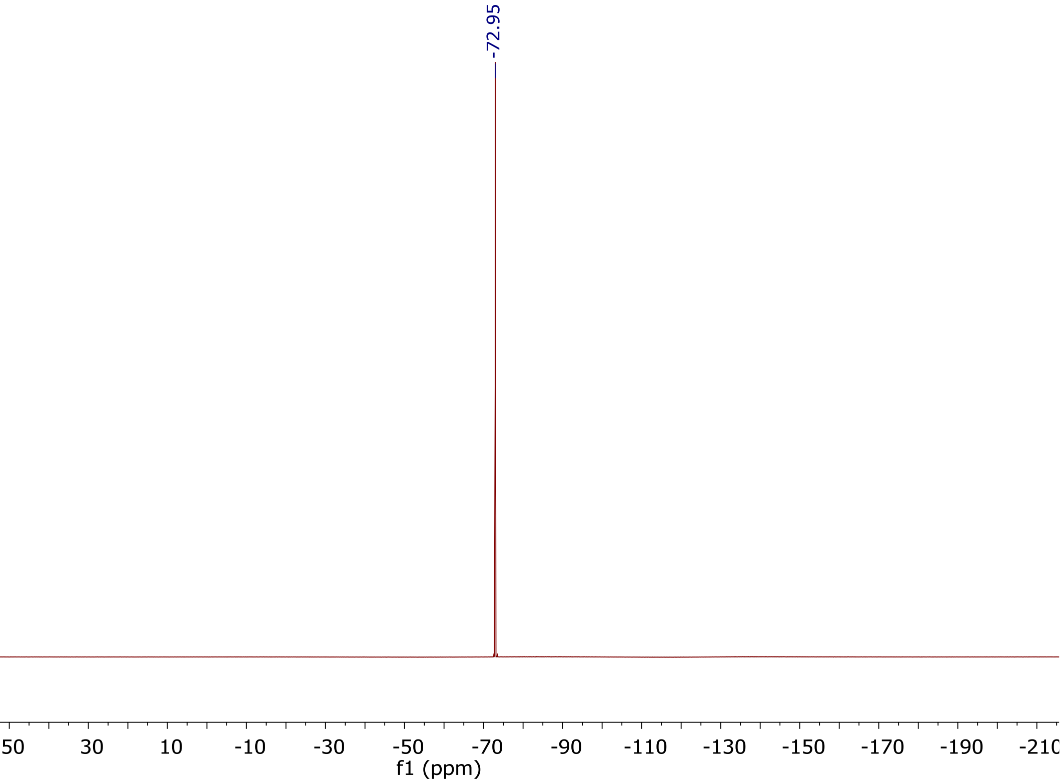

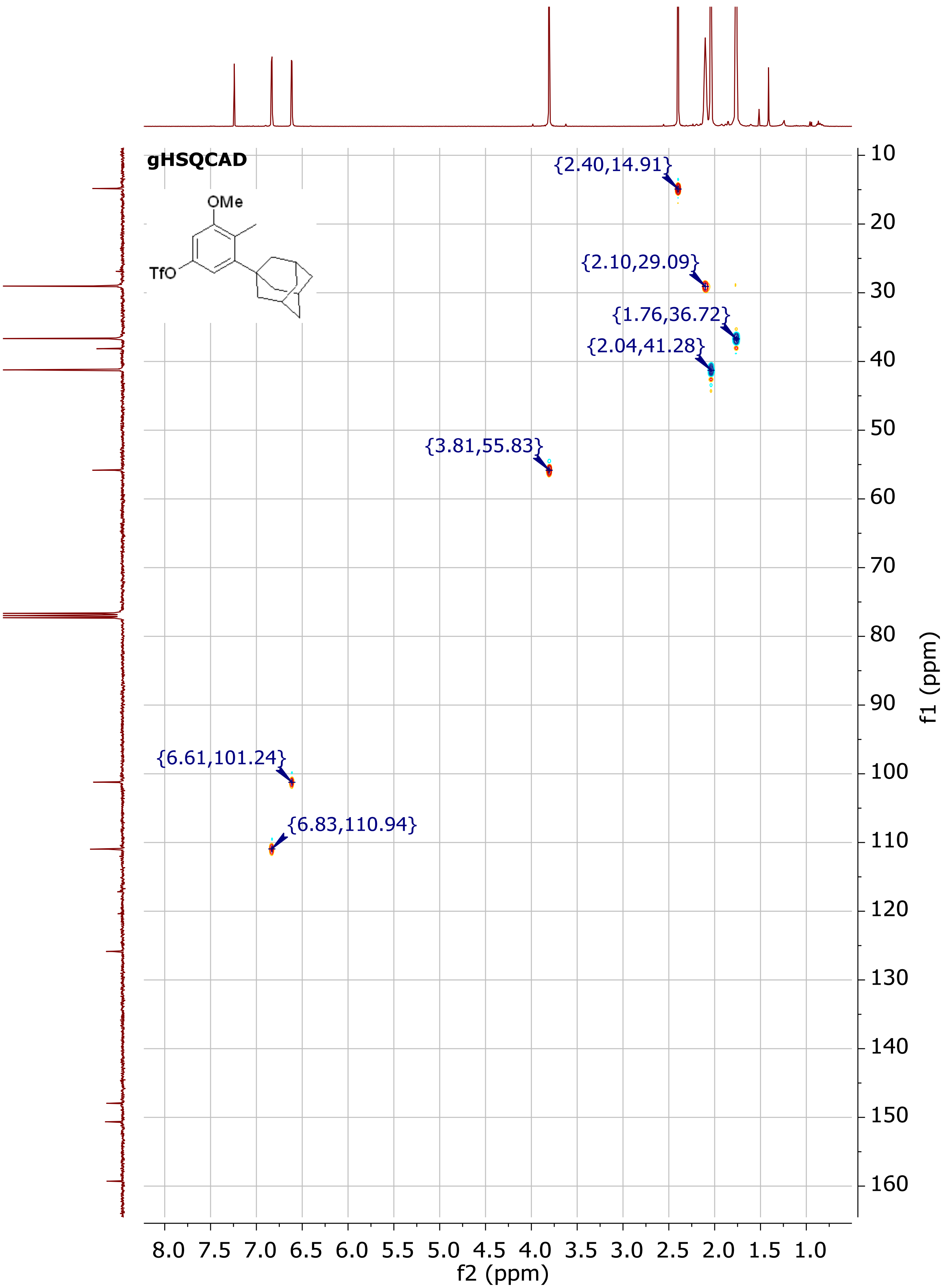
gHSQCAD
OMe
TfO
{2.40,14.91}
{2.10,29.09}
{1.76,36.72}
{2.04,41.28}
{3.81,55.83}
{6.61,101.24}
{6.83,110.94}
f1 (ppm)
f2 (ppm)

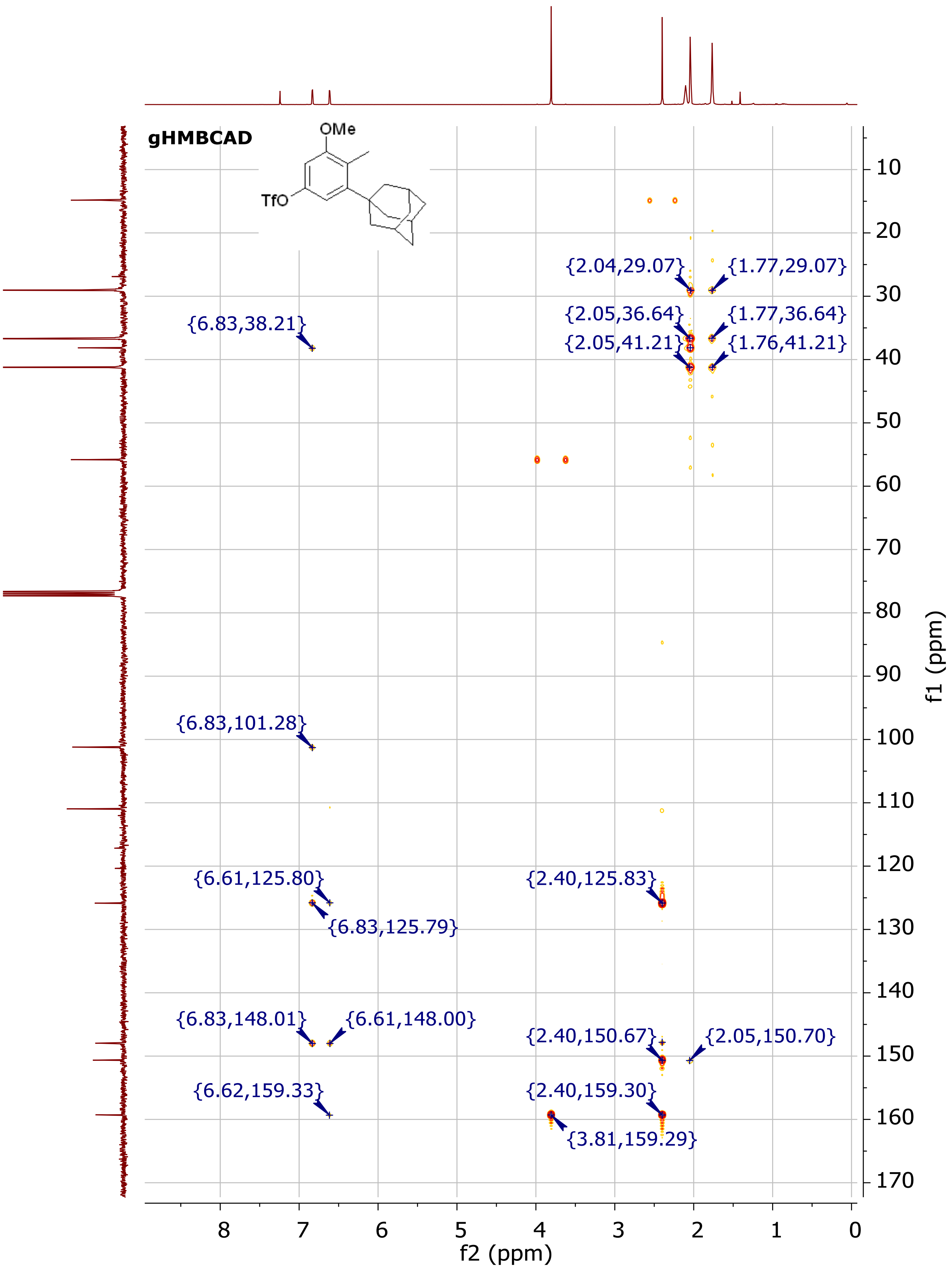
gHMBCAD
OMe
TfO
{2.04,29.07}
{1.77,29.07}
{6.83,38.21}
{2.05,36.64}
{1.77,36.64}
{2.05,41.21}
{1.76,41.21}
{6.83,101.28}
{6.61,125.80}
{2.40,125.83}
{6.83,125.79}
{6.83,148.01}
{6.61,148.00}
{2.40,150.67}
{2.05,150.70}
{6.62,159.33}
{2.40,159.30}
{3.81,159.29}
f2 (ppm)
f1 (ppm)

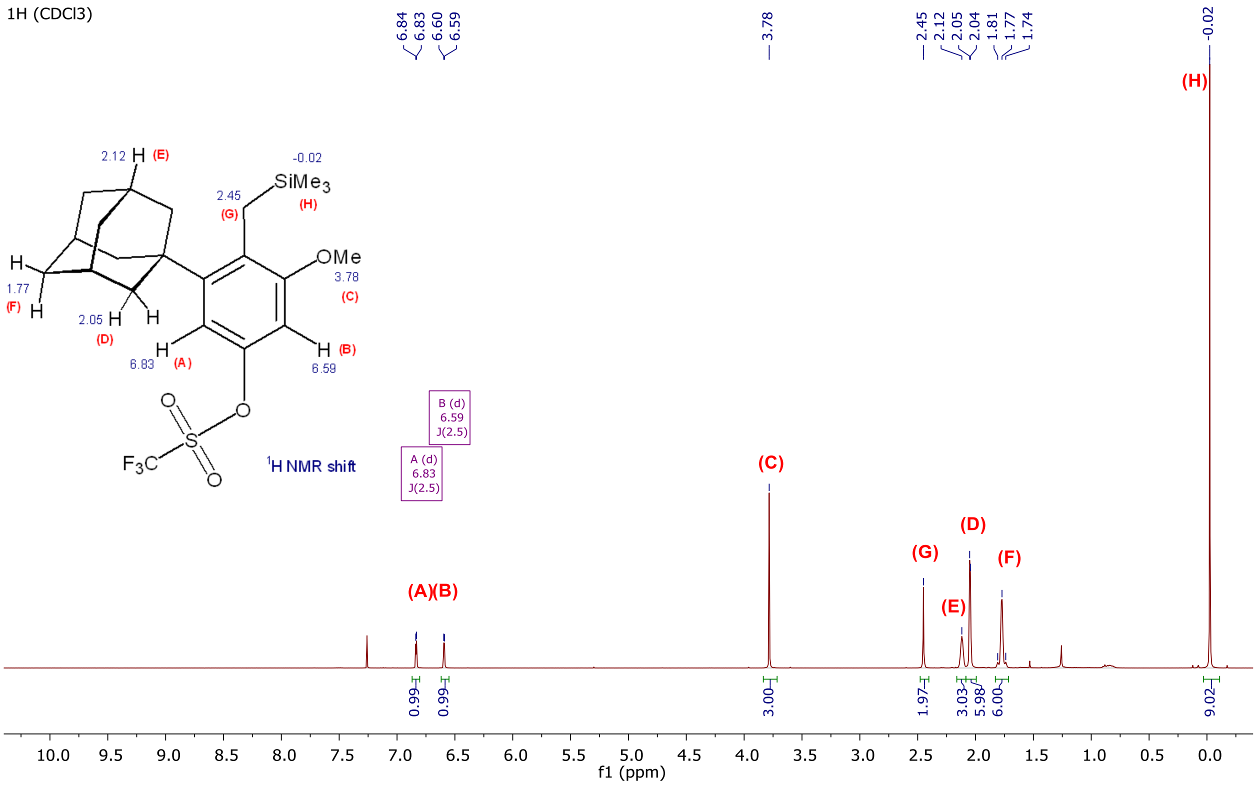
1H (CDCl3)
6.84
6.83
6.60
6.59
3.78
2.45
2.12
2.05
2.04
1.81
1.77
1.74
-0.02
2.12 H (E)
-0.02
SiMe3
2.45 (G)
(H)
OMe
3.78
(C)
1.77 (F)
2.05 H (D)
H (B)
6.83 (A)
6.59
F3C
1H NMR shift
B (d) 6.59 J(2.5)
A (d) 6.83 J(2.5)
(C)
(D)
(G)
(F)
(E)
(A)(B)
0.99
0.99
3.00
1.97
3.03
5.98
6.00
9.02
f1 (ppm)

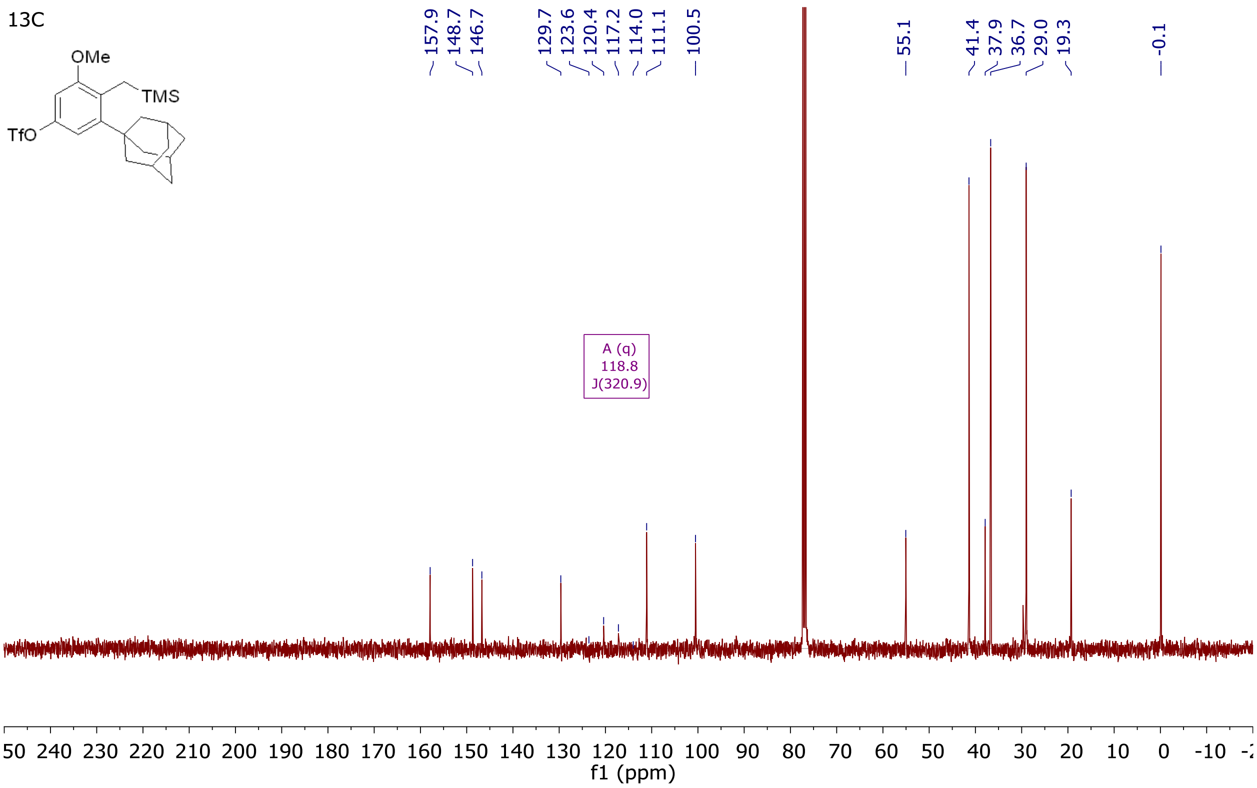
13C
157.9
148.7
146.7
129.7
123.6
120.4
117.2
114.0
111.1
100.5
55.1
41.4
37.9
36.7
29.0
19.3
-0.1
OMe
TMS
TfO
A (q) 118.8 J(320.9)
f1 (ppm)

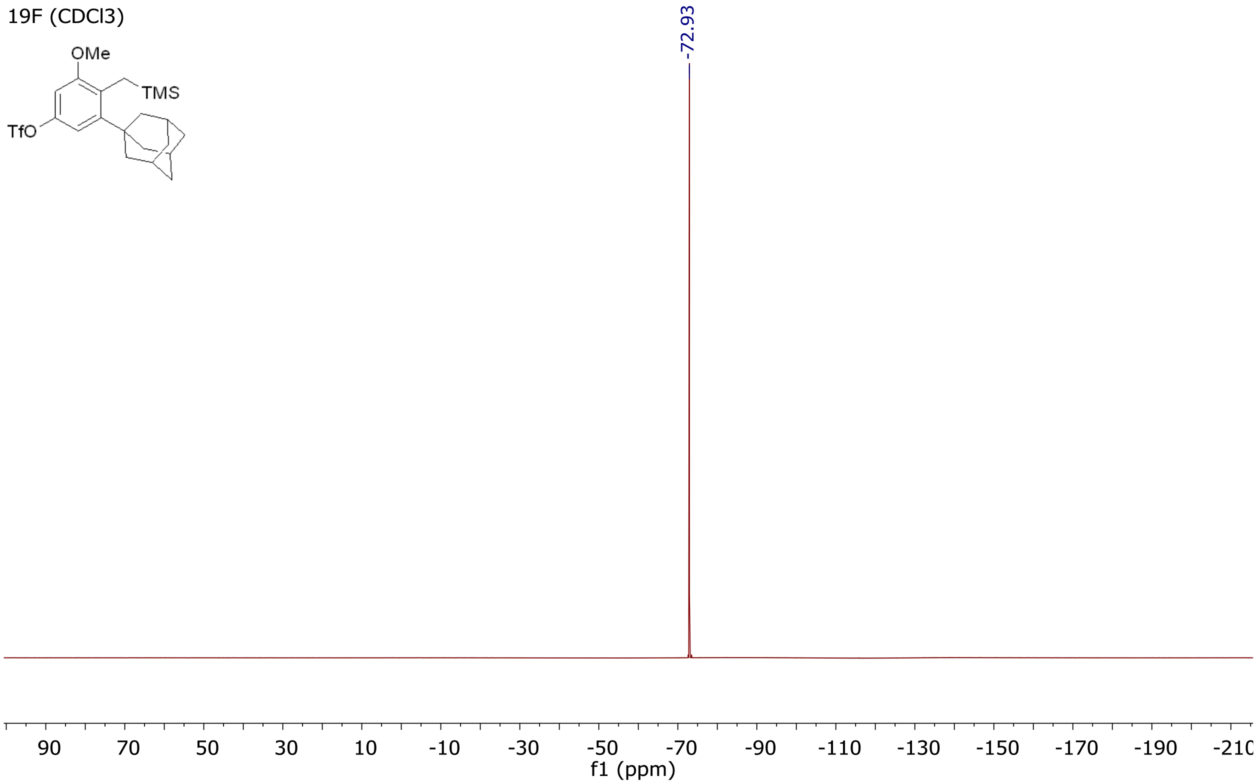

19F (CDCl3)
OMe
TMS
TfO
-72.93
90 70 50 30 10 -10 -30 -50 -70 -90 -110 -130 -150 -170 -190 -210
f1 (ppm)

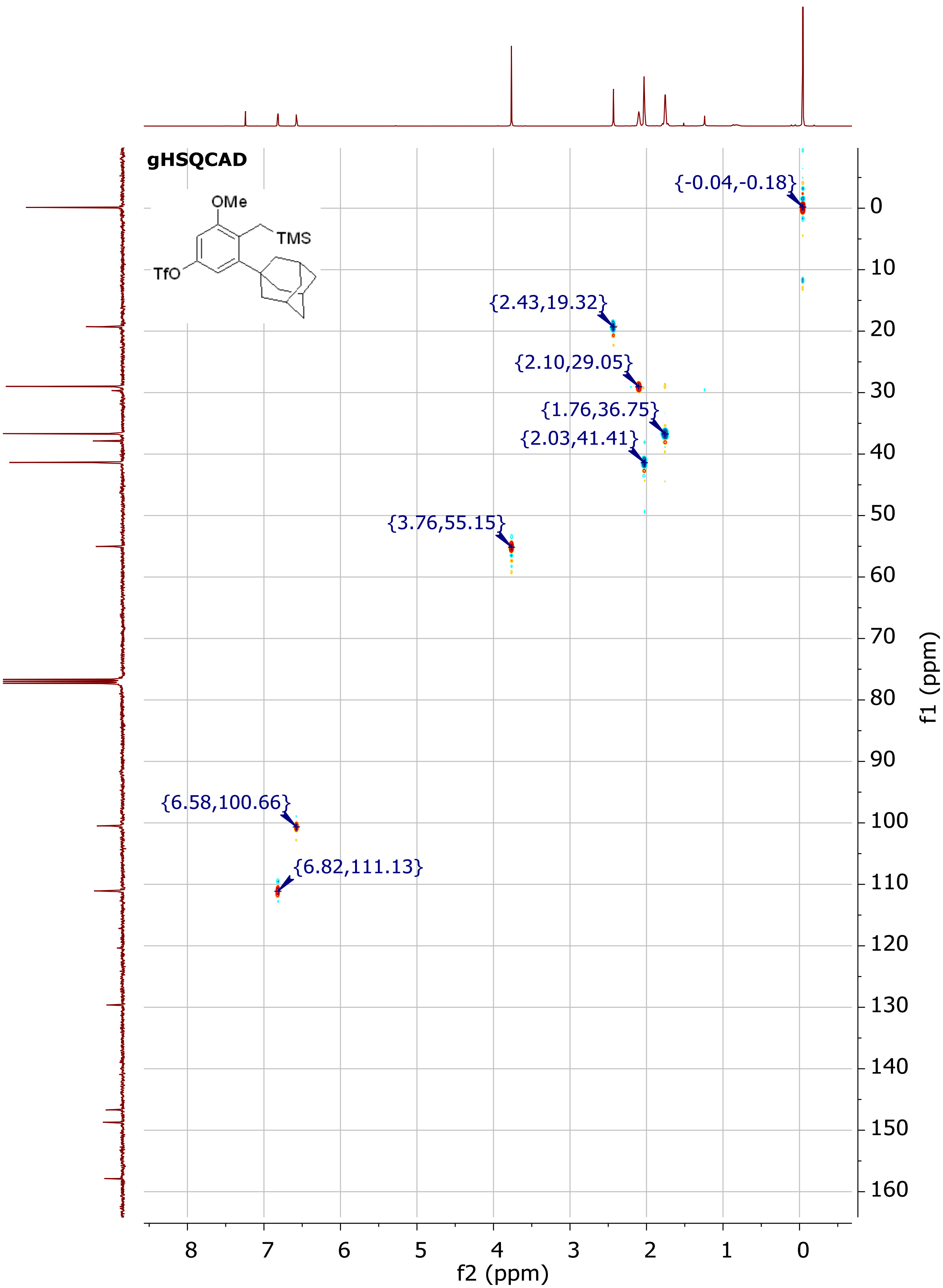
gHSQCAD
OMe
TMS
TfO
{-0.04,-0.18}
{2.43,19.32}
{2.10,29.05}
{1.76,36.75}
{2.03,41.41}
{3.76,55.15}
{6.58,100.66}
{6.82,111.13}
f2 (ppm)
f1 (ppm)

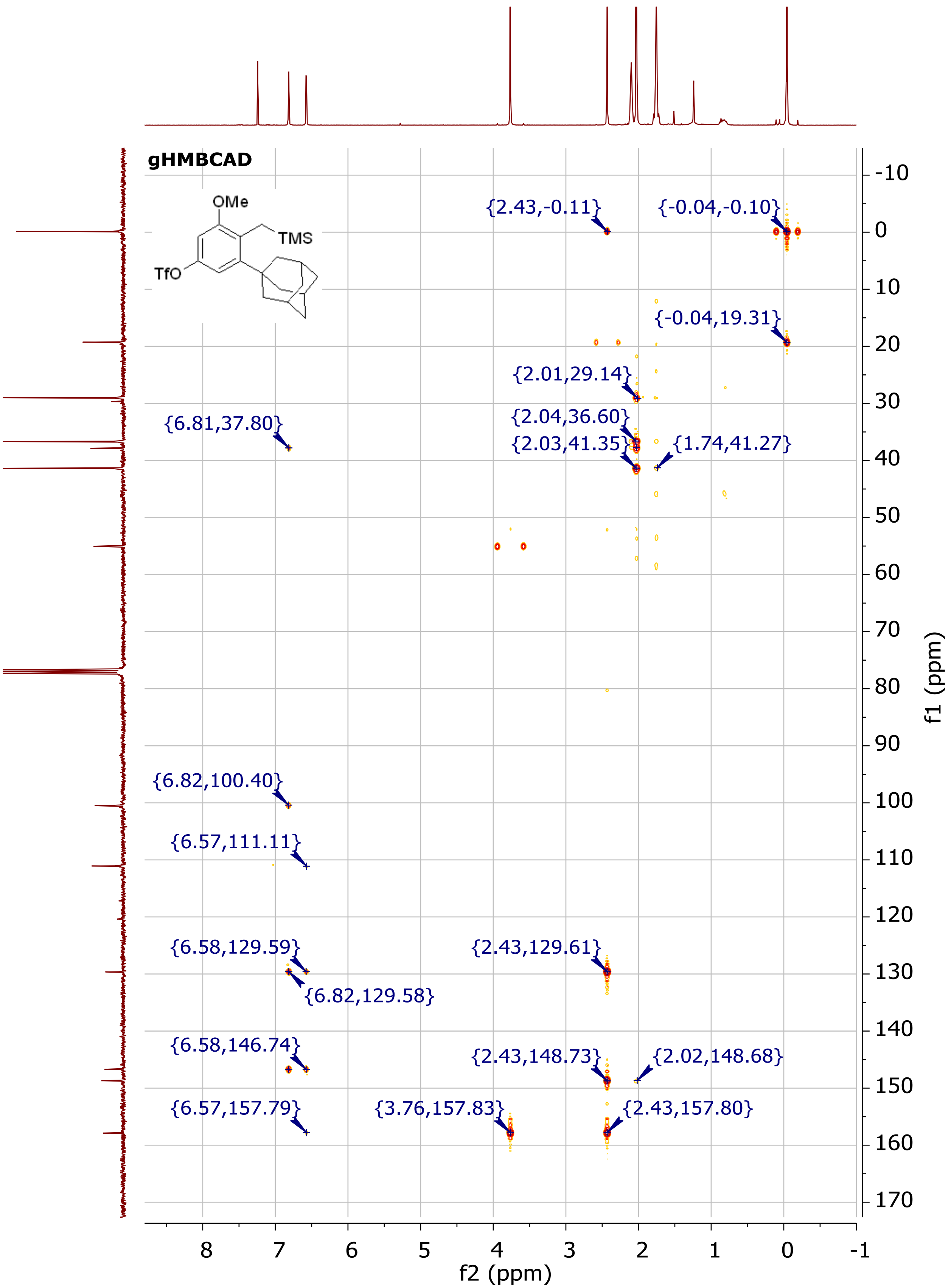
gHMBCAD
OMe
TMS
TfO
{2.43,-0.11}
{-0.04,-0.10}
{-0.04,19.31}
{2.01,29.14}
{2.04,36.60}
{6.81,37.80}
{2.03,41.35}
{1.74,41.27}
{6.82,100.40}
{6.57,111.11}
{6.58,129.59}
{2.43,129.61}
{6.82,129.58}
{6.58,146.74}
{2.43,148.73}
{2.02,148.68}
{6.57,157.79}
{3.76,157.83}
{2.43,157.80}
f1 (ppm)
f2 (ppm)

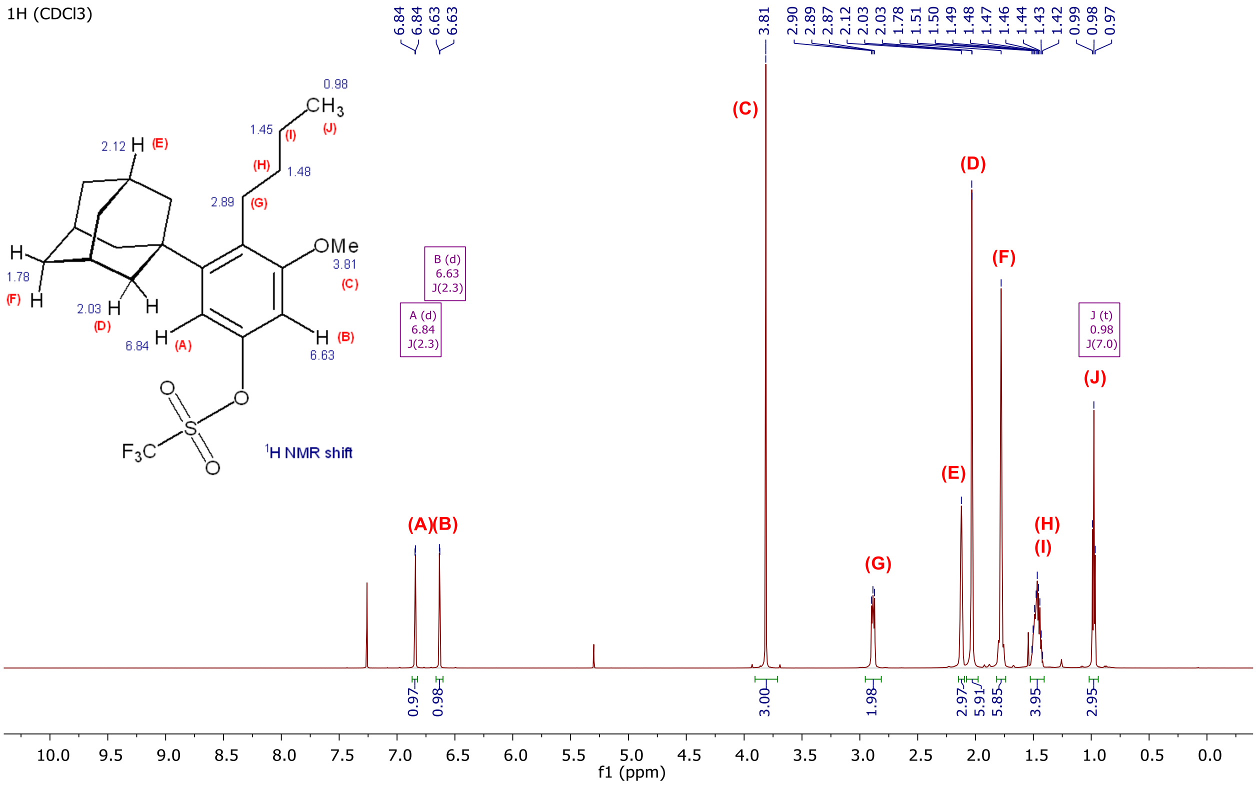
1H (CDCl3)
6.84
6.84
6.63
6.63
3.81
2.90
2.89
2.87
2.12
2.03
1.78
1.51
1.50
1.49
1.48
1.47
1.46
1.44
1.43
1.42
0.99
0.98
0.97
B (d)
6.63
J(2.3)
A (d)
6.84
J(2.3)
J (t)
0.98
J(7.0)
(A)(B)
(C)
(D)
(E)
(F)
(G)
(H)
(I)
(J)
OMe
F3C
1H NMR shift
0.97
0.98
3.00
1.98
2.97
5.91
5.85
3.95
2.95
10.0 9.5 9.0 8.5 8.0 7.5 7.0 6.5 6.0 5.5 5.0 4.5 4.0 3.5 3.0 2.5 2.0 1.5 1.0 0.5 0.0
f1 (ppm)

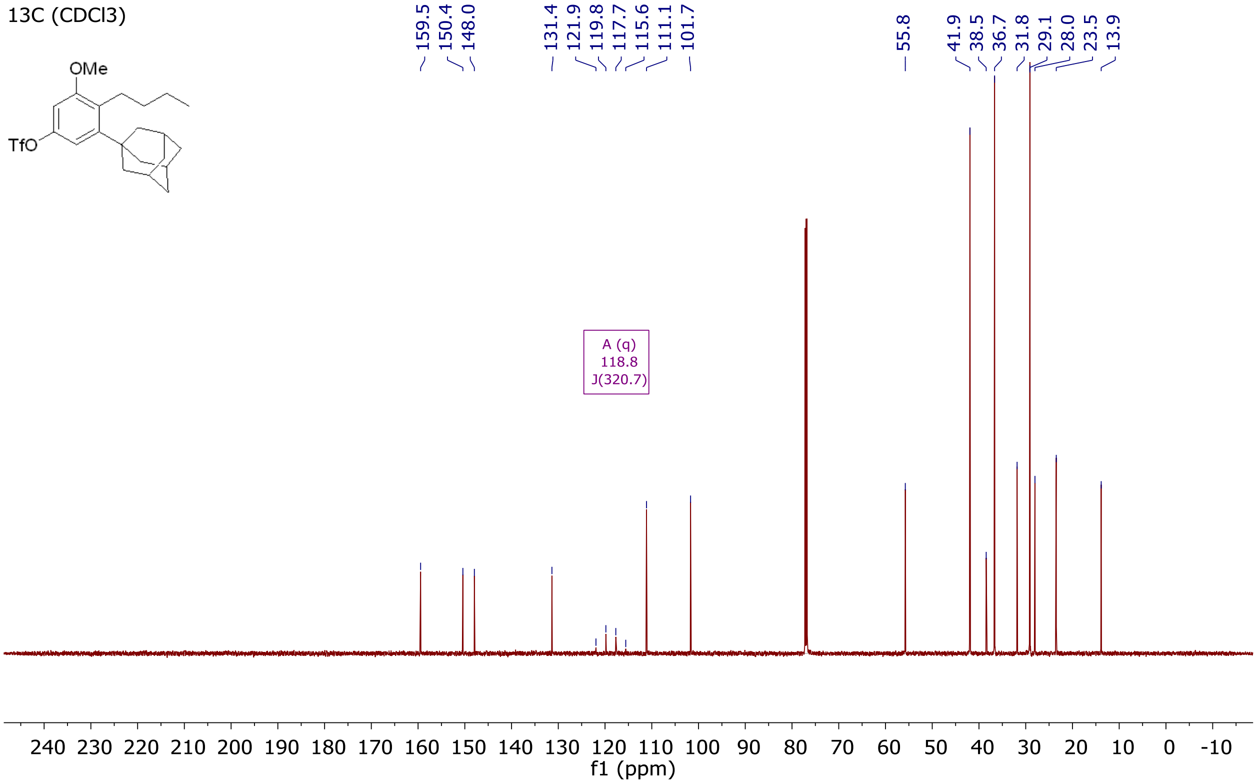
13C (CDCl3)
159.5
150.4
148.0
131.4
121.9
119.8
117.7
115.6
111.1
101.7
55.8
41.9
38.5
36.7
31.8
29.1
28.0
23.5
13.9
OMe
TfO
A (q)
118.8
J(320.7)
240 230 220 210 200 190 180 170 160 150 140 130 120 110 100 90 80 70 60 50 40 30 20 10 0 -10
f1 (ppm)

19F (CDCl3)

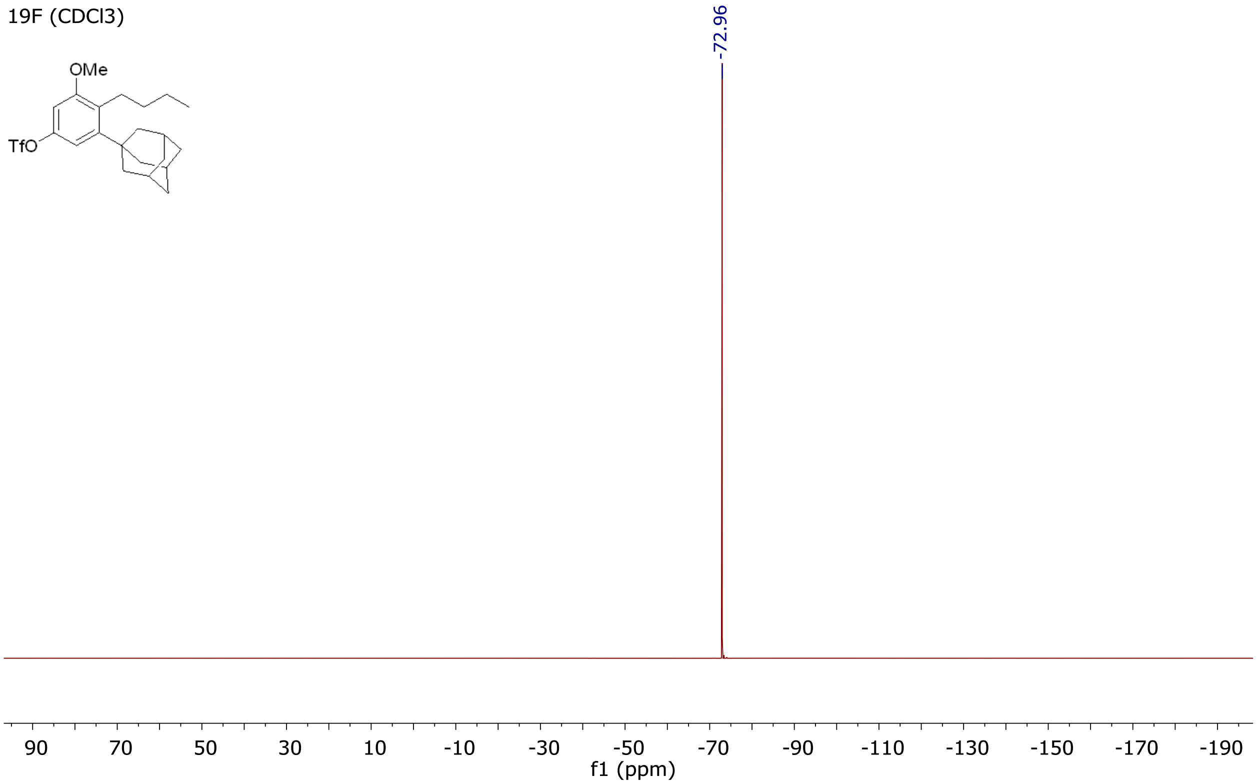
OMe
TfO
-72.96
90
70
50
30
10
-10
-30
-50
-70
-90
-110
-130
-150
-170
-190
f1 (ppm)

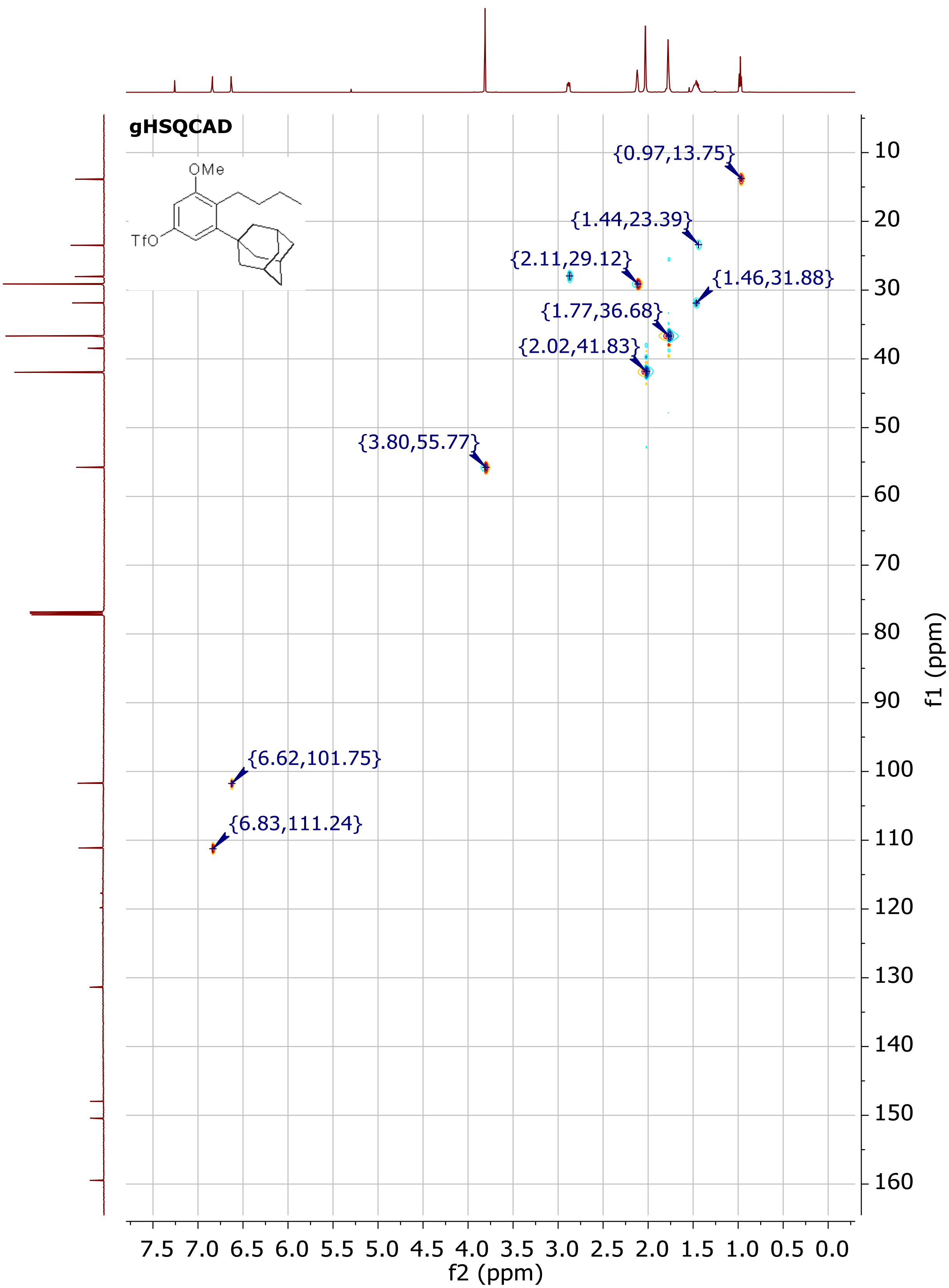
gHSQCAD
OMe
TfO
{0.97,13.75}
{1.44,23.39}
{2.11,29.12}
{1.46,31.88}
{1.77,36.68}
{2.02,41.83}
{3.80,55.77}
{6.62,101.75}
{6.83,111.24}
f2 (ppm)
f1 (ppm)
7.5 7.0 6.5 6.0 5.5 5.0 4.5 4.0 3.5 3.0 2.5 2.0 1.5 1.0 0.5 0.0
10 20 30 40 50 60 70 80 90 100 110 120 130 140 150 160

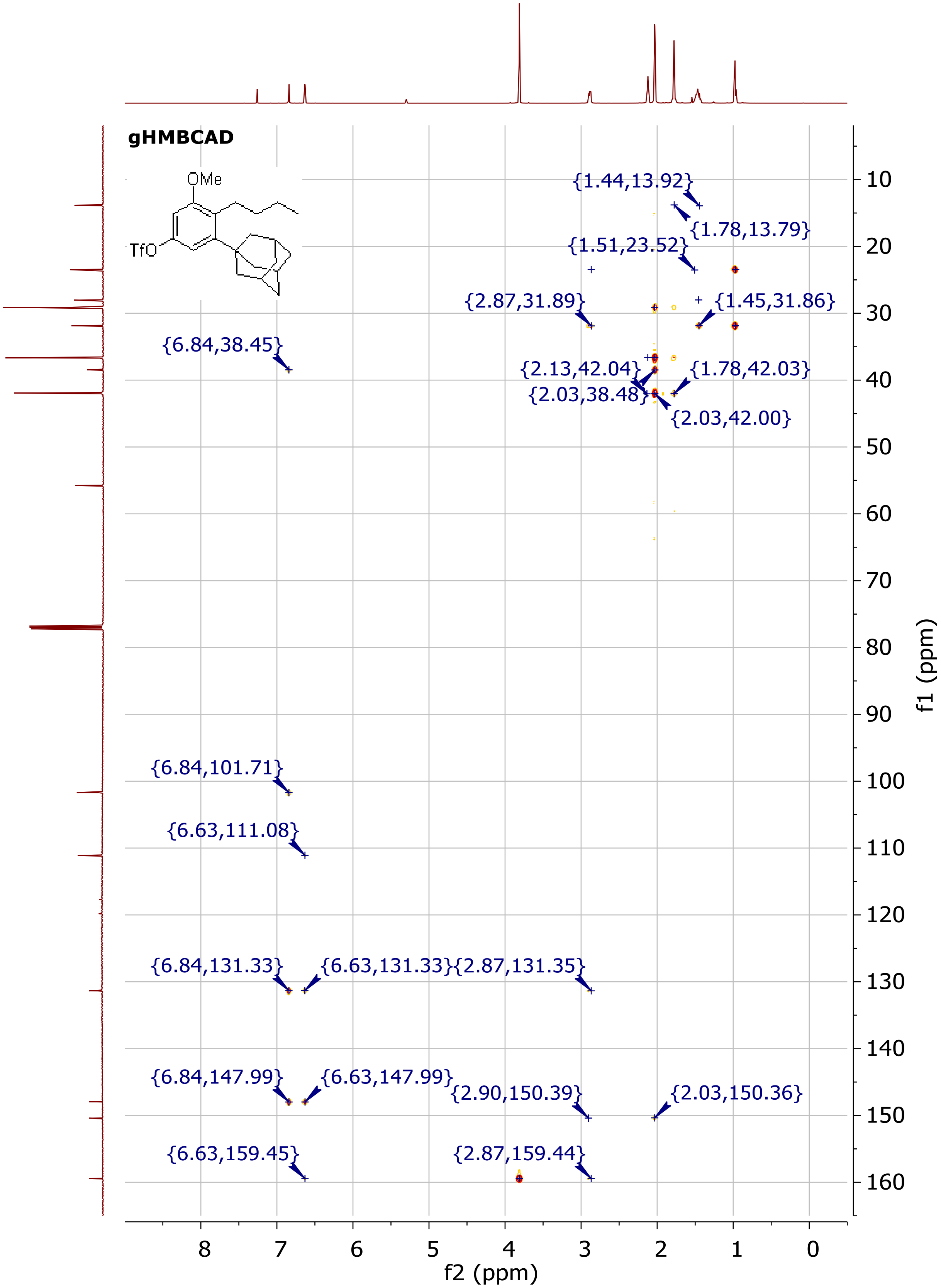
gHMBCAD
OMe
TfO
{1.44,13.92}
{1.78,13.79}
{1.51,23.52}
{2.87,31.89}
{1.45,31.86}
{6.84,38.45}
{2.13,42.04}
{1.78,42.03}
{2.03,38.48}
{2.03,42.00}
{6.84,101.71}
{6.63,111.08}
{6.84,131.33}
{6.63,131.33}
{2.87,131.35}
{6.84,147.99}
{6.63,147.99}
{2.90,150.39}
{2.03,150.36}
{6.63,159.45}
{2.87,159.44}
f1 (ppm)
f2 (ppm)

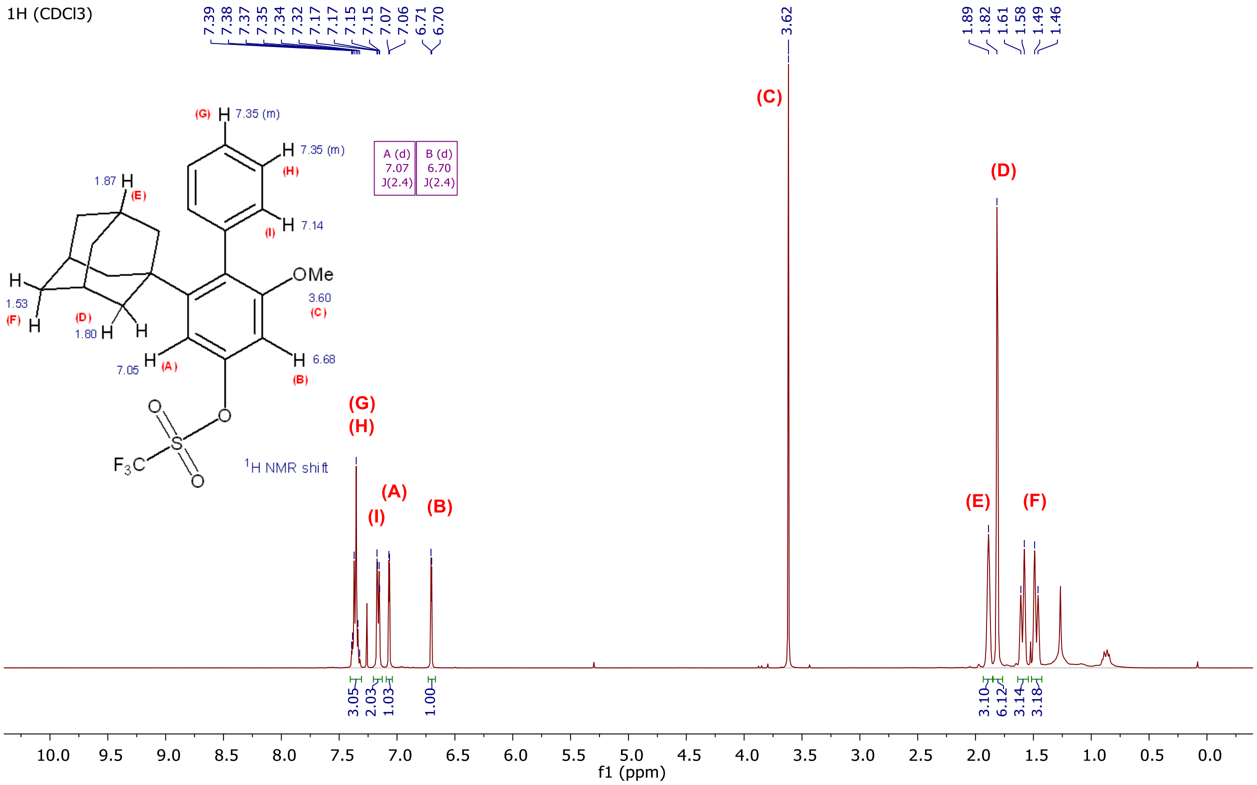
1H (CDCl3)
7.39 7.38 7.37 7.35 7.34 7.32 7.17 7.17 7.15 7.15 7.07 7.06 6.71 6.70
3.62
1.89 1.82 1.61 1.58 1.49 1.46
(G) H 7.35 (m)
H 7.35 (m) (H)
H 7.14 (I)
1.87 H (E)
OMe 3.60 (C)
1.53 (F)
(D) 1.80
7.05 H (A)
H 6.68 (B)
F_3C
^{1}H NMR shift
A (d) 7.07 J(2.4)
B (d) 6.70 J(2.4)
(C)
(D)
(G) (H)
(A)
(I)
(B)
(E)
(F)
3.05 2.03 1.03 1.00
3.10 6.12 3.14 3.18
10.0 9.5 9.0 8.5 8.0 7.5 7.0 6.5 6.0 5.5 5.0 4.5 4.0 3.5 3.0 2.5 2.0 1.5 1.0 0.5 0.0
f1 (ppm)

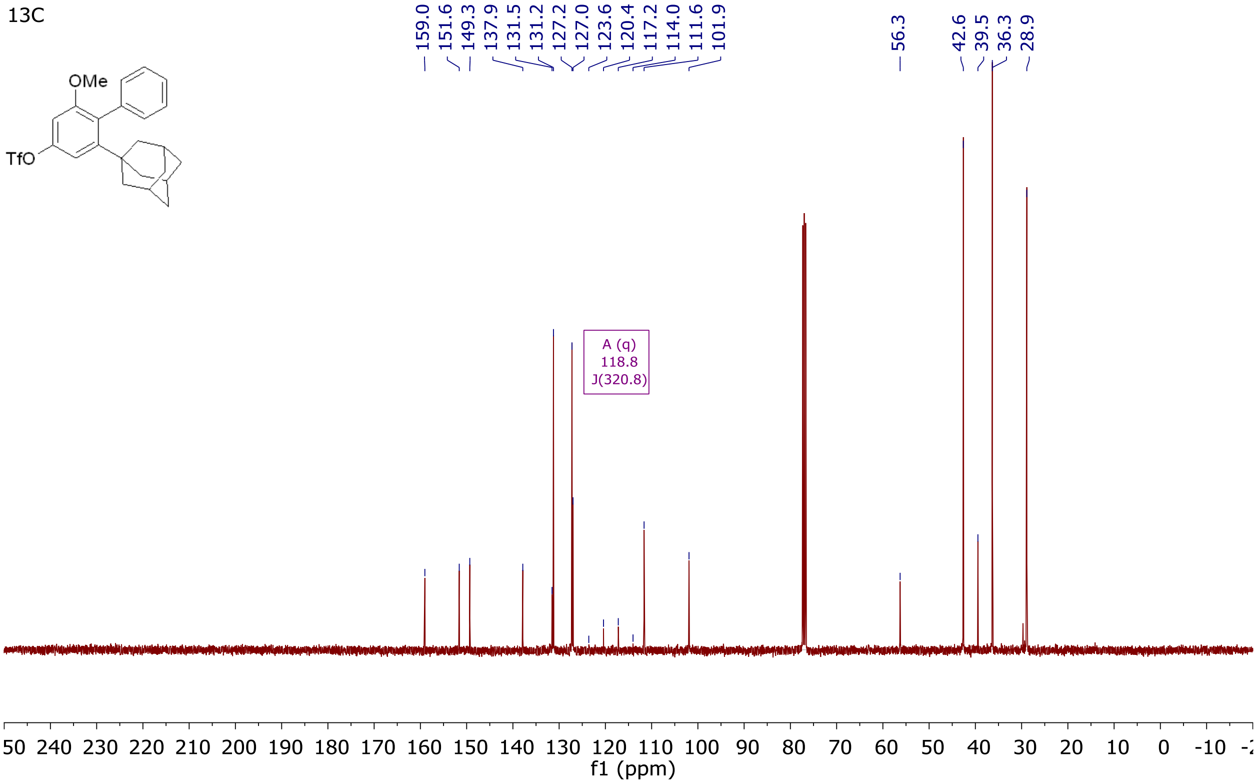
13C
159.0 151.6 149.3 137.9 131.5 131.2 127.2 127.0 123.6 120.4 117.2 114.0 111.6 101.9
56.3
42.6 39.5 36.3 28.9
OMe
TfO
A (q) 118.8 J(320.8)
50 240 230 220 210 200 190 180 170 160 150 140 130 120 110 100 90 80 70 60 50 40 30 20 10 0 -10
f1 (ppm)

19F (CDCl3)

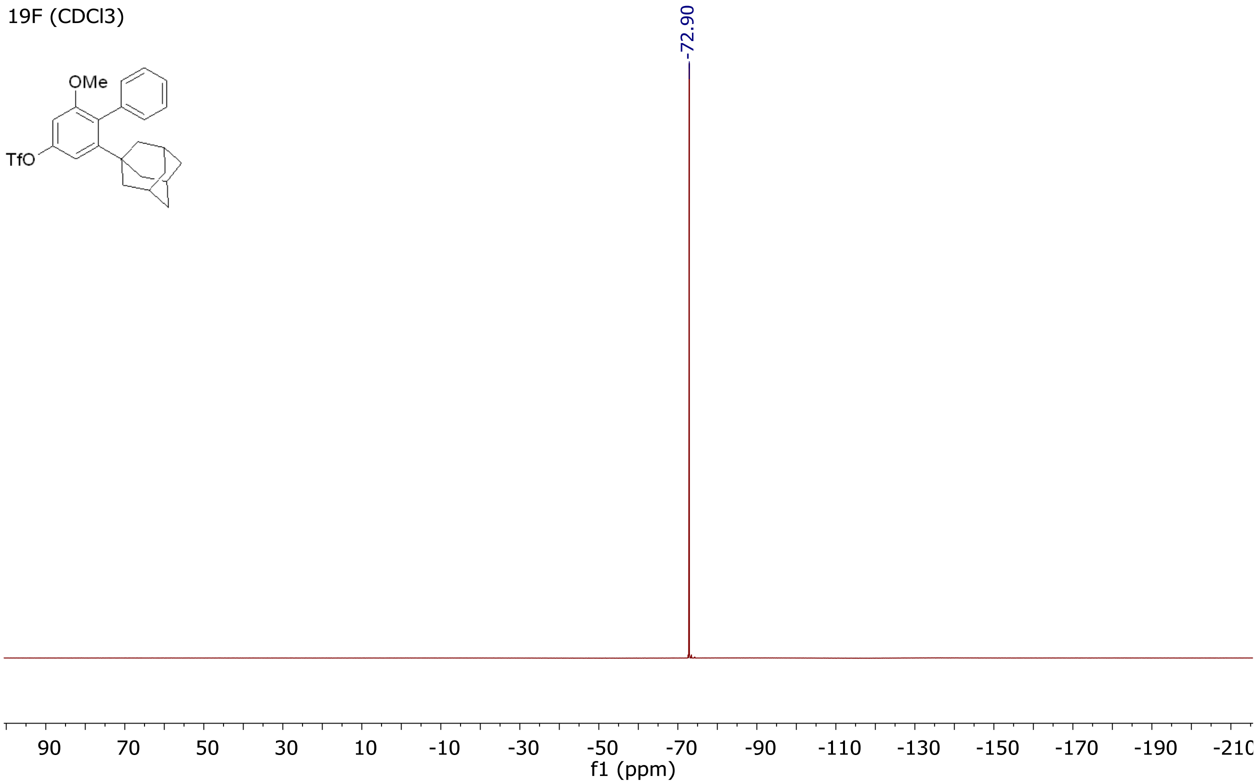
OMe
TfO
-72.90
90
70
50
30
10
-10
-30
-50
-70
-90
-110
-130
-150
-170
-190
-210
f1 (ppm)

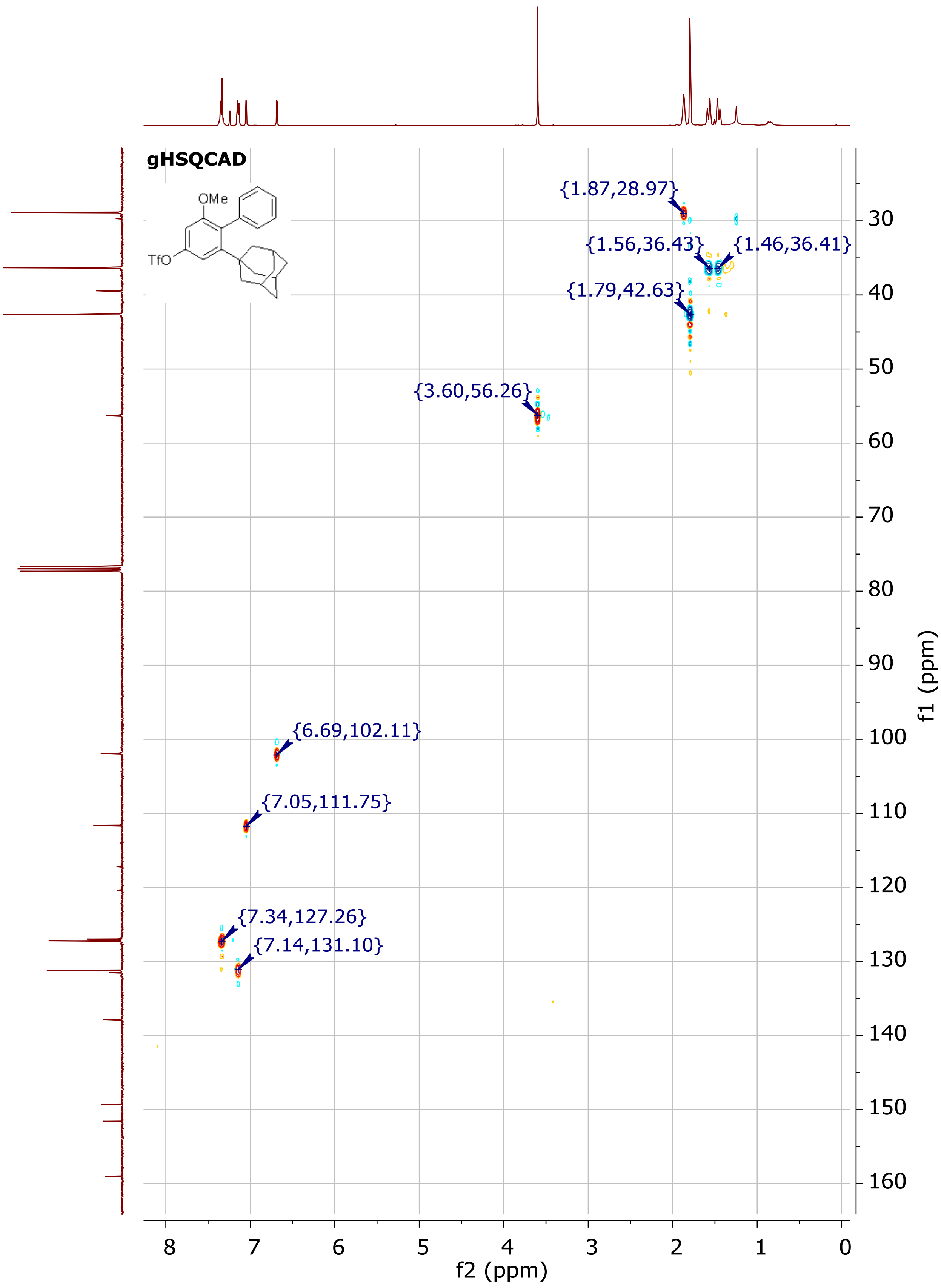
gHSQCAD
OMe
TfO
{1.87,28.97}
{1.56,36.43}
{1.46,36.41}
{1.79,42.63}
{3.60,56.26}
{6.69,102.11}
{7.05,111.75}
{7.34,127.26}
{7.14,131.10}
f2 (ppm)
f1 (ppm)

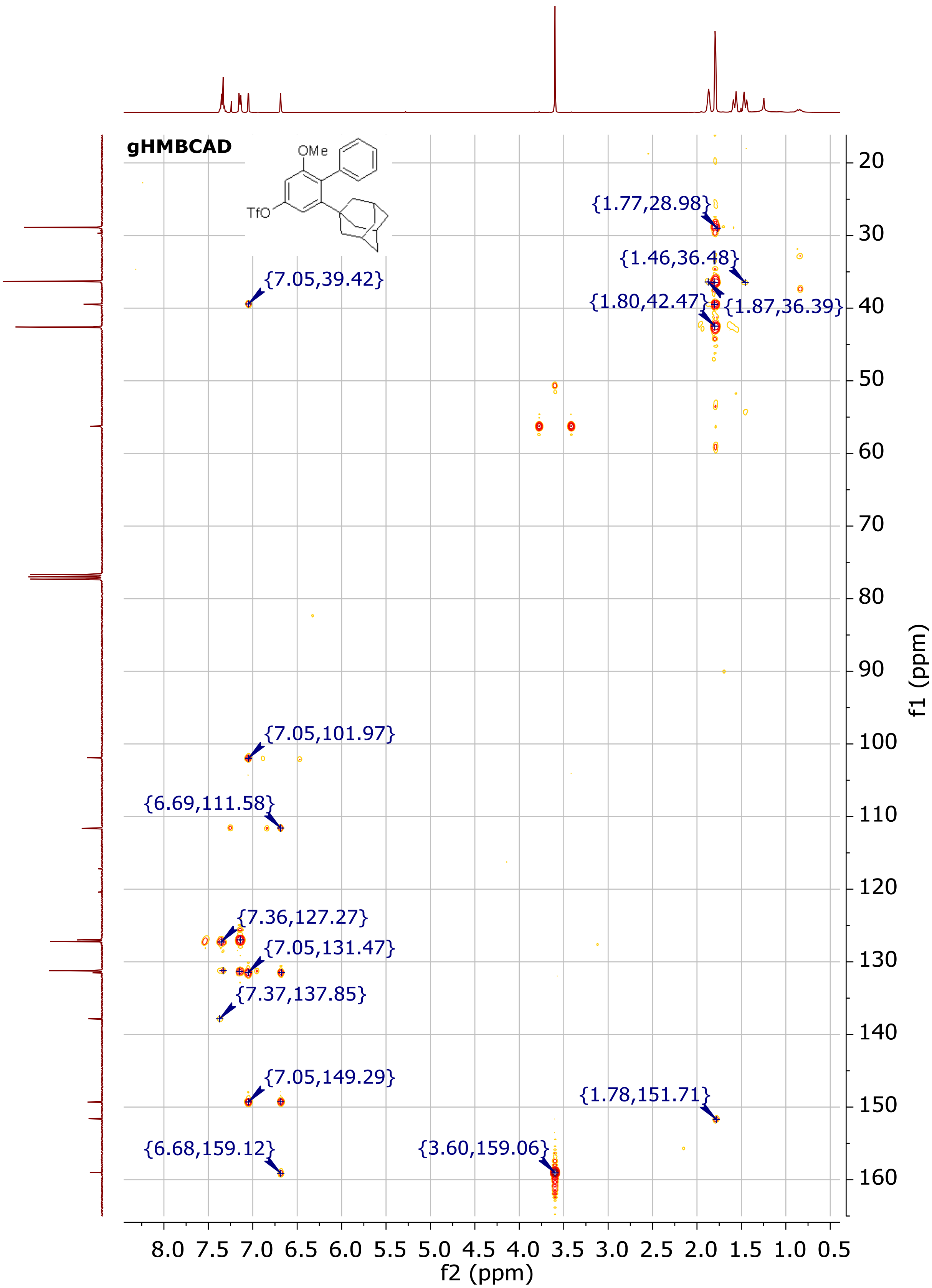
gHMBCAD
OMe
TfO
{1.77,28.98}
{1.46,36.48}
{1.80,42.47}
{1.87,36.39}
{7.05,39.42}
{7.05,101.97}
{6.69,111.58}
{7.36,127.27}
{7.05,131.47}
{7.37,137.85}
{7.05,149.29}
{1.78,151.71}
{6.68,159.12}
{3.60,159.06}
f1 (ppm)
f2 (ppm)

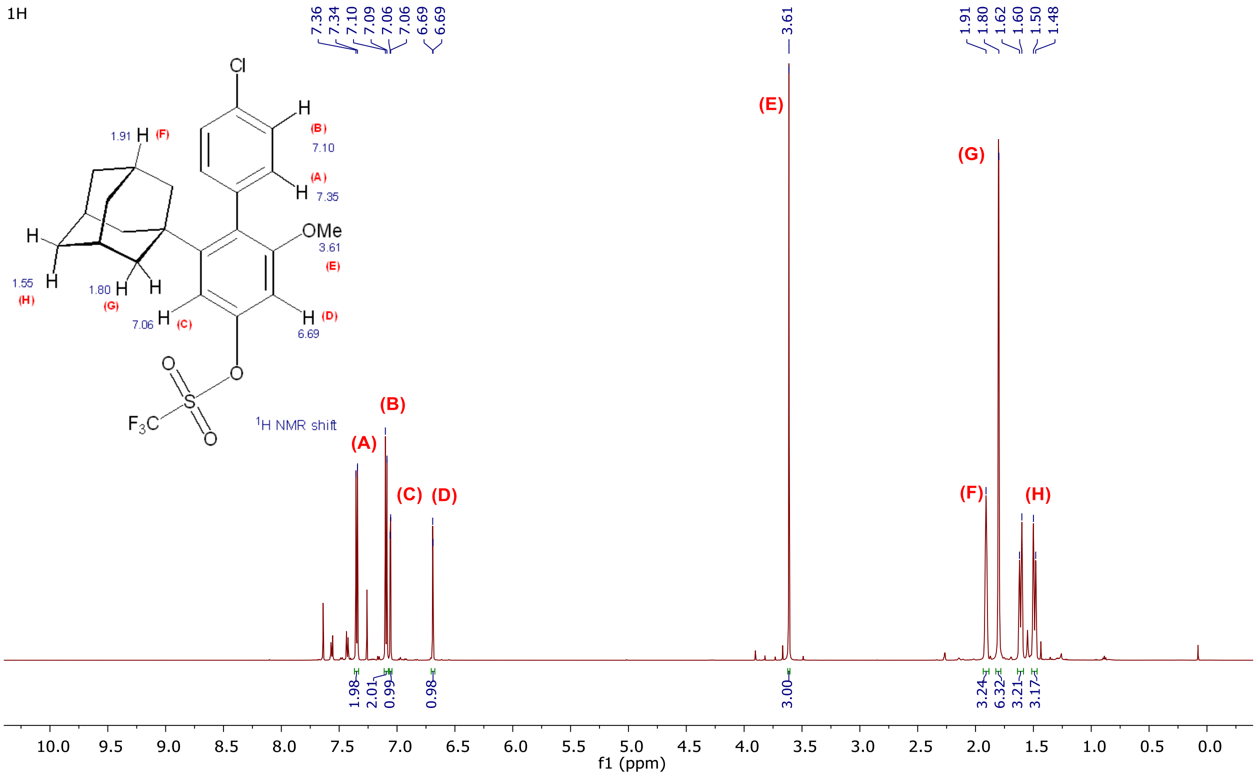
1H
7.36
7.34
7.10
7.09
7.06
7.06
6.69
6.69
3.61
1.91
1.80
1.62
1.60
1.50
1.48
Cl
1.91 H (F)
H (B) 7.10
(A) H 7.35
OMe 3.61 (E)
1.55 H (H)
1.80 H (G)
7.06 H (C)
H (D) 6.69
F3C
O
S
O
O
1H NMR shift
(A)
(B)
(C)
(D)
(E)
(F)
(G)
(H)
1.98
2.01
0.99
0.98
3.00
3.24
6.32
3.21
3.17
10.0 9.5 9.0 8.5 8.0 7.5 7.0 6.5 6.0 5.5 5.0 4.5 4.0 3.5 3.0 2.5 2.0 1.5 1.0 0.5 0.0
f1 (ppm)

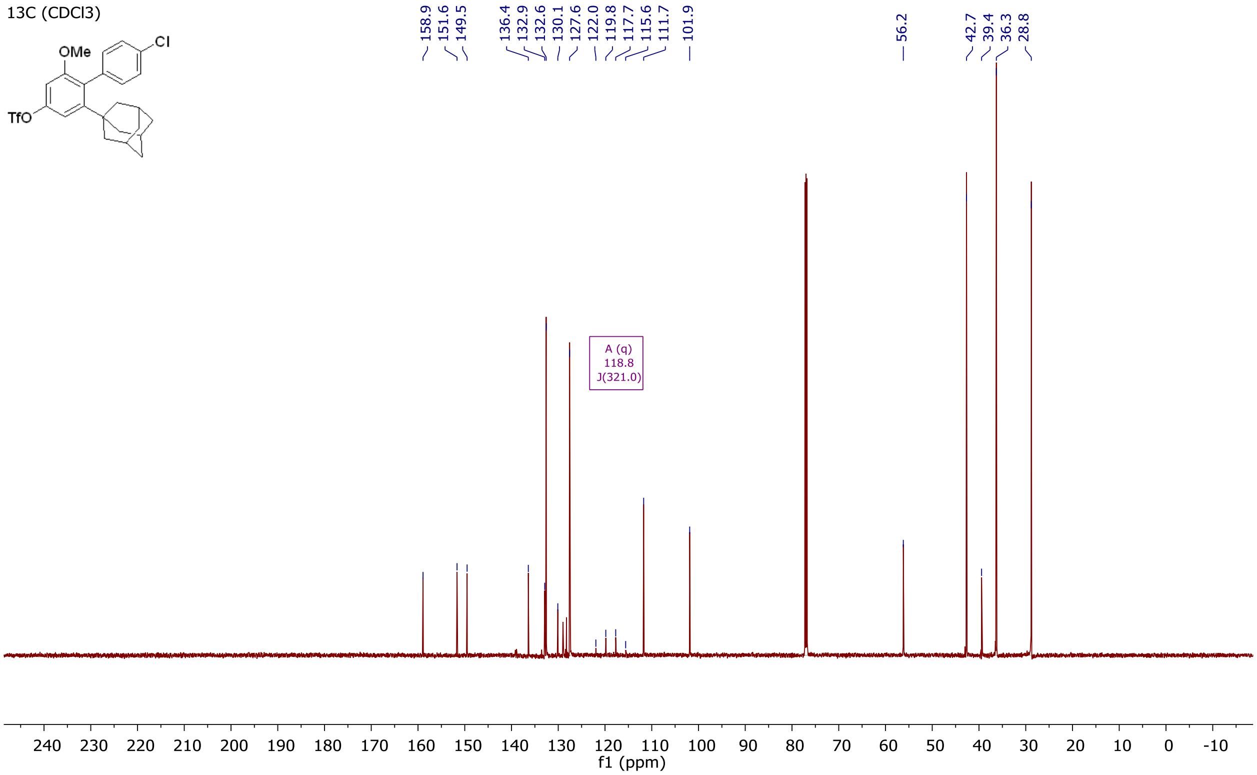
13C (CDCl3)
OMe
Cl
TfO
158.9
151.6
149.5
136.4
132.9
132.6
130.1
127.6
122.0
119.8
117.7
115.6
111.7
101.9
56.2
42.7
39.4
36.3
28.8
A (q)
118.8
J(321.0)
240 230 220 210 200 190 180 170 160 150 140 130 120 110 100 90 80 70 60 50 40 30 20 10 0 -10
f1 (ppm)

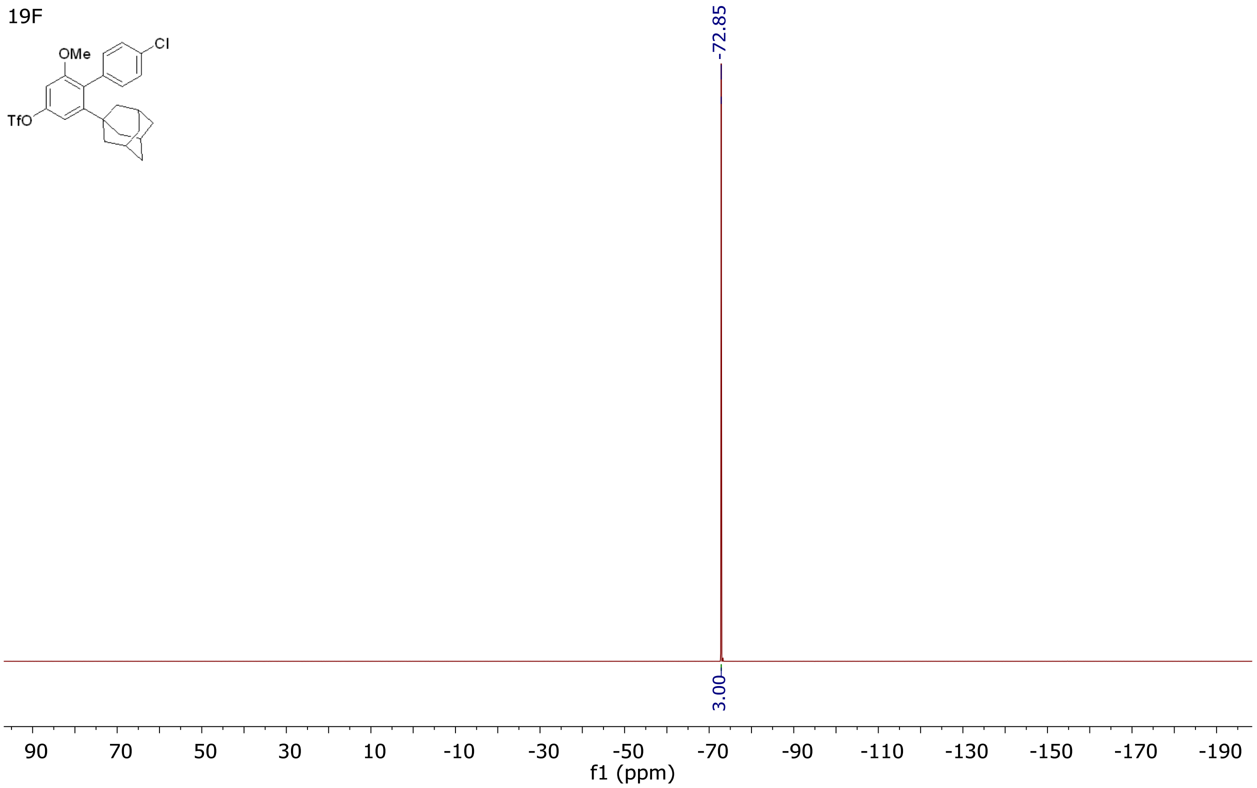

19F
OMe
Cl
TfO
-72.85
3.00
90
70
50
30
10
-10
-30
-50
-70
-90
-110
-130
-150
-170
-190
f1 (ppm)

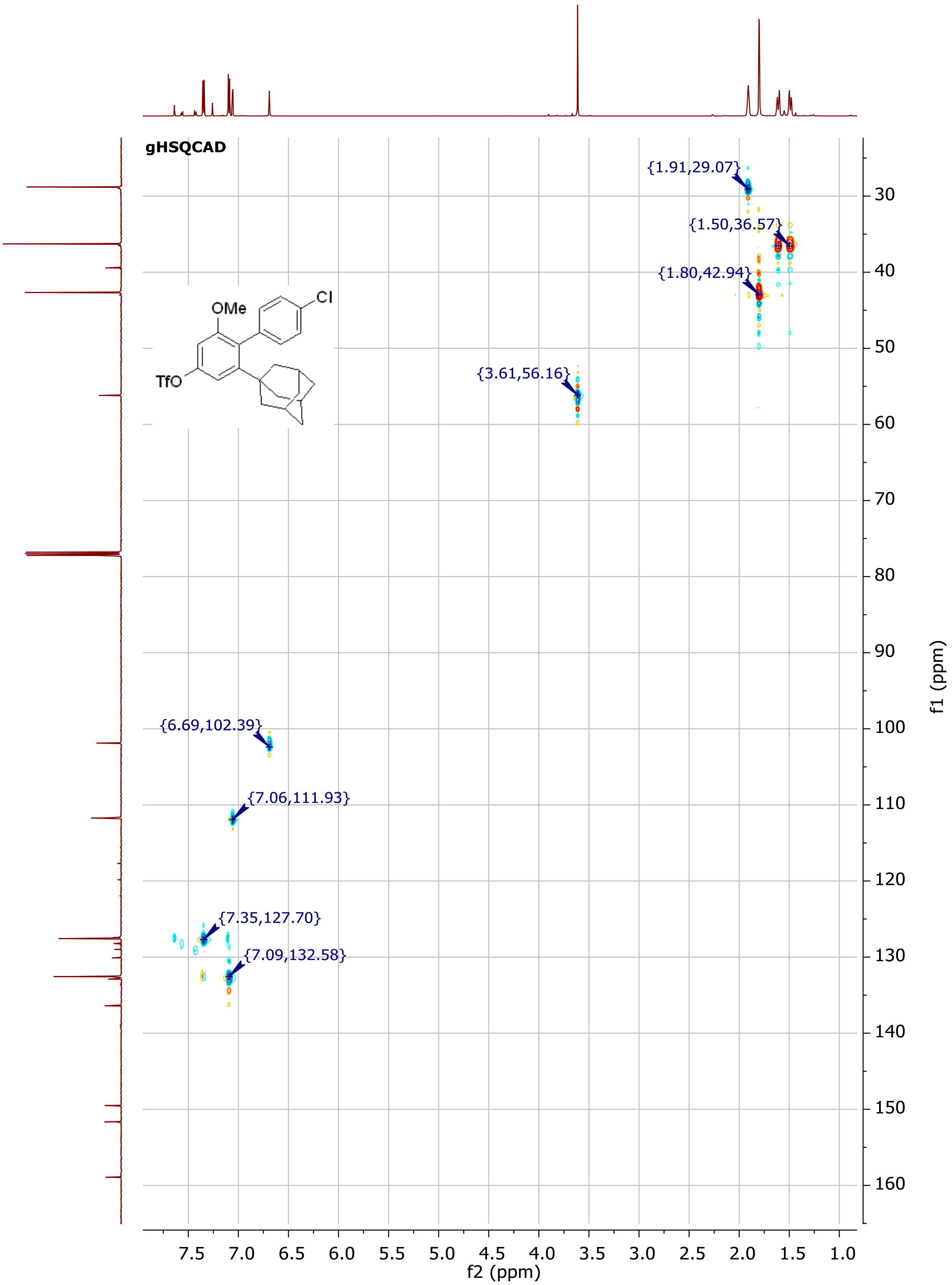

gHSQCAD
{1.91,29.07}
{1.50,36.57}
{1.80,42.94}
{3.61,56.16}
{6.69,102.39}
{7.06,111.93}
{7.35,127.70}
{7.09,132.58}
OMe
Cl
TfO
f2 (ppm)
f1 (ppm)

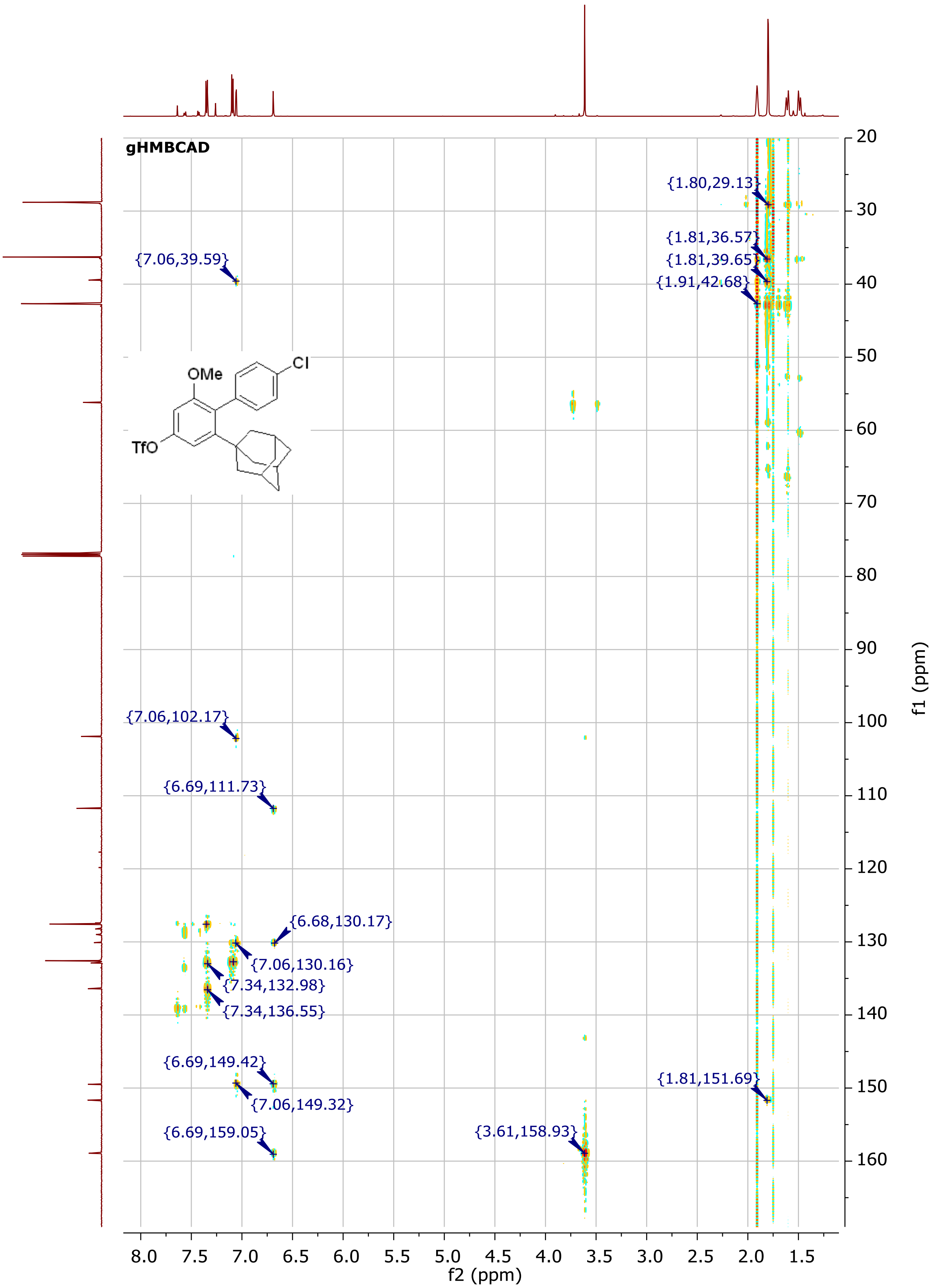
gHMBCAD
{1.80,29.13}
{1.81,36.57}
{1.81,39.65}
{1.91,42.68}
{7.06,39.59}
OMe
Cl
TfO
{7.06,102.17}
{6.69,111.73}
{6.68,130.17}
{7.06,130.16}
{7.34,132.98}
{7.34,136.55}
{6.69,149.42}
{7.06,149.32}
{1.81,151.69}
{6.69,159.05}
{3.61,158.93}
f2 (ppm)
f1 (ppm)

1H (CDCl3)

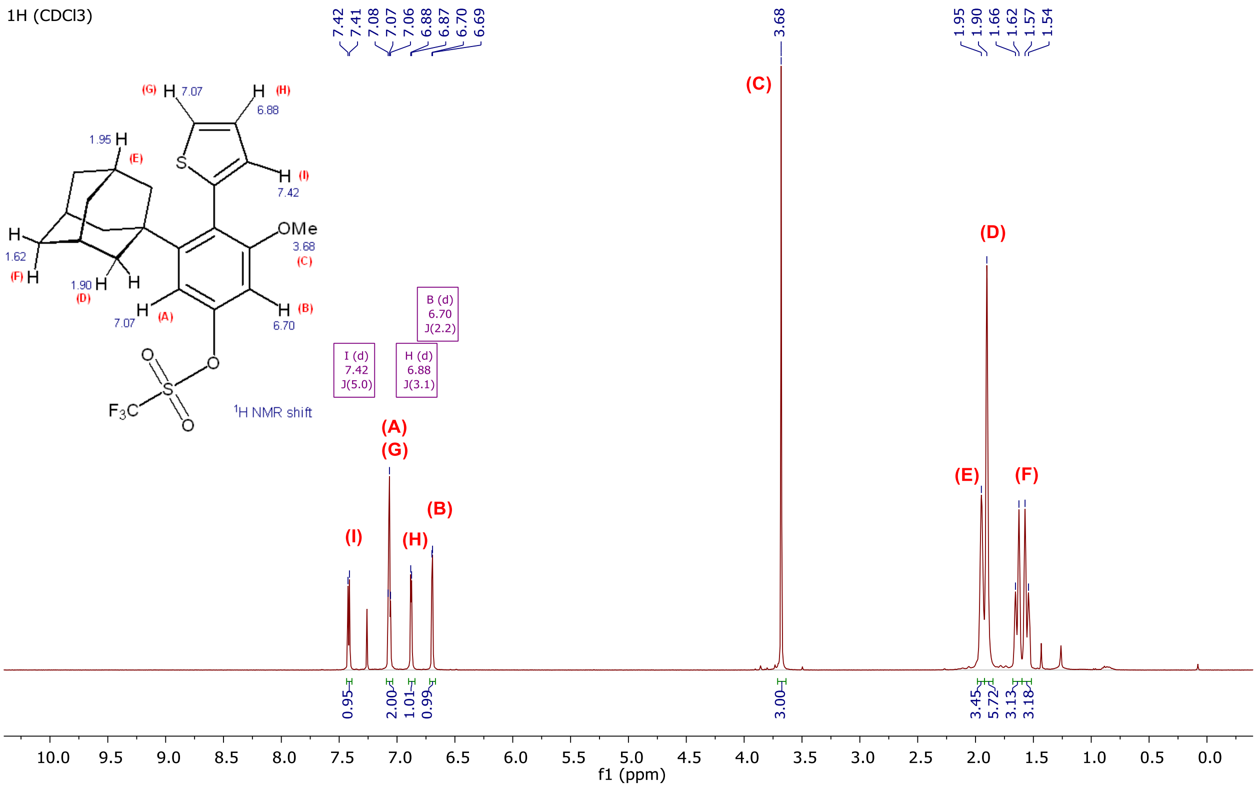

7.42
7.41
7.08
7.07
7.06
6.88
6.87
6.70
6.69
3.68
1.95
1.90
1.66
1.62
1.57
1.54
(C)
(D)
(E)
(F)
(A)
(G)
(I)
(H)
(B)
I (d) 7.42 J(5.0)
H (d) 6.88 J(3.1)
B (d) 6.70 J(2.2)
1H NMR shift
OMe
F3C
0.95
2.00
1.01
0.99
3.00
3.45
5.72
3.13
3.18
f1 (ppm)

13C

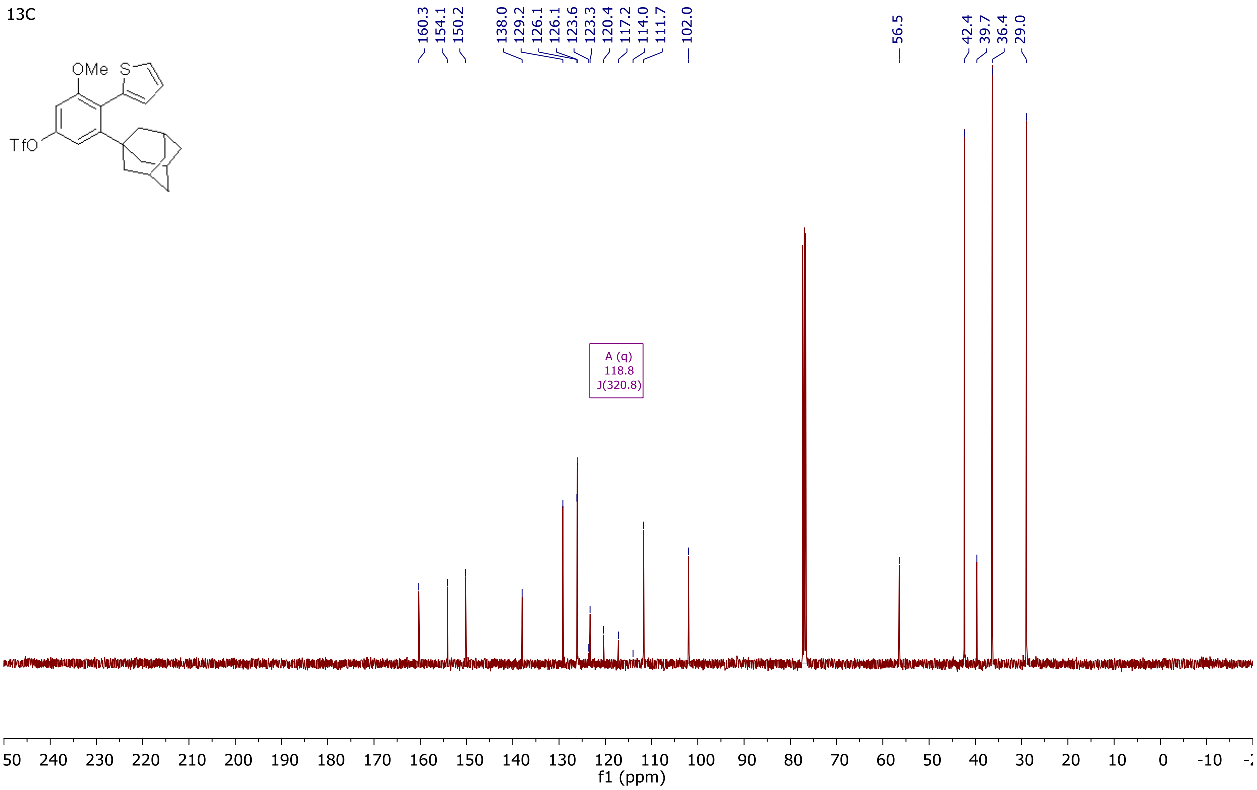

160.3
154.1
150.2
138.0
129.2
126.1
126.1
123.6
123.3
120.4
117.2
114.0
111.7
102.0
56.5
42.4
39.7
36.4
29.0
OMe
TfO
A (q) 118.8 J(320.8)
f1 (ppm)

19F (CDCl3)

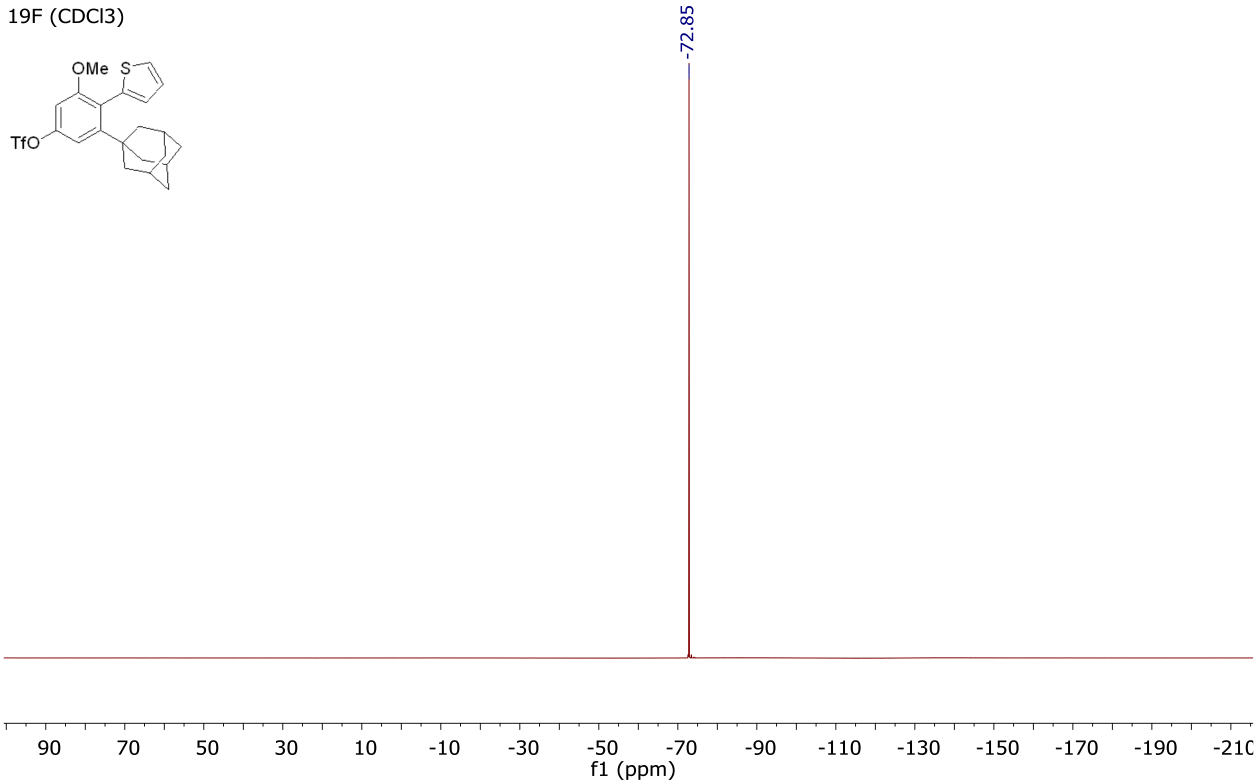

OMe
S
TfO
-72.85
90
70
50
30
10
-10
-30
-50
-70
-90
-110
-130
-150
-170
-190
-210
f1 (ppm)

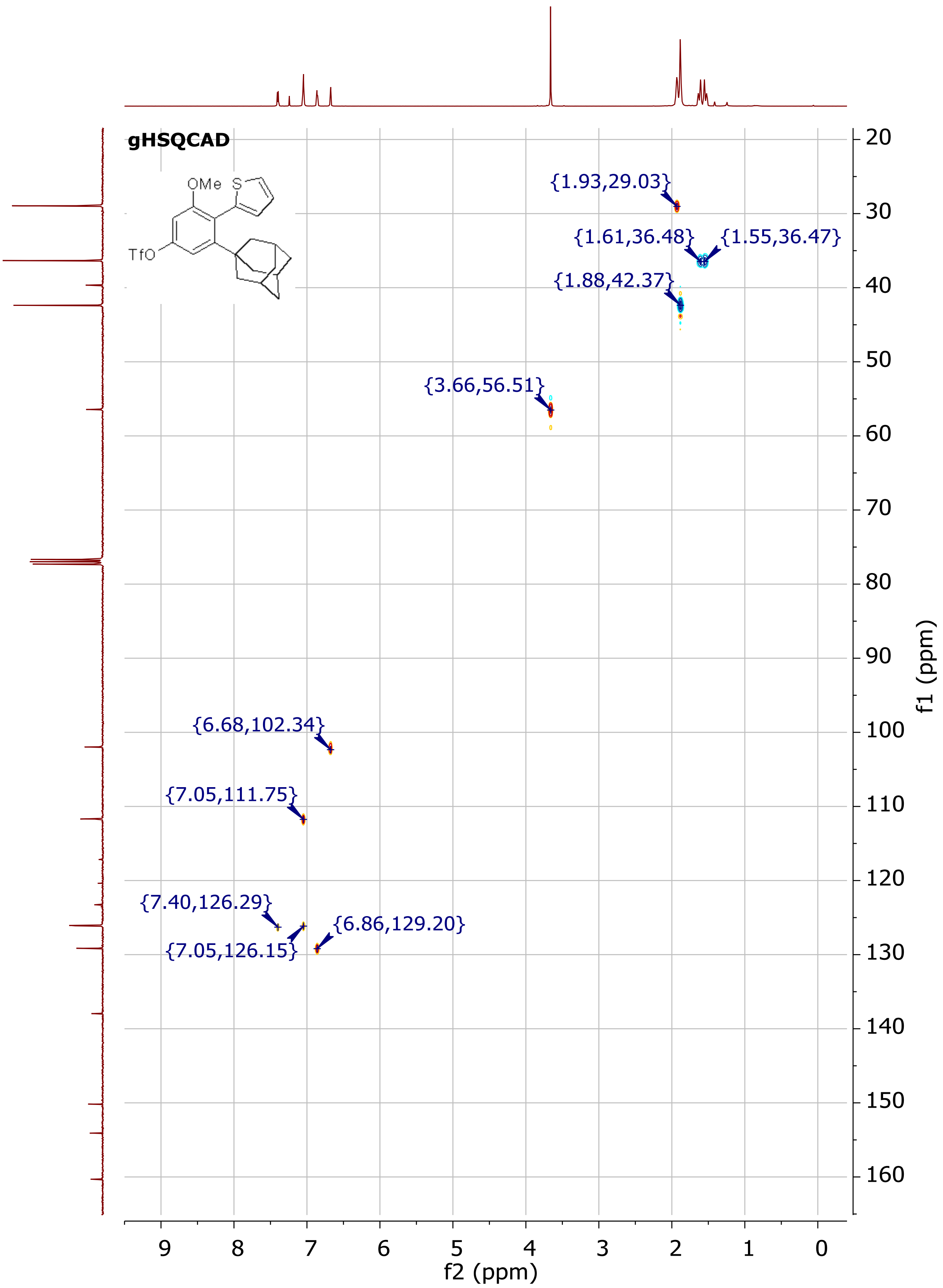
gHSQCAD
OMe
S
TfO
{1.93,29.03}
{1.61,36.48}
{1.55,36.47}
{1.88,42.37}
{3.66,56.51}
{6.68,102.34}
{7.05,111.75}
{7.40,126.29}
{7.05,126.15}
{6.86,129.20}
f2 (ppm)
f1 (ppm)

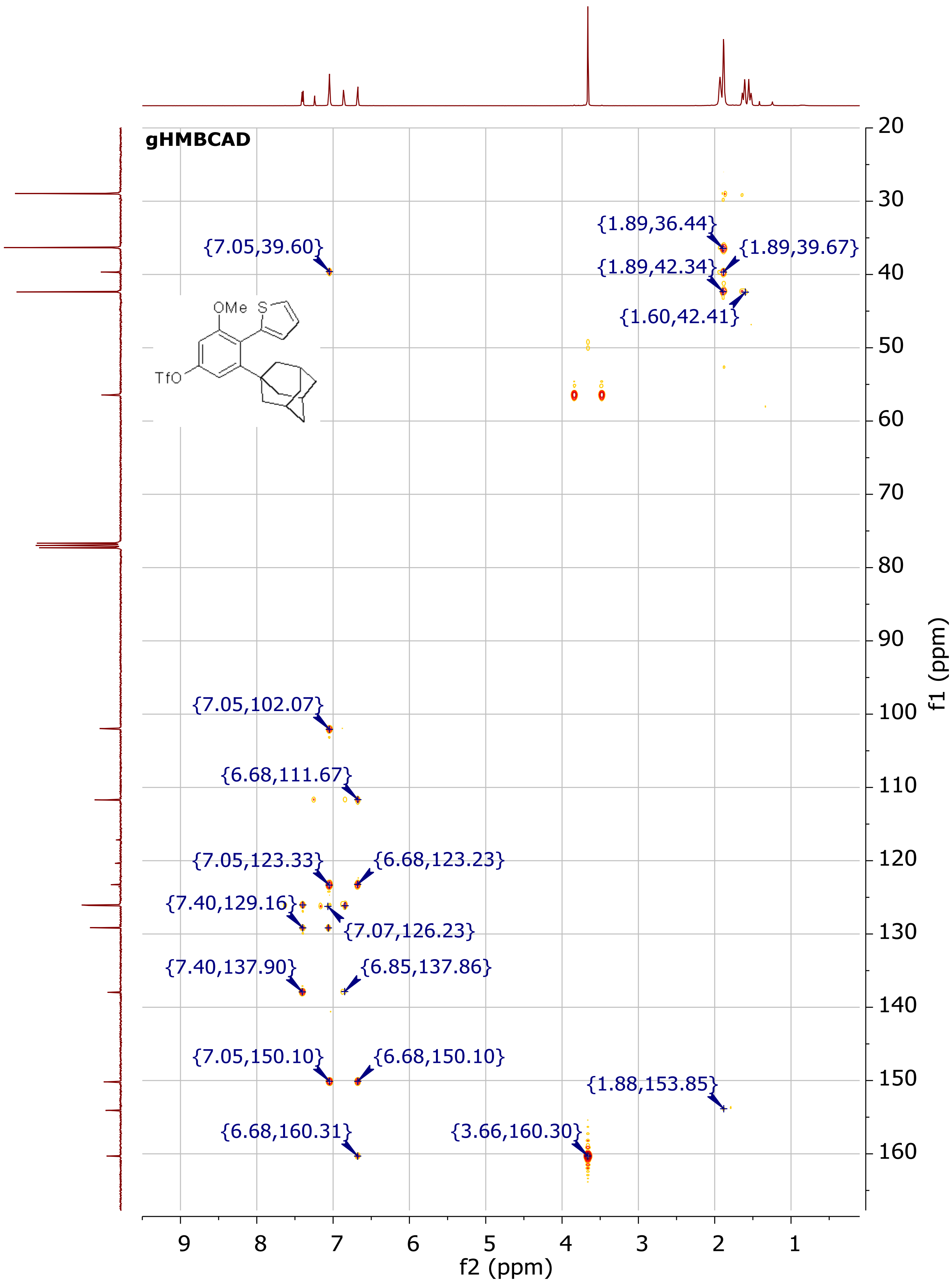
gHMBCAD
OMe
S
TfO
{7.05,39.60}
{1.89,36.44}
{1.89,39.67}
{1.89,42.34}
{1.60,42.41}
{7.05,102.07}
{6.68,111.67}
{7.05,123.33}
{6.68,123.23}
{7.40,129.16}
{7.07,126.23}
{7.40,137.90}
{6.85,137.86}
{7.05,150.10}
{6.68,150.10}
{1.88,153.85}
{6.68,160.31}
{3.66,160.30}
f1 (ppm)
f2 (ppm)

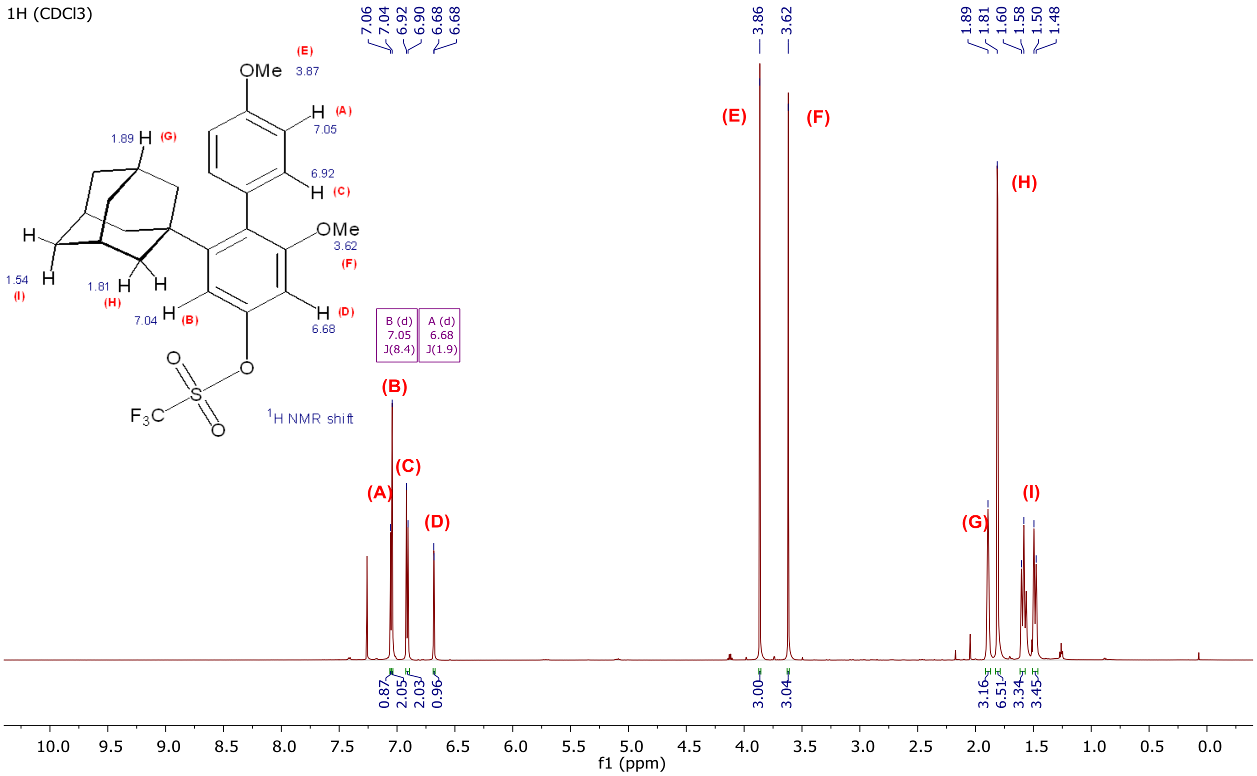
1H (CDCl3)
7.06
7.04
6.92
6.90
6.68
6.68
3.86
3.62
1.89
1.81
1.60
1.58
1.50
1.48
OMe
(E)
3.87
H (A)
7.05
1.89 H (G)
6.92
H (C)
OMe
3.62
(F)
H
1.54 H
(I)
1.81 H H
(H)
7.04 H (B)
H (D)
6.68
O
S
O
F3C
O
1H NMR shift
B (d)
7.05
J(8.4)
A (d)
6.68
J(1.9)
(A)
(B)
(C)
(D)
(E)
(F)
(G)
(H)
(I)
0.87
2.05
2.03
0.96
3.00
3.04
3.16
6.51
3.34
3.45
10.0
9.5
9.0
8.5
8.0
7.5
7.0
6.5
6.0
5.5
5.0
4.5
4.0
3.5
3.0
2.5
2.0
1.5
1.0
0.5
0.0
f1 (ppm)

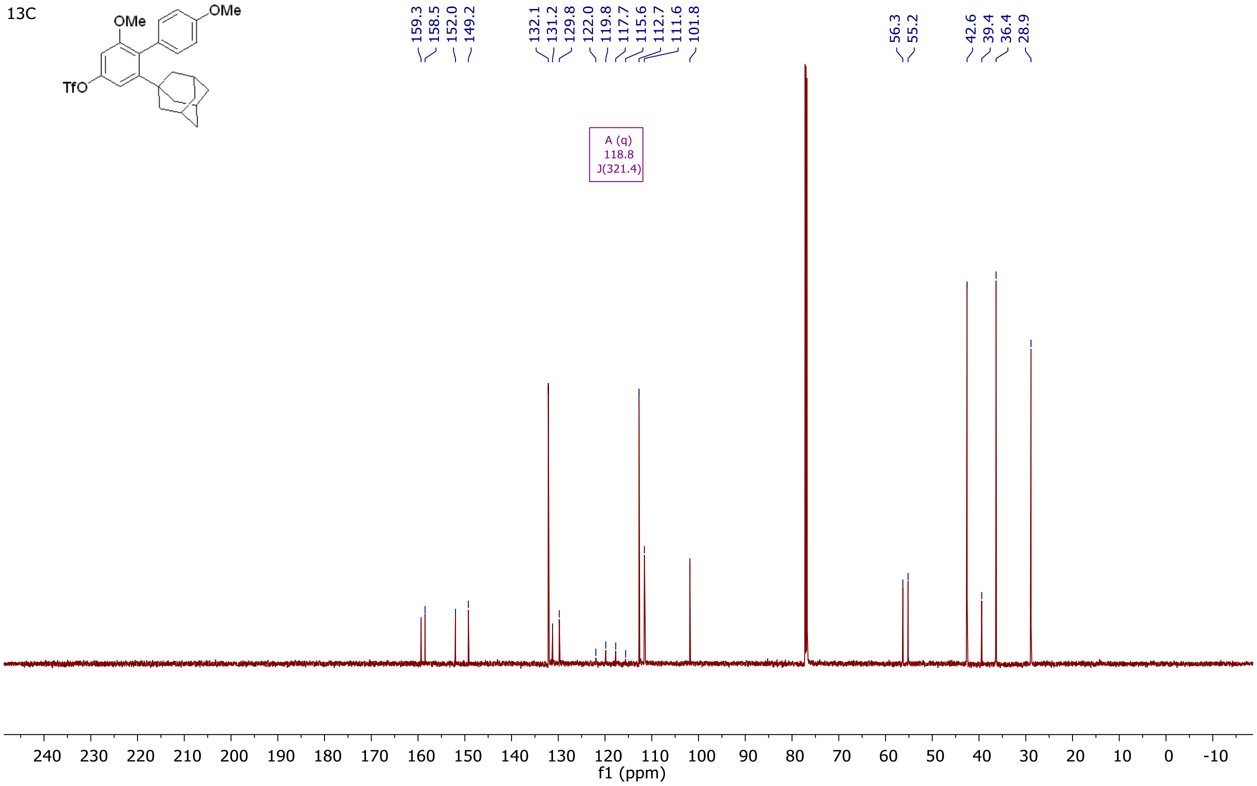
13C
OMe
OMe
TfO
159.3
158.5
152.0
149.2
132.1
131.2
129.8
122.0
119.8
117.7
115.6
112.7
111.6
101.8
56.3
55.2
42.6
39.4
36.4
28.9
A (q)
118.8
J(321.4)
240
230
220
210
200
190
180
170
160
150
140
130
120
110
100
90
80
70
60
50
40
30
20
10
0
-10
f1 (ppm)

19F (CDCl3)

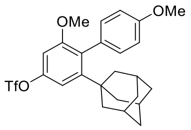

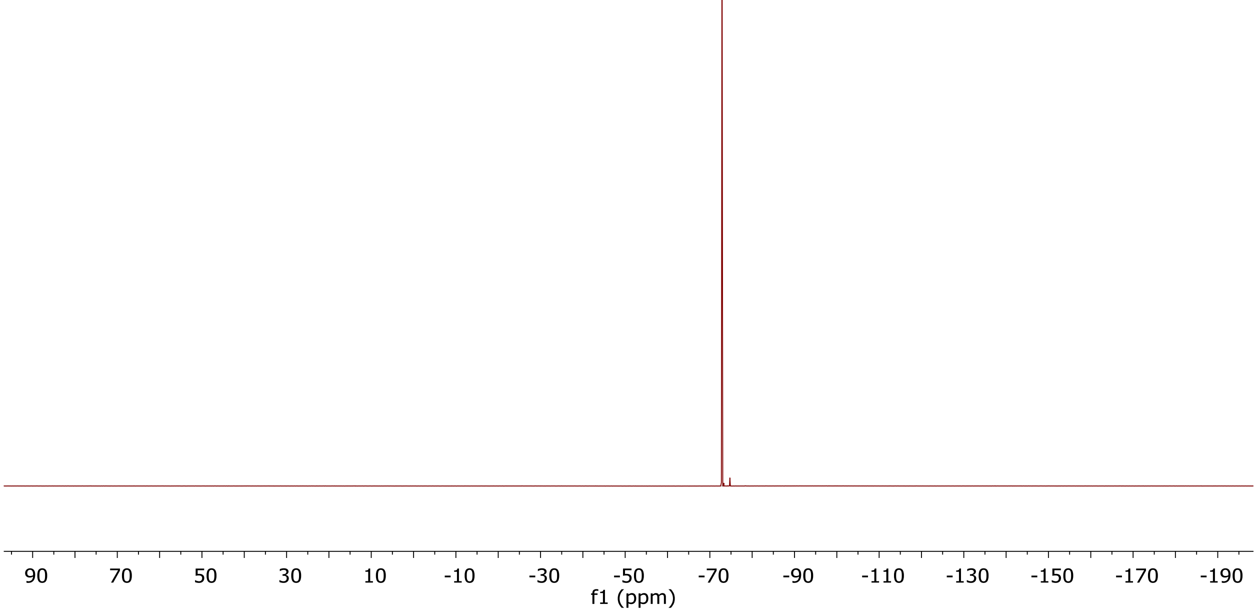

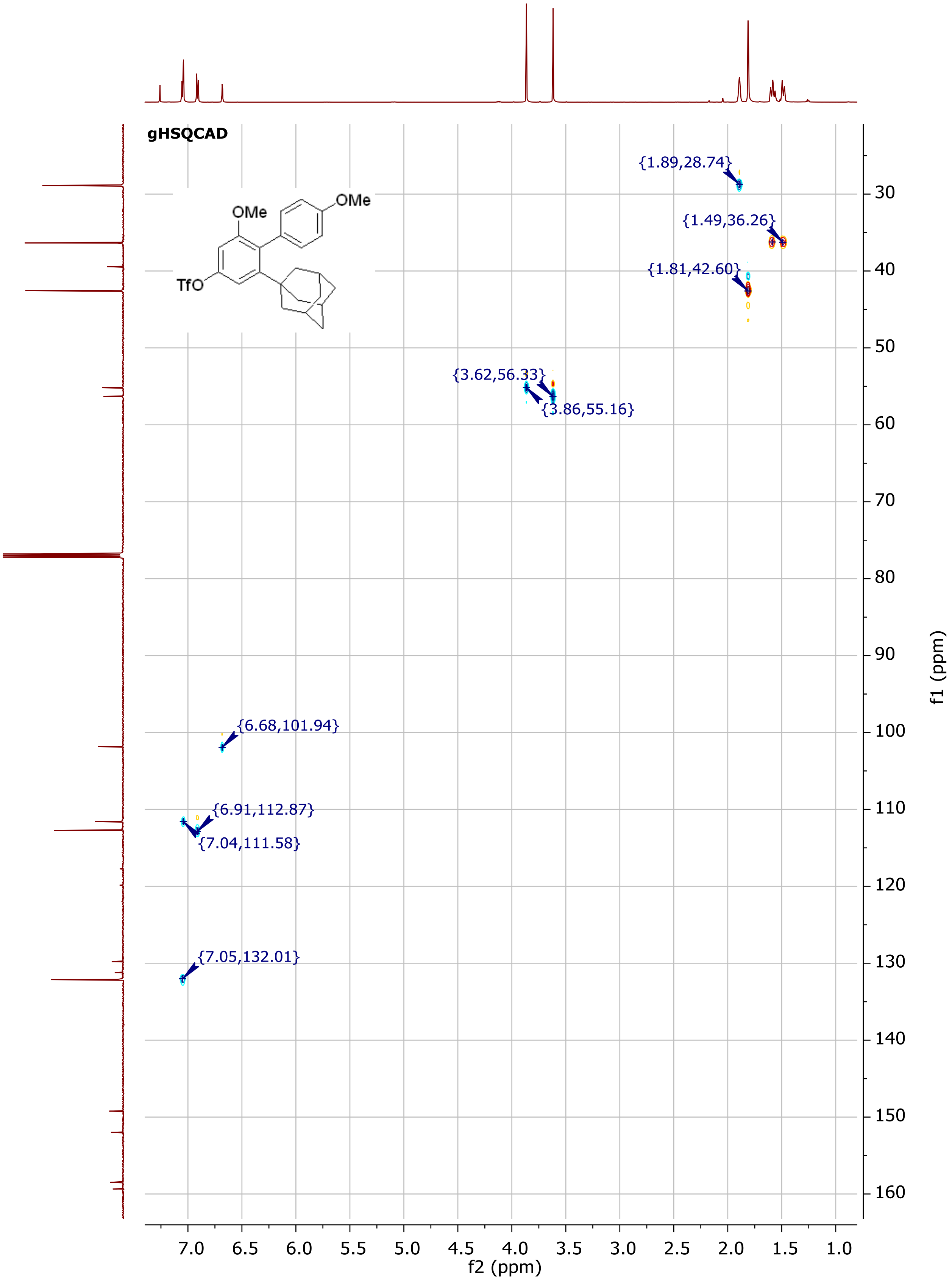
gHSQCAD
OMe
OMe
TfO
{1.89,28.74}
{1.49,36.26}
{1.81,42.60}
{3.62,56.33}
{3.86,55.16}
{6.68,101.94}
{6.91,112.87}
{7.04,111.58}
{7.05,132.01}
f2 (ppm)
f1 (ppm)

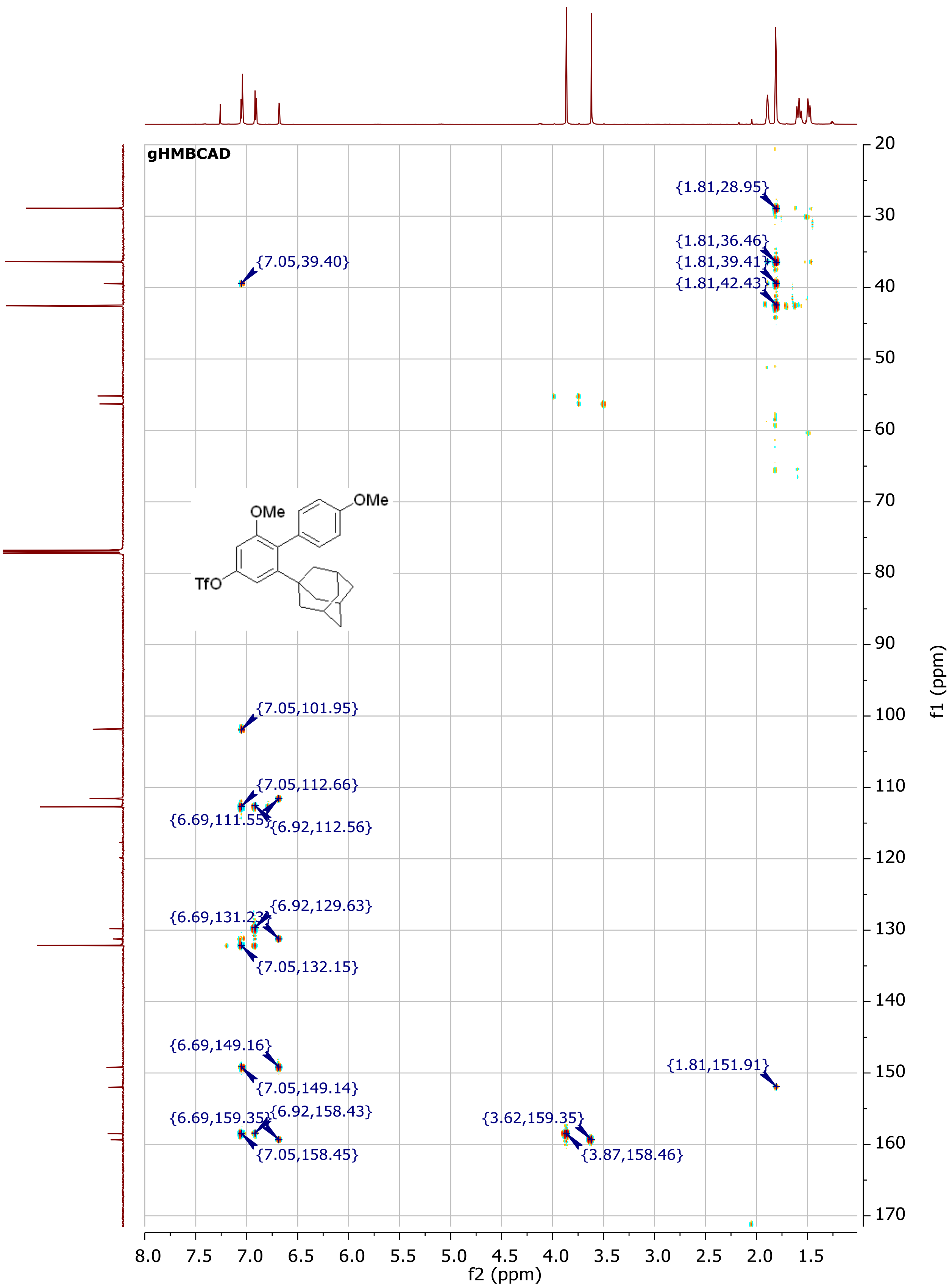
gHMBCAD
{1.81,28.95}
{1.81,36.46}
{1.81,39.41}
{1.81,42.43}
{7.05,39.40}
{7.05,101.95}
{7.05,112.66}
{6.69,111.55}
{6.92,112.56}
{6.92,129.63}
{6.69,131.23}
{7.05,132.15}
{6.69,149.16}
{7.05,149.14}
{1.81,151.91}
{6.92,158.43}
{6.69,159.35}
{7.05,158.45}
{3.62,159.35}
{3.87,158.46}
OMe
OMe
TfO
f1 (ppm)
f2 (ppm)

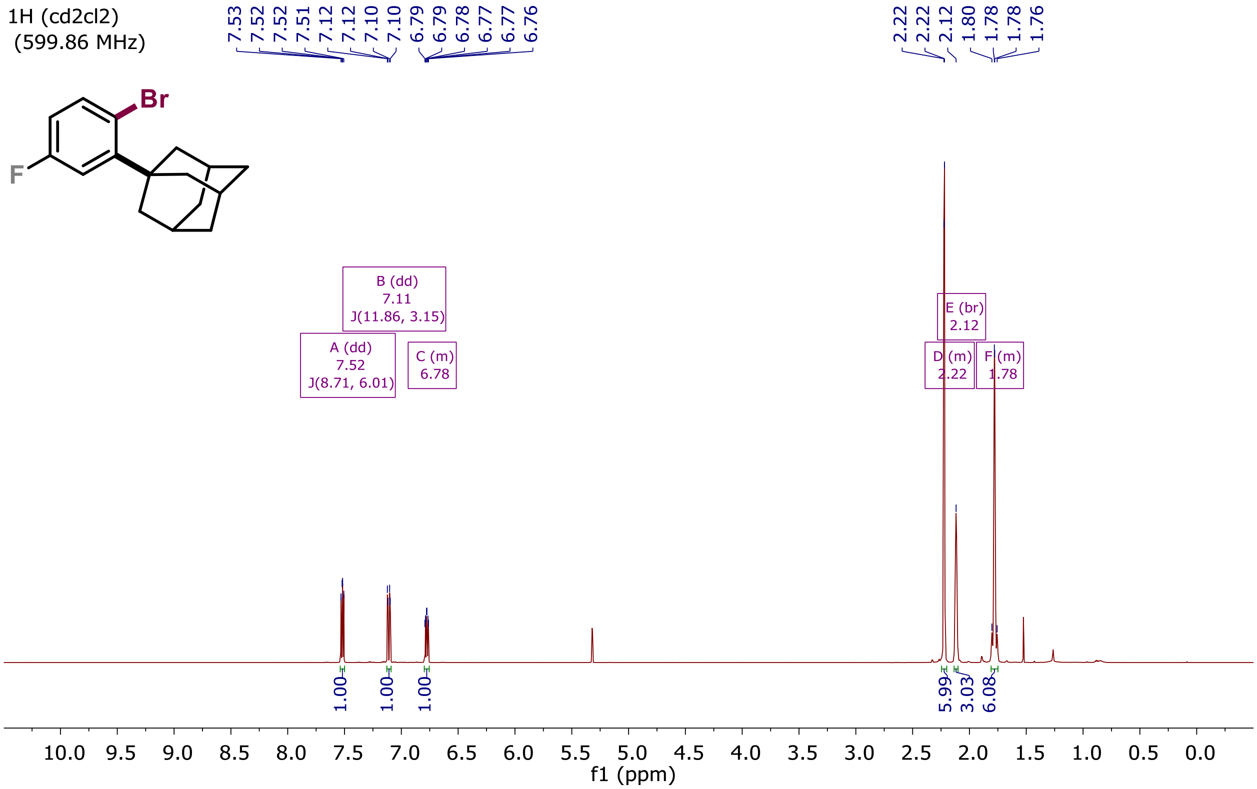
1H (cd2cl2)
(599.86 MHz)
Br
F
7.53 7.52 7.52 7.51 7.12 7.12 7.10 7.10 6.79 6.79 6.78 6.77 6.77 6.76
2.22 2.22 2.12 1.80 1.78 1.78 1.76
B (dd)
7.11
J(11.86, 3.15)
A (dd)
7.52
J(8.71, 6.01)
C (m)
6.78
E (br)
2.12
D (m)
2.22
F (m)
1.78
1.00
1.00
1.00
5.99
3.03
6.08
10.0 9.5 9.0 8.5 8.0 7.5 7.0 6.5 6.0 5.5 5.0 4.5 4.0 3.5 3.0 2.5 2.0 1.5 1.0 0.5 0.0
f1 (ppm)

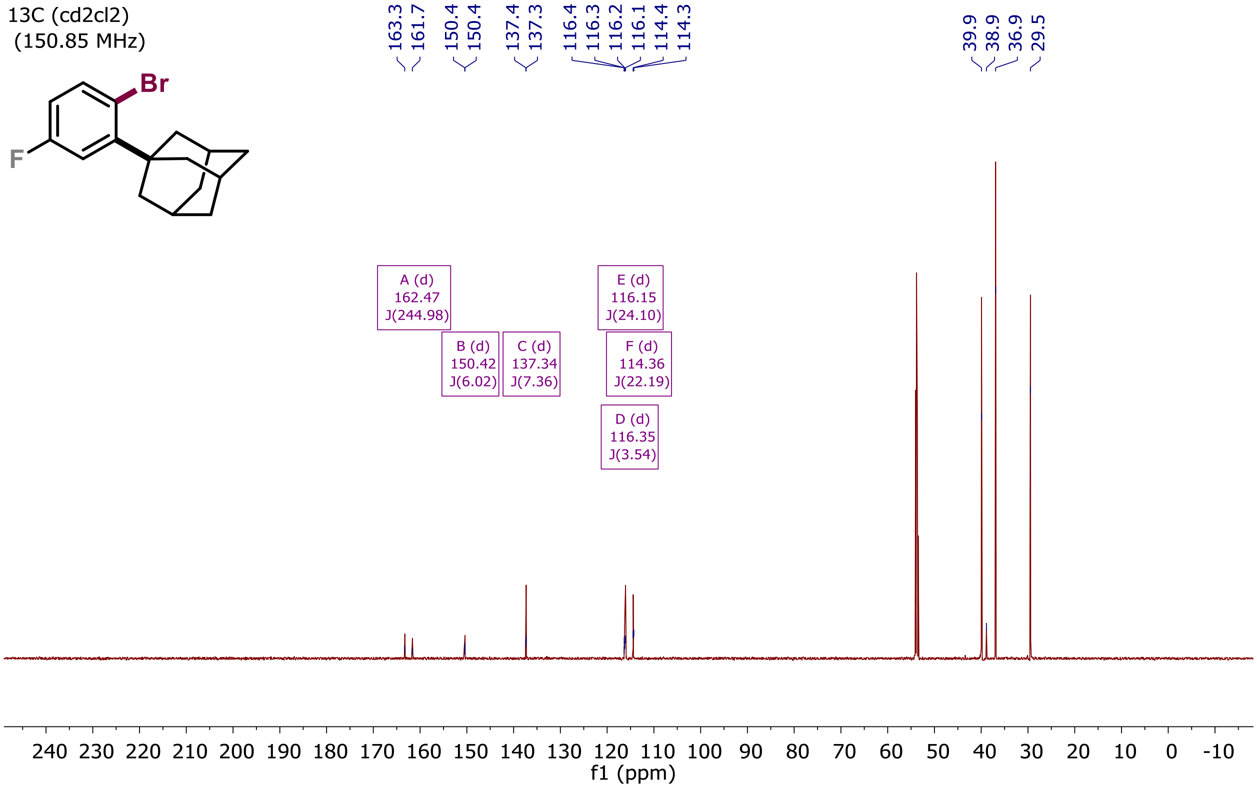
13C (cd2cl2)
(150.85 MHz)
Br
F
163.3 161.7 150.4 150.4 137.4 137.3 116.4 116.3 116.2 116.1 114.4 114.3
39.9 38.9 36.9 29.5
A (d)
162.47
J(244.98)
B (d)
150.42
J(6.02)
C (d)
137.34
J(7.36)
E (d)
116.15
J(24.10)
F (d)
114.36
J(22.19)
D (d)
116.35
J(3.54)
240 230 220 210 200 190 180 170 160 150 140 130 120 110 100 90 80 70 60 50 40 30 20 10 0 -10
f1 (ppm)

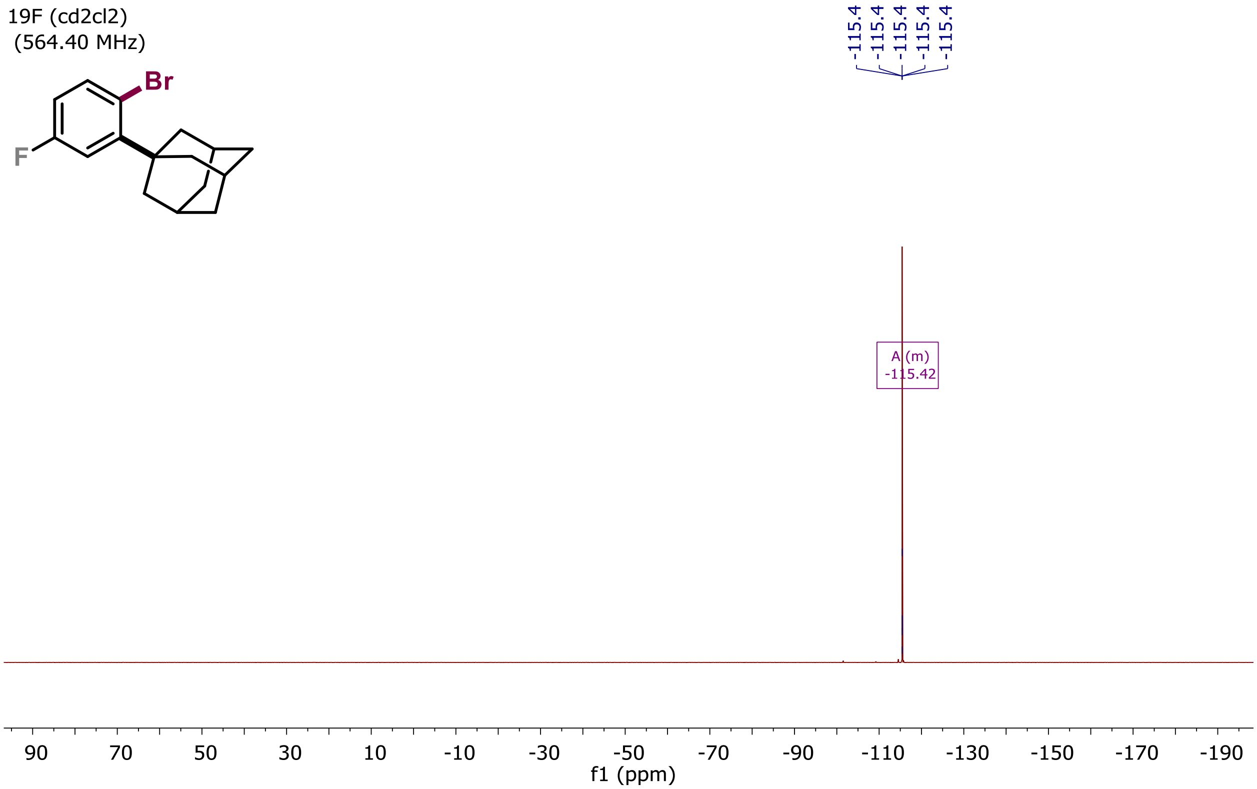
19F (cd2cl2)
(564.40 MHz)
Br
F
-115.4
-115.4
-115.4
-115.4
-115.4
A (m)
-115.42
90 70 50 30 10 -10 -30 -50 -70 -90 -110 -130 -150 -170 -190
f1 (ppm)

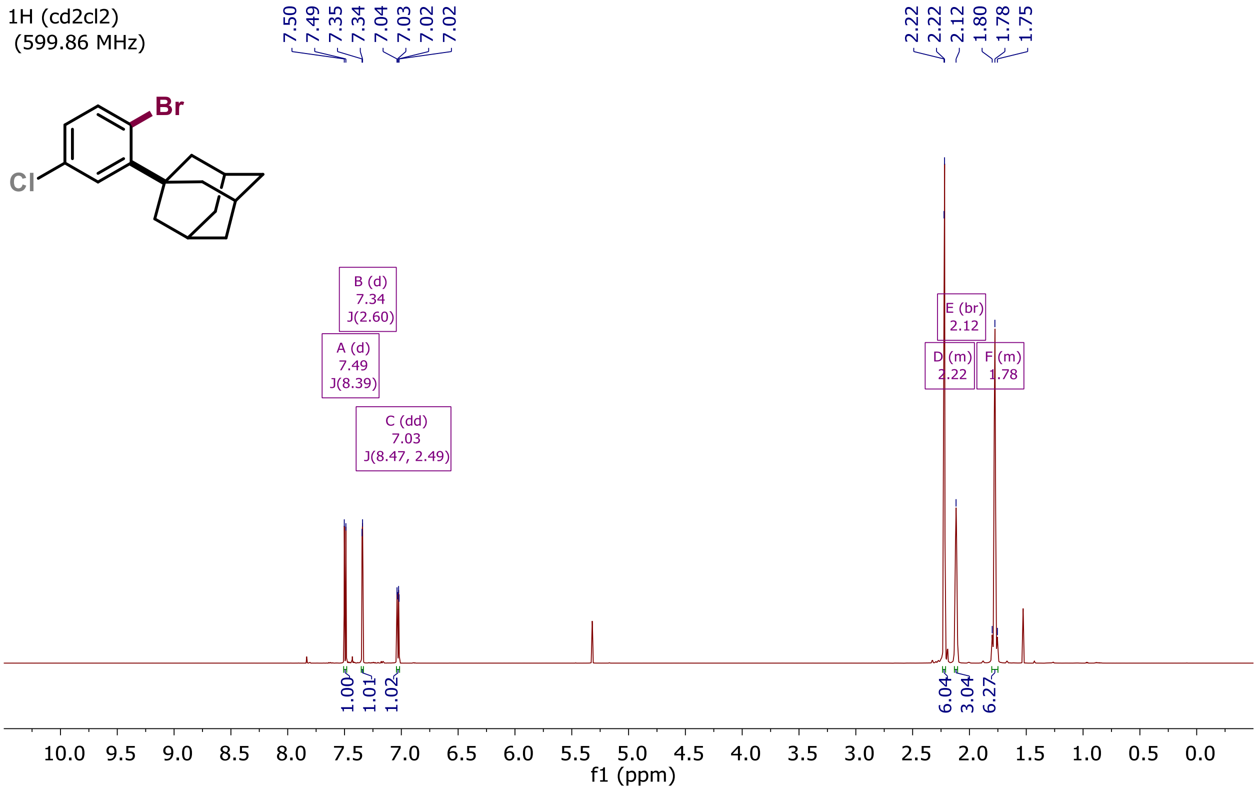
1H (cd2cl2)
(599.86 MHz)
Br
Cl
7.50
7.49
7.35
7.34
7.04
7.03
7.02
7.02
2.22
2.22
2.12
1.80
1.78
1.75
B (d)
7.34
J(2.60)
A (d)
7.49
J(8.39)
C (dd)
7.03
J(8.47, 2.49)
E (br)
2.12
D (m)
2.22
F (m)
1.78
1.00
1.01
1.02
6.04
3.04
6.27
10.0 9.5 9.0 8.5 8.0 7.5 7.0 6.5 6.0 5.5 5.0 4.5 4.0 3.5 3.0 2.5 2.0 1.5 1.0 0.5 0.0
f1 (ppm)

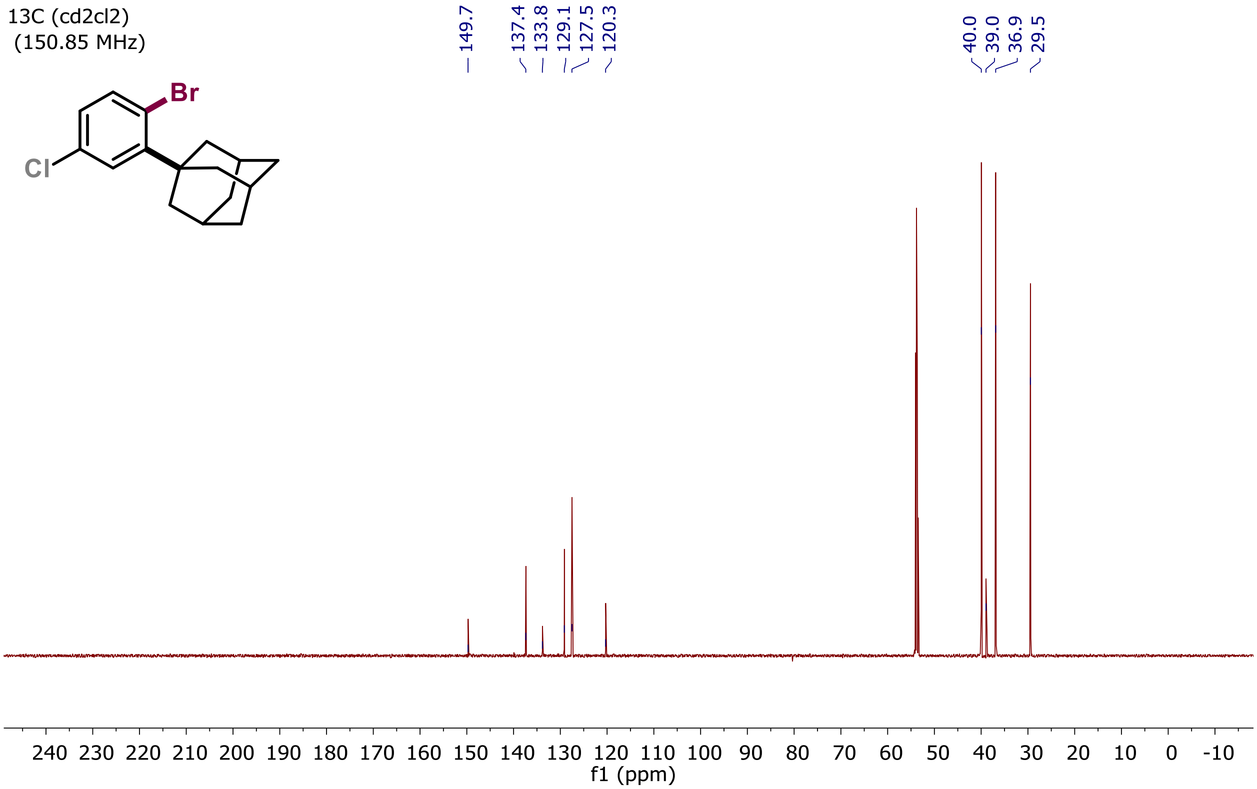
13C (cd2cl2)
(150.85 MHz)
149.7
137.4
133.8
129.1
127.5
120.3
40.0
39.0
36.9
29.5
Br
Cl
240 230 220 210 200 190 180 170 160 150 140 130 120 110 100 90 80 70 60 50 40 30 20 10 0 -10
f1 (ppm)

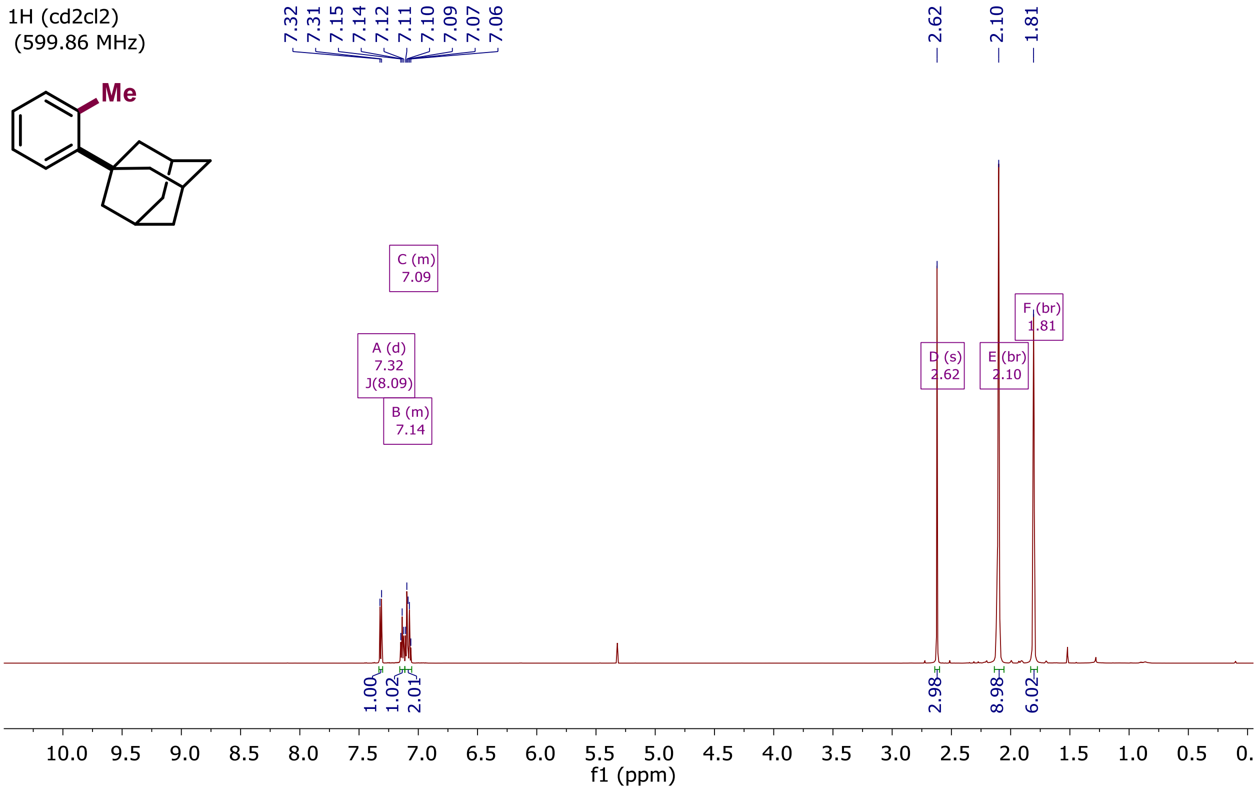
1H (cd2cl2)
(599.86 MHz)
7.32
7.31
7.15
7.14
7.12
7.11
7.10
7.09
7.07
7.06
2.62
2.10
1.81
Me
C (m)
7.09
A (d)
7.32
J(8.09)
B (m)
7.14
D (s)
2.62
E (br)
2.10
F (br)
1.81
1.00
1.02
2.01
2.98
8.98
6.02
10.0 9.5 9.0 8.5 8.0 7.5 7.0 6.5 6.0 5.5 5.0 4.5 4.0 3.5 3.0 2.5 2.0 1.5 1.0 0.5 0.
f1 (ppm)

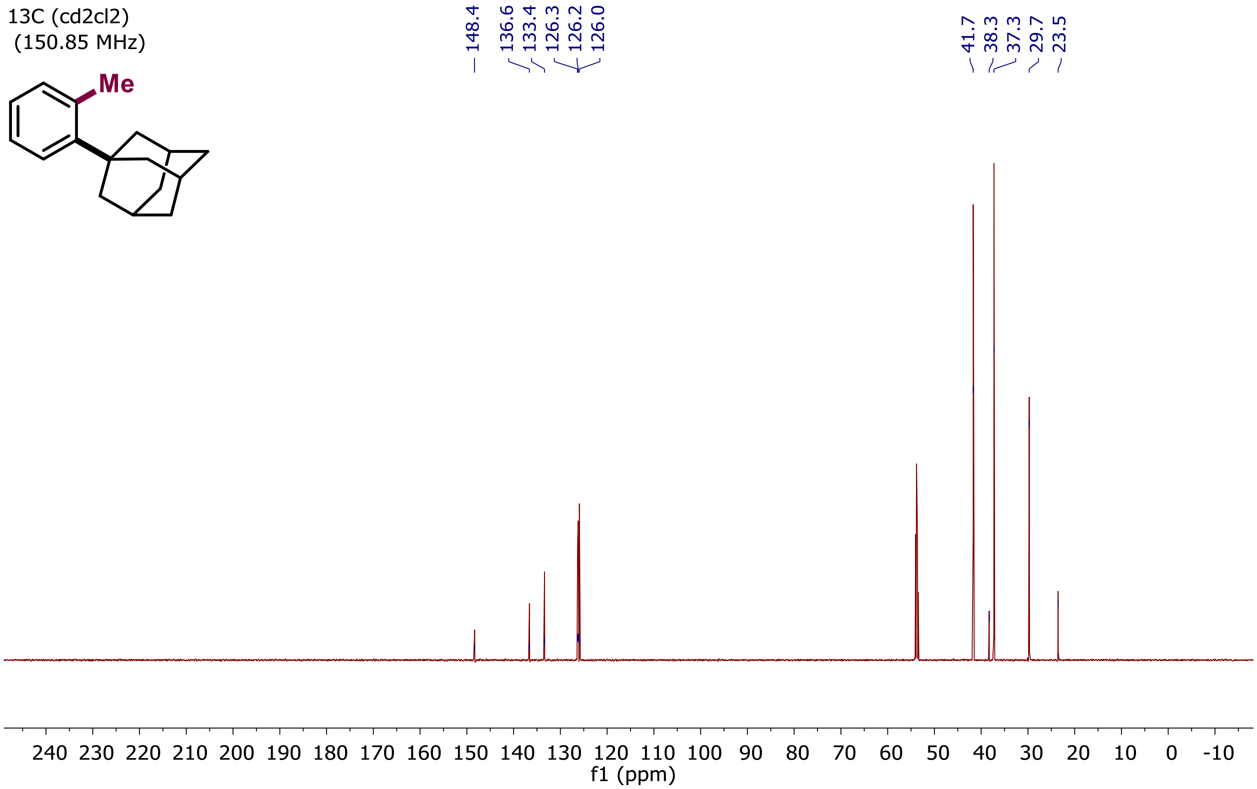
13C (cd2cl2)
(150.85 MHz)
Me
148.4
136.6
133.4
126.3
126.2
126.0
41.7
38.3
37.3
29.7
23.5
240 230 220 210 200 190 180 170 160 150 140 130 120 110 100 90 80 70 60 50 40 30 20 10 0 -10
f1 (ppm)

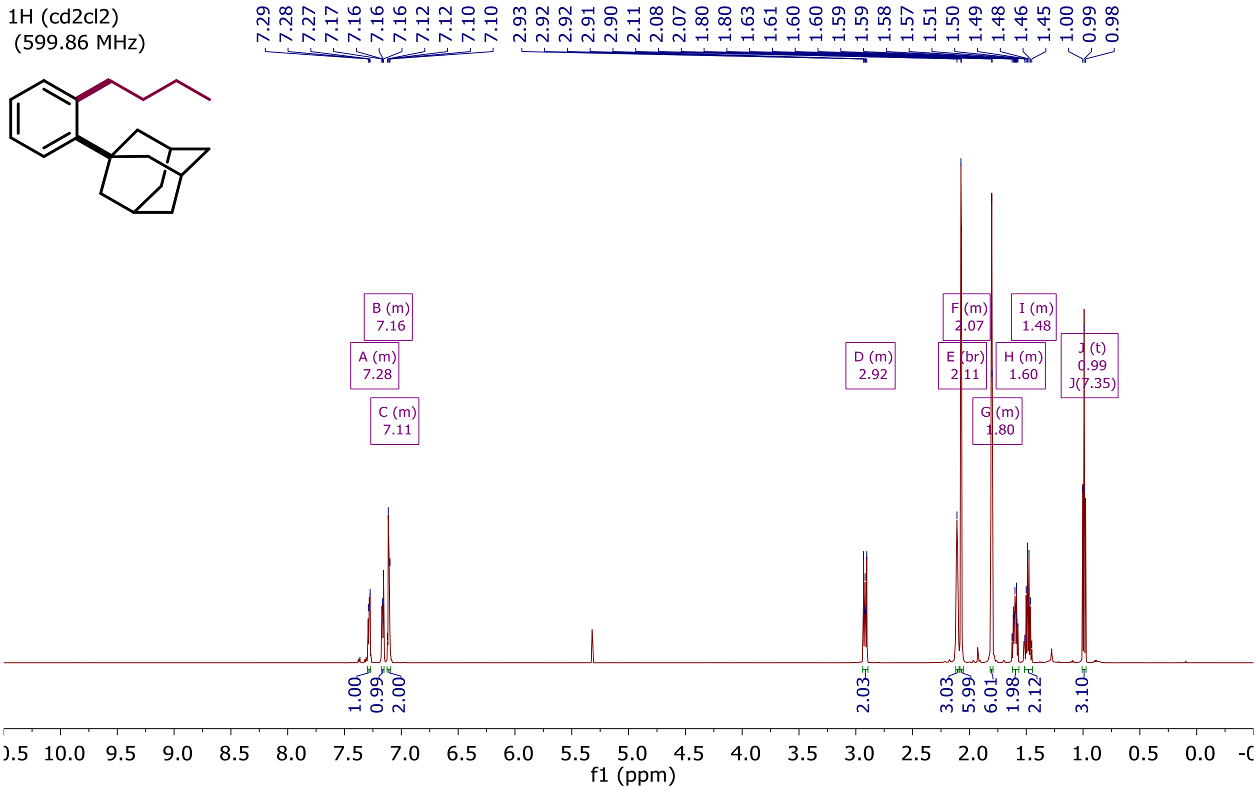
1H (cd2cl2)
(599.86 MHz)
7.29 7.28 7.27 7.17 7.16 7.16 7.16 7.12 7.12 7.10 7.10 2.93 2.92 2.92 2.91 2.90 2.11 2.08 2.07 1.80 1.80 1.63 1.61 1.60 1.60 1.59 1.59 1.58 1.57 1.51 1.50 1.49 1.48 1.46 1.45 1.00 0.99 0.98
B (m) 7.16
A (m) 7.28
C (m) 7.11
D (m) 2.92
F (m) 2.07
E (br) 2.11
G (m) 1.80
I (m) 1.48
H (m) 1.60
J (t) 0.99 J(7.35)
1.00 0.99 2.00 2.03 3.03 5.99 6.01 1.98 2.12 3.10
10.5 10.0 9.5 9.0 8.5 8.0 7.5 7.0 6.5 6.0 5.5 5.0 4.5 4.0 3.5 3.0 2.5 2.0 1.5 1.0 0.5 0.0
f1 (ppm)

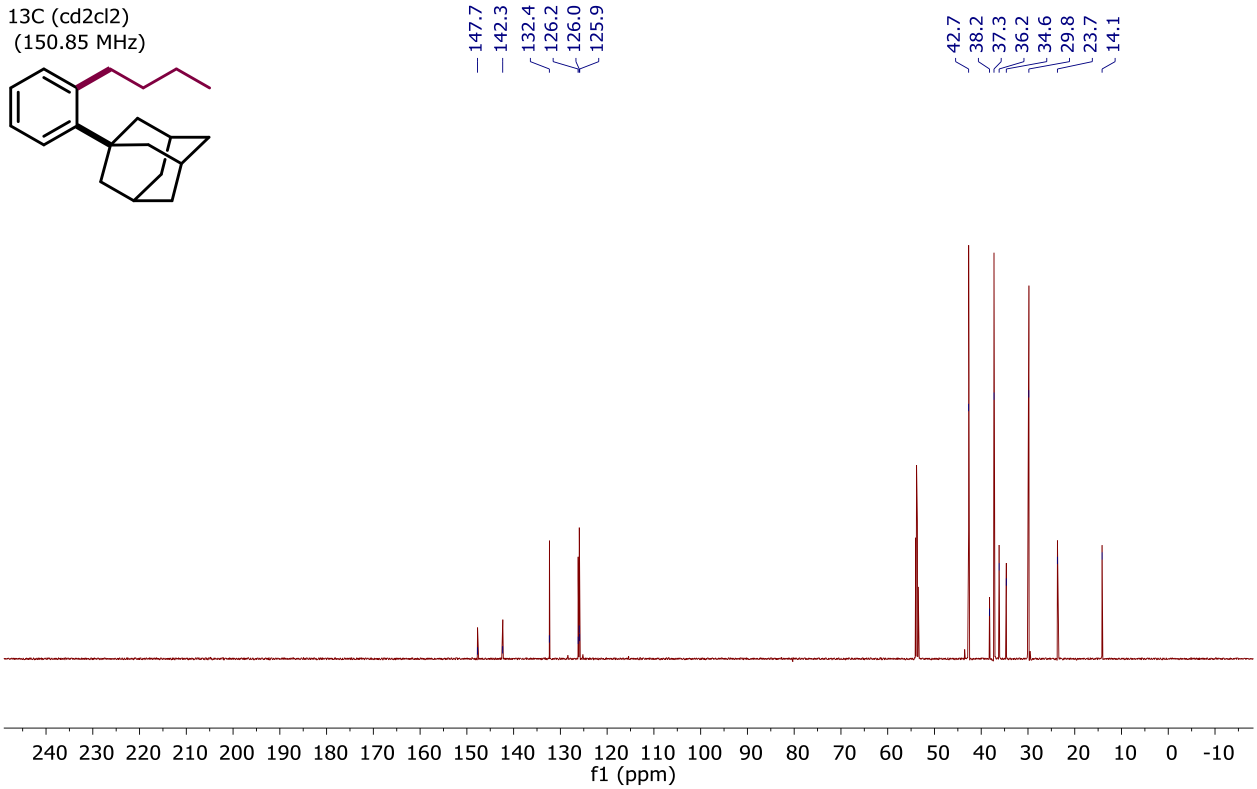
13C (cd2cl2)
(150.85 MHz)
147.7
142.3
132.4
126.2
126.0
125.9
42.7
38.2
37.3
36.2
34.6
29.8
23.7
14.1
240 230 220 210 200 190 180 170 160 150 140 130 120 110 100 90 80 70 60 50 40 30 20 10 0 -10
f1 (ppm)

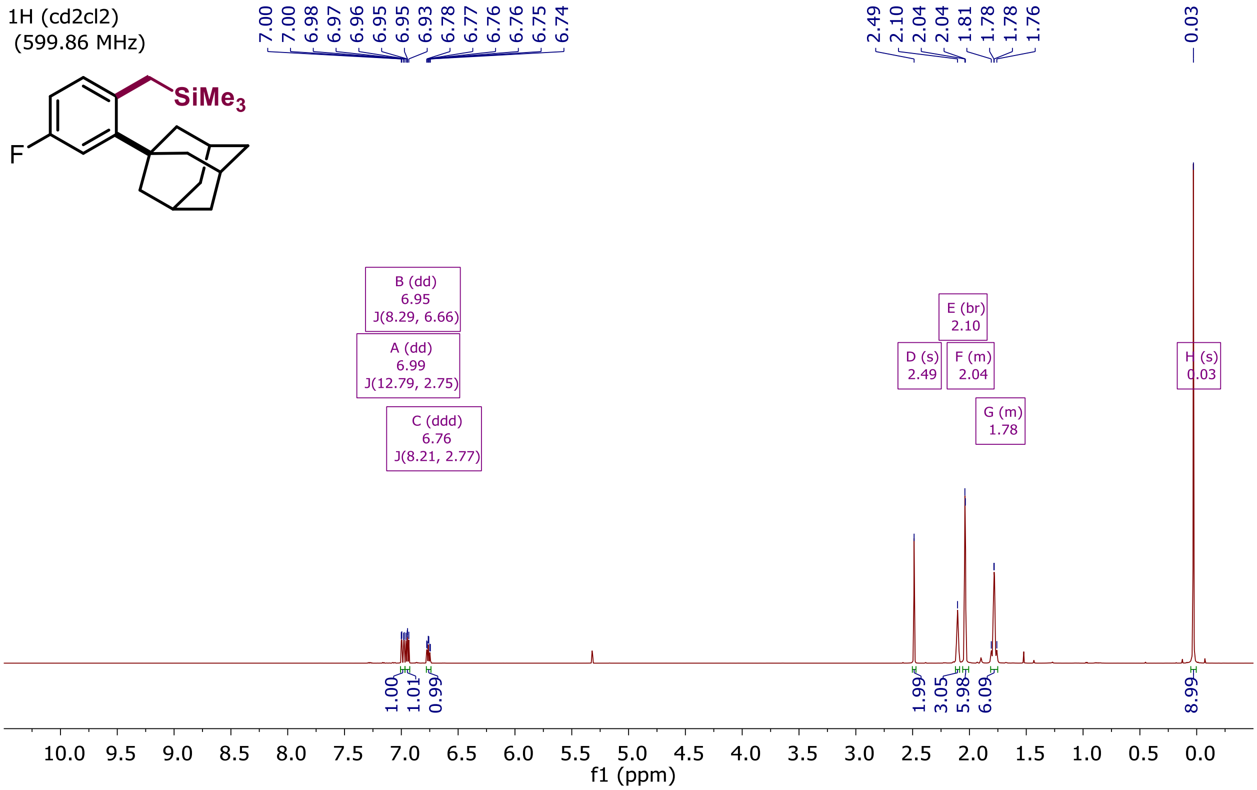
1H (cd2cl2)
(599.86 MHz)
SiMe3
F
7.00
7.00
6.98
6.97
6.96
6.95
6.95
6.93
6.78
6.77
6.76
6.76
6.75
6.74
2.49
2.10
2.04
2.04
1.81
1.78
1.78
1.76
0.03
B (dd) 6.95 J(8.29, 6.66)
A (dd) 6.99 J(12.79, 2.75)
C (ddd) 6.76 J(8.21, 2.77)
E (br) 2.10
D (s) 2.49
F (m) 2.04
G (m) 1.78
H (s) 0.03
1.00
1.01
0.99
1.99
3.05
5.98
6.09
8.99
10.0 9.5 9.0 8.5 8.0 7.5 7.0 6.5 6.0 5.5 5.0 4.5 4.0 3.5 3.0 2.5 2.0 1.5 1.0 0.5 0.0
f1 (ppm)

13C (cd2cl2)
(150.85 MHz)

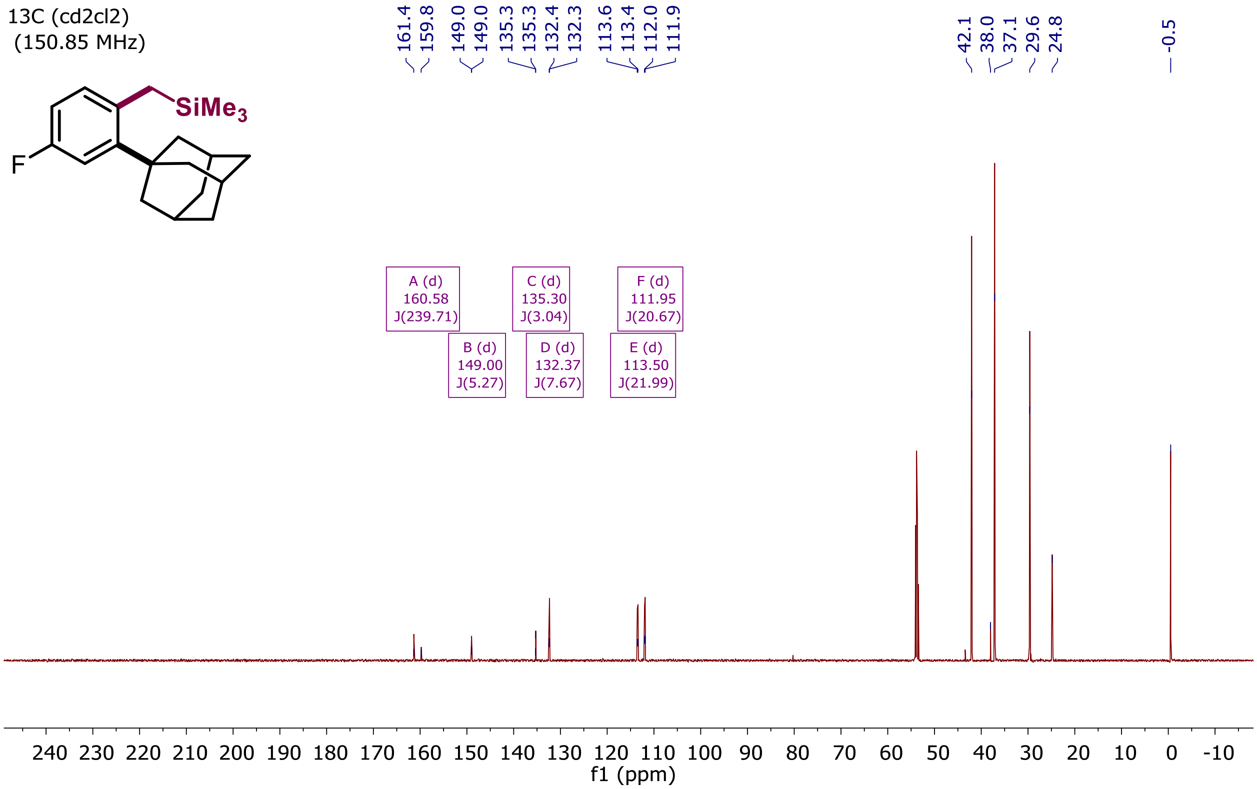

19F (cd2cl2)
(564.40 MHz)

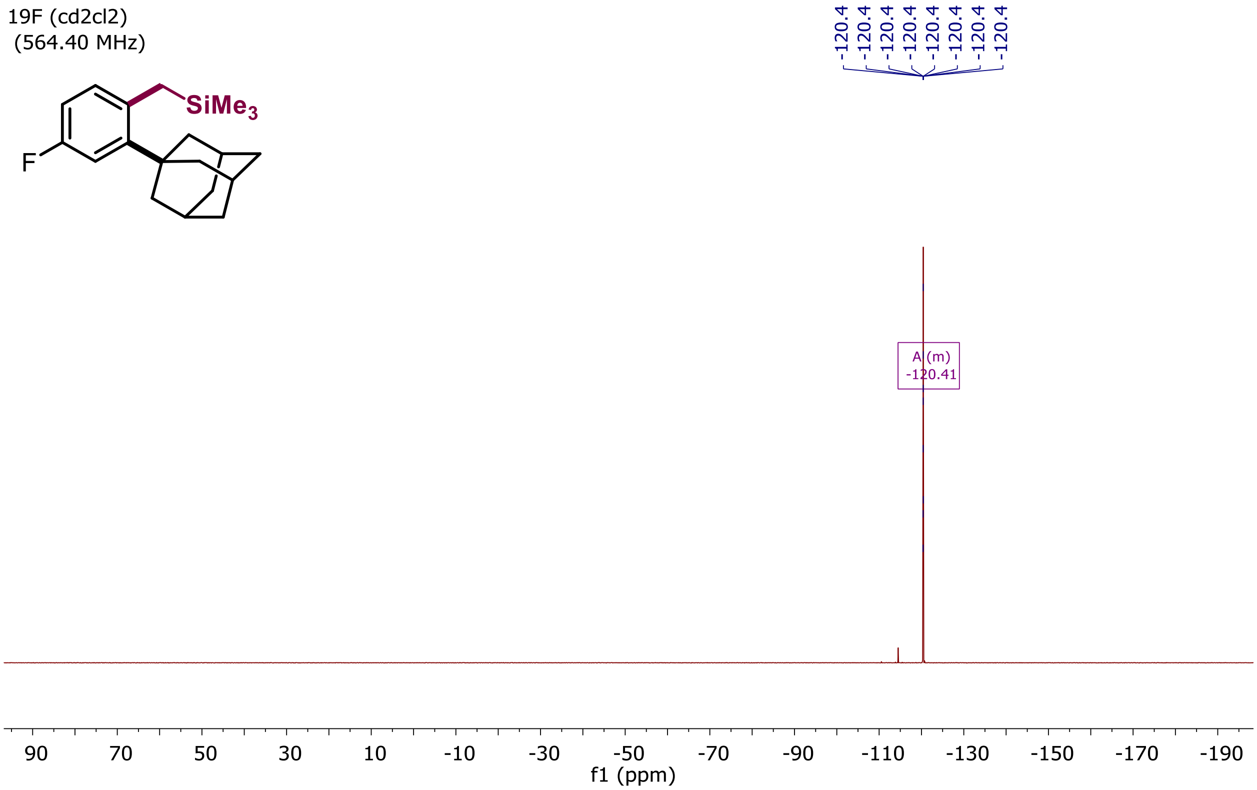

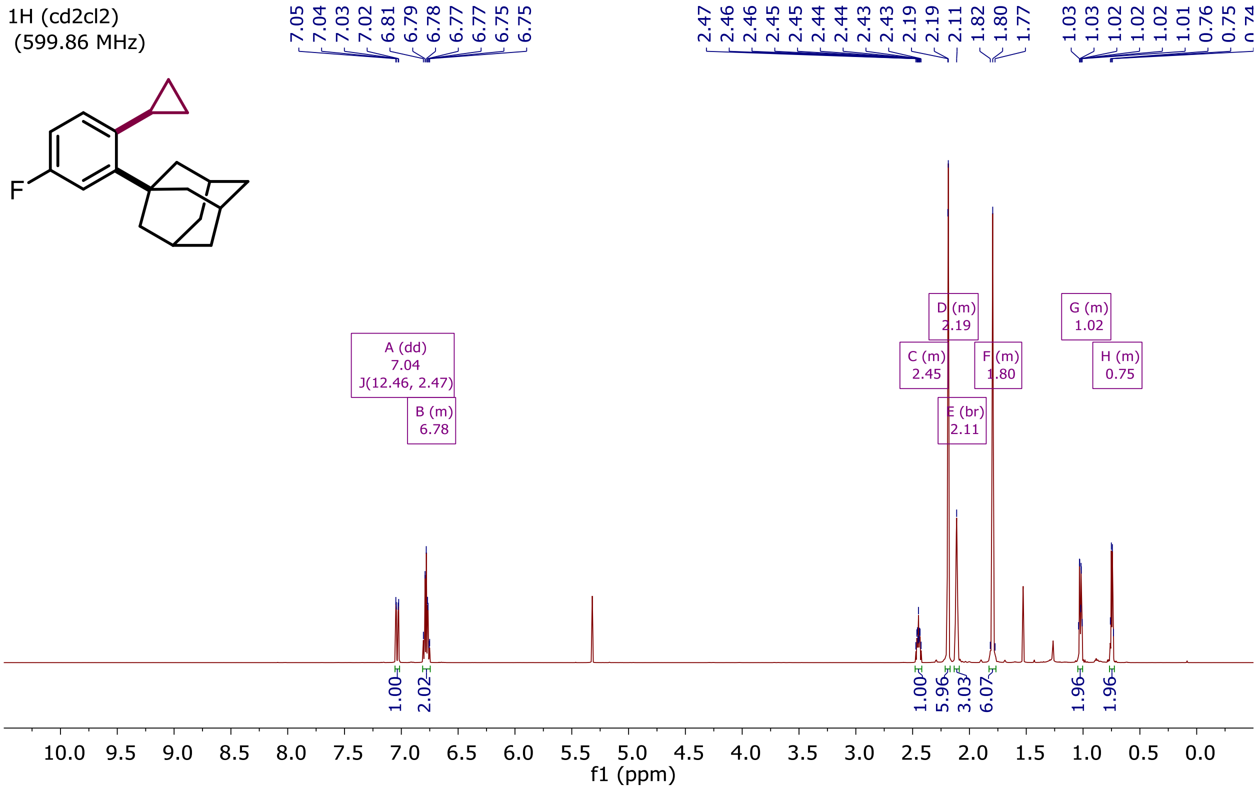
1H (cd2cl2)
(599.86 MHz)
F
7.05 7.04 7.03 7.02 6.81 6.79 6.78 6.77 6.77 6.75 6.75
2.47 2.46 2.46 2.45 2.45 2.44 2.44 2.43 2.43 2.19 2.19 2.11 1.82 1.80 1.77
1.03 1.03 1.02 1.02 1.02 1.01 0.76 0.75
A (dd) 7.04 J(12.46, 2.47)
B (m) 6.78
C (m) 2.45
D (m) 2.19
E (br) 2.11
F (m) 1.80
G (m) 1.02
H (m) 0.75
1.00 2.02 1.00 5.96 3.03 6.07 1.96 1.96
10.0 9.5 9.0 8.5 8.0 7.5 7.0 6.5 6.0 5.5 5.0 4.5 4.0 3.5 3.0 2.5 2.0 1.5 1.0 0.5 0.0
f1 (ppm)

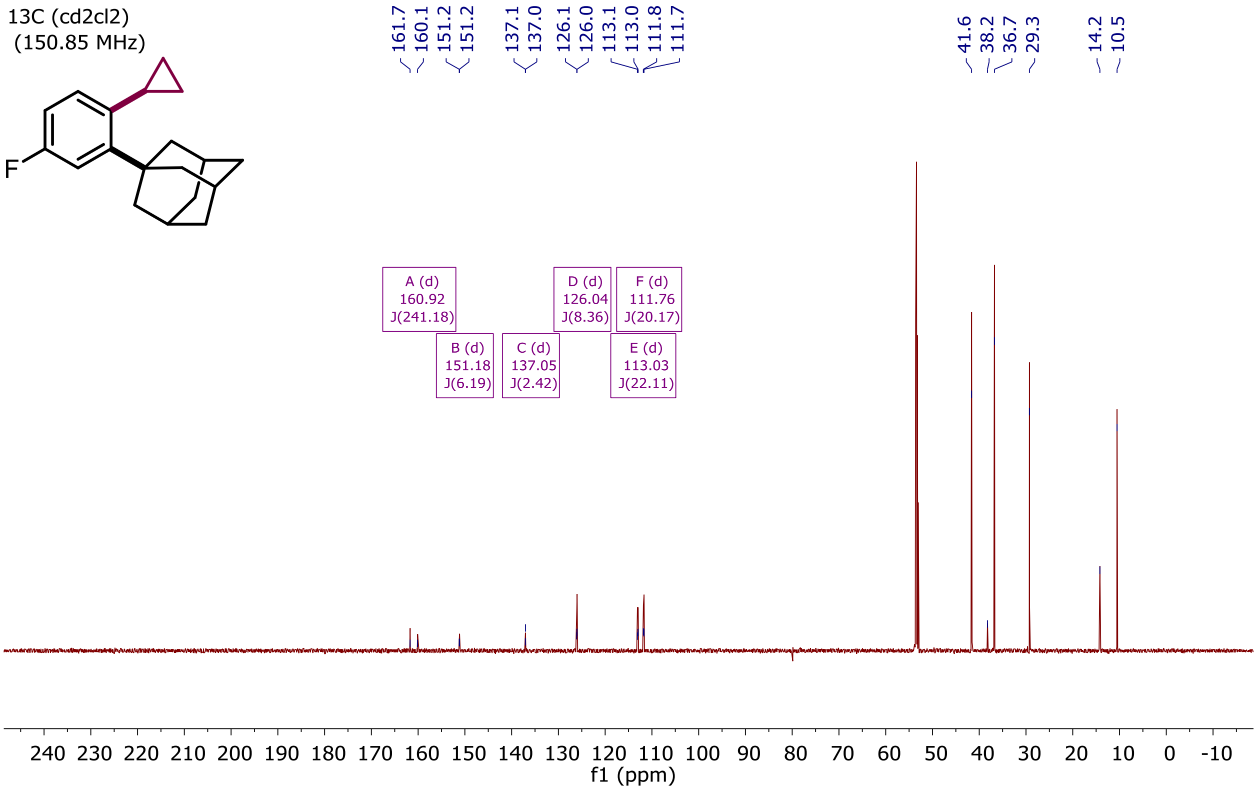
13C (cd2cl2)
(150.85 MHz)
F
161.7 160.1 151.2 151.2 137.1 137.0 126.1 126.0 113.1 113.0 111.8 111.7
41.6 38.2 36.7 29.3 14.2 10.5
A (d) 160.92 J(241.18)
B (d) 151.18 J(6.19)
C (d) 137.05 J(2.42)
D (d) 126.04 J(8.36)
E (d) 113.03 J(22.11)
F (d) 111.76 J(20.17)
240 230 220 210 200 190 180 170 160 150 140 130 120 110 100 90 80 70 60 50 40 30 20 10 0 -10
f1 (ppm)

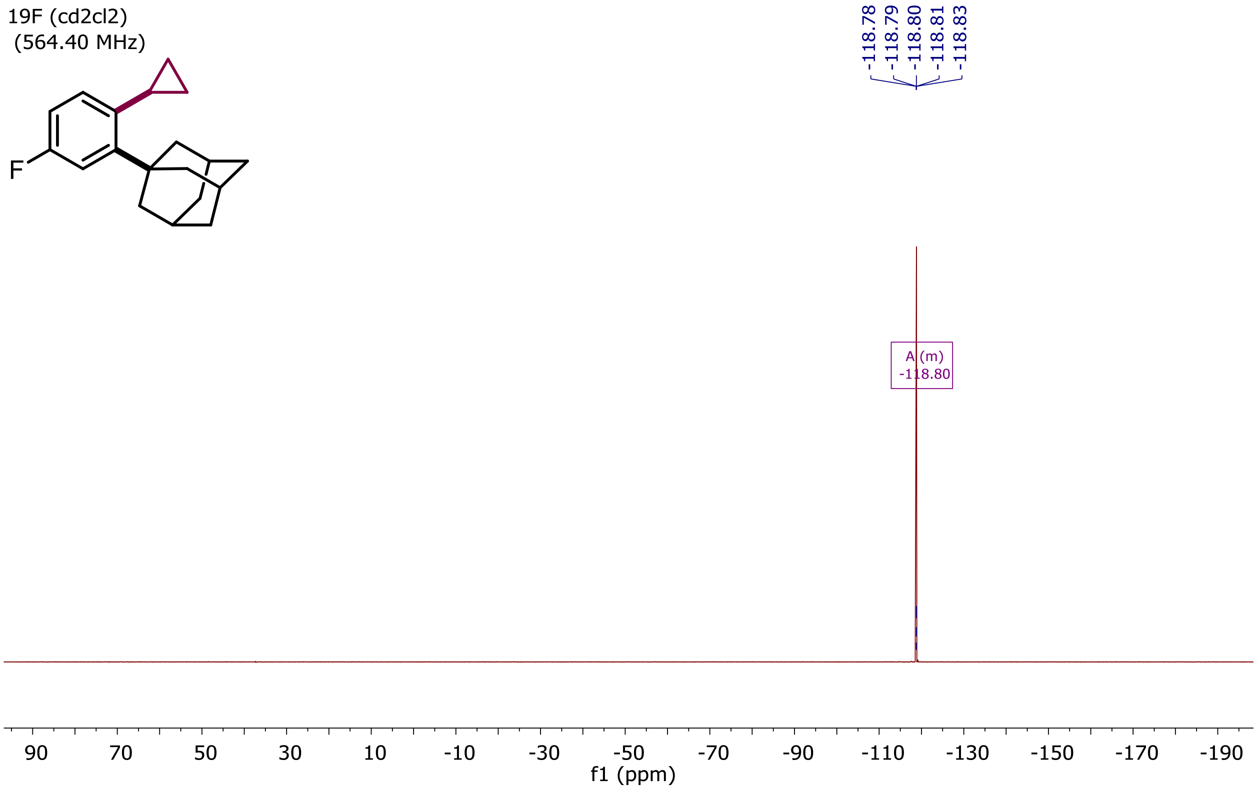
19F (cd2cl2)
(564.40 MHz)
F
-118.78
-118.79
-118.80
-118.81
-118.83
A (m)
-118.80
90 70 50 30 10 -10 -30 -50 -70 -90 -110 -130 -150 -170 -190
f1 (ppm)

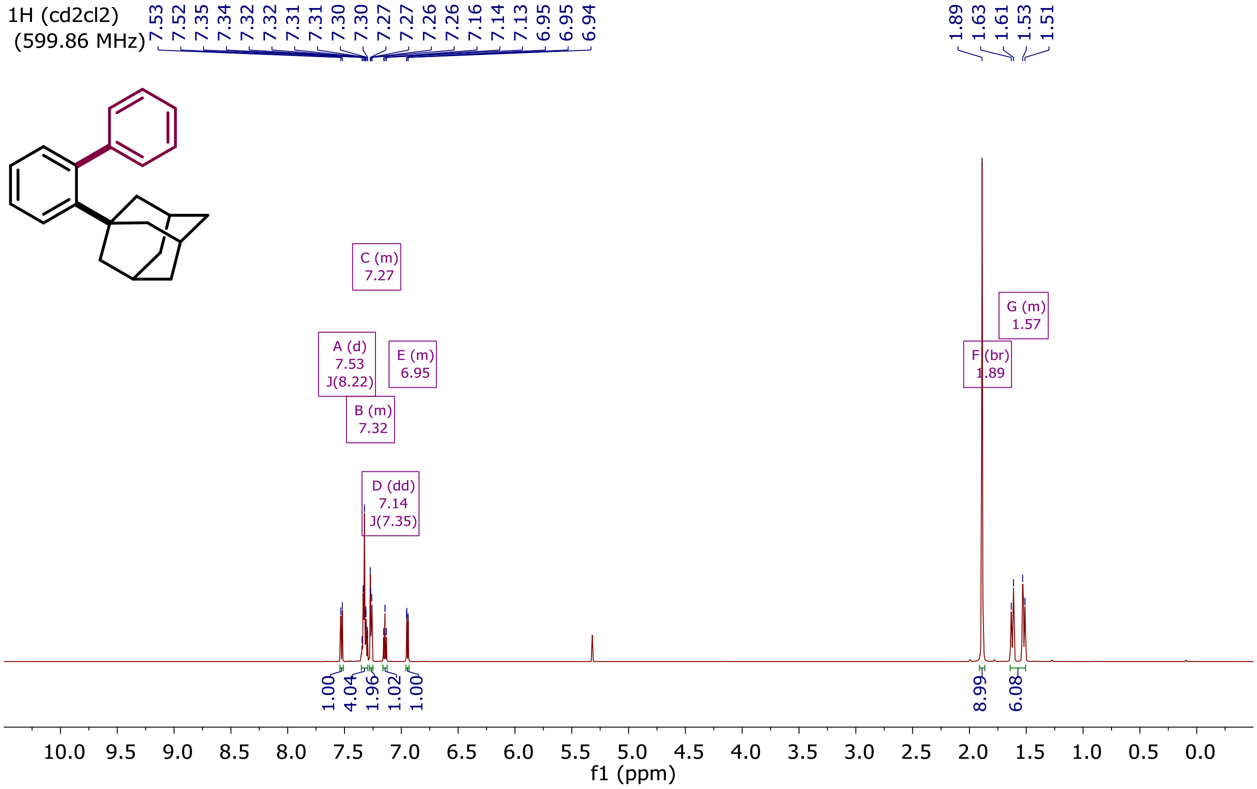
1H (cd2cl2)
(599.86 MHz)
7.53 7.52 7.35 7.34 7.32 7.32 7.31 7.31 7.30 7.30 7.27 7.27 7.26 7.26 7.16 7.14 7.13 6.95 6.95 6.94
1.89 1.63 1.61 1.53 1.51
C (m)
7.27
A (d)
7.53
J(8.22)
E (m)
6.95
B (m)
7.32
D (dd)
7.14
J(7.35)
G (m)
1.57
F (br)
1.89
1.00
4.04
1.96
1.02
1.00
8.99
6.08
10.0 9.5 9.0 8.5 8.0 7.5 7.0 6.5 6.0 5.5 5.0 4.5 4.0 3.5 3.0 2.5 2.0 1.5 1.0 0.5 0.0
f1 (ppm)

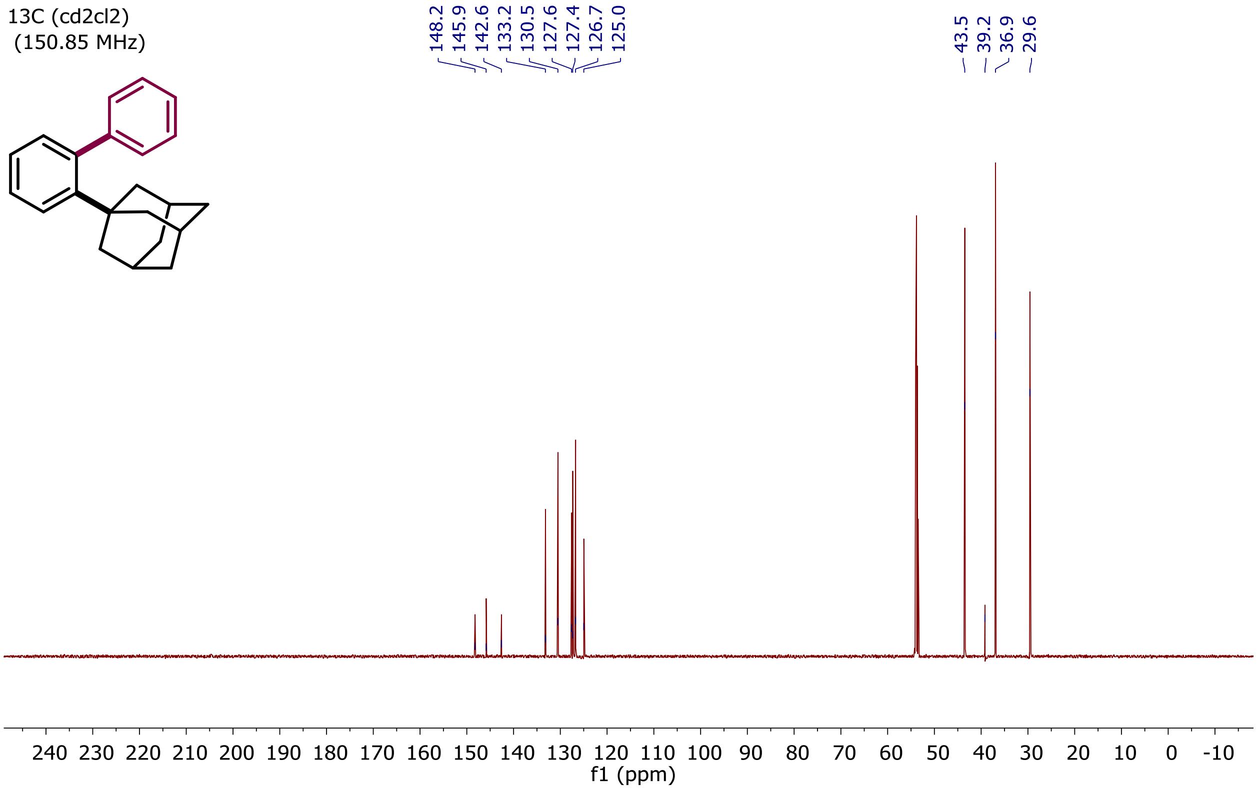
13C (cd2cl2)
(150.85 MHz)
148.2
145.9
142.6
133.2
130.5
127.6
127.4
126.7
125.0
43.5
39.2
36.9
29.6
240 230 220 210 200 190 180 170 160 150 140 130 120 110 100 90 80 70 60 50 40 30 20 10 0 -10
f1 (ppm)

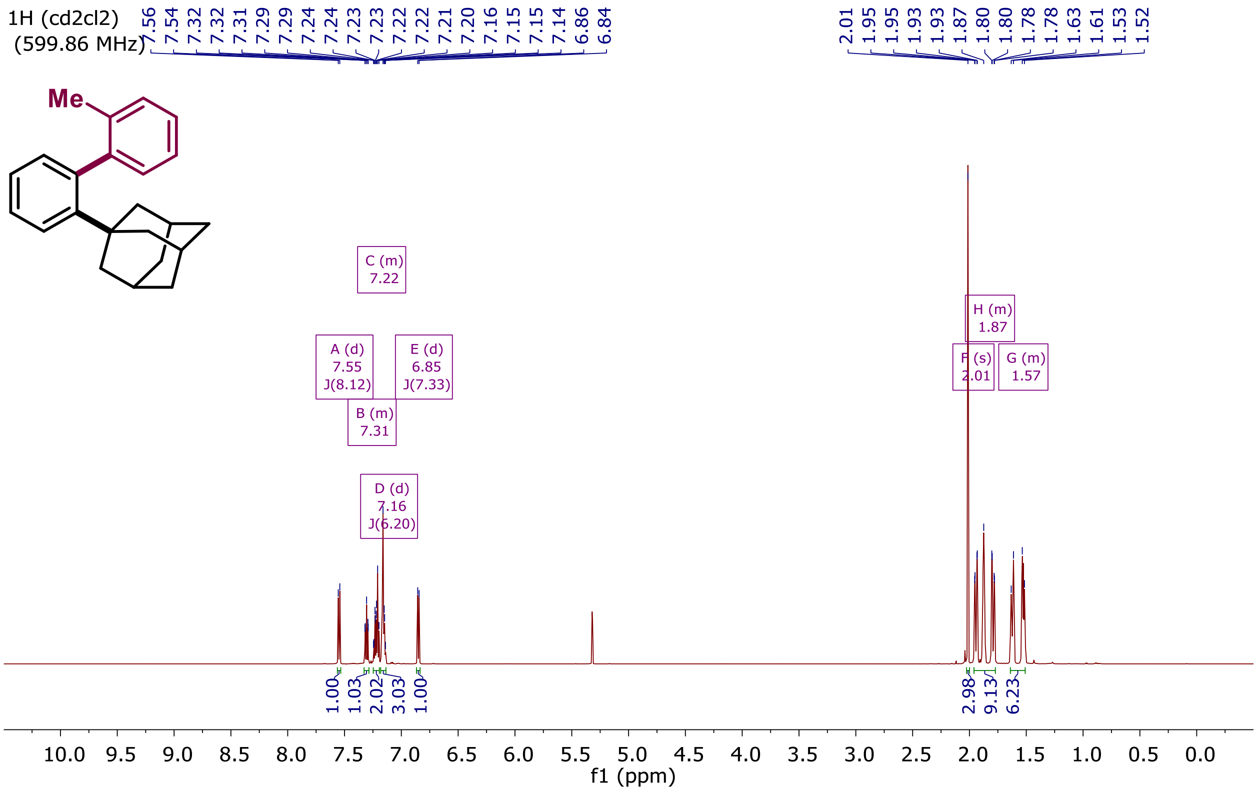
1H (cd2cl2)
(599.86 MHz)
7.56
7.54
7.32
7.32
7.31
7.29
7.29
7.24
7.24
7.23
7.23
7.22
7.22
7.21
7.20
7.16
7.15
7.14
6.86
6.84
2.01
1.95
1.95
1.93
1.93
1.87
1.80
1.80
1.78
1.63
1.61
1.53
1.52
Me
C (m)
7.22
A (d)
7.55
J(8.12)
E (d)
6.85
J(7.33)
B (m)
7.31
D (d)
7.16
J(6.20)
H (m)
1.87
F (s)
2.01
G (m)
1.57
1.00
1.03
2.02
3.03
1.00
2.98
9.13
6.23
10.0 9.5 9.0 8.5 8.0 7.5 7.0 6.5 6.0 5.5 5.0 4.5 4.0 3.5 3.0 2.5 2.0 1.5 1.0 0.5 0.0
f1 (ppm)

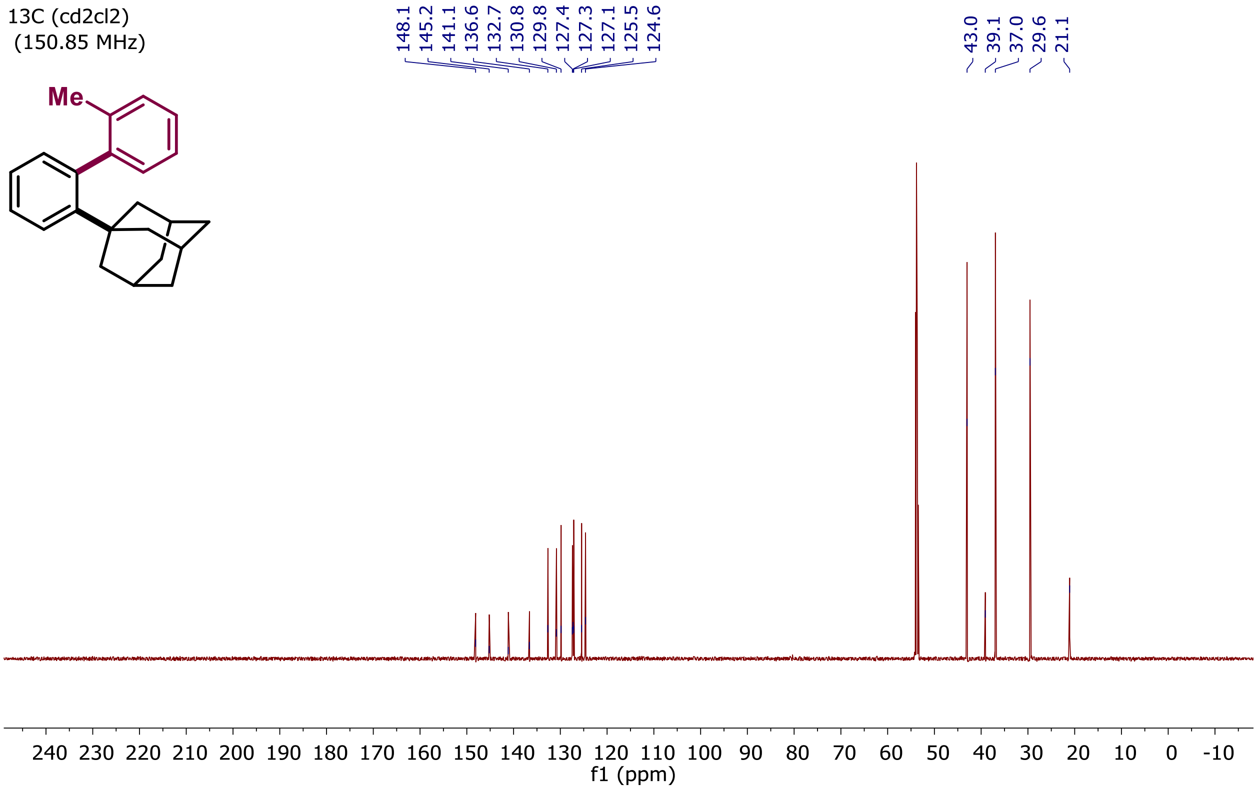
13C (cd2cl2)
(150.85 MHz)
Me
148.1
145.2
141.1
136.6
132.7
130.8
129.8
127.4
127.3
127.1
125.5
124.6
43.0
39.1
37.0
29.6
21.1
240 230 220 210 200 190 180 170 160 150 140 130 120 110 100 90 80 70 60 50 40 30 20 10 0 -10
f1 (ppm)

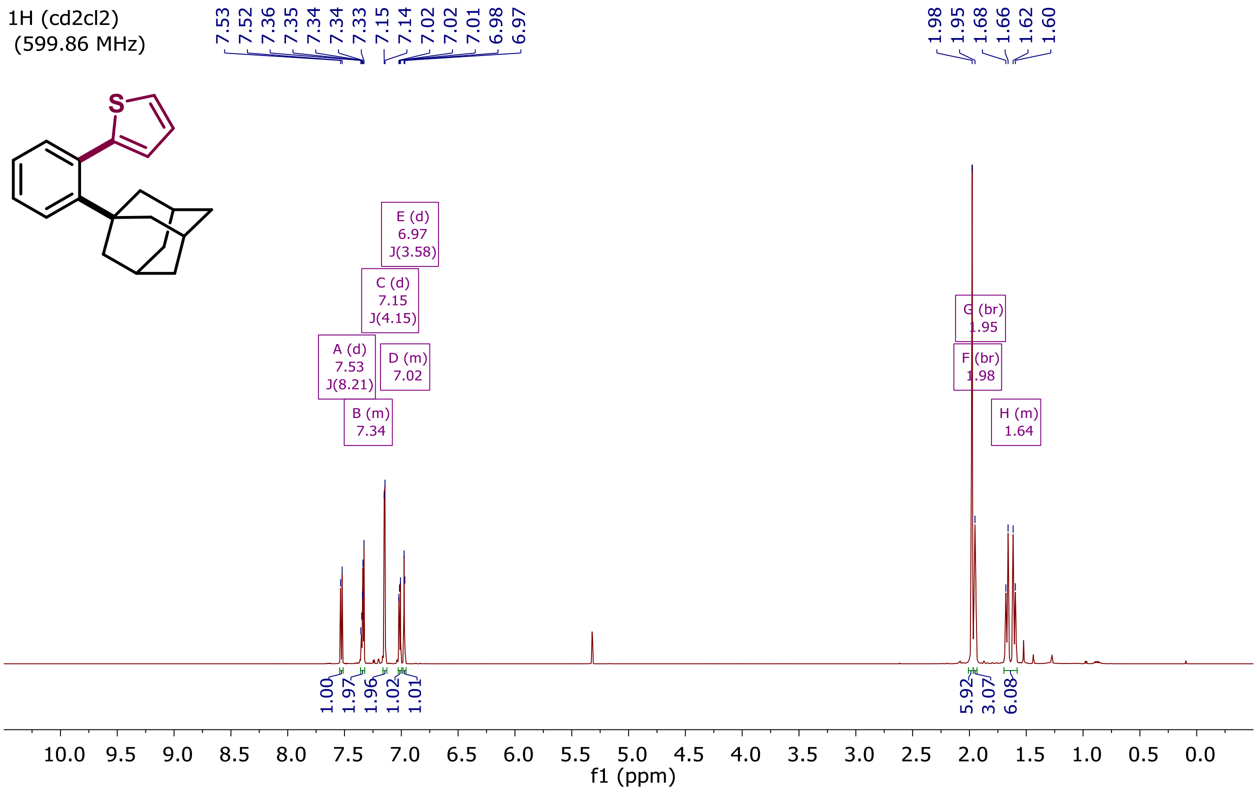
1H (cd2cl2)
(599.86 MHz)
S
7.53
7.52
7.36
7.35
7.34
7.34
7.33
7.15
7.14
7.02
7.02
7.01
6.98
6.97
1.98
1.95
1.68
1.66
1.62
1.60
E (d)
6.97
J(3.58)
C (d)
7.15
J(4.15)
A (d)
7.53
J(8.21)
D (m)
7.02
B (m)
7.34
G (br)
1.95
F (br)
1.98
H (m)
1.64
1.00
1.97
1.96
1.02
1.01
5.92
3.07
6.08
10.0 9.5 9.0 8.5 8.0 7.5 7.0 6.5 6.0 5.5 5.0 4.5 4.0 3.5 3.0 2.5 2.0 1.5 1.0 0.5 0.0
f1 (ppm)

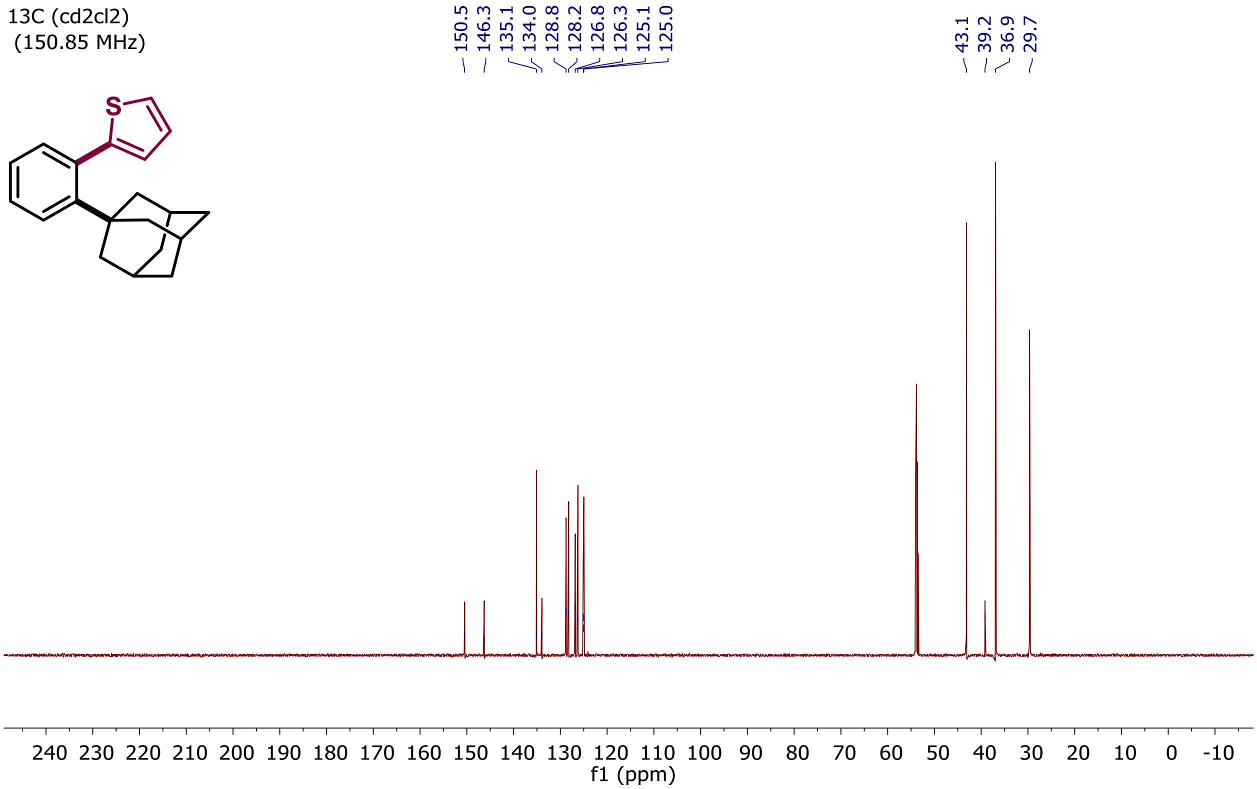
13C (cd2cl2)
(150.85 MHz)
150.5
146.3
135.1
134.0
128.8
128.2
126.8
126.3
125.1
125.0
43.1
39.2
36.9
29.7
S
240 230 220 210 200 190 180 170 160 150 140 130 120 110 100 90 80 70 60 50 40 30 20 10 0 -10
f1 (ppm)

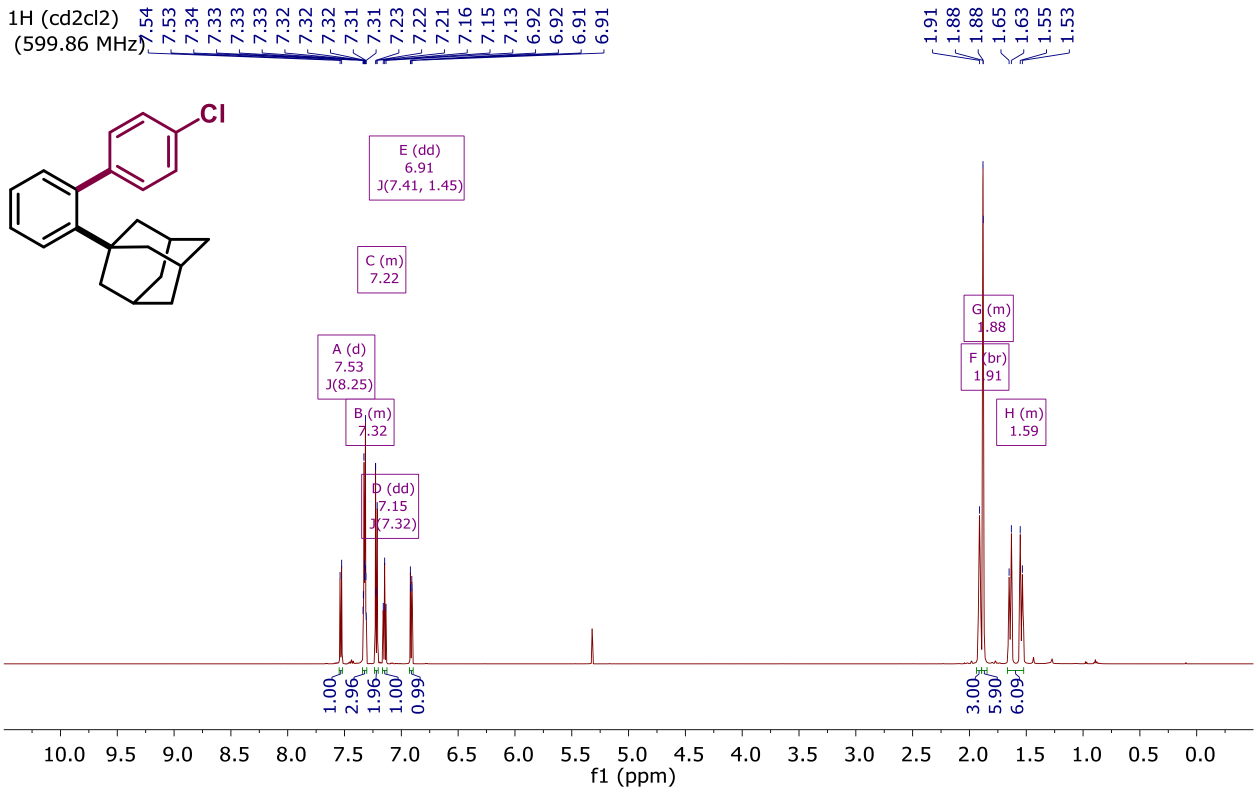
1H (cd2cl2)
(599.86 MHz)
7.54
7.53
7.34
7.33
7.33
7.33
7.32
7.32
7.32
7.31
7.31
7.23
7.22
7.21
7.16
7.15
7.13
6.92
6.92
6.91
6.91
1.91
1.88
1.88
1.65
1.63
1.55
1.53
Cl
E (dd)
6.91
J(7.41, 1.45)
C (m)
7.22
A (d)
7.53
J(8.25)
B (m)
7.32
D (dd)
7.15
J(7.32)
G (m)
1.88
F (br)
1.91
H (m)
1.59
1.00
2.96
1.96
1.00
0.99
3.00
5.90
6.09
10.0 9.5 9.0 8.5 8.0 7.5 7.0 6.5 6.0 5.5 5.0 4.5 4.0 3.5 3.0 2.5 2.0 1.5 1.0 0.5 0.0
f1 (ppm)

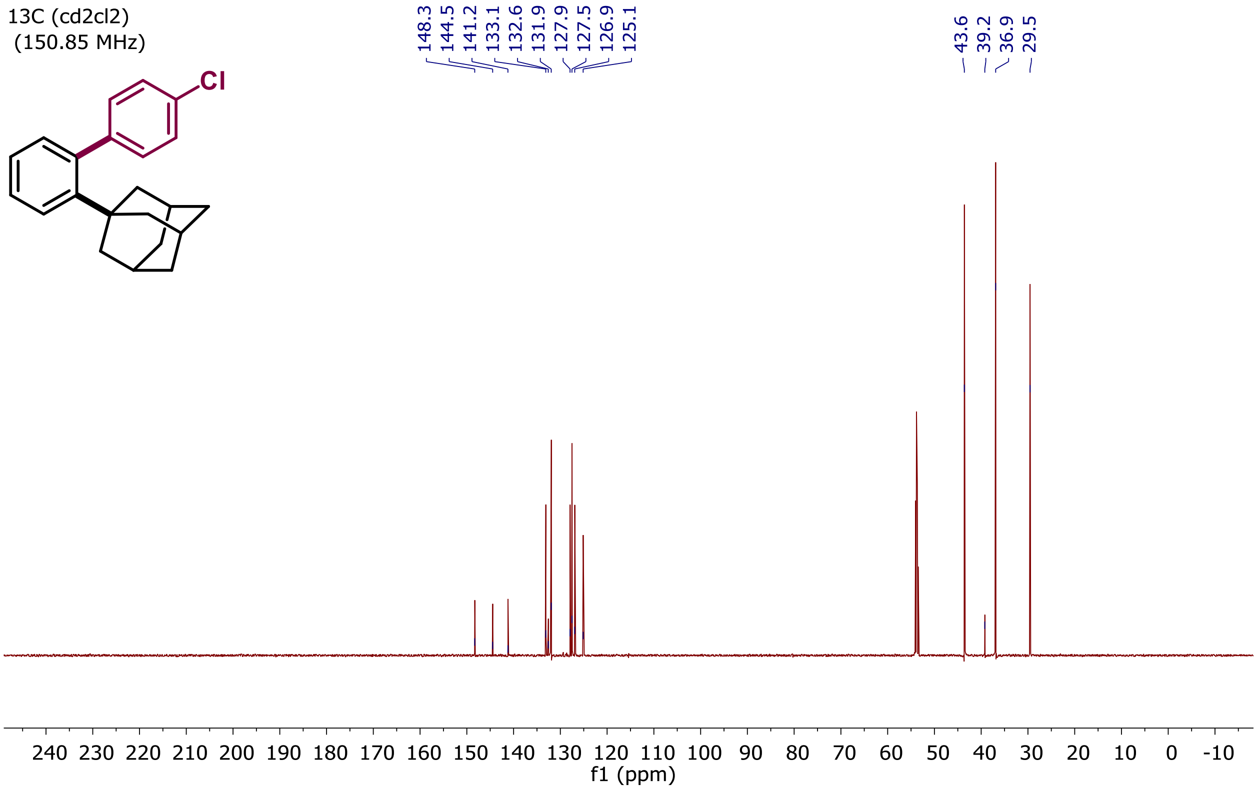
13C (cd2cl2)
(150.85 MHz)
148.3
144.5
141.2
133.1
132.6
131.9
127.9
127.5
126.9
125.1
43.6
39.2
36.9
29.5
Cl
240 230 220 210 200 190 180 170 160 150 140 130 120 110 100 90 80 70 60 50 40 30 20 10 0 -10
f1 (ppm)

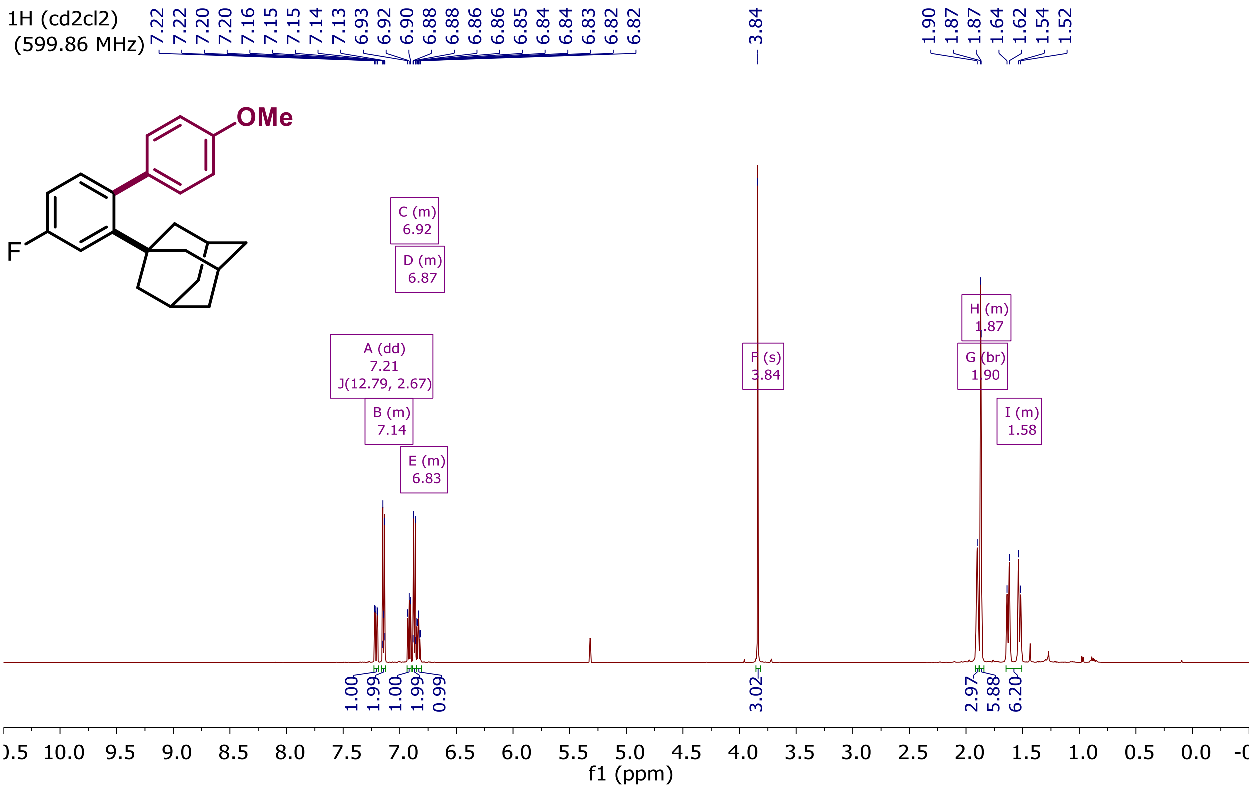
1H (cd2cl2)
(599.86 MHz)
7.22
7.22
7.20
7.20
7.16
7.15
7.15
7.14
7.13
6.93
6.92
6.90
6.88
6.88
6.86
6.86
6.85
6.84
6.84
6.83
6.82
6.82
3.84
1.90
1.87
1.87
1.64
1.62
1.54
1.52
OMe
F
C (m)
6.92
D (m)
6.87
A (dd)
7.21
J(12.79, 2.67)
B (m)
7.14
E (m)
6.83
F (s)
3.84
H (m)
1.87
G (br)
1.90
I (m)
1.58
1.00
1.99
1.00
1.99
0.99
3.02
2.97
5.88
6.20
f1 (ppm)

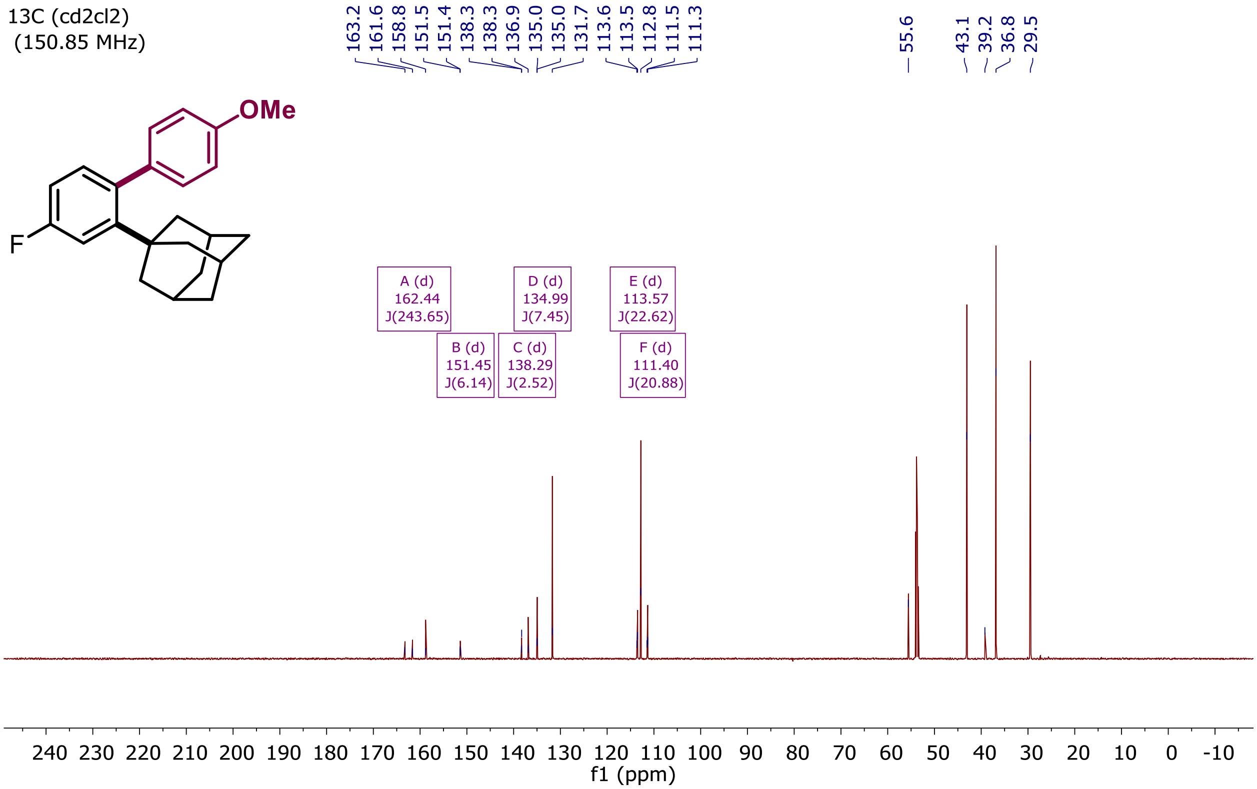
13C (cd2cl2)
(150.85 MHz)
163.2
161.6
158.8
151.5
151.4
138.3
138.3
136.9
135.0
135.0
131.7
113.6
113.5
112.8
111.5
111.3
55.6
43.1
39.2
36.8
29.5
OMe
F
A (d)
162.44
J(243.65)
B (d)
151.45
J(6.14)
C (d)
138.29
J(2.52)
D (d)
134.99
J(7.45)
E (d)
113.57
J(22.62)
F (d)
111.40
J(20.88)
240 230 220 210 200 190 180 170 160 150 140 130 120 110 100 90 80 70 60 50 40 30 20 10 0 -10
f1 (ppm)

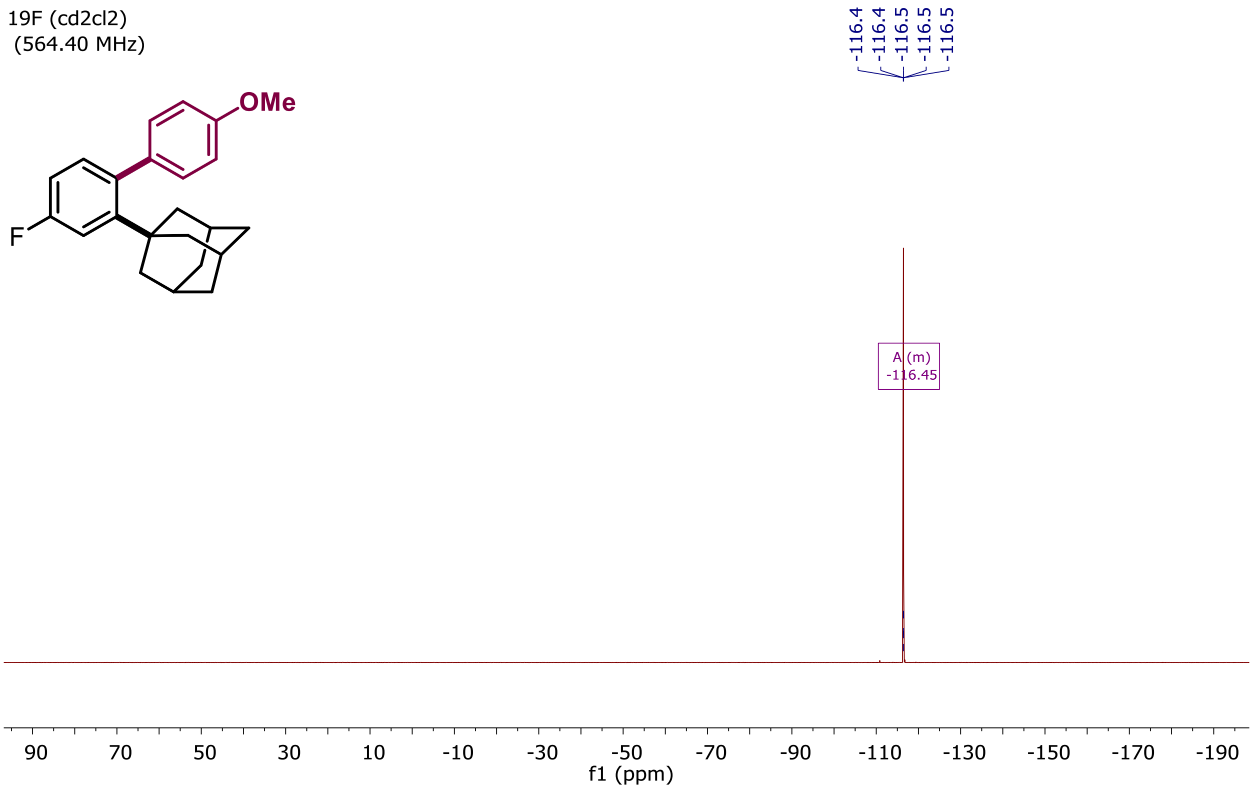
19F (cd2cl2)
(564.40 MHz)
-116.4
-116.4
-116.5
-116.5
-116.5
OMe
F
A (m)
-116.45
90 70 50 30 10 -10 -30 -50 -70 -90 -110 -130 -150 -170 -190
f1 (ppm)

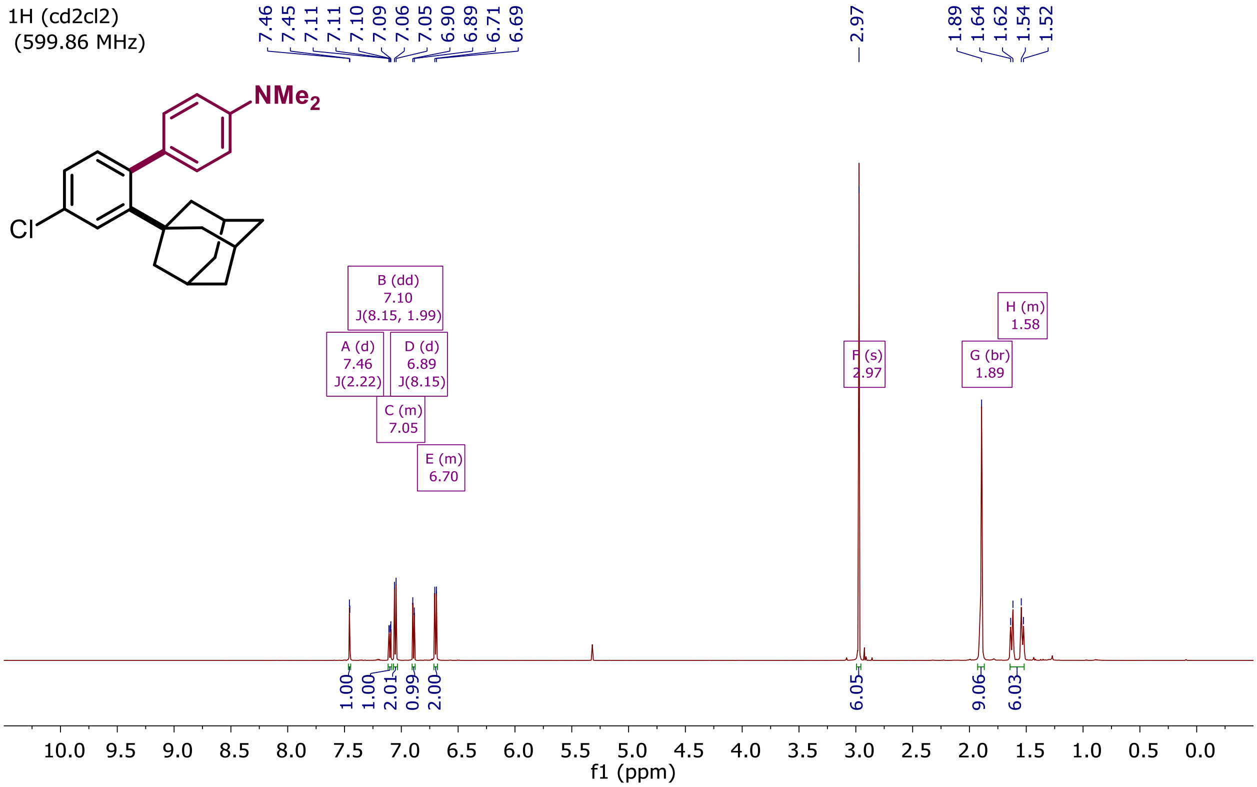
1H (cd2cl2)
(599.86 MHz)
7.46
7.45
7.11
7.11
7.10
7.09
7.06
7.05
6.90
6.89
6.71
6.69
2.97
1.89
1.64
1.62
1.54
1.52
NMe2
Cl
B (dd)
7.10
J(8.15, 1.99)
A (d)
7.46
J(2.22)
D (d)
6.89
J(8.15)
C (m)
7.05
E (m)
6.70
F (s)
2.97
G (br)
1.89
H (m)
1.58
1.00
1.00
2.01
0.99
2.00
6.05
9.06
6.03
10.0 9.5 9.0 8.5 8.0 7.5 7.0 6.5 6.0 5.5 5.0 4.5 4.0 3.5 3.0 2.5 2.0 1.5 1.0 0.5 0.0
f1 (ppm)

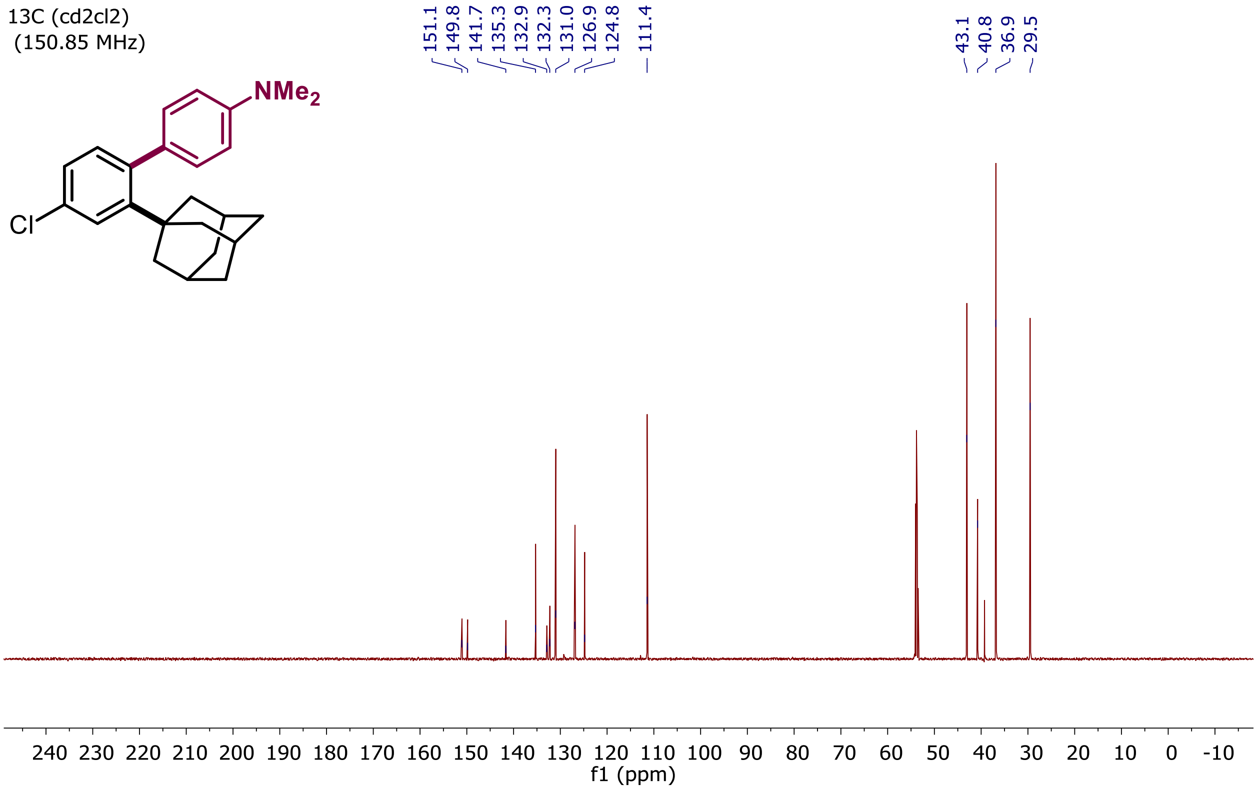
13C (cd2cl2)
(150.85 MHz)
151.1
149.8
141.7
135.3
132.9
132.3
131.0
126.9
124.8
111.4
43.1
40.8
36.9
29.5
NMe2
Cl
240 230 220 210 200 190 180 170 160 150 140 130 120 110 100 90 80 70 60 50 40 30 20 10 0 -10
f1 (ppm)

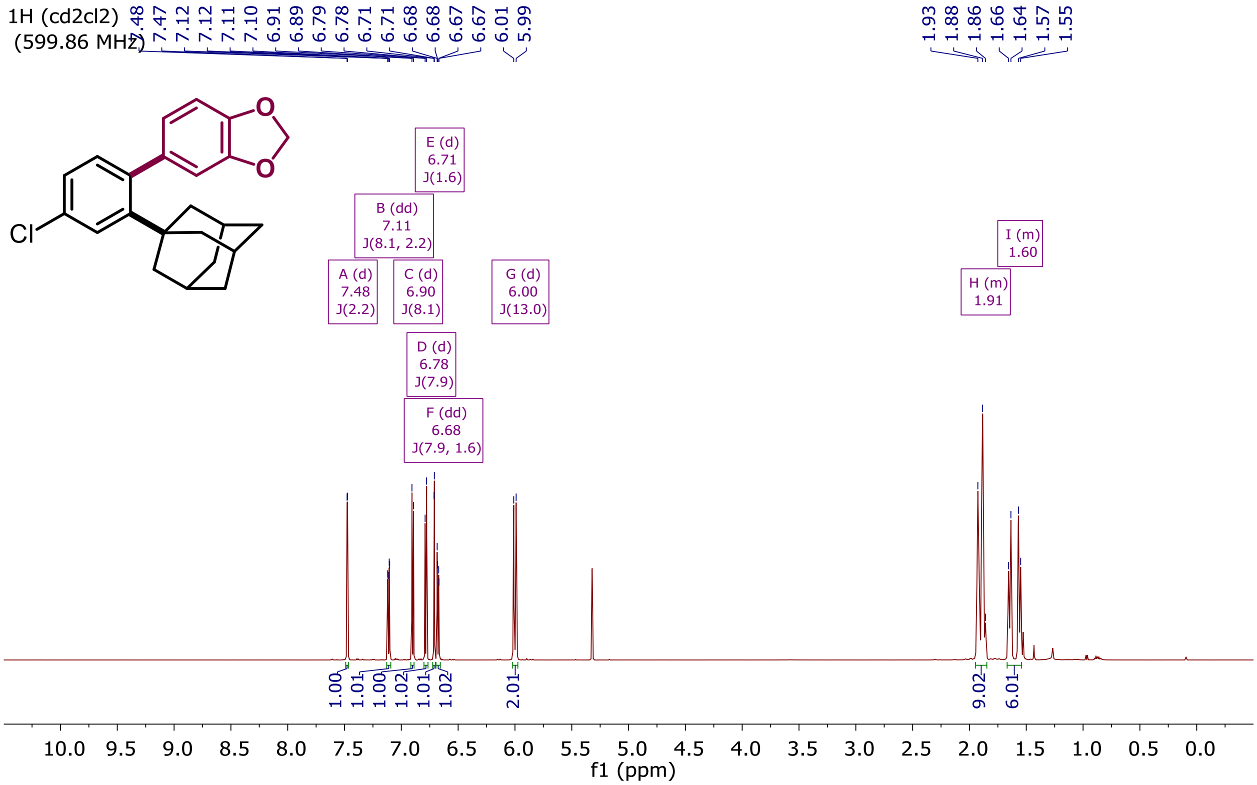
1H (cd2cl2)
(599.86 MHz)
7.48 7.47 7.12 7.12 7.11 7.10 6.91 6.89 6.79 6.78 6.71 6.71 6.68 6.68 6.67 6.67 6.01 5.99
1.93 1.88 1.86 1.66 1.64 1.57 1.55
Cl
O
O
E (d) 6.71 J(1.6)
B (dd) 7.11 J(8.1, 2.2)
A (d) 7.48 J(2.2)
C (d) 6.90 J(8.1)
G (d) 6.00 J(13.0)
D (d) 6.78 J(7.9)
F (dd) 6.68 J(7.9, 1.6)
I (m) 1.60
H (m) 1.91
1.00 1.01 1.00 1.02 1.01 1.02
2.01
9.02 6.01
10.0 9.5 9.0 8.5 8.0 7.5 7.0 6.5 6.0 5.5 5.0 4.5 4.0 3.5 3.0 2.5 2.0 1.5 1.0 0.5 0.0
f1 (ppm)

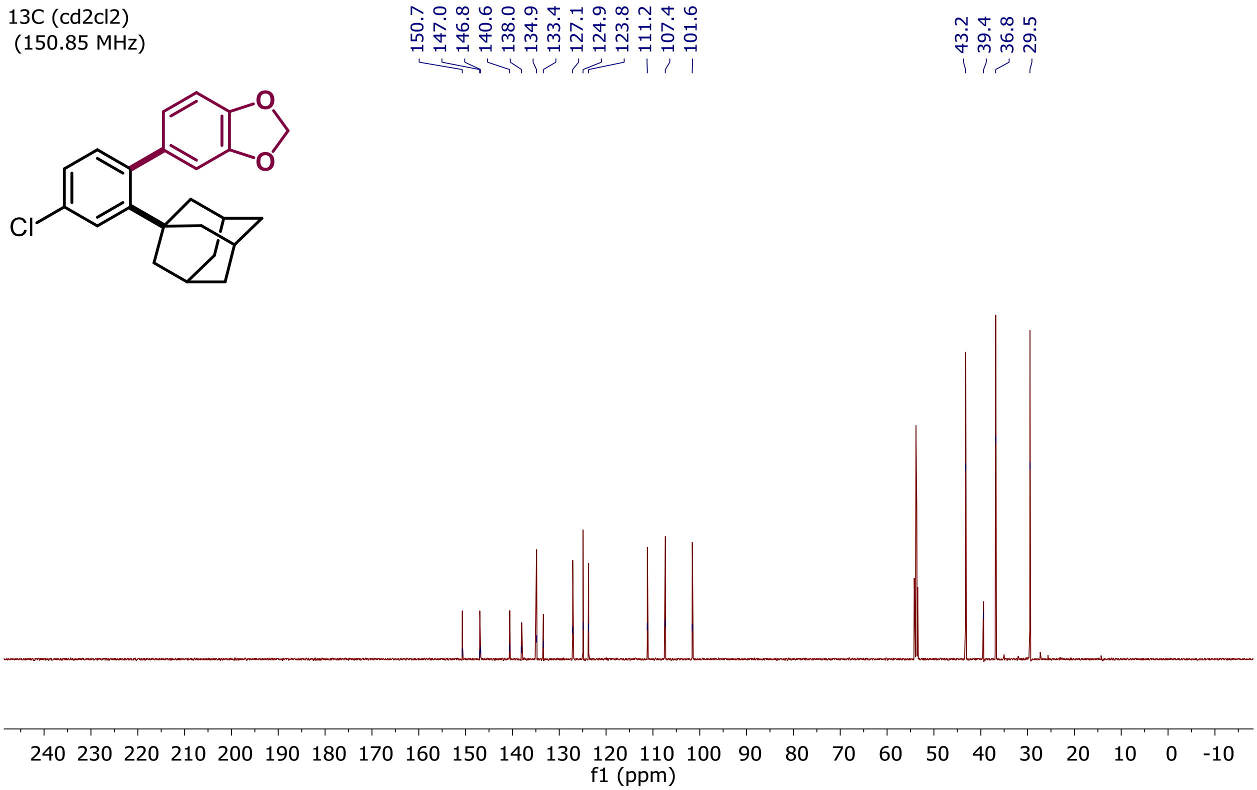
13C (cd2cl2)
(150.85 MHz)
150.7 147.0 146.8 140.6 138.0 134.9 133.4 127.1 124.9 123.8 111.2 107.4 101.6
43.2 39.4 36.8 29.5
Cl
O
O
240 230 220 210 200 190 180 170 160 150 140 130 120 110 100 90 80 70 60 50 40 30 20 10 0 -10
f1 (ppm)

6. References

[1] G. R. Fulmer, A. J. M. Miller, N. H. Sherden, H. E. Gottlieb, A. Nudelman, B. M. Stoltz, J. E. Bercaw, K. I. Goldberg, *Organometallics* **2010**, *29*, 2176-2179.

[2] M. Aufiero, T. Sperger, A. S. K. Tsang, F. Schoenebeck, *Angew. Chem. Int. Ed.* **2015**, *54*, 10322-10326.

[3] D. Lu, Z. Meng, G. A. Thakur, P. Fan, J. Steed, C. L. Tartal, D. P. Hurst, P. H. Reggio, J. R. Deschamps, D. A. Parrish, C. George, T. U. C. Järbe, R. J. Lamb, A. Makriyannis, *J. Med. Chem.* **2005**, *48*, 4576-4585.

[4] A. R. Kapdi, I. J. S. Fairlamb, *New Journal of Chemistry* **2013**, *37*, 961-964.

[5] G. Chiellini, N.-H. Nguyen, H. A. I. Yoshihara, T. S. Scanlan, *Bioorganic & Medicinal Chemistry Letters* **2000**, *10*, 2607-2611.

[6] S. M. Maddox, A. N. Dinh, F. Armenta, J. Um, J. L. Gustafson, *Org. Lett.* **2016**, *18*, 5476-5479.

[7] H. Y. Choi, D. Y. Chi, *J. Am. Chem. Soc.* **2001**, *123*, 9202-9203.

[8] L. Testaferri, M. Tiecco, P. Spagnolo, P. Zanirato, G. Martelli, *Journal of the Chemical Society, Perkin Transactions 2* **1976**, DOI 10.1039/P29760000662, 662-668.

[9] M. J. Frisch, G. W. Trucks, H. B. Schlegel, G. E. Scuseria, M. A. Robb, J. R. Cheeseman, G. Scalmani, V. Barone, G. A. Petersson, H. Nakatsuji, X. Li, M. Caricato, A. V. Marenich, J. Bloino, B. G. Janesko, R. Gomperts, B. Mennucci, H. P. Hratchian, J. V. Ortiz, A. F. Izmaylov, J. L. Sonnenberg, Williams, F. Ding, F. Lipparini, F. Egidi, J. Goings, B. Peng, A. Petrone, T. Henderson, D. Ranasinghe, V. G. Zakrzewski, J. Gao, N. Rega, G. Zheng, W. Liang, M. Hada, M. Ehara, K. Toyota, R. Fukuda, J. Hasegawa, M. Ishida, T. Nakajima, Y. Honda, O. Kitao, H. Nakai, T. Vreven, K. Throssell, J. A. Montgomery Jr., J. E. Peralta, F. Ogliaro, M. J. Bearpark, J. J. Heyd, E. N. Brothers, K. N. Kudin, V. N. Staroverov, T. A. Keith, R. Kobayashi, J. Normand, K. Raghavachari, A. P. Rendell, J. C. Burant, S. S. Iyengar, J. Tomasi, M. Cossi, J. M. Millam, M. Klene, C. Adamo, R. Cammi, J. W. Ochterski, R. L. Martin, K. Morokuma, O. Farkas, J. B. Foresman, D. J. Fox, *Gaussian 16, Revision A.03*, **2016**, Gaussian, Inc., Wallingford, CT.

[10] a) A. D. Becke, *J. Chem. Phys.* **1993**, *98*, 5648-5652; b) C. Lee, W. Yang, R. G. Parr, *Phys. Rev. B* **1988**, *37*, 785-789; c) P. J. Stephens, F. J. Devlin, C. F. Chabalowski, M. J. Frisch, *The Journal of Physical Chemistry* **1994**, *98*, 11623-11627; d) S. Grimme, J. Antony, S. Ehrlich, H. Krieg, *J. Chem. Phys.* **2010**, *132*, 154104-154121.

[11] a) F. Neese, *WIREs Comput. Mol. Sci.* **2012**, *2*, 73-78; b) F. Neese, *WIREs Comput. Mol. Sci.* **2018**, *8*, e1327.

[12] a) P. J. Hay, W. R. Wadt, *J. Chem. Phys.* **1985**, *82*, 270-283; b) W. R. Wadt, P. J. Hay, *J. Chem. Phys.* **1985**, *82*, 284-298; c) P. J. Hay, W. R. Wadt, *J. Chem. Phys.* **1985**, *82*, 299-310.

[13] a) W. J. Hehre, R. Ditchfield, J. A. Pople, *J. Chem. Phys.* **1972**, *56*, 2257-2261; b) P. C. Hariharan, J. A. Pople, *Theoretica chimica acta* **1973**, *28*, 213-222; c) M. M. Francl, W. J. Pietro, W. J. Hehre, J. S. Binkley, M. S. Gordon, D. J. DeFrees, J. A. Pople, *J. Chem. Phys.* **1982**, *77*, 3654-3665.

[14] a) F. Weigend, *Phys. Chem. Chem. Phys.* **2006**, *8*, 1057-1065; b) F. Weigend, R. Ahlrichs, *Phys. Chem. Chem. Phys.* **2005**, *7*, 3297-3305.

[15] a) V. Barone, M. Cossi, *J. Phys. Chem. A* **1998**, *102*, 1995-2001; b) M. Cossi, N. Rega, G. Scalmani, V. Barone, *J. Comput. Chem.* **2003**, *24*, 669-681.

[16] C. J. Cramer, *Essentials of Computational Chemistry: Theories and Models*, Second Edition ed., John Wiley & Sons Ltd, West Sussex, U.K., **2004**.

[17] C. Y. Legault, *CYLview*, Version 1.0b, **2009**, Université de Sherbrooke, (http://www.cylview.org).
